# Supplementary material for: Understanding Fear after an Anterior Cruciate Ligament Injury: A Qualitative Thematic Analysis Using the Common-Sense Model
Source: Int J Environ Res Public Health. 2023 Feb 7;20(4):2920. doi: 10.3390/ijerph20042920 (PMC9957354; doi:10.3390/ijerph20042920)
Supplement: Supplementary file 1 [file ijerph-20-02920-s001.zip › File S1-P1 Transcript.pdf]

<u>P1 Transcript</u>

Interviewer: Hello. So today you will be participating in a study researching fear post ACL injury. This interview will consist of a series of open-ended questions that will explore your fear levels, distress and your confidence after your injury, please feel free to say as much or as little as you want on any topic and if you feel uncomfortable at any time, please let me know and we will move on, okay.

P1: Okay

Interviewer: So tell me about your ACL injury.

P1: Um so, I did my ACL when I was quite young, 12 years old or, so quiet a few years ago now and it was the first game of my football season, 1st quarter and I'd come off a really good season before that as well. So I was obviously very excited about to play and during the first quarter someone came into the side of my knee and I just like remember hearing that distinct pop and like it was very painful. I didn't, I could still walk on it. Like I was able to walk off the ground. But yeah, I was in a lot of pain and I just remember everyone was saying like, you know, try walk it off. Can you move all this stuff and I was still able to move it pretty up and down. I was able to walk it was very painful still so I had no idea. What was happening with it, but as I tried to run and yeah, the pain was still there. I was like no I can't keep playing and then also like that day I just remember going home and like collapsing if I was like going in different directions that I was like, okay well, that's definitely not good and

then we are we finally starting to get all the tests done and then it came back with an ACL and back then because I was so young. I had no idea about ACL ruptures and how like bad they are and how much time off sport you would have? And then yeah, I was pretty lucky because I got the surgery pretty quick because we went to like a private hospital so it was only like three months after I did it I think I got surgery, because I know some people have to wait like years and stuff and then yeah it all started from there.

Interviewer: Yeah, so you remember in that initial period. How did you feel when the knee was collapsing? And how did you feel throughout that initial period?

P1: Yeah. I was definitely, especially because sport was like a massive part of my life, like I played three sports like sport was my favourite thing to do and having yeah, your knees not be able to hold you up. Like it would literally just collapse, very very scary. And yeah, I knew that especially once I started doing research into ACL and the recovery. I knew that it was going to affect me long term, which is also very scary because, any sport like I love it so its very scary and I wasn't happy at all.

Interviewer: So when you were 12, you were doing some research. What what kind of things were you seeing? What kind of messages were you hearing?

P1: Obviously about just how long the recovery was, just to hear that a whole year off, or even like six months of like no movement like that was the hardest thing to us to handle. I feel like and then obviously like the actual surgery wasn't too bad for me and I didn't mind

the actual surgery aspect of it, but knowing that you have to sit on the sidelines and not be able to play sport with your friends. Yeah, that's the main bit, just how long it takes.

Interviewer: So do you remember where those messages of having six months without moving and things like that we're coming from, is that something that people told you or were you more researching independently?

P1: I think I also started to it. So I think it was when I heard a bit more of it through the AFL, and like you might have, Daniel Menzal who has done it like eight times with geelong I'm not sure but I started following his journey a bit as I was going into surgery. And yeah, I know, just seeing athletes going through so much rehab everyday like that's where I got most of it from and he'll post videos of like four months done, just able to move or something like that. And yeah I have mostly gone up from their website ACL stuff on social media

Interviewer: And you also mentioned but it was scary as well. What aspect of it were scary to you.

P1:I knew that after my surgery, I feel like I wouldn't be able to go back to playing sport or just going sport normally because like my knee would be a lot different or it would have like a massive screw in it or all these bolts and stuff in it that just seems scary as in like you might not be able to move it properly so if anything goes wrong, stuff like that. Yeah. It's not being able to play sport with the same mindset and confidence because you know that your knees different.

Interviewer: What did that mean to you to not be able to play a sport at your previous level or intensity.

P1: Yeah. As I said I have a twin brother and we played sports all through, ever since we were little like I played footy, played basketball, played Cricket. I was like the best and fairest when I was 12, like I thought I was maybe going to make this a career, I was gonna have a good chance of doing really well and then obviously being injured I knew that I wouldn't be able to get back to that same like athletic ability as I had before and yeah,

Interviewer: So you felt that you won't be able to get back to that level that you kind of assumed. what kind of effect did that have on your life.

P1: obviously I didn't go back to playing football? Because I was, yeah, too scared of it happening again. As I said before like from watching AFL I see so many players, you see the player is going down and it's like the same exact same feeling that like you felt or hear them say, oh I felt the pop and It sends shivers down your spine or you see it happen, because it's so easy to do, it looks so easy. I was scared to go back so I never… I still go for kicks but I never you know, go back to playing it because I feel like it wouldn't be able to handle the like agility and how much tackling is involved in it

Interviewer: Around that fear or the issue of the re-injury, what would, if you were to go back? What would a re-injury mean to you?

P1: Well, because in 2017 I did my Meniscus really badly and that was going back to basketball and then I also re-injured my meniscus in 2018. So I've already had like some setbacks like obviously not another ACL but some little setbacks and it makes it worse like every time because it's just more time away from the thing that you love and like. Yeah, you just feel you know that each time is even worse than it was before, like not worse, but it's just not going to handle the like the pressure. Because I remember not feeling as agile on it even like coming back from the ACL and yeah, you just don’t have as much belief in it, I guess

Interviewer: So in terms of the things that you love that you have gotten back to, what are they?

P1: Yeah, so I was able to play a few games of basketball like for fun and then I've been able to continue to play Cricket, crickets been like my main sport that I've kept up mainly because like obviously it's not as contact and I feel like there's less pressure my knee which has been good. But yeah even basketball now because I did my Meniscus two other times afterwards through basketball. I feel scared of that sport so that's another sport I feel like I've lost like footy and basketball now, but um, yeah, I'm happy that I've been able to keep Cricket up.

Interviewer: Okay, and that sense of loss, In terms of the sport that you love is there anything else around that sense of loss?

P1: Well also sense of achievement like I thought I could achieve a lot through football and basketball because you know, I knew I was like, all right at sport and I love playing it so like knowing that I could have done well during the sport or I could have played in premierships. So yeah stuff like that. Like I have lost that sense of achievement through these ways. Yeah because I haven't been able to get back to playing them at like a higher level.

Interviewer: And is that had any effect on how you perceive yourself?

P1: Um, yeah, especially like when you hear your friends talking about sport now and then you just can't you can't play, you’re like Oh, well I played when I was 12, and I thought I was good enough then but it's nothing compared to like playing now because everything's changed. Like I don't know if I'd be able to get back to being as agile as I was or as quick as I was because of my knee now, so yeah,

Interviewer: Do you think other people's perceptions of you have changed because of your knee.

P1: Yeah, definitely especially because I did it so young as well like I only had a little snippet of it I feel like and I wasn't able to progress. I could have gotten stronger and I could have gotten better. But I don't know that well, they just yeah everyone else just sees me as like, yeah. I may have been good for a little bit, but I don't know, they don't know what my full potential could have been or what I could have got to as well.

Interviewer: So is in that regard that your perception, has your perception of yourself changed.

12:21

P1: Yeh because I feel like before I did my ACL I was like known as the sporty person and like that kind of came down a bit because obviously I wasn't playing for a year. And then there was another six or so months taken off through my Meniscus injuries so I wasn't able to be that sporty person and always be out training, playing sport, doing well because like me and my brother were known as the sporty twins we both played sport all our lives in the same teams. And yeah that whole perception of this being me being the sporty person, yeah, it just kind of went out the window

Interviewer: How are things now with your brother has he got injured or has he still continued on that sporty identity

P1: He’s still continued. He hasn't really had any major injuries and he's played a few rep football games.

So that's obviously made me feel a bit sad because yeah he's progressed and then I haven’t been able to, but at least with cricket I have been able to progress a little bit, like I am still happy because I was able to still do Cricket and like somewhat progress It's just yeah basketball was like my first love and I was wasn't able to progress in that.

13:42

Interviewer: Yeah, and so you mentioned in that questionnaire we did prior to the interview that you had fear associated with your ACL injury. Yeah, what does that fear mean to you?

P1: The fear of having to go through, yeah, just that long lonely rehab like 12 months of your life because like even though you have so much help and you probably know, like, you've got so much help with physios and everyone around you It's still like so lonely because you can't do the things that you love, you cant, even little It was like, I wasn't able to go skiing for a school trip because you just know in the back of your head, what happens if I do it again, so just having to go through the rehab again is something that's like the first thing that comes into my head because yeah, so lonely and hard, like you don't know, you might get pain one day and that's just fear again, what happens if it's not going properly, what happens if I'm not going to get there. Yeah. It's just the long lonely road of rehab that comes to mind.

Interviewer: How is it lonely, were you supported during your Rehabilitation by other healthcare professionals?

P1: Yeah I definitely was, physios helped me out, like family was obviously there for me, but it's just like you're sitting back and watching your team train or sitting out and watching them play like no one else knows what you're feeling or what you're going through. So you feel

lonely from your teammates or from the cricket Club of from the football club because, yeah, you're just unable to play and be in that team environment. So yeah, yeah.

Interviewer: So did your anyone talk to you about your feelings during your Rehabilitation?

P1: Through the hard times I probably spoke to my mom a few times, I'll be like crying too some nights. Like is it ever going to end or whatever and as much as they help you get through it, it's still hard because I didn't know anyone going… if I knew someone going through an ACL injury that probably would have been better so we could talk with them about it. But yeah, because no one can really understand what you're going through. It's really hard and I love sports so much that they couldn't be at that level. Yeah, so there was help but yeah, it's hard to time for them to understand exactly what you're feeling especially if they haven't been going through the rehab they haven't been sitting in the brace for how many months and stuff. So, yeah.

Interviewer: Okay, and what was your main issues that you wanted to go through when you're talking to your mom about it.

P1: Mainly, obviously during rehab there can be like some hiccups, you don't know what's happening with your knees. So if you're at the right stage to progress, If you're doing the right things, so you feel like you might not get back to the time that you wanted to get back, if that makes sense. Will this progression keep going or will another setback happen? Am I going to, am I not going to be able to play a sport for longer than 12 months, when will it end kind

of or even like I remember some nights, because you are so immersed in it. Like it's the only thing you think about you start thinking, why did this have to happen to me? And it's like a bad trap you get into, but yeah, you just start feeling really sorry for yourself because everything you love is taken away.

Interviewer: And how did you deal with those emotions?

P1: Um, I definitely you just try and, it sounds stupid, but like obviously just other hobbies because the only thing before my knee that I really did for fun. I like had to try and figure out different ways, and yeah bring fun back in because like even wanting football, sometimes it would make me very scared or becuase you would see players go down and that sparks feelings inside you so yeah, new hobbies that werent sport-related or saying to your teammates sorry, I can't come to training and watch, I can't come to a game and watch because it's just not the right environment here at that time because yeah, you miss the game so much so you don't want to be in that environment. Yeah.

Interviewer: So what kind of emotions would say seeing someone injured on TV spark?

P1: Yeah, sadness obviously, because you know the pain they're going through and especially like if it's someone that's done it before, you know they're in for that long recovery and they are going back to that like loneliness its kind of what we said before. It's like your road to recovery and you have to be going in the gym by yourself, so everything yeah, you just feel lonely through the whole process even though you have helped It's just not the same like you

have to get up every morning do those exercises by yourself. It's all up to you sort of, obviously the pain as well because like you remember the pain as well and the feeling of the pop in my leg, that yeah, when you see it on tv its scary.

Interviewer: Was your biggest issue the pain as you're progressing through, as in physical pain, or was it you've mentioned that feeling of kind of feeling the pop, that fear of the re-injury.

P1: I think it was a bit of both like I definitely couldn't say one or the other because during rehab. And after surgery It's very painful as well and yeah the pain that that you felt when it did happen, you still remember but then I know the feeling of the pop as well, it's just such a… it's like I can still imagine it to this day, like knowing that that's had such an effect on my life It's like yes scary to think about that if it happens again I'll have to go through the same things.

Interviewer: Do you still think about it?

P1: Yeh definitely and as I said watching footy and you just see it happened. It just brings back all those emotions and even when I'm at the footy oval because it happened at a local oval, like if am at the place where it happened I still think about it because it just triggers those memories and stuff again.

Interviewer: Yeah. What kind of memories does it does it trigger?

P1: All the emotions through rehab, you just feel lonely because I was so young obviously that's all I was thinking about, I feel like id I did it today or my recent meniscus injuries I was able to deal with that a bit better, but back then because sport was everything I did, it so much harder to handle and I was very like, very emotional through the rehab.

Interviewer: Okay, so you've mentioned some confidence there as well. What did that initial injury do to your your confidence?

23:02

P1: Yeah, well, I definitely didn't feel like I had less confidence because I wouldn't want to go back to it. I just felt my knee will never be as strong or it could so easily happen again because yes such a easy injury like it happened so easily and even though you can get strong enough through gym and Rehab you still never think it's going to be strong enough, as strong as it was before.

Interviewer: Yeah, so what kind of messages did your Healthcare professional team give you around the stability of your knee on going and to improve your confidence.

P1: I remember, I did try and wear one of those braces that you buy from the chemist just to have the confidence in your head and it worked for a little bit. I don't use it now, but I remember yeah, I did use that brace for a bit, so that it's a bit of like psychological confidence to make you feel like yeah, your knee was good enough. It was like what it was before but um definitely for football I could never have that confidence again, like even now I still wouldn't play a game of football which is like crazy, cuz it's a lot of years on from it. But yeah, it still kind of freaks me out.

Interviewer: So what do you think would happen if you were to play a game of football?

P1: Well I would just be scared the whole time, I wouldn't be able to play to my ability or like turn around as quick or tackle as hard because I would be worried that someone would come into my knee and hurt it again.

Interviewer: Did you have those same thoughts with the basketball?

P1: um only a little bit, not as much I feel like it's only football that really has the same amount of fear with it. cricket and basketball I don't have as much fear. I think it was just because ACL was so much more impactful because it's like that 12 months of rehab, when like, because I did my meniscus playing basketball and it's just it was more like a minor injury. I just have a bit more confidence with that than football. Yeah and actually watching the AFL women's, you see so many women going down with ACLS and that just Sparks

more fear because they are like elite players and you see it happen so often so yeah football, I've never had the same confidence.

Interviewer: How would you describe that fear you get when you see other women go down.

P1: Yeah, just hearing that like now I still think about the changes my knees has made in my everyday life. Like if I did it again how much worse that would be, maybe I would have to stop playing Cricket as well. I don't know. It's just like you don't know. It's, without explaining it. You just don't know what might happen. Will you be able to keep playing sport or…Yeah, just the fear about not being able to play as much sport as you want.

Interviewer: So is that your biggest fear, not being able to play as much support as you want. Are you fearful about it affecting anything else in your life?

P1: Um, yeah, definitely not being able to play sport is the main one but I see every now and then like little day-to-day things. Like as I said for the camp, like a ski camp at school, I didn't go on that because I was fearful of that, so there are minor things like that or if you go camping or hiking or whatever like you still have that thing in the back of your head saying this could be a risk as well. Yeah, just like the little risks in everyday life as well.

Interviewer: So when was that skiing incident that you didn't want to go? What or how many years after the injury or how close was that after the injury you think that was?

P1: Two years afterwards. It was still quite a while. It was quite a bit afterwards.

Yeah, I still thought that skiing is a lot of pressure on your knee and even my parents were a bit iffy about it. They're like, yeah, you probably shouldn't because you could reinjure it again. So yeah.

Interviewer: What's been the effect on your parents and the messages that they've been giving to you throughout this injury?

P1: They're, sometimes I feel like they're more like, they'll tell me should you be doing this or should you be doing that? So sometimes they, I hate to say it but they sometimes put a bit of fear back onto me, like even stuff at the gym that I think I'm fine they might be like, should you be doing that and that just kind of reinforces a bit of fear even though they're just doing it because obviously they want me to be safe and they don't want to see me have to go through it again. But um, yeah, they're like, oh should you be going skiing, should you be doing this, should you be doing that? Because they don't want to see me going through it again as well. But that adds to the fear sometimes.

Interviewer: And do you think if their message changed a little bit that would be helpful?

P1: yeah. It's hard to say, like obviously it may but then they're just doing what parents do because they don't want to see me going through it again, but I feel like if I had someone, if I

knew someone who had gone through an ACL injury like mine and they would have had, they would know a bit more about it so more positive, give me more positive like feedback on what to do in those situations. Yeah.

Interviewer: So as you were going through it, obviously that initial phase was super distress, super fearful and confronting, at that middle phase of the rehab about three to four months. Maybe when you started to walk again possibly got the brace off. How were you feeling at that stage?

P1: Yeah, I feel like as the rehab went on I felt more and more little bits of confidence to get back to being able play cricket again, but I still remember, there was the smallest setbacks, like even just like a little bit of pain one day or I think I had some fluid in my knee like six months afterwards and I still felt sometimes I'd go three steps forward but then a couple steps back and yeah, even though everything works out in the end and I was still able to get back, I just remember feeling as though you'd be able to run and then, you still feel like you're missing out and you still you still missing out on a lot so you have like a little win but in the back of your head you're always, Yeah, you're always not as confident, there's such long such a long road ahead of you.

Interviewer: Yeah, and was that your experience at six months or did that experience change or stop when you were starting to return to sport.

P1: I think like at 12 months, a little bit after 12 months I would still have, I'm still having little, I don’t want to say set backs, because they weren't setbacks but like you still have, you might wake up one day and it doesn't feel as normal as what it should feel like and you’ve had another bit of a setback but nowdays or like a couple years after the ACL injury even though I had fear still for football I'm much more confident with like my everyday, like, what I do every day and I'm able to play cricket now a lot so it's definitely improved as the years have gone on.

Interviewer: during that process of the initial year did anyone talk to you at that time about any of the mental aspects of the injury?

P1: Yeah, I think I spoke to a few people that have had like similar injuries after, like a couple of years on and having someone that has gone through it obviously helped mentally, or seeing. There's this documentary and Daniel Mendoza, like I said before he's a football player and following him just to see him go through the same challenges, but being able to… reassuring that your knee is strong and you can get, because like obviously he could get back to playing AFL games. So definitely seeing people or talking to people that have been able to get back gave me some confidence.

Interviewer: Did anything else help you with your confidence?

P1: I think just like playing more games, like obviously the first couple of games for cricket, the first couple games I was still a bit nervous, but getting through seasons without any

injuries. Yeah, you feel more confident and having more time at the gym, even now like heaps of years later. I'm obviously way more stronger and yeah,

Interviewer: Have you noticed that that strength has changed your perception of yourself now.

P1: Yeah a little bit. Yeah although I still feel like I won't be able to be as agile for footy, I won't be able to get back for footy or go to the top of my ability. Definitely for other sports or like even just playing PE at school, playing like little minor games, I'm still confident doing that. Yeah, nowadays. Yeah.

Interviewer: So you believe you're still going to have some fear with football but do you believe you have fear in the current time with anything else?

P1: Um, like now it's not as much fear. It sucks. It's just in the back of your brain like not necessarily fear it's just like hard to explain it. It's just like they're like it's more worrying, like it's not necessarily fear but every now and then you might feel worried that it could potentially that your knee.

Interviewer: And how would you describe this worry that you have?

P1: Um just that feeling of… Yeah, you're just worried that this could lead to another injury or another surgery. Yeah, I know it's hard to explain. It's like worrying is the only way to say it, Its just in the back of the head. Like it's there. It's not like the main thing that you're thinking about while playing sport like it's not your main, its not my main fear, but yeah, it's still there every now and then.

Interviewer: And is this worry been a feature throughout the experience, the injury experience

P1: Yeah, I think so, It was obviously more prominent at the start but now years on from that. It only, I wouldn't say I think about it every day, but every now and then during the week, I might just have a bit of, I might be doing something and start to feel very worried about it if this will have a negative, if it will still affect my knee in any way or like if my friends go for a kick of the footy I'll go and I'll still feel confident as having a kick, but I am still worried in the back of my head a little bit because yes its football, It's the sport that ruptured my ACL.

Interviewer: So if you think of any way that you could improve your confidence or reduce your worry.

P1: I don't know it's hard to answer. If I started training like first of all started training in a football environment like doing some drills my confidence may start to increase then if I feel myself feeling confident or agile or still as fast during trainings then that might make me feel more confident to actually play games. I think that's the only thing I can think of.

Interviewer: And what would being able to go back to football mean to you now?

P1: Yeah, well, obviously, just like make me very happy and like I could play with my friends and see whether I am able to be as good as what I thought I could be. I don't know sounds a bit up myself but yeah, just like not having that what if any more you're able to yeah, like see what could have been or be able to have fun and enjoy playing football and not just watching it, just going for a kick you're able to actually achieve things with your teammates. Yeah, but I feel like playing.

Interviewer: Okay, and throughout any of these process have you discussed your potential to head back to sport or potentially discussing the worry or the fear with anyone? In terms of your like Rehabilitation team or anything like that.

P1: Like as in discuss going back to playing?

Interviewer: Yeah if you were.

P1: Yeah, I feel like my parents and like my career coach now, like they would say to me, don't go back because you know, we want to see you playing Cricket or we want to see you, I feel like I had to say it at the beginning, but there's like added fear from my career coaches and my parents which um, obviously doesn't help me getting back to playing football. But if I

if I spoke to my physio then honestly I think she'd give me confidence and if I speak to some other people who’ve done they’re ACL they might have the confidence. So I think if they've gone back to their preferred sports. They may give me confidence but definitely my parents and my career coach they wouldn't give me that much confidence.

Time: 41 minutes 01 seconds
