# Supplementary material for: Understanding Fear after an Anterior Cruciate Ligament Injury: A Qualitative Thematic Analysis Using the Common-Sense Model
Source: Int J Environ Res Public Health. 2023 Feb 7;20(4):2920. doi: 10.3390/ijerph20042920 (PMC9957354; doi:10.3390/ijerph20042920)
Supplement: Supplementary file 1 [file ijerph-20-02920-s001.zip › File S1-P10 Transcript.pdf]

P10

P10 Transcript

P10: That's fine. That's easy.

Interviewer: Awesome. Well thanks, P10 for taking the time. Today you'll be participating in a study that is researching fear post ACL injury. The interview will consist of a series of open-ended questions that will explore your fear, your levels of distress, and your confidence after your injury. Please feel free to say as much or as little as you want on any topic and if you feel uncomfortable at any time, let me know and we will move on. Does that sound okay?

P10: Yes. All good.

Interviewer: Tell me about your ACL injury.

P10: I got a lot of laughs when I finally went to see doctors about it because I'm a, probably what's considered now a middle-aged woman who thought I could totally play netball again, that would be completely fine. On one of my various health kicks, I played a lot of netball as a kid and into university but hadn't played for a long time so I joined the local primary school moms team. I hadn't had any knee injuries before then. Maybe a dodgy ankle here or there but nothing in particular. I knew I wasn't the fittest I could have been by any means but when it happened, it was really quite shocking.

There was nothing leading up to the ACL that made me think I had to be careful of knees or I should be doing this in a different way. I think the 18-year-old person in my head was in conflict with the 42-year-old person's body, it was doing the things that I thought I was capable of doing at the time. So it was exceptionally painful, I've never been in as much pain.

I've had kids. It was horrifically painful for a short period of time. It was really weird, really short, distinct pain and then it seemed to be okay and I was like, "No, no. It's fine. I've made a big deal of this. I'm being silly," but obviously, I wasn't.

I think what really got me about it was just not having that leed up, not expecting it, feeling slightly more invincible and this has really cut through how slightly invincible I feel, to be honest its made me feel a bit old as well. It's that kind of like, "Ah, maybe I should be doing old people things. I used to have a health kick where I'd go I'd be like, "That's it, I'm going to do running," and I may not look like it but I used to be able to do 5 to 10 K's every couple of days and now it's just like, "Ooh maybe I'm past all of that." It hasn't just affected, say, going back to netball, but it's affected a whole lot of sport and I think maybe over 2020 it's even more so a good excuse.

Interviewer: So--

P10: I probably, yes go on.

Interviewer: I was just going to say what was your timeline, how many years ago? What's your timeline been of your injury?

P10: It'd be 2018 when I did it, so two years ago now. I think that's right. I got surgery pretty quickly on it and then I tried really hard to do the rehab, but I'm not very well behaved, like I'm sure a lot of ACL patients and to be honest, I'm not an athlete. I wasn't someone who was jogging every day. That's why netball was great because netball engaged my head and engaged my brain and I enjoyed the team aspect of it, but I'm not naturally someone who's like, "Oh if I don't go for a run today, I'm a horrible person."

I'm an academic person, I'm not a sporty person as much, so the rehab just felt like a chore that I had to do rather than something that I was really invested in, even though intellectually I knew that shouldn't have been the case. I wasn't really great at that. Having said that about four months, I ended up in Bhutan on a work trip, four months after I had the surgery and we walked to the top of mountains, and I was quite fine had tested it and it was fine. It's weird in terms of fear because the fear is still there even though when I know that I do it, it's probably okay. Does that make sense? It's really silly.

My family and I go skiing a lot, so the first time I went back to the snow I was actually terrified. I was like if I get onto something slippery, I'm going to literally step onto the snow, fall over because I won't be able to keep my balance or it'll slip out from under me or something. The first time I went up I was like, "Yes. Actually, this is totally fine. I can totally do this. I'm less fit and I take it a lot more easy when it comes to skiing but I've done it, but having said that I'm a little bit fearful every time I go and do it. I'm a little bit fearful and it's running probably that I haven't gone back to as much because of that fear.

Interviewer: What does this fear mean to you? How would you operationalize?

P10: Look, I don't know the technical words to a lot of what the doctor explained to me. What's it called when it affects your knee here but your knee can do that a bit? What is that? That's--

Interviewer: Hyperextension knee.

P10: Hyperextension. Exactly. That's what my knee does and even now I'll walk funny in the hallway and it'll feel like it's done that and that is painful and I'm just like, "That's not the knee. I'm sure that's the muscles around it." That's me not being disciplined enough to have

the right muscles working around my knee, it's not the tear, its not the surgery but it freaks me out a little bit every time it happens. The fear--

Interviewer: So. You go.

P10: No. You go.

Interviewer: I was going to say with the pain you mentioned that twice already, what is the pain in relation to your knee? How does that make you feel?

P10: It's not like I've got PTSD and flashbacks but literally before I did the ACL, I'd never had anything like that so my only reference point for that pain is something catastrophic. That make sense?

Interviewer: It does, yes.

P10: When it happens, I don't just go, "Oh, that's me rolling my ankle or something," which I've done a million times before. It's like that could possibly lead to the catastrophic event because I've never had that sort of knee pain before and it feels- I'm trying to think of how to describe it. I think it's because it's that short, sharp, which is the same as the injury. It was short, sharp, and then it went away and you're like, "Okay, I think it's okay now." It's just so reminiscent of a short, sharp version of that and then it takes you straight back.

Interviewer: What kind of stuff goes through your head when you think about being taken straight back to the initial injury?

P10: There's the pain aspect of it, like, is this going to continue but it's also just the being made incapable. I hadn't had any injury before. I've never broken a bone. I've twisted ankles but you just walk on them, it's fine. I've never had crutches. It was so debilitating to have this.

We have a two-story house, a terraced house, the thought of not being able to go up and down the stairs, it's the debilitating aspect of it because it was like going from having everything or my version of everything, playing netball as a 42-year-old to having nothing. It was my first experience and, "Oh, this is what actually debilitated is." I did not like debilitated. I do not like debilitated at all and I didn't want to be that person.

Interviewer: What effect was that having on your mental health throughout that debilitating process, debilitation?

P10: I probably had issues with that elements of self-esteem around health and fitness and body image and all those kind of things, and I've vaguely got that under control because I could one day wake up and go, "That's it. I'm going on a health kick. I'm going to go for a run. I'm going to do these things." I might be a bit overweight, but I'm really fit on the inside, I'm strong. All those things manage that sense that perhaps health-wise, it wasn't the best. Whereas this was like, "Oh no, now you're really screwed." There was no going back to any of those things that you had relied on in the past, to go, "I know it's okay because I'm still strong. I can still do these aspects, even if I'm not the fittest I have ever been," and you just went, "Oh, this is like zero absolutely."

I can't even walk around the block. Also, things like we are a family who goes skiing for example, and I'm not a best skier. I potter around, but I'm like, "Am I going to be the mom who sits inside the apartment and watches everyone else go and do things." That idea of being the observer, rather than being the participant in everything, I think that's what it was like. It was like, "Oh, I don't want to be just the observer, I want to do things in life for a lot longer than this." That's what was scary about it, is that, "I'm too young to be the observer person just yet."

Interviewer: When you say the word scary, what does that mean to you?

P10: The concept that brings about a level of anxiety about being that person, but that anxiety is often situated around points in time when either-- I've got that short sharp pain in my knee or there's an event that I'd like to able to do and now I can't do it. Because to be honest, that anxiety doesn't last the entire time because if that anxiety lasts the entire time, I'd freaking do the rehab I was meant to do, and I would probably be better. It's not an ongoing nagging in the back of my brain the entire time.

It's something which sits there and then pops up every time I've got pain or every time someone says, "Do you want to go skiing?" And I go, "I want to, but is that the wisest move. With skiing, I just have images of helicopters and ski patrol and having to be towed off the mountain because my body went one way and my knee went the other way.

Interviewer: That's one of the best descriptions I think I've ever heard of it. Would you be able to give me that anxiety, that "in the back of the brain," when it comes up? Would you be able to give me that throughout your rehab journey? At what point in the initial phase, how are you feeling then throughout your rehab then at the end stage, what was going on?

P10: Massive at the start. Actually, probably a little bit of denial about it at the very start, I was like surely I haven't done my ACL, that's what athletes do. I am not an AFL footballer this is not happening to me at the start. Then I think the anxiety is lessened when you're under direct medical treatment for something, but when you feel like you've got a team of people around you who are helping you take care of this, and you've got crutches, I had a brace, it's like, "Oh no. I've got things.

When it's good enough that it's just me and it's just me and rehab, that's when I think it gets more heightened in that journey. I think 2020 has played a really interesting role because I

think early on, like I said, I did that trip to Bhutan and we climbed mountains, like really in the Himalayas climbing mountains, and I'm like, "Hey, I'm cool. This is fine, I can do this." Then I went skiing with the family like, "Actually, I think I can do this, this is great." Then I have literally been doing Zoom meetings since March and pretty much this seat and that background is where I have sat for nine months.

It's been exceptionally busy and it's been really hard to schedule. Obviously, anything other than a walk or a run, and run is probably the one thing that causes me a little bit more anxiety. It's that hard pounding kind of thing, I was never good at it to start off with really, now it worries me. I know that the knee pain is worse now in 2020 because I haven't done much.

My trajectory, it wasn't very steep, it was not. It was a very, very slack trajectory, but 2021 made it do this, then slightly like this. I think as it's gone down that way, you just go, "Ooh, that knee does hurt more, because you haven't done anything, you moron." Intellectually, I know why all of this is happening, but it hasn’t changed my behavior.

Interviewer: You mentioned some emotions there, especially the word "worry," what does the worry about your knee mean to you?

P10: I worry that something will become irreversible in a way, in that you'll do something again and there'll be no fixing it this time. There's this guilt with that, I know I screwed up rehab, I know I didn't do what I was meant to do. I haven't made it as strong as it should be and if I do it again, then maybe it's actually just a little bit of my fault that it came out of the blue the first time but actually, maybe it becomes my fault this time and I know I think that guilt plays into that worry about doing it again. It has a messy impact, it's not just me, it has a messy impact on my family. I'm no longer the mum who can do certain things with her daughter. I'm no longer the wife who can do certain things with her husband or with the

family. There are certain holidays we wouldn't be able to do, and I think that those limitations feel awful. If I'm honest, it's probably exacerbated by that feeling of guilt, if it happens again then it's my fault because I didn't do the right things when I was told what to do in the first place.

This interview could be a very good motivation for me to get back and do those things now that I talk about it. Maybe I should explore that, talking about it with someone. When you're by yourself and you're the only one motivating yourself, it's like it's a period of rehab when it's really just you. That's not the easiest thing for someone who's not sportily inclined like me. It's having someone push you along sometimes, hold you accountable.

Interviewer: How would you describe that period of rehab where it's just you?

P10: Guilt-inducing with very little activity. High amounts of guilt, but not actually doing just what I think I'm meant to be doing. It triggers all of those insecurities about me and sport and me and exercise, like "You're not very good at this, you're a bit lazy at this." All of those things that my brain has been telling me for 40 years get triggered in this kind of moment.

It's interesting because my husband's had not an ACL, but he's had other injuries, and he does three weeks of the exercise. And he’s like, "Oh my God, I feel really better." It doesn't happen as fast, it doesn't feel like I'm getting anywhere with anything. That's what the hard thing was, I was like, "I'm doing these things, but it doesn't feel like I'm getting anywhere.

Interviewer: You've mentioned, you're incapable, feeling old, feeling less invincible, no longer the same mom, not as strong as it should be [crosstalk]

P10: Thanks for repeating that back Cam. That's great.

Interviewer: Thanks, sorry. You've got strong theme there, what's it meant to you to just not feel as invincible, as changing your perception of yourself? What's the effect of this?

P10: I think it is very easy and particularly in my stage of life, I guess, so I'm 44. You're transitioning into being an older person, and even without the ACL things are changing and things are more limiting. I'm not the same as I was when I was 20. Though actually, it's a really challenging period in life. One that you are realizing that you are not that young person who is in your head. The 20-year-old is still in your head and up until this point, that was fine because the 20-year old and the 30-year old were kind of similar.

Whereas there are physical things happening now which are really challenging, "Oh, you're not the 20-year old that's in your head anymore." I think regardless of the ACL, a whole heap of things are happening at that period of life, and then you add the ACL onto that and it's just a really poignant, extreme example of that. And a demonstration of potentially where you're headed. You're there, you can't move, you can't move your leg, you're in a lot of pain the entire time, you can't really get up and down the stairs very well. You're like, "Oh, so is this what it's like to be 70 or 80." It's one of those things where it's like, "Oh, this is the future being shown to me which I have been ignoring to the entire time." I've been ignoring that I've been getting older. I've been ignoring that I'm more limited in things, because you can. This is a reality thing where you just go, "Oh, this is what it would be like to be far more disabled, but also just far older as well." It was a couple-- Now, how long was it? It would have been three or four months after the surgery, we walked into my new house, we walked to the place that was like a townhouse that was four levels. It was the first time I've ever been into a place where I'm like, "Oh, actually these stairs are challenging, and with my knee would I want to do this every single day? Is this the right thing, but also if I did my ACL again, is this the right--"

I've never had those sort of limitations because of my physical capacity put on every much anything before. The only thing else that plays into that fear is-- I remember my surgeon saying that 'once you've had one ACL, you are much more likely to have another'. Even if that one heals, there's this feeling that something might happen to the other one. It's like, "Oh, yes. I had my first ACL when I was whatever and now I've had four," and you're like, " Shit. If my surgeon who looks like he should play AFL, cannot stop himself from having four ACLs, I'm screwed."

I think in the back of my mind, regardless of what I did, there was a bit of an inevitability about it. The way to avoid the inevitability of an ACL is to go, "Well, I won't do those things which would produce another ACL injury because actually, just doing all of the work around it doesn't help people who are really fit, why on earth would it help me?"

Interviewer: What then would another ACL injury mean to you?

P10: Oh my God, it would be so depressing. Although I need a break from work at the moment, so you never know there could be some upside if I just had to stick around for a little while. Oh my God, it would be awful. At the moment, I've got one good leg. I've got one good knee. If I go skiing I at least know that 50% of me works the way it's meant to. I really think something like skiing, I'd just be terrified that if both legs potentially were fallible that it would be almost too terrifying to do.

Interviewer: You've mentioned--

P10: I've set an aim to try do more walking and do one of those couch to 5k things, but I'm like if I did an ACL tomorrow that would just be-- It just seems like so far off in the distance and it'd be like hopelessness or what's the point. The ACL reminds me a little bit too. It makes me feel like I've got limited time to get this whole fitness health thing sorted. An ACL

sucks up a good six to 12 months of that time minimum. In whatever plans I might have for getting myself together, you might as well write off 12 months of that. I don't have enough 12 months left while I'm motivated and I'm feeling youngish. It sucks time out of that capacity to get yourself together.

Interviewer: That's a really interesting point, actually.

P10: I'd be fascinated to know with these kind of interviews. I do wonder about time of life and what it means in particular point of human development really, what it does for a teenager versus what it does for someone in their 20s. I'm a social worker, I'm all into context around people, but that context of where you are situated in life before this happens, not just for the physiological perspective but psychologically and in terms of just your lifespan trajectories, it triggers those things that are going on anyway I would say.

Interviewer: It would be very interesting to see what comes out of it. You've mentioned terrified twice, particularly around the skiing. What does being terrified mean to you in regards to this?

P10: When I picture skiing and I picture it all going wrong-- You need to know too that skiing is something which I do and I like, but actually has a level of fear factored into it already, so I came to skiing quite late, "My husband is really good, I'm really not." I try to keep up as best I can, but I'm slightly scared the entire time I'm doing it because I don't quite know what I'm doing. The image is, you hit some sort of bump and literally your knee goes in that direction, the rest of you goes in this direction and you're stuck on the middle of a ski run on the top of a mountain in agony.

Its also, Being on the netball court, I literally fell and was screaming. It was that much pain. I couldn't hold it in and I just made a big fat fool of myself. People came running and were

like, "Oh my God, what's--" I'm just so embarrassed as well because the pain then goes away and I'm like, "Oh my God, I'm so embarrassed, this is appalling. What am I doing? Shut up. Just get off the court."

With skiing, it's a similar thing. It's this intense pain but then it's like, "Oh my God, I'm stuck on a mountain, the ski patrol are going to have to come, I'm going to have to be lunged down, everyone is going to be watching as this stupid woman who shouldn't be skiing in the first place is carried off the frigging the mountain." There's a level of pain-fear, but there's a level of embarrassment-fear about it as well, which I do find embarrassment slightly terrifying, to come back to you're original question.

Interviewer: The initial injuries come with a level of attachment to not only the pain but also to the experience. What kind of effect has this experience uncoupled?

P10: It plays into a lot of stuff I've talked about. I was too old to be playing netball anyway. I was too unfit to be playing netball anyway. I was too fat to be playing netball anyway. What the hell was I thinking, that was a stupid thing and now look, you're on the ground screaming and everyone's looking at you going, "Oh look, she was too unfit to playing netball." It triggers into all of those kind of things. Now, when I think about running for example. Before ACLs, I went for a run one day and I stepped on a stick and I twisted my ankle and I fell over. It was terribly embarrassing.

We're talking bloody knees, I was like a child. Bloody knees, and bloody hands. I couldn't get up probably because everything I wanted to kneel on to get up was sore. This very nice gentleman came and helped me up and I'm like, "Oh my God, that's the most embarrassing thing of all time. This middle-aged person who was running had to get someone to help him off the ground." It triggers all of that, it's like you're too old to be doing this kind of exercise,

you're too unfit to be doing this anyway. If you then injure yourself while you're doing it, it's like, "Oh my God, look at the old woman who's injured herself." Of course, she has because she's old; she shouldn't have been doing it in the first place.

As I say those things, I know that irrational and I know they're not true. I don't know when he's actually giving a shit about what I do. When I run or ski, no one is paying any attention, but it triggers those insecurities that are probably long-held insecurities of mine, anyway. They're very deep-seated insecurities, which means that the going through rehab, the intellectual process of what I need to do, doesn't actually solve those insecurities, or doesn't address how those insecurities play into the ACL in any way. Even if I've done all the rehab, I can guarantee you I would still feel that way because they're triggering something in me that was before the ACL even.

Interviewer: That ties in maybe to a lot. The ACL has contributed to a lot of how the perception of yourself has changed after this injury. Do you think the way that other people perceive you has changed at all because of the injury?

P10: I worry that it has a little bit, I don't want to be seen as the person who can't do things as opposed to the person who can do things. I definitely get a lot more questions, "Oh, are you going to be all right with this? Will this be okay for your knee?" I'm just like, "Yes. Look, I'm here doing it." I get annoyed, l don't need anyone babysitting me, asking about it. It definitely is a factor in what people ask about. [crosstalk]

Interviewer: You go.

P10: What it does is it brings into sharp focus, how people might perceive you. Without an event like that or something that they're commenting on, you don't get that impression as

much, and because you don't get that impression as much, you can pretend they think about you in any way you want to think they think about you, if that makes sense.

Interviewer: It does.

P10: You make up a narrative in your own head about the way you're perceived by other people because you never really know, so you make up that narrative yourself. This kind of event and those kind of comments go, I can't stick with the narrative that I had because it is showing to me that I think about me in a particular way. It just brings that into a bit more sharp focus.

Interviewer: What kind of effect does that narrative have on your confidence?

P10: My confidence tends to come from denial. Ill just pretend something hasn't really occurred and if I'm completely confident about it and it's fine. If someone keeps just asking you that question and brings you back to the thing you're trying to forget ever happened, that I don't like as much. It's not to say that it's a bad thing, it's probably something I should be more aware of. It's probably a trait of, "I'm going to go on this health kick, I'm going to pretend I have not been unfit for such a long time.

I'm just going to pretend I'm fit now and do stuff, just how I was. Whereas this is like, you can’t pretend or be in denial about the reality of something that happened as much. I find that frustrating, it's just not my normal way of proceeding with things.

Interviewer: Then is that in regards to your confidence for the whole ACL injury experience then?

P10: Yes, it does. There are times when I've been doing okay with rehab and I feel like I'm getting back on board with things. Then someone would say, "Are you okay?" "Yes." It

makes you feel like you should not be okay with it, and you see something that I can't say that makes you think I'm walking funny. At the University of Melbourne, the social work department with the physio department, there was nothing better for one's confidence than coming back to work and finally being off crutches and actually walking and you think you're doing fine and people going, "Oh Jen, that gait is not good. You should not be doing that. Oh, what have you done to yourself?"

I just want to pretend that I'm walking and walking is good. Walking is an achievement rather than have someone tell you the whole time that there's still stuff that's really fundamentally wrong and it's obvious. It's obvious to everyone who looks at you.

Interviewer: With the exception of them, throughout your journey, did anyone discuss with you, or any health professionals specifically, discuss with you any psychological considerations throughout the initial injury or rehab or surgery at all?

P10: Nope

Interviewer: What about those messages from the surgeon, the surgeon you mentioned that he said that he had four. What effect did that have on you, hearing the surgeon messages?

P10: It made me nervous about my other leg in a way that I haven't been nervous about my other knee at all. I think you break your arm, you don't then start going, "Oh my God. I could break my other arm." Whereas I walked out of that and went, "Oh my God. I could just as-- It felt like there was a much higher possibility of doing that or of doing it again. Even though the surgery was really, he's like, "Oh yes. They're really strong, but we don't know what it is."

People are just far more likely to have more ACLs once they've had one ACL, it's like, "Cool, okay. ACL was not even on my radar two days ago and now suddenly I'm in this high

possibility group." It definitely increases the fear about that. I don't think there was a great linkage between the surgeon and physio about why that's the case or what would I do to avoid that being the case or who is that the case for? Because it was like, "What's he talking about?" I think in your brain, you can go, "Oh, if you're a really active person, therefore maybe really active people are more likely to do an ACL twice. Maybe I'll just be a less active person, then I'll avoid that possibility."

There really wasn't the linkage into physio or into more general health around what does that mean. And not being rude, I was what, 42, definitely overweight. There's a whole lot of health stuff around me that in hindsight really could have been picked up at that time in conjunction with the knee injury, rather than seeing it as a completely, simply biomechanical kind of thing. Like, "Why was a 42-year-old playing netball? What does that mean? This is an overweight person and that is not good for this kind of injury recovery, but what does that person need in order to resolve--

That whole holistic. It felt very biomechanical, if that makes sense, like this is just about the knee, the rest of you doesn't exist, just the knee exists.

Interviewer: You've mentioned, you've got, if you don't mind me saying, quite a strong self-blame causative message there for the whole thing. Then earlier you mentioned that you hadn't had any previous problems in general and you could just go play sport. How do you view the whole experience in terms of your emotional state? I'm trying to vocalize this. How do you view that self-blame? Where does that come from or is that how you view direct cause of your injury? Because you had previously been good and you didn't just suddenly whack on 20 kilos and change your whole body before you jumped on the court, so where's this?

P10

P10: Long history of always being that slightly overweight teenager. Still good at sport, but never the fittest person, never the skinniest person. Definitely never the fittest person more than anything. It's a long-seated kind of thing. Those really stereotypical teenage girl things that never get let go really. It wasn't a case of surprisingly putting on a lot of weight and then suddenly stepping onto the netball court, let's say. It's just always how it's been. I think it's always made sport challenging. Sport's fun and I really enjoy it, but I've always had that idea that people are looking at me funny when I do it, that there is a lot of judgment around doing it because you don't look exactly the way you're meant to look when you're on a netball court or when you're running. That's into teenage-hood. That's very long-held.

Interviewer: Will you return to netball? Do you think that your confidence in your knee can improve?

P10: I think of all the sports I could do, I think netball would be a really dumb ass choice. Really fit people do their ACLs all the time. It's a ridiculous sport. Why would you catch the ball and stop? It's stupid, stupid as a concept and as much as I loved it, I really do not trust and I actually don't trust the 20-year-old who still lives in my brain. Just stop being competitive about it, just stop trying. I could not play relaxedly and it doesn't matter how fit I am, I do not play in a relaxed manner. I play in a very competitive manner and my body pays the price so I don't think I would go back to netball.

I really want to try and improve my fitness for skiing because it's a family activity that I don't want to give up on. I would like to get back into running if I could because I think there's more of a sense of personal achievement with that and I can moderate myself in doing that a little bit better.

Interviewer: You think your knee function will improve or can improve in general?

P10: I think it could but I think there's a lot more to it than just my knee, do you know what I mean? If you took the knee out of it, I would still be on a path of trying to improve those things. They weren't things that I had and I lost because of the knee, they were things that I've always struggled to have and to obtain and I struggled to have a level of fitness and I struggled to regularly do those sort of activities anyway. The knee just totally exacerbates the struggles that I was having before.

Interviewer: Did you employ any strategies to deal with the psychological struggles throughout your injury journey? What kind of things helped?

P10: You know that word denial? That's probably the key strategy. I'm not very good at these things. Intellectually I know what's going on but I'm not very good at doing it differently.

Interviewer: Could you be better? Could your fear and distress at this point in time be improved?

P10: Yes, potentially. I think what helps with the fear is proving that it's not true because fear is built from not knowing whether it's possible or not knowing whether something will occur and that then stops you from doing things because you don't want to test whether or not it's not possible. It does have to be that combination of having a good enough emotional state to actually try the thing at a level which then proves that you can do it. I think for me fear is dissipated by incremental displaying to myself that it's possible. Friends and I went for a big long walk just a couple of days ago and I'm like, "Actually, that wasn't too bad. I totally did that," so that helps dissipate fear.

Intellectually knowing that I could do that and that's stupid, of course, I could do that, didn't work at all. It's this combination of the physical, actually getting out there and doing stuff and

testing what you can do but also trying to fix those emotional little barriers that you have actually as well.

Interviewer: I'll throw just a bit of a curveball in there then. From your experience, how do you view your ingrained fear maybe subconscious versus that fear you just described which maybe is a bit more fear that you processed and thought about? Where do you view your knee in terms of the trauma and things?

P10: I think the knee definitely triggers those ingrained fears around embarrassment and self-image and what I should be doing and that it's your own fault kind of stuff. It definitely triggers those kind of things. No, I take it back. The thought of having another ACL injury triggers those ingrained ones. The thought of my knee hurting or not being quite capable on a walk doesn't trigger those as much. Does that makes sense? It's the fear of the catastrophic injury rather than the fear of going out and the knee being sore, or the fear of going out and having to limit myself in some way. That I can get over but I think it's the catastrophic nature of an ACL, it triggers the deep-seated stuff. Is that what you're getting at? I'm not sure if I've answered your question very well.

Interviewer: That's good, thank you. The catastrophic nature, that's just fear, you've probably explained it very well, but give me a bit of a background on what that catastrophic nature entails as a synopsis.

P10: It's that whole combination of being unable to move, being absolutely and utterly reliant on other people, which I really do not like and enjoy. Having to have attention drawn to you, then actually you have emergency services and things being out of your control, that idea that you're on the ground and other people are panicking and calling an ambulance on your

behalf. You're not in control of your body, you're not in control of what other people are doing around you so that catastrophic nature.

Then when you come home, you're not in control again of your body. You can't make it move the way you want to make it move. You're reliant on other people and I hate being reliant on other people. I really do just want to be reliant on me.

Interviewer: Extrapolating on that, is it fair to say that maybe those feelings of helplessness, for want of a better word, is an extreme driver of maybe you’re thinking about returning to think of the helplessness is a--

P10: Yes, because at the moment, I may not be completely capable it might hurt in different spots but I am totally able and in control of what I'm doing at the moment. I might be a little more limited but I'm not reliant on anybody. I can climb the stairs, I can get there. I don't need to be able to go for a run, I don't need to be able to play netball to show you that I'm capable and reliant on myself, but if I go and do those things, and push myself, I will become reliant on other people. I don't want to push it to that point.

I really hate to do this but I'm going to have to go into another meeting. Is that okay?

Interviewer: That's fine. Sorry, you've been amazing.

Time: 51 minutes 07 seconds
