# Supplementary material for: Understanding Fear after an Anterior Cruciate Ligament Injury: A Qualitative Thematic Analysis Using the Common-Sense Model
Source: Int J Environ Res Public Health. 2023 Feb 7;20(4):2920. doi: 10.3390/ijerph20042920 (PMC9957354; doi:10.3390/ijerph20042920)
Supplement: Supplementary file 1 [file ijerph-20-02920-s001.zip › File S1-P11 Transcript.pdf]

P11 Transcript

Interviewer: Hi. Today, you will just be participating in a study that is research into post ACL injury. This interview will consist of a series of open-ended questions that will explore your fear, levels of distress, and your confidence after injury. Please, feel free to say as much or as little as you want on any topic. If you feel uncomfortable at any time, let me know, and we'll move on. It sound okay?

P11: Yes, sure. It sounds good.

Interviewer: Tell me about your ACL injury.

P11: I injured my knee when I was probably about 16, 17 years old in a motorbike accident back home in India. Then I remember getting some X-rays done back then, but I don't think I ever got an MRI done, so it was never diagnosed. Then I used to play basketball at my school, and after I had my accident and my knee healed, I stopped playing basketball as well. I didn't have any trouble walking when there was no pain or anything from my injury. I always knew that I had a bad knee, but I just thought that it's due to my accident, and that was it.

I just accepted it as it's always going to be a bad knee. Then a few years down the line, I've had incidents where if I was, say running or dancing or doing some movement and my knee gave away. Again, I would wear my bandage for a few days, and then it's back to normal. I've played sports; I went to the gym. I've had a very normal active life, and it's never been a problem up until recently when I was just standing at work and my knee gave away while I

wasn't even moving. That's when I was like, "Okay, something's wrong. I need to get this checked."

When I went to the GP, and they just did some movements and stuff, and they're like, "Oh, I suspect an ACL injury." Got an MRI done, and that's how it was diagnosed. When I saw the specialist, he said that it's quite common. It's not painful, and just that your knee will feel a bit unsteady, but that's pretty much the extent of it. Since the last time it happened, it's been getting worse, and I can feel that it's a lot more unsteady than what it used to be. That's where I'm at. I'm looking to get surgery next year.

Interviewer: In the questionnaire, you also mentioned that you had a little bit of fear associated with the knee, what does the fear mean to you?

P11: In the sense that I'm safely going skiing, like if my partner is going skiing and then I normally do sports, but I would tell him, "Oh, look, I don't think I want to go skiing because I've got a bad knee, and I think I'm going to end up injuring it." That's the kind of fear I was eluding to. I'm not sure whether it classifies like fear, or is it called something else? I was always scared of hurting my knee, and then that's why I often didn't do a lot of things or took it easy, while I was doing certain things.

Interviewer: What things, or what situation would make you fearful or worried?

P11: It was mostly sports and, say, if we are traveling, then we often did adventure activities like skydiving or we did canyoning or just things like that that could potentially injure my knee. I was always very careful when I did things like that. It's always been in the back of my mind that I've got a bad knee, and I need to take care.

Interviewer: What would be the feeling, or how would you feel about being careful about it? What would that mean in practicality?

P11: Since actually knowing that it's an ACL injury, so now I don't go running. If say, I'm walking my dog, I always have my bandage on. If I'm turning around, my knee is a lot bigger, so if I'm turning around, it gives away if I'm not careful. Just things like that. Because it's an everyday thing, it becomes part of my normal movements as well. Even my muscles on my left leg, my left knee is got the torn ACL. My muscles on my left leg are a lot more tenser than on my other leg.

Interviewer: With the muscles being tenser, is that something that you think about or consider a lot?

P11: Yes, because it has ended up being painful at times. I've got some-- I don't know or not, but I've got pain from my hip to my knee. It's because of my knee and how I-- My muscles are always very tensed. Because it's been happening for over a long period of time, it's catching up.

Interviewer: What's the effect of the pain on you and your everyday function?

P11: I wouldn't go to the gym. I wouldn't do certain types of exercises if I had to walk to and from work. I'll make sure that I walk only once a day and not exert myself or go on hikes, which normally I wouldn't have thought much about.

Interviewer: How does that make you feel, not being able to participate in those activities?

P11: I don't like missing out, so I don't really like that. The other thing is this is something that could have been fixed a long time back, just because it wasn't diagnosed.

Interviewer: I lost you there. Sorry, you just cut out for a sec.

P11: Sorry. Hopefully, you'll be able to hear me better. It's that regret that goes with hindsight and thinking that if this would have been diagnosed better or diagnosed earlier, then it would have been a lot easier for me to do normal things.

Interviewer: What was the message from your health care professionals at the time?

P11: As I said, they just did an X-ray, and I didn't have any broken bones. That was basically it, and they were like, "All right, don't exert yourself too much for the next few weeks." Once my knees healed and I was able to walk, it was just back to normal. Because this feels like-- a while back, I don't think MRI was as common back then as it is now. Even if you've got a bit of a doubt, you'll probably just get an MRI done. Whereas back then it was supposed to be a big deal, but I was a teenager, and I probably didn't know enough as well. It was more like what my parents decided to do at the time.

Interviewer: At the time, how did you manage the psychological aspects or the return to usual activity?

P11: It was that big a problem back then. After I had my injury, I was playing basketball on my school's team again. It didn't really affect me back then, but over time, as my knee gave away, every couple of years, every time, it just kept getting worse and worse.

Interviewer: What's the effect on your fear or your distress throughout this whole process of it just giving way?

P11: I was more distressed about not knowing rather than about the ACL because one, up until now, I didn't even know what an ACL is. It's just a ligament for me. After being diagnosed with a torn ACL, that's when I started doing some research and I was like, "Okay,

Oh, this is pretty common, and this is where the ligament is, blah, blah, blah." Up until then, it was more like just not knowing and just accepting the fact that there's something wrong with my knee, but it wasn't anything major to require medical attention. Just that sense of not knowing that was causing me distress.

Interviewer: How do you define, or how did this distress manifest itself?

P11: In a way, it probably affected my confidence in some sense. If I was skiing, I would be like, "Oh, I can't ski." I wasn't able to do it because maybe I had a bad knee, and if I try it again after I get my surgery, it would be different.

Interviewer: You've mentioned bad knee a couple of times. Where did bad knee come from?

P11: As I said, because it wasn't diagnosed, I always thought that, "Oh, it's a bad knee because of the accident, but it's nothing serious."

Interviewer: Did that have any effect, that kind of mentality that this is your bad knee, your good knee?

P11: Yes. I will always use it as an excuse if I thought that I couldn't do something physical. Say, if I've been on a long hike, like 15, 20 kilometers hike, at the end of the hike, I knew that my knee was hurting, and it wasn't normal. Then I would just say, "Oh, I've walked so much, and I normally don't walk this much. It's probably because of my bad knee." Not realizing that it's actually a torn ACL.

Interviewer: Did you have any other feelings or did anything else occur because of this injury or having a bad knee?

P11: I don't think, not unless it was diagnosed. A bunch of that, it was always just a bad knee and nothing more than that.

Interviewer: There's nothing else you missed out on?

P11: Sometimes I do think that's why I can't do these things because I've had an accident when I was younger, and I should have been more careful back then, just all the usual stuff. Apart from that, I never knew that it was this serious, and it would require surgery.

Interviewer: You didn't have any issues in terms of feeling ongoing distress about the function of the knee?

P11: Not in the day-to-day. Actually, when I went to the gym, I wouldn't do box jumps or things that required my knee. I always told the trainer, "Look, I've got a bad knee, so I'd rather not do those." The coping mechanism was basically avoiding what I couldn't do rather than trying to fix why I couldn't do that.

Interviewer: Did any health professionals or anyone ever asked you how you were feeling about the knee?

P11: Not really. It was more about, "Oh, is it painful?" There were more questions related to the knee rather than how I was feeling about it.

Interviewer: You mentioned that you looked up the ACL online, what messages were you getting from online?

P11: It was more the technical side of things that I looked up, as in where the ACL is, what the function of the ACL is, and how it's common to have the ACL being torn. ACL reconstruction surgery is really common among people my age.

Interviewer: You've said you have discussed with the surgeon already, you've got it booked in for next year?

P11: I've already met with a surgeon at the Alfred Health, but I'm waiting to meet someone in the private health. To be honest, my experience so far has been so much better with Alfred Health than with the private health.

Interviewer: What kind of messages have they told you in regards to your knee?

P11: Just telling me that it's okay to have this thing-- Normally, you would associate a torn ASL with someone who's older. My sister and my brother-in-law, they're both doctors. When I found out that I've got a torn ACL, obviously, I consulted with them. I was like, "What do you think is the best step forward?" They were like, "Oh, surgery is probably going to be an option but best to see a knee specialist, an orthopedic surgeon." They suggested meeting someone who works with younger people compared to someone who does knee replacements on older people.

That was my criteria in picking out a surgeon that I wanted to go to and then just asking them what they've done in terms of working with past patients and what's the time of recovery and what's involved in the surgery basically. The surgeon explained to me what they were doing in surgeries. I would cut out a hamstring from the other leg and then just put it in my knee. It will be like-- what's it called? Arthroscopy is the word?

Interviewer: Arthroscopy surgery, yes.

P11: Arthroscopy, yes. That's basically not very invasive. It was more like just explaining what the whole process was going to be like. Then my questions were more about what the recovery is going to look like and how it's going to affect my day-to-day after the surgery. I

was told that it'll take up to a year for recovery, and that would involve physio and no running, ect cetera, for the first few months.

Interviewer: How did you feel about all those messages?

P11: Personally, I like knowing what's coming up, so I did like that. It helped me prepare myself for what to expect, and at the same time, just knowing that this is a common occurrence in people my age gave me a bit more confidence in just thinking that something drastically is not wrong with me. This is quite normal in a sense. It just took out that bit off-- It's like, "Oh, you have to get a heart surgery versus you just get to get a ligament restructure, preconstruction." It's just the intensity of the surgery was taken out of it for me. I feel a lot more confident going into surgery now than what I was feeling before I met the surgeon.

Interviewer: Oh, good. How distressing was the messages from the surgeons then?

P11: After meeting the surgeon, I was feeling a lot better. Then, of course, because my sister and brother-in-law are both doctors. They had told me that this is most likely what's going to happen. I reckon it's a cultural thing as well. When I spoke to my mom, and I'm like, "Oh, I might need to get surgery." Getting surgery is considered a very big deal back in India. She's like, "Oh, my God, something's going to happen to your knee." That panic set in for her. I'm the one who's trying to tell her, "Mom, it's nothing major. I could have my normal life back, even better than what I'm having right now." That communication from my family, while they're supportive, but at the same time, because of lack of awareness rather than education for my parents, that was a cultural factor as well.

Interviewer: When you say, "Get your normal life back." What's that going to mean to you?

P11: As in my knee is not going to hurt if I walk 5 kilometers or something. I don't play any sports or anything as such. It's more like hiking and that odd adventure activity that I might do once in a while. Just going to the gym, that's my extent of physical activity.

Interviewer: Currently, if you [crosstalk]. Sorry, you go.

P11: Sometimes if we're going away for the holidays, then if it's winter, we might go skiing or snowboarding. If it's summer, you might go to the beach, do some water sports. I would be able to do all those things without the fear of being hurt or injuring my knee.

Interviewer: Currently, do you have that fear of being hurt?

P11: Yes.

Interviewer: What's your response normally when you do movement that--

P11: I just don't do it. I just avoid it. Ever since I found out, I don't do any running at all.

Interviewer: Ever since you found out that you had a ruptured ACL since the last twist.

P11: In the last couple of months, two or three-- I found out in probably August or September. Since then, I haven't been doing any running, whereas, since the last 10 years, I've just been doing whatever I felt like.

Interviewer: What made you stop to run specifically after this last relapse episode?

P11: Just the fact that I know that something's wrong with my knee and what exactly it was. I know that running could harm it further if I sprain my knee again. Just knowing that it's not going to be good for my knee. That's what made me stop doing these things. I still do walk a fair bit, but yet my knee hurts. Then I just take it easy.

Interviewer: Again, these feeling, these beliefs you've got, this is all a response to you looking it up or there are other influences that you've drawn on to?

P11: Combination of both. Just having that awareness made me a lot more conscious about what I'm doing and how it could affect my knee versus just thinking it's a bad knee, and I should just take it easy. I've been a lot more conscious about not doing certain things now.

Interviewer: Tell me about how this has had an effect on your overall well-being?

P11: Generally speaking, I'm a very anxious person, so I was a bit anxious in the beginning. Then as I did more research. My research told me that it's fine, it's not a big deal, I'm not going to die or anything. It's just a minor surgery. Just knowing about things has helped me cope with it. The not knowing was making me anxious. Before the time I saw the surgeon, I was a lot more anxious than after.

Interviewer: What was this anxiety? What kind of things were going on?

P11: It was just like, "Oh, what is wrong with my knee? Will it be okay? Will it not be okay? Will I be able to go to the gym? Will I be able to go on hikes with my friends? Will I be able to take my dog for a walk?" Just little things like that. Because we're discussing about it right now, I'm thinking of them, but that's what was going on in my mind, even though I might not have paid particular attention to it at the time.

Interviewer: In reflection, you're thinking these things were going on a bit. Do you think your own perception of yourself has changed because of this injury?

P11: I wouldn't say so. After I have my surgery and after I finish my rehab, my answer to that question might probably change.

Interviewer: In what way?

P11: It's like a challenge then you learn something, and you overcome it. You grow in a certain sense.

Interviewer: How has maybe other people's perception changed of you because of the knee injury?

P11: What do you mean?

Interviewer: Do you think anyone else's perception of you has changed due to this injury?

P11: Probably, my parents. Because they're not here and because of lockdown, it's just exacerbated. My mom keeps on feeling that she's not here to take care of me, even though I'm 30, and I don't really need my mom to take care of me. I live with my partner, but she still feels that-- and I don't know whether that's what you mean by perception. She still treats me like I'm a kid who doesn't know what I'm doing. It's like, "Oh, it's surgery, it's a big deal. Are you sure you've talked to the doctor, are you sure you've asked him all these questions?" It's one thing that I've noticed is that my mom started treating me like a teenager, just because she isn't here to take care of me. That's how she's feeling.

Interviewer: How does that make you feel?

P11: Knowing my mom, I expected that. I'm fine. I know my mom is even more anxious than I am. That's quite expected behavior from her.

Time: 25 minutes 36 seconds
