# Supplementary material for: Understanding Fear after an Anterior Cruciate Ligament Injury: A Qualitative Thematic Analysis Using the Common-Sense Model
Source: Int J Environ Res Public Health. 2023 Feb 7;20(4):2920. doi: 10.3390/ijerph20042920 (PMC9957354; doi:10.3390/ijerph20042920)
Supplement: Supplementary file 1 [file ijerph-20-02920-s001.zip › File S1-P12 Transcript.pdf]

**P12 Transcript**

**Interviewer:** Let me submit that one, and that one. All right. I'll just do a bit of an intro. Today you'll be participating in a study that is researching fear post-ACL injury. The interview will consist of a series of open-ended questions that will explore your fear, levels of distress and your confidence after your injury. Please feel free to say as much or little as you want on any topic. If you feel uncomfortable at any time, let me know and we'll move on. Does that sound okay?

**P12:** Yes, that does. Yes.

**Interviewer:** Awesome. Tell me about your ACL injury.

**P12:** I actually, long, long time ago, I had dislocated my shoulder and I had done a full shoulder repair. I went back for my first season returning to sport with my shoulder, so I was fine. Played what I thought was my best season. In the grand final, I did my ACL, it was devastating. I had no idea what was happening. I had never hurt my knees before.

My shoulder was fine, recovered from that. Never had any further issues, but my knee. It was out of the blue, playing just social netball when I think I was 26 or 27 at the time. I wasn't at an age where I was too old to recover from it. Definitely not. It was just one of those things. It just happened out of the blue. I thought everything was fine. I thought it was just that I'd hurt myself, but I was like, "I'll be all right." I'll have a break and I'll keep playing. Then standing on the sidelines, trying to do the little run and did a little jump, and almost threw up. It made me feel sick.

I had to go lie down and still went to drinks after the game. We lost terribly, but not because I wasn't there. Yes, it was such a shock. Then, to find out I needed surgery because I had had shoulder surgery a year before. Again, I had a great physio, fully recovered from shoulder. Then everyone had said, when I was having my shoulder re-fixed and everything that, "Oh, at least it's not a knee." I was like, "Shoulder is pretty hard work."

Shoulder was pretty full-on to recover from, but now I can see what everyone meant because starting to get my knee fixed, yes. At first, my physio didn't think it was that bad. She was like, "Oh, it's probably just--" I can’t remember what she thought it was. Then a week later I called her. I was like, "I can't even walk today. It's pretty bad." We went for scans and everything and she was like, "I'm so sorry, it's a full ACL tear."

I had a full replacement, don't know what it was called. I did all the physio. I did all the rehab. I went and saw an exercise physiologist. They both said I was fine. Tried to go back. I tried to play a game of netball. Running in the gym was different. Going on a treadmill was different. All my exercises were fine, but when I tried to actually run and play a game, it was definitely a mental block.

Also I felt like something wasn't right still. I was like, "My knee is definitely not the same." I went and got more scans and there was a pretty significant meniscal tear. I had to have another surgery to fix that. I did another round of the physio and all the rehab, not as much. The second surgery wasn't as bad as the ACL recovery. I did a lot more than they-- They kept saying I was fine and that I should go back, but in that time I also then started having kids. I just kept using that as an excuse.

My physio, I started just doing Pilates. Didn't have any problem doing other exercises, gym work, walking, Pilates. I just got addicted to doing Pilates a couple of times a week instead of

playing netball. I've since had two kids so haven't been back on the netball court. I ended up having my second surgery after my son was born. He’s three-and-a half, so probably three years ago that I tried to play netball again.

There's no reason I can't. According to the physio and the exercise physiologists, they both said I was fine. My physio tried to convince me to join her netball team because she was like, you just need to do it. I was like, "Oh, I want to." I loved netball. I loved the social aspect of it, but the recovery was full-on. Now, that I've got little ones as well, the thought of not being able to walk properly for that long, it's pretty scary because it did just happen out of the blue.

I wasn't doing anything wrong. I wasn't doing anything different. I didn't have previous injuries to my knees. Although they did say in the scans, it looked like there was a previous injury maybe when I was younger, but I don't recall anything bad. When I saw your study, I was like, "Wow, that's exactly me." I definitely have a fear of returning to sport. I'm only 33 now. I'm certainly not too old to play again. It's not an age factor, could have been playing.

**Interviewer:** Right at the start, you said it was devastating and it made you feel sick.

**P12:** Yes.

**Interviewer:** Can you elaborate a little bit on how you were feeling at the point of that injury.

**P12:** I think because I had done so much hard work to get my shoulder back and I felt I was the fittest I had ever been because I used to just play. I used to just play sport, netball a couple of times a week. I didn't really exercise, whereas the recovery for my shoulder involved some pretty full-on physio-led exercises. I started doing Pilates and I was doing pool hydrotherapy.

I was actually the fittest I'd ever been because I was training to play netball basically, and I did. I had an amazing season. I thought I was the best player on the court. To hurt my knee--

It was the grand final. The night that it happened, I felt sick. Just physically sick from-- it wasn't pain because I was like, "Well, it mustn't be that bad," but there was just the sense that something was really wrong.

Then when I couldn't go back out on the court, I was like, "Oh." I already had a good physio so I basically just called her the next day. That was fine, but yes, I felt like something was really, really wrong, and had no idea that it was as bad as it was. It was almost Christmas time, so I was like, “We've got a four to six-week break anyway, so that'll be fine. That's more than enough time to recover from a strain or whatever.”

Then when I found out it was another surgery and another minimum six-week recovery, that was devastating for me because I had been through that. I had already recovered from something that I thought was pretty fun. [off mic conversation]

**Interviewer:** The recovery, you mentioned that was pretty full-on. What were your thoughts and feelings around that?

**P12:** It's a hard one because I just-- I did my knee and I had my first surgery before I had kids. It was fine. I'm in an office job, so they were fine with work and everything. I know what waking up from surgery is like. I didn't wake up well from anesthetic. I went a bit crazy the first time. That was a couple of days and the anti-inflammatory medication made me really sick and I don't like taking too many of the painkillers because I just felt really sick from those.

I knew what I was in for waking up from the surgery. I didn't know what I was in for not being able to walk, but then it got explained to me and everything that, you'll be walking straight away. I had people that had had it done before, like a friend's mom. She was like,

"Oh, my mum was climbing mountains four weeks later." That didn't help mentally for me because I wasn't. I don't feel like I recovered as well as I thought I was going to.

After four weeks I was still really struggling to walk normally. The surgeon said everything was fine and my physio was saying it was fine, but I just felt like it just wasn't the same. Maybe it was because there was that extra meniscus tear in there that it never really felt like it was recovering as well as I wanted it to. I was doing a lot of physio. I did hydrotherapy again. After, I can't remember exactly, but I waited six to eight weeks to go back to do Pilates and I got back to doing floor Pilates a couple of times a week. I really struggled with running and jumping. I could do it under supervision in the gym, but when I had to go and do it out in real life, I just didn't want to. It seemed too risky, and I just don't like it. I got pregnant, so everything seemed to relax a bit as well. My knees seemed a bit looser than it should have been. Apparently, that just happens with the hormones and stuff from pregnancy.

That was another excuse not to play again. Then, I did play, because I was doing Pilates three times a week. I was doing some sessions in the gym, and I was walking 5Ks a day sometimes, before I had my baby. Then, after, I was still doing the Pilates a couple of times a week and I was doing some strength stuff with an exercise physiologist.

Everyone said I should have been right to play, so I did go back to try and play. I was just like, "No, I can't. There's something wrong, so I can't keep playing." I went and had another surgery, which wasn't a full six-week recovery, it was just a little fix and then straight back into it. I just never went and tried playing again. I lost touch with my team and just moved on. I never played again.

**Interviewer:** What would it mean to you or what has it meant to you not being able to play?

**P12:** I definitely miss the social side of it. It was my thing that I did. Everyone goes into a workplace and there's a netball player, or there's someone that asks to join the netball team. I also have also found that I'm a bit scared to chase the kids as well. My son's really quick. [chuckles] He is a runner. I've got two kids that like to run away. That pushed me to actually get my knee better again, after having the second baby because I needed to chase people. Before that, actually, I hadn't done any running again. I just avoided it. [off mic conversation] Sorry.

**Interviewer:** What part of the chasing the kids or the running was scary?

**P12:** The scary part for me was, if I was to hurt myself mid-chase, and I was there at the park by myself, that then I wouldn't be able to catch them. If I was to do my ACL again, there's no way I'd be getting up and running. My son, if I go to a park that doesn't have a gate, and you'll find there's not many that do, he would just run away. He'd think it's funny, even if I fell over and couldn't get back up.

I'd be able to get up and stand, but he's pretty quick, and I'm not anymore. That's really scary. For me. I don't like going to the park by myself with the kids, just in case something was to happen and I couldn't catch them. Even the little one, she's pretty tiny, but she would just think it's funny and run away, just to copy her brother. That's something that I just can't get past. If I was to be running like In my netball game, I was running low to pick up the ball, whereas with chasing kids, you've got to run low to try and pick them up. It's a similar motion where you're running down low, changing directions and trying to pick up a little person that's running off. My husband doesn't get it. He doesn't understand why I can't just go and chase after them, because I did do all this rehab.

I wasn't one of those people that just didn't do the rehab, I actually did it. I did it weekly, for months and probably did more than people do because I wanted to go back. We thought about it, actually. We were going to join a team at the start of the year before the COVID hit and had a few friends that were keen to create a team, and then it didn't happen. So I got an extra six, seven months of not playing.

I do want to again, but again, I just keep making excuses. I know some teams have started up again and I still haven't said I'm going to do it. It's actually been so long now that I feel like my knee is better, but definitely the fear is there. I don't think I'd want to recover from something like that again, and needing help with the kids would be hard work because I've only done it without kids. [off mic conversation]

**Interviewer:** Do you think, even now, you could improve on the scariness and those aspects I'm hearing about?

**P12:** Yes. I used to just go and join a random team. I did that, after my shoulder. My husband used to play footy and I was just like, "I'm going to find a team that's near a friend's house that I usually hang out with." She didn't want to play netball, so I just did it by myself. Just joined a team random. Whereas now, I'm like telling my husband, "We're going to join a mixed team, because I know the girls don't have to do as much and you'll be there if something goes wrong."

I don't have the confidence. Obviously, I'm a bit older now too. I used to just be like, "I'm going to join a team. I'm going to do this position, I'm good at it." Now, I'm just like, "I'm going to have to join a basic team with people that have never played before so they don't think I'm hopeless at it." Whereas, I had played for years and years. Even with my shoulder

injury, I'd played anyway. Shoulder injury is very different to not being able to run, which I now know.

**Interviewer:** How do you feel about that thought that you might think that people might think you're hopeless at netball?

**P12:** It's not great. I've always been good at sport. Sport you play at school. It was one of those things. I showed up to every sports day and swimming stuff. There's a lot of girls that don't like doing that. It's probably not as much these days, but you definitely got your two different types of people, the ones that like sport and the ones that don't. I'm the one that does. I would probably make a lot of excuses. If I was to join a team where no one knew me, I would probably say I hadn't played before, just risk them not knowing about my injuries.

That's why I think I'm going to join a team with people I do know. I'm going to make some friends that I know haven't played in a while as well play so we don't look-- I'm very competitive. I like winning. That's what most netball people are like, I'm typical of that. Which is why It was pretty devastating, because we had such a good season. We made it to the grand final. It was really good. It was a good team. I kept going and watching for a while until I realized I wasn't going to be coming back, and then I just stopped.

**Interviewer:** You said devastating a few times, what do you mean by that?

**P12:** That was my thing. That was what I did other than work. When I was at school, I played almost every sport I could get on the team for. Then when I finished school, went to uni and stuff, I just started playing social netball. That was my thing. I just did it a couple of times a week, not really a big gym-goer. I now do Pilates. I only found that after I started doing rehab.

Some people go to the movies every week and watch every single movie that's out, some people do craft or volunteering and stuff, and I played netball. That's what I used to do. Obviously, the timing of having the kids after this means I've been busy and had other things to keep me busy, but if I hadn't have had kids, I probably would have tried harder to go back because I don't really have any other hobbies or anything, that's my thing which I love.

**Interviewer:** Has your perception of yourself changed in any other regards because of this issue?

**P12:** Obviously, I was a bit younger then too, but I used to think I was invincible. My shoulder, [chuckles] It was an accident. Someone else actually dislocated my shoulder for me. I didn't fall over or anything, someone actually slammed into me and dislocated my shoulder. I always thought of that as someone else had done that to me, whereas my knee, that was me running. No one else was involved. I only gave way. It was my knee that happened, not someone else’s fault. It was definitely a different mentality. It just made me realize that I wasn't invincible, I guess.

**Interviewer:** That was about changing so far with having--

**P12:** Yes, it gave me less options. I guess I have less options of what I'm wanting to do. I used to like playing tennis as well, just socially with friends that play tennis, and I haven't done that either. Obviously, I know there's a lot of knee stuff involved with tennis, stopping and starting in the same sort of court. Whereas I loved doing that. I used to be the person that would say yes to anything. Anything sports-related, I would just say, "Yes, let's do it, give it a try." Now, I don't because it's too hard, even though my physio and the exercise guy, all said I was fine. I just don't believe them. I think they don't know as much as I know about my knee and how it feels.

**Interviewer:** What do you know about your knee, about how it feels?

**P12:** I know it doesn't feel the same as the other one anymore. I know it sometimes, gets stuck if I'm trying to do squats and things. Sometimes, if I've been sitting too long, I have to shake it out a bit. It's definitely not the same. They helped me train, but they don't know how it felt for the first few weeks after surgery, because they haven't had knees done, neither of them had. Definitely a fear of the recovery again, that's pretty full on.

**Interviewer:** What's the fear with the recovery, what did that mean to you?

**P12:** Just the six weeks. After both my kids, I had a six-week recovery as well. I just had this thing against having a six-week time limit. Everyone's like, "Oh, it's only six weeks." All the time, those six weeks went for so long for me. They was super, super long. Like I said, now that I've got two kids as well, my God, if I was trying to chase after them, I'd have to have someone here to help me. I wouldn't be able to do, especially the first few weeks. Even though they get you up and walking, it's still crutches for a week or two and it would be hard, hard work, definitely.

**Interviewer:** What would be the effect on your life? What are the things that you think about if you were to have to go through that again?

**P12:** I guess just the needing, I would need someone else to help. All the little stuff would be harder.

**Interviewer:** How does that make you feel, thinking about that stuff?

**P12:** I don't want to have to-- I say the six weeks just because I just remember being told six weeks, but I don't really remember how many weeks I actually would have needed help after the full ACL, because I did go back to work after a bit. I don't like the attention either, so

going back to work on crutches, and then when I stopped using crutches and still walking a bit funny for a few weeks. I don't like people asking, I don't like that sort of attention. I know some people do, they like that sort of thing.

**Interviewer:** Do you think the way that other people were perceiving you changed because of the injury?

**P12:** No, I don't think so. I think it was just small talk, but no, I don't think so. They knew I was a crazy netballer. It was just one of those things where I was now the knee person, the person with the knee injury. The first thing everyone asks is, "How's your knee?, How’s your knee”?

**Interviewer:** How do you feel about being the knee person?

**P12:** Look, I wouldn't wish it on anyone else, but I certainly wouldn't have preferred it to be me. [chuckles] Eight other people in the team that could have had a knee thing, but it was me.

**Interviewer:** You also mentioned it was scary, it could easily happen. It was out of the blue.

**P12:** Yes.

**Interviewer:** What are your thoughts with those ones?

**P12:** Okay, sure. It was out of the blue. Yes, it just happened. [off mic conversation] Sorry. Yes, it was definitely out of the blue. I didn't have even a slightly sore knee going into the game, it was just a normal game. Then, all of a sudden, I didn't really play again. It was definitely a shock. It was not something I was expecting.

**Interviewer:** Do you think the shock of that is contributing to the difficulty you're having with the--?

**P12:** Maybe. I know lots of people that have rolled their ankles or had small injuries and wear wrist guards, and things like that, but for me, it was my two injuries. The only netball-related injuries I've had was a full dislocation, hospital needed, and then did surgery a few years later because it kept dislocating, and then a full ACL. I never rolled my ankle or needed to wear ankle brace for a little bit. I never hurt my thumb.

I've had people on my team that have dislocated fingers, getting them caught in the nets and things like that. It was never something small that I could recover from or keep playing from. They were big, really, really big injuries. That was something I definitely used to get upset about, why couldn't I have just rolled my ankle? Why couldn't it have just been a dislocated finger in a net or something? I got a full ACL and a full dislocated shoulder.

**Interviewer:** Are you thinking about this stuff now? Are you doing anything at the moment to improve psychologically?

**P12:** Not at the moment. At the start of the year, I had. I actually moved house two years ago. I moved into Richmond two years ago. I stopped seeing my physio that I was seeing and doing Pilates with. I've tried, I've seen to two new ones since then and haven't made the same-- I was with my physio for four years in like almost weekly because of the shoulder. Then a little bit of a break and then I started doing Pilates, and then full ACL. Then Pilates and having babies and stuff.

The end of last year, I was like, "Okay, I want to go back." My baby was turning one at the start of this year, so I was like, "I do want to go back. I want to play a sport next year." I went and saw a new physio here. I told her about my knee, and I told her about my shoulder. I was like, "I haven't done much other than some Pilates in a while, so I just want to see what's going on."

I think we did six sessions where she did a running test. She filmed me running and she did the jumping tests, and everything. She said I would be fine to play, gave me some exercises to start working on and things, and I did do that. That was at the end of last year, early this year. Then, I signed up for a team that they were like, "Oh, we've got some games starting in March but this Richmond or South Yarra or wherever I was going, was going to be April. Then, of course that didn't happen. Since then, I haven't really been doing anything. I've gotten pretty lazy over the virus. I probably have to go and do some actual exercise again. Although my house has stairs, and I've been doing a bit of walking. Every now and then I do some videos and stuff. At the end of last year, I was like, "I'll need to go and see someone," and for them to tell me again, that it's okay. I did that, but then the teams just weren't starting up as early as-- If they did start it earlier, if I'd found somewhere in Feb and they'd just started up, I think I would have played. Again, I'd convinced my husband that he was playing too and another friend was going to join as well. Now, I haven't got back in contact with the club to see if they're playing again. I definitely haven't noticed any emails coming from them to say they're opening up again.

**Interviewer:** Great. With these trips of physio, did she at any point ask you what any other health practitioner asks you about your psychological aspects of the injury?

**P12:** No.

**Interviewer:** Throughout your whole start, middle, end? No one's ever kind of--?

**P12:** I think my first physio I did, but we became friends. We're friends outside of doing physio as well, so we definitely chatted about it. She used to always come back to, physically, I was fine to play. I'm not sure if that's something that she would ask other people. She ended

up making me go see the exercise physiologist because she thought I was being too lazy because we were friends.

That's why I saw two people because she thought I'd listened to someone else a bit more. No, no one really actually asked about it. She definitely pushed me. She used to tell me that I was okay to play, she definitely gave me a heap of reassurance that I was okay to play. No, the second one didn't either. This one in Richmond, who I didn't really gel with either. I don't know if she was taking me seriously enough or. Actually, maybe that's why I didn't like her as much because she said, it was like, "Yes, you're meeting all the targets. You're doing it all fine." I'd gone to see someone. I actually went to my GP to get a plan for my knee and she referred me to physio and after the six weeks it was like, "You'll be fine to play." I prefer to have someone actually guiding me through exercise rather than just going to a gym by myself. Even though I've been taught all the exercises I need to do. I know how to do them.

There's definitely a mental aspect to that because I prefer to have someone coaching me, rather than-- I haven't tried a personal trainer just because of the actual injuries. I've never actually needed to go to a personal trainer because I only really started having to do gym stuff after my injuries, seeing the exercise physiologists. Now, I don't know if it's a thing that I've developed like a dependency on the trust thing, where I can't just trust anyone. I have to trust someone that knows about the injuries I've got. That's probably an issue as well. Yes, no one's ever suggested I go see someone to talk about the fear and all that. Which is why I was interested when my husband saw your thing actually and he tagged me to say, I have to do it. He played footie, so he's had his rolled ankles. He has dislocated fingers and he's done all the small stuff and never needed surgery and recovered from them and gone back and played and not cared as much. I'm just like, "Well, you haven't had the same level of recovery as I have."

I don’t know what those are like, because I've never done a small injury I've never, just hurt myself from general wear and tear, I guess.

**Interviewer:** Great. How do you differentiate between those small injuries and those big injuries?

**P12:** For me it's surgery, like my two injuries have needed surgery. That's like a full tear that needs to be repaired or otherwise, like they said, I didn't need my ACL repaired, unless I wanted to go back to sport and I chose to. I was like, "Well, I definitely want to go back to sport." That's something I want to do. I had it repaired within three weeks, because it took us a week to realize that it was a full ACL. Then two weeks after that I was booked in and had it done. I was like, I'd worked so hard after my shoulder to come back to netball, I'm not going to let my knee stop me, and then… it did.

**Interviewer:** When you say the knee stopped you, what role did fear have throughout your initial injury and progressing into now?

**P12:** It's definitely the fear of the recovery stage. Because everyone's just like, "Oh, it's only a few weeks then only a few months and then you'll be right." Every time it's been a few weeks and a few months, that's felt like forever to me, now I can look back and go, "Oh yes it wasn't that long and my knee's fine now." That time that I spent and because I had to do-- My kids, I had caesareans as well, not by choice,

Again, like I actually, with my second child, I was told I had to have surgery so that was again devastating for me because I knew what the recovery was. Actually, that was my fifth surgery by then, and my obstetrician did not want a bar of me trying naturally because of what had happened with my first child. The second knee surgery was fine, but all others, all

four other major surgeries I've had have been major and they've been like heaps of recovery and heaps of rehab and heaps of quiet time.

When did I have my first one? I think my shoulder was in 2014, so the last six years I've had five surgeries, which is a lot. I think I'd be fine if I just rolled my ankle. [laughs] I think I'd be okay with a bit of ice and a couple of weeks off. The thought of another surgery, just like every time I have to fill out a form with, "Have you had any previous surgeries?" It's like, it's a bloody list now.

**Interviewer:** How does that feel that thought of normal surgery, being a list what does that mean to you?

**P12:** When you meet new people and like some friends that have got boyfriends and stuff and they're like, "Oh, I've done my ACL," and we share our little horror stories about having surgeries and stuff like that. I then start going, and "I've had this, and I've had that, and I've had this." It's like, most people have had one or some major thing, and then there's lots of people that haven't had anything. Yes, it's pretty full-on.

**Interviewer:** What do you mean by horror stories?

**P12:** Oh, everyone has their story of, did they go straight to the hospital after? Or being kept up all night by the machine that moves your leg? You've had four, you said, you're getting used to the machine that just rotates your leg all night. How it feels when you first stand up in the morning that was pretty full-on. You don't really just tell everyone. I don't tell everyone all the details about all my surgeries and stuff.

Then when you talk to someone that's had it as well, it's like, "Oh, what did you think of the-- What time of the day did you have your surgery? Did they let you out the next day?" That

sort of thing. It's fun to talk to if you find someone, but if you're talking to someone that's never had a surgery at all, they just have no idea what you're talking about.

The knee rotation that was a shock to me as well. I had no idea that was going to be happening. They didn't really explain that very well. It's just like they strapped my whole leg into this machine that rotated it all night, so I couldn't sleep because I had to keep it moving so it didn't go stiff.

**Interviewer:** What were your thoughts?

**P12:** I don't want to do that again, that's for sure. [laughs] Obviously, it was only one night, but standing up the next day.

**Interviewer:** Do you have any issues at the moment still thinking about it or any issues sleeping?

**P12:** No. Only because of talking about it. I haven't actually thought about-- [off mic conversation] Okay. Sorry, they're playing hide and seek.

**Interviewer:** Okay. You mentioned that your first health professional, at the time of your initial injury, did she actually ask you about any psychological considerations or did that more come in later when you brought it up?

**P12:** I think it came out when I was taking longer than she was expecting to actually go back and play. Because she kept saying, "Have you started playing yet?" She'd message me if I hadn't been in for a few weeks, saying, "Hey, have you played netball yet?" Then, she actually was like, "Do you want to join my team? Because you can play." By then, I had been avoiding it for so long that I actually was pregnant already. I was like, "I think I'm pregnant, so I can't play now."

**Interviewer:** So is it fair to say there wasn't really that communication, and then throughout the rehab, there wasn't really that question. No one specifically asked you about psychologically, maybe the end more returning to sport.

**P12:** Yes, it was definitely more of a return to sport, not a, "Why haven't you?" Just a, "When are you going to?" They definitely let me put it off. They definitely-- yes. Because it was up to me to book in to go and see them as well. The first couple of weeks, I think we did maybe four weeks of I was going in every couple of days. Then, after that, it was up to me to book in and go in. Then, I chose to do some hydrotherapy and I chose to do extra stuff. If I had just done the initial four weeks and moved on, no one would have asked me. I think it was because I kept coming back and wanting to do more.

**Interviewer:** How did you feel past that six weeks into that rehab, say the three to four-month mark? Did you have any fear? What were your thoughts and feelings into that point?

**P12:** The fear was definitely there. I always felt like my knee hadn't recovered enough to play, even though I was being told it was fine. I definitely, in the early days, had a fear of running. I was never a runner anyway, I only ran to play netball. I don't like actually going for runs, so that was not something I'd do. I was being asked to start a running program, to get back to running, and I found that really hard to do. I tried it a couple of times I never really gave it a good go. Because they were asking me to do that, I used that as an excuse not to play, because I hadn't done it. I was very focused on the technical what you should be able to do before you play netball, I really focused on that. I didn't actually achieve all of that in the early days, so I just didn't go back to playing.

**Interviewer:** In that period you mentioned, that going back period, 9 to 12 months that we're talking about now, how did you approach improving your confidence or addressing your fear at that time?

**P12:** At one stage, I was like, "You know what? I need to try. I need to go and play." I wanted to. After I had my first surgery, I think it was probably 18 months after, because I had a baby in that time. I did my six months of recovery, then I had a baby. It actually might have been two years, because then I had another six months after having a baby and I was fine to play again. I was doing Pilates a few times a week, again I was walking and doing lots of stuff. I just did, I tried it. Back with my old team, I used to watch their group. One night, they were looking for a spare player, so I was just like, "Oh, I'm able to play tonight. I'll come down."

Yes, that was a pretty horrible, horrible game. I wouldn't have been of any value. I might as well have been watching. That's when I was like, "No, something's definitely wrong. We need to fix this again." There's so many things. I played, I showed up. I was a bit shocked that I couldn't run as well as I thought I could, which could have been partly mental, but also there was something still going on in my knee, some scar tissue and stuff that had built up, and a meniscus tear.

**Interviewer:** You said that a few times, you were shocked. What does the shock mean to you?

**P12:** It's just that I definitely wasn't expecting it. I showed up thinking I'd be able to play, not at the level I was at. If you ranked people on really hardcore netball players that play every night of the week to the person that plays every week, but still doesn't understand the rules, I was always maybe towards the top. Not quite hardcore, because I was a bit lazy. I was never

a do other exercises sort of person. I always thought, "I'll go back. I definitely won't be as bad as the person that hasn't bothered learning the rules, I won't be at the level that I was. I'll be better than the person that doesn't know the rules." I was just a bit shocked that I wasn't running, I wasn't chasing the other person. I was letting them bump into me if they were that way inclined, just to avoid running and to avoid pivoting and stuff.

**Interviewer:** Throughout this whole experience, there's the shock and there's avoidance. Were you distressed at all throughout your ACL journey?

**P12:** Stressed or distressed, which one?

**Interviewer:** Distressed in any way.

**P12:** It definitely impacted my social life. My midweek social life was no longer existent. It was the friends that I caught up with on weekends and then nothing else. I used to have a definite set game, and I used to put my hand up to play other games another night as well if I could. I went from having lots to do to doing nothing, really. That's why I took up Pilates. That was one or two nights a week. The social aspect of a non-contact, non-actual sport is very different. It was a group of much older ladies, occasionally they'd be younger people, but not consistently like I did. It was genuinely a really older crowd who were lovely. There was definitely a social aspect to it, but not a team sport where you won or you lost. It was different.

I miss it. I miss the sport, I want to go back, but I need to be brave to do that. It's a risk I'm going to have to take, I guess. I'm going to have to risk it. I think I'm also stressed I haven't found a physio that I really like here and trying them out, try before you buy sort of thing. I probably will go back to the suburbs if I do another injury, which is going to be interesting if it happens. Again, I just need to suck it up, and I need to just take the risk, I think.

**Interviewer:** I think I've already asked, but what kind of things? Is there anything else that you could think of that would help you get over that emotional, mental block?

**P12:** I really don't know, to be honest. There haven't been any other contact sports that I've found that have been as easy. I tried, in the early days, when I fell in love with social netball, I tried volleyball, which would have been probably a bit easier, not as much. It's still got the team aspect, but I just didn't like it as much. I know a few people that play tennis and again, no one's going to run into and knock you over. I don't know, there was something about netball, and how many people were involved, and having a team that could easily replace you but, it was something that I just really enjoyed. I'd never really found anything else that was the same as that.

**Interviewer:** What other effects does this injury had on your overall well-being?

**P12:** I have been busy because I had kids. It's one of those things where I'm busy, so it's easier to not notice. It's easy to go a few months and go, "I should probably see if there's a netball team around." I'm constantly busy now, whereas when it first happened, I wasn't. I would go home from work, and sit around, and think that I needed to be doing something, which is why I found Pilates, but I'm not-- Obviously, it's only been a few weeks back anyway, but I haven't found somewhere to do Pilates and stuff here. I don't know if you actually played netball or if you injured yourself in other sports, but it's a different sport to-- It's probably comparable to the team stuff that actual footy teams and stuff, the bigger teams play in terms of-- Actually, my team, we used to go out for dinner before or after games, and we used to actually do-- It was a really social team. It's just something that I have always found important, especially when I was younger.

**Interviewer:** When you are faced with a fearful movement now, how do you react?

**P12:** Definitely cautiously. I think before I run. If I need to chase something, unless it's my child, and I have to chase it, if a ball or something was to roll away, I'd stop and really assess the situation before I chase it. I think I'm trying to teach my kids the same thing, don't just chase something for no reason, genuinely. I don't think they're going to do any serious injuries, but they'll see me being cautious.

**Interviewer:** How would you describe that feeling, that constant assessment or thinking about it, how would you--?

**P12:** It's definitely at the forefront of my mind. It's always there. I think I do compare it to driving a manual car versus driving an automatic car. Back before I did my knee, it was just always on automatic, and you'd get in and you'd drive. Whereas now it's a bit more manual, there's a bit more I have to think and I have to go, "Oh, if I bend down too quick, is my knee going to get stuck?" or things like that. It's is a bit more involved, which other people don't have to do. People that don't have an injury, they will just run. You see something happening and you just run. Whereas me, I'm just like, "Okay, I'm going to think first, and then I'm going to run."

**Interviewer:** You just mentioned then, that people who still have an injury, in the present tense. You mentioned that present tense, a couple of times.

**P12:** Yes. I get told I do that. My sister, I've got a twin sister actually, her husband is an ex-basketball player that did his knee, and he's also done something to his ankle that needed surgery. She constantly goes, "But Jamie's fine. He can do that. He didn't do anywhere near as much physio as you." It is. It's she compares me to him and that we're different. He's, obviously, one of the 55% that just goes back, who goes back to sport. He's older than me, he's much bigger than me, and he's got a lot more weight on him to put on the knees and the

ankles that he's injured. He's just one of the people that can and does, and obviously, doesn't care as much about the recovery and the potential for injuries.

I do get told. My sister recently went ice skating. She lives in Cairns and they have an ice skating rink set up. She's like, "When you guys come next, we'll have to go and do this with the kids." I'm like, "Woah, I don't think I can do ice skating." She was like, "Why? Your knee is fine. It's been years." I was like, "I don't know. I guess I could. I don't know. It's pretty risky though." To hurt yourself ice skating while on a holiday, it's pretty risky to me. She's like, "Jamie did it, and he's had his ankle, and his knee--" and all the rest of it. It is, it's definitely a mental barrier. I haven't had knee pain. I didn't even have much pain when it first happened. There was some pain associated with the surgery, but it's not a pain thing. It's definitely a fear.

**Interviewer:** Did that fear, in your opinion, occur more so when you found out about the injury in the initial phase or was it more the process of the surgery and rehabilitation? Where did it occur?

**P12:** I reckon towards the end. I don't really like timeframes being put on things. Now, looking back, I think I put a bit too much emphasis on timeframes, even though I know everyone's different. I got really stressed when I was pretty close to the six weeks and I wasn't walking properly yet, I wasn't as comfortable as I'd heard other people were. I think I started out pretty optimistic, that it would be six weeks, and it was a bit of, "Let's rush back into it." Then, by the time I got to the end of it, I still wanted a lot more help than I'd heard other people that had. It didn't start straight away. I had faith in the process, and I'd had seen my shoulder recover, I had done that successfully. Then my knee just never felt the same.

**Interviewer:** At the initial phase when you just first found out about the ACL, was that very fearful and distressing at that time?

**P12:** No, not fearful. The thought of surgery was stressful, definitely, and recovery was stressful. The fear of not being able to go back to sport and stuff, that didn't come until well after the six weeks. Then a few months, and I definitely was fearful to go back and didn't for a long time. I guess because I've had my shoulder done, I've done this stuff and I played such a good season going back. At the start, I was like, "Right, we've got to get this surgery done, we're going to do the six weeks, " and I did it properly. I did all the sessions, I did hydro, I was running in the water, and then it just started getting too hard.

It didn't feel normal, it didn't feel like it was supposed to. My friend that was like, "My mum was climbing mountains four weeks after her ACL." I was like, "I'm nowhere near as old as your mum and I don't feel like climbing a mountain right now." That sort of stuff doesn't help. People that say that sort of thing don't realize how bad they actually are in helping people, that probably need less encouragement than that.

**Interviewer:** The messages from other people made a big thing?

**P12:** Definitely. Because I definitely wasn't meeting the-- It was always like, "Oh, no you've done that, but I heard this person did that." I was just like, "Oh, great. It might be done in four weeks, I might be fine in four weeks." I started out optimistic, and like, "We can do this, don't want to have surgery, but we've got to." I could have just recovered and never played sport again, but that wasn't my-- I chose to, I wanted to play sport. again, I wanted to play netball again. That was really important at the time. I guess it's got less and less important as the years have gone on. I want to again, but my desire to play it isn't as strong as it ever was. It's a, "I would like to do it again." It's not a, "I have to do it again," sort of thing anymore.

**Interviewer:** Just to confirm, the messages you were getting were for the recovery at six weeks, not six months?

**P12:** Yes, I think so. Maybe I'm thinking about my shoulder. I know it was after six months that my physio started saying I should go back, but I think just the recovery to walk and be back to normal was six weeks. I think it was the same with my shoulder. It was six weeks of lots of physio, lots of stuff, and then it was a build back up to sport. I wasn't walking normally after six weeks. I was still limping a bit, I was still in a bit of pain. You're right, it was six months for sport. I didn't get to that, even though they were telling me around that time I should try.

Time: 60 minutes 33 seconds
