# Supplementary material for: Understanding Fear after an Anterior Cruciate Ligament Injury: A Qualitative Thematic Analysis Using the Common-Sense Model
Source: Int J Environ Res Public Health. 2023 Feb 7;20(4):2920. doi: 10.3390/ijerph20042920 (PMC9957354; doi:10.3390/ijerph20042920)
Supplement: Supplementary file 1 [file ijerph-20-02920-s001.zip › File S1-P13 Transcript.pdf]

P13 Transcript

Interviewer: Awesome so I'll just record these too. All right, perfect. Okay, Hi. Today you will be participating in a study that is researching fear post ACL injury. This interview will consist of a series of open-ended questions that will explore your fear, your levels of distress, and your confidence after your injury. Please feel free to say as much or as little as you want on any topic, and if you feel uncomfortable at any time, let me know and we will move on. Does that sound okay?

P13: Sounds good to me.

Interviewer: All right, so tell me about your ACL injury.

P13: Yes. I don't remember the exact year this was, but I think it was more than 10 years now, but my injury was from playing netball. I guess I hyperextended my knee on a jump or my legs on a jump and then landed with my legs straight. Then initially the physio didn't even diagnose it as ACL injury, but he was just telling me it was a strain. I actually left it for quite a few years before I actually got a proper MRI and actually got a diagnosis, an ACL tear and then I waited the one year for a private health insurance to kick in before actually having the operation. I'd say it probably wasn't until three years, at least before from the injury to actually having the surgery that I had the operation.

Interviewer: You said it was roughly, it was over 10 years ago, the actual injury. When was the surgery?

P13: I want to say when I was maybe like 22. I probably should of looked this up before I spoke to you but I reckon it was around then.

Interviewer: 2000 and--? Ish?

P13: 2010 or 2011.

Interviewer: That was the initial injury, or that was the surgery?

P13: Oh, no, the initial injury, I think, was maybe 2008. Then the surgery I'd say was like 2010, 2011.

Interviewer: Awesome. Good. Sorry, I cut you off-- Any more? How's it been going since then?

P13: Well now I just don't even notice it, but I guess the main reason for me wanting to get it fixed was because it would give way a little bit here and there and I wanted to be able to do running and all that without getting scared that it's going to pop out while I'm actually running or something, and I did want to go back to sport, but I never went back to netball. I did play soccer, indoor soccer for a period of time, but that's the closest I ever got to going back to sport.

Interviewer: Why do you think you didn't go back to netball?

P13: Well our team [chuckles] dissolved a couple of years after I got my knee surgery. I guess I wanted to give it time to feel 100% after rehab before I was going to play again. I don't know. Then I guess I just never wanted to play that sport again. I guess you could say it's probably fear out of the same thing happening again because everyone says netball's just really bad for ACLs.

Interviewer: What does the fear of that thing happening? What does that word fear mean to you?

P13: I don't know. In what context? I guess it could mean fear as in like having to go through surgery and rehab again, and there's also fear of just not being able to play to 100% as well. I guess those are the two things that come to mind.

Interviewer: The surgery, what was your experience of the surgery how did you feel about the surgery and the rehab?

P13: I think it was fine. I think I remember the post-surgery how it felt, the pain and stuff more than I remember the actual injury, how that felt at the time. I guess I had a positive experience. I went with a surgeon that was more of just a local surgeon, whereas I know my friends and my partner had gone to surgeons that were known to do footballers and like high profile sports players. People were telling me, "Oh, you should go get in to see this surgeon," but the waiting list was just so long. I just wanted to get it fixed at that stage. Everything turned out really positive. I haven't had any problems. I haven't reinjured it. He was really good with the rehab, telling me what to do and that sort of thing so I think I had a positive experience from all of it.

Interviewer: You mentioned that you were worried about not playing at the same level as before. What did you mean by that?

P13: Well, I guess you always have, in the back of your mind, like "If I do a jump like I did that time I injured it, is that going to happen again?" Then you might start to restrict the way you play a little bit because of that. I'll never know because I never played a game of netball again since, but I can imagine I would have been like that. Certainly, I was a little bit wary, playing indoor soccer because that's not the best for your knees as well. I think once I had an initial game of that, I was fine, My knee felt fine. I was like, "Okay, well there's nothing to be

worried about, you can still play." I don't know. [chuckles] I was just going to say I hope my answers make sense.

Interviewer: They're good. Thank you. At the moment you challenged your knee playing indoor soccer, and you said that that was a good experience after you played. What's your biggest fear of the netball at this point in time then?

P13: I guess probably just re-injuring it the same way I did the first time. I think the fear of any injury is greater than how I'm going to play. Obviously, who gives a shit if you have a shit game, you can always get better. I think the main thing would be, "Am I going to injure myself again?"

Interviewer: That injuring yourself again, except for those big things of going through the surgery again and not playing at the same level, that thought of reinjuring, does it mean anything else to you? What kind of other things would that mean to your life?

P13: I guess it would be a burden in that it's a long process. I don't have private health insurance, which means I'd had to live with an injury for another year. Then last time I did a bit of prehab or I can't remember what it's called. You do a bit of rehab before you actually go into surgery. I would probably try and do that as well, which means you'd have to spend more time doing those sort of exercises.

It's not a simple recovery. My partner just finished-- he's just one-year post-op last month from another ACL injury, it reminded me a lot of how long it takes to actually get to that point where you can be up and running, especially if you don't have the money to go see physios all the time or have the knowledge to rehab it yourself. Unless you've been through it again, you'd probably know what to do. Especially me, it was so long ago I'd probably need

to see a physio just to make sure I'm on track and I'm getting the movement that I would need to do. I guess it's a big burden to have an injury again.

Then also, I don't know if it's true but the cartilage in your knee probably gets worse and worse every time you do an ACL injury so I guess that's worrying in terms of, "Am I going to need a knee replacement at an early age. Is it going to cause me arthritis, pain later on?" and that sort of thing.

Interviewer: That word 'worrying' what does that word mean to you? Worrying?

P13: I don't know. I guess I don't really worry about it too much now. It would be something that would be more at the forefront of my mind if I was actually playing sport again. When I did a lot of your questionnaire thing, I felt like a lot of the questions were really like right now and also maybe a bit more applicable for people who are really fresh ACL injury. I feel like I might be a case where I may not worry too much and it doesn't really-- I don't think about it too much if that makes sense? I don't know.

Interviewer: Present time, yes. Do you think your knee function in the present time now will continue to improve?

P13: No, I don't think so. I'm 32 now, I feel like it's just going to go down from now as your cartilage and all that starts to wear out around the knee. Eventually, it's just going to-- I don't know, it feels 100% to me. I do squats at the gym, all the other stuff and it never gives me any issues.

Interviewer: What kind of effect does that have on your confidence at this point in time, that you can do all those things and everything else?

P13: Good. I can go for a run and not think about my knee at all, whereas when it was a new injury, even my partner, he's going through it now because he's been training to do a little run as a race against his friend. Here and there, he has to think about his knee a little bit more. If it's a little bit sore one day, he won't go for a run or something like that because he's still on the one-year post-op. It's probably not 100% yet.

Whereas me, I wouldn't even think about, "I've had an ACL reconstruction, maybe I should take it easy on the run this day." I'll just run freely, I'd be fine. I guess my confidence isn't affected at this point in time because it's been so long.

Interviewer: You mentioned before your fear. How would you respond if you were challenged with a fearful movement at this point in time or a fearful activity?

P13: The only thing I'd be fearful of doing is probably playing netball I reckon to be honest. If I was to play a game of it tomorrow, I would still do it now. I think if you would have asked me that the first couple of years post knee reconstruction, I probably wouldn't have done it. I probably would have said no. I think actually a couple of people did ask me to fill in a few times for a random team and I was just like, "No," because I was too scared to play. Whereas in another sport, I'd just be like, "Yes, okay, I'll play that." I guess I have a fear of that particular sport and doing that particular movement again whereas everything else, I'm okay.

Interviewer: You mentioned that at the initial phase, your first few years after you were a little bit more fearful, do you want to tell me a little bit how you were feeling? Maybe that 12-month mark of rehab to the first few years after?

P13: It's hard to say because I guess it has been so long. I guess there were times when even little things like in the cold - I don't know if you experience this as well - but your knee

especially would feel a little bit stiff and sore just when the weather was cold, when you weren't even doing much. That used to upset me because I used to think, "Is this pain going to be forever? When the cold weather hits, am I always going to feel this pain in my knee?" Eventually, that just went away. I never thought of that again. I guess in the initial stages, I'd be upset thinking, "My knee's never going to be 100%." I think that changes.

Interviewer: What were some of the factors that helped that change, that thinking pattern, and those thoughts?

P13: I don't know actually. I think actually doing things, sporty things, and playing a sport again helps a lot with that. I can't remember when I started, I would have had the operation at 22 and maybe around 26 was when I played sport again. I think that helped a lot. I did running. I did a fun run and trained for a 10K run in between that. That was really good to do. I think just challenging yourself and pushing your knee rather than just sitting around and being upset that it's sore in the cold. That eventually helped push that feeling of being upset away.

16:00

Interviewer: You mentioned a few times there, being upset about that initial injury, kind of ongoing how was your mental state regarding your knee in that immediate post-op phase and then for a little bit longer?

P13: I don't know. I think I remember feeling a little bit - I don't know what the right word is - but just feeling like there's a long road ahead and a little bit of feeling like unknown because

this is the first time I've had a major injury like that and not knowing when I'd be able to do things normally again. What was your question again?

Interviewer: The journey-- I can't remember my question. I was following on from you. Sorry.

[laughter]

Interviewer: Maybe just that initial phase of the injury. Did you experience distress immediately after the surgery or after the initial injury?

P13: Yes, both I think. I think probably more so straight after the injury because I guess after the surgery, I was more just excited that I had the surgery and was on the path to getting normal-ish again. Whereas the injury when I first did the injury, I was probably more distressed because the physios didn't diagnose it as an ACL and were just telling me it was a sprain. I could feel like it was not just a sprain. I think that this--

Interviewer: Stressed you a lot, yes. What's been your feelings when you reflect on that experience with the initial physiotherapy?

P13: I'm pretty angry about it because I could have sped up the time. I would have booked in and done the operation and that sort of thing. It did push me back a whole year which was a whole year I was just sitting around with an ACL injury. Then a whole another year, I had to wait before I actually got the surgery. It's just annoying that that happened. I wish I would have just gone for an MRI straight away or a doctor would have just said, "Go straight for an MRI," or I would have known someone who had that same situation. It was a bit annoying. I was just going to say, I think that part of the whole process was the most frustrating out of everything. Besides the actual injury.

Interviewer: What about your rehab journey? How did you feel about that?

P13: Again, it's hard to remember because it was so long ago.

Interviewer: I was going to say, do you remember if you had any fear during that time or just stress you had after that, through that rehab?

P13: I would say the first three months were probably the worst because you make little progress over that first three-- Oh, well, not really because I was off crutches after a week. I'm trying to remember. I would probably say the first three months were more-- you have more fear about, "How's this going to go? How much time is it going to take for me to get there? I haven't got full range of motion yet. Should I be at this stage yet? Should I be more advanced?" and that sort of thing.

At the same time, I was seeing physios regularly anyway so they would always give me new exercises and assure me that everything's on track. I don't think it was that bad. Sorry, I guess in a way I kind of block it out, some of that, from my mind because it was so long ago and it wasn't a fun time learning to walk properly again or weight bear on a knee. As I'm sure you'll know.

Interviewer: With reflection, do you think you had a bit of trauma associated? Emotional or mental trauma when you're looking back now and trying to think about it?

P13: Yes, I reckon because it's a big thing to fix a knee and learn how to use it again and have function in it and range of motion in it like it had before. Yes.

Interviewer: During all this, do you remember if any health professionals were asking you about any psychological considerations of the surgery or the journey?

P13: I'm pretty sure never but actually I was talking to Archy about this the other day and he said that his surgeon actually asked him about the psychological aspects of post-injury or something. I was like, "Wow, I don't think my surgeon or even my physio ever asked me anything to do with the psychological aspect." That was 10 years ago, maybe they do that now, I don't know.

Interviewer: What kind of effect do you think that could have had on your knee journey if someone had have asked those questions?

P13: Yes, I guess it always helps if people are checking in on your well-being as opposed to just your physical well-being. Whenever you can squeeze anything like that in, I think it's always a positive so-- It's hard to know if that would have helped, me having less fear in going back to sport and that sort of thing. I guess I would have liked some guidance on that aspect, returning to sport, and how do you overcome the fear of reinjuring yourself and that sort of thing.

If I'd probably would have had support like that, maybe I would have played netball again, I don't know. In a way, it was just up to myself to make a decision on do I want to do that and do I want to play sport, thinking about whether or not the injury was a part of playing sport.

Interviewer: You just mentioned your effect on your well-being. What effect has this injury had on your overall well-being as you look throughout your whole journey?

P13: I don't know. Now I don't really think that it's-- Probably back then, I probably would have thought it's affected me a lot because it's taking time out of your normal day-to-day focusing on something else, whereas you could have been playing a sport you could have been out with friends, you could have gone out to that club back then. It took away from

doing all those other things that people with injuries don't have. I guess, in a way, it affected my well-being back then, but versus now, I guess I don't really think about that too much.

Interviewer: I know you don't think about it, but do you think your perception of yourself has changed as a result of the ACL injury?

P13: I don't know. I guess I'm pretty proud of the way I came back from it and spent the time doing the rehab and got my knee to that point where I could do that 10K run. I can play sport and be good at it again. I guess I'm proud of myself in that regard. I don't know. I guess it's bad to go through an injury but I guess it's good to come out the other side and know that-- I was going to say, "You'd fixed yourself," but that's probably not a good quote. You got yourself back to where you needed to be.

Interviewer: Again, reflection on it, how has that made you feel thinking about that success, I guess?

P13: I guess happy. I don't know. I'm not very good at expressing my emotions. [laughs] Sorry.

Interviewer: You mentioned yourself but what about, has the way that other people perceived you changed because of the ACL injury?

P13: I don't know. I guess so because I guess I look at my partner and think he's done a really awesome job at rehabbing all of his injuries but I don't know. I'll have to ask my friends or my family.

Interviewer: How was their role during the whole experience?

P13: Yes, I guess very supportive. There's a couple of snippets of things that I remember during that time of really nice things that people did for me to help me especially post-op and during that first injury phase. I think you've spoken to Tash, I know, I guess she was a big driver in pushing me to go get MRIs and get peace of mind on what's happening with your knee and stuff.

Interviewer: How has that been-- again I know it's a long time ago, but reflecting on that whole experience and talking to other people, what kind of emotions or feelings have you uncovered from those interactions?

P13: Which ones? Like the--

Interviewer: The talking to other people that have experienced the injuries like Tash and chatting through some of those aspects?

P13: I guess it's good to know that you're not the only one that it's happened to. It's nice to relate to someone about an injury and talk about things that you might be feeling with it not feeling 100% or little things happening here and there.

I guess it's really good to bounce that off another person and have another person to share their experiences with. I think it was really helpful at the time because I think Tash was the only other person I knew that had had an ACL injury. She was really positive to have around. Also to talk about the rehab side of things as well because she's really into all that stuff as well.

Time: 30 minutes 17 seconds

P13
