# Supplementary material for: Understanding Fear after an Anterior Cruciate Ligament Injury: A Qualitative Thematic Analysis Using the Common-Sense Model
Source: Int J Environ Res Public Health. 2023 Feb 7;20(4):2920. doi: 10.3390/ijerph20042920 (PMC9957354; doi:10.3390/ijerph20042920)
Supplement: Supplementary file 1 [file ijerph-20-02920-s001.zip › File S1-P14 Transcript.pdf]

P14 Transcript

Interviewer: All right. Hi, how are you?

P14: Good.

Interviewer: Today you'll be participating in a study that is researching fear post ACL injury, the interviews will consist of a series of open-ended questions that will explore your fear, levels of distress and confidence after your injury, please feel free to say as much or as little as you want on any topic and if you feel uncomfortable at any time let me know and we'll move on.

P14: Yeah.

Interviewer: All right, so tell me about your ACL injury.

P14: Okay. Well I've had three.

One was my first one was when I was 16. The second one was 19. Third one was really five years ago. They were all from soccer.

So do you want the details of what happened in them all. Or just give you a quick overview of all of them?

Interviewer: Were they contact related or non-contact related. How do they all kind of occur?

P14: So it was contact related. So I was sandwiched between two… I was dribbling the ball and sandwiched between two girls and you know fell and then did my ACL and LCL and the second one I was slide tackled from behind and that was just the ACL and then the last one was indoor my knee actually just gave way and I fell and that was my MCL and a ACL.

Interviewer: So you had two kind of contacts that you almost weren't in control of and then one cut, was it a cut and pivot twist? or what was the mechanism for the third?

P14: So yeah, it was a cut so I was trying to cut in when I had the ball and then I was trying to yeah change directions and it just yeah gave way.

Interviewer: Okay. And what was the process with all those three injuries? What kind of management did you undergo?

P14: So, what did I do?

All of them? Yes as soon as I got home. I didn't walk or anything because it just blew up straight away and I was in a lot of pain but then it went away, the pain, and I was just icing it and everything when I got home and elevating it then I went to the physio for all of them and

then got a referral for an MRI and then pretty much had surgery. So it was a little bit different for all of them because with the meniscus, my surgeon wanted to operate on that straight away. So from memory, I can't really remember the first one but from memory, I think we operated not long after because I had Private health insurance as well. The second one, I think I just rehabbed it for a while and didn't play sport or anything like that for about a year because I was doing year 12. I was like studying or something. I can't remember and then so I just left it.

Oh, no, no, no. Sorry. I didn't return after my first one. I did return to sport after a year and then because of year 12 and after that I went back to school and then this second one I can't actually remember too much about the second one. I must've just oh, yeah, I got it done. Eventually. I can't really remember how long after anything like that. I got the surgery done and then I wasn't going to return to sport again and then my friend was like I'm starting an indoor team do you want to come play just for fun. So that's when I did that and then that was probably my worst one and then that one he pretty much operated, just did the meniscus so I was in a Zimmer splint for like six weeks or something rehabed my knee and then went for more surgery again and then did the ACL.

Interviewer: So to clarify, first one surgery then one year off, then went back to sport, then you reinjured before the second one was the year 12 when you did it again?.

P14: So start again the first time I was 16, and then I did the surgery pretty much straight away and then I took a year off because then I was doing year 12 or whatever because I think it might of happened towards the end of the year or something like that. So I took a year off because I was doing year 12 and then a couple years later or a year or two later I started

playing soccer again. I think I only played one season and then I did my ACL again and then I didn't return to sport for quite a few years. I can give you dates after, I just can't remember right now. If that makes it easier the last one was indoor. And yeah, I had two separate surgeries for that one. And so the first surgery was the meniscus repair because he wanted to do that straight away. And then the other one was, the second one was the ACL after.

Interviewer: Injured at 16, one year of sport, so plus or minus a little bit longer you had surgery at that time, then 18-19 the second one. Did you have surgery for that? You said that all had surgeries as well?

P14: Yes. Yeah, absolutely for all. Yeah,

Interviewer: And then you went back you said several years after that.

P14: Yes at 24-25 maybe.

Interviewer: You went back because your friend was like come play indoor soccer and it would just be, that amount of time had passed.

P14: Yeah, I just thought my knee felt really good. I was going to the gym, you know, everything was really good and I just was pretty confident to go back and I thought just

because it was for fun. Oh my God I might be that competitive, but I am, and then I just got probably too involved in it and then yeah.

Interviewer: So did you do full rehab things for each one. You went to the Physio after.

P14: Yeah after the surgery. I went to the physio. I went to the gym. I probably could have, I was probably focusing more on strength than like proprioception and agility, so I probably didn't really rehab it properly and do sports-specific. Although I went to training and everything like that. I probably should have in hindsight should have done more of that. But yeah, I went to the physio. Yeah and I was going to the gym as well

Interviewer: And you stayed with the physio all the way through for kind of that end stage, nine to 12 months of the classic program?

P14: Testing my memory. Um

Not for the whole nine months. No.

I think also because I was studying, you know exercise physiology as well. I was kind of prescribing myself, you know, I'll be going to the gym and doing my own thing as well. So no, I didn't stay for the whole time. I don't even remember how long I was there for to be honest.

C:And how old are you now If you mind me asking so the last time was about 23 that was how long now?

T I'm 31.

Interviewer: so about 8 years ago?

P14: Sorry Ill need to I need to confirm dates with you because I actually cant remember, I should, I'm sorry. I should have probably looked that up before but I will send through the details and I'll confirm the years of when they're all done.

Interviewer: So in regards to that injury, you mentioned before or previously that you've had fear as part of that that injury. How would you describe this fear.

P14: So for after my first one, I felt really good and went back, after my second one It took a little while, but my knee felt really fine like no pain nothing. So that's why I went back again to play indoor because I honestly felt fine and then the last one now is actually like really bad so I still get like a sharp pain through my knee. I think that's got to do with the cartilage or something maybe so that sometimes when I turn certain way or even when I'm walking I'll just get a sharp pain through my knee every now and then and so that has really, you know messed up my confidence with what I can and can't do now so, you know. I'm a quite an

active person's I work out and go to the gym and things but you know, I'd love to do other things and I just don't have the confidence in my knee to do that anymore

Interviewer: So you're biggest problem at the moment is that confidence and that pain

P14: I think that pain is what you know rocks my confidence, I guess because I don't know when that's going to come on. I can't really identify what movements bring that on. It just happens. Sometimes it just goes away straight away. It's just a sharp pain and it goes away. But yeah, actually sometimes it does flare up like some days it actually quite hurts a bit. I can walk, but it feels tight or a bit stiff and there's like a little bit of a dull ache through there some days and then some days Iike today, it's absolutely fine.

Interviewer: You haven't identified any link or any anything between those days and those pain related flare ups?

T:Not with the sharp pain, I haven't identified anything that brings that on. it just comes on sometimes, sometimes I will be just walking and it will happen. Yeah, when I kind of change directions or something it will happen. With the puffiness sometimes in those days when it's a bit more sore and stiff it is a bit more puffy. I can't really identify what it is. I don't know if it's because of exercise Maybe, I'm not sure because I tend to work out. I have been a bit slack lately, but like three to four times a week or so, so I don't know if some days I've just worked a bit too hard. I'm not too sure of doing something that you shouldn't do.

Interviewer: And you attribute that to the meniscus you said before?

P14: Yeah, I'm not too sure if it's the meniscus that's causing that, I don't really know, the way that the surgeon explained it. Was that which I think I may have explained it to you the other day, but he said that there was a chip in the tibial like, you know the end of the bone kind of bit and it's so like the chip is here and it's where the weight bearing bit is and so he said that if that didn't fil out over time, he would have had to have drilled into, Sorry the femur, and then the bone marrow would have seeped out or something like that and it would have filled out the hole but he didn't have to do that or something. So I don't know if that's what's contributing to that sharp pain or not or if it's the meniscus. I'm not a hundred percent sure, but I think there was some bone damage there which means.

Interviewer: So at the moment the biggest issue is more about that pain and that bone damage

How do you feel about that that message from the surgeon looking back at it now. That sounds like it was quite a severe message.

P14: Yeah, he did say was well, you know he's quite a good surgeon. He's the surgeon for the Western bulldogs. And he said that it was ‘one of the worst injuries he had seen that year’ and he said, ‘you must have been in a world of pain at the time’ and I was praying that I didn't have to have the drill into my bone and do all that other stuff. But he said that it, you know, with all the scans and stuff. He said it filled out enough. So it was okay. I just didn't realize that I'd still get that pain.

Interviewer: So by drilling into the bone you mean he was going to take some marrow from somewhere else or do you mean he was going to let it bleed and harden up.

P14: Yeah, that one, sorry the second one, to let it harden up so it filled out the gaps. Yeah. but I still get a bit of pain generally as well, which I don't think that's just that bone part. I think that's just all the trauma I guess I've done to the knee, like I still get sometimes, I remember one time in London, which was what two years ago or something now, I just woke up one morning and I was struggling to walk because I had to walk with a limp. Absolutely no explanation for it was just so sore and that hasn't happened again since but that's just…., sometimes I do get a bit of pain, not to that extent. But yeah where I'm just like actually it's quite sore today, you know bit stiff and you know.

Interviewer: So how do you feel about all that, your current state of your knee? How do you feel about the fact that it can get aggravated and painful.

P14: Yeah, it makes me anxious and sad as well I guess because you know I'm quite an active person so I would have liked to have taken up more you know activities, you know dance or you know something different, but I just feel like I can't because of my knee and it makes me anxious as well, like in the future just for example, to say if I get pregnant or something like that I'm going to have a lot more weight on me. You know, I'm going to put on like what 15-20 kilos or something like that just makes me anxious if my knee is going to hold up during that time as well. So all of that makes me, some days when I think about it a

lot. Like it makes me quite anxious I guess, but in terms of sports and everything, yeah, I just know I can't even really play for fun, you know at the park with friends or you know, just yeah. I know it's really affected my life a bit and I feel like soccer was a passion of mine. So yeah, it's sad that I can't even just play it for fun or anything like that. If I really think about it I get quite down, but I try not to think about it too much now, but yeah,

Interviewer: So you've lost this really important aspect of who you are and what you were doing with yourself. Was that something you used to really define some of your personality or some of who you perceived yourself as being.

P14: Yeah. Yeah, definitely. Yeah. Definitely.

Interviewer: You said that you are anxious about it. How do you describe that anxiety? What are the symptoms or how would you how would you operationalize for want of a better word that anxiety?

P14: Well, I get more anxious about. Well, I do probably avoid doing things like how I said before I'd love to take up dance. But I'm just quite anxious of how my knee will hold up. I just think it might I just don't want to be put back in that situation again where it gives way and I have to have another one or even the pain.

Interviewer: You also said the word worry, that you worried about it in that regard you would not want to go back to dancing because you would be anxious or worried.

P14: Yeah that I would do it again because I haven't done dance before but it's just something that I have an interest in, just because there's lots of different movements and you know a lot of twisting and you know spinning and all that kind of stuff. It makes me, I guess yeah just by doing all those movements that it might give way again or might cause some pain. I just really don't want to go through that again. And you know, not only just me, you know it created a lot of stress around my family as well. So there's a lot of components I guess to think about,

Interviewer: So when you think about possibly going back you think about all those things, you get, is apprehensive maybe a good word for it when you are looking at doing this thing

P14: Yeah, I would never return to soccer now that just yeah makes me really apprehensive. I just can't even fathom the thought of but not even playing around, you know, maybe juggling the ball or something like that. I feel like I could do but not when you're running and change of directions. Running is fine actually, but just like the change of directions and agility component of it and there's a lot of that in there. You have to be quick and everything like that. So I wouldn't even think about even playing against anyone really.

Interviewer: You mentioned a lot of things that you're kind of really worried about in there. But is this something you wouldn't consider doing again because of the fear of the pain or the fear of the possible re-injury.

P14: Probably the re-injury side.

Interviewer: So when is the lot of experience kind of pain you mentioned your knee gets swollen after you run? and that time in London in 2016 or so. Has it been recurrent episodes of pain or has is it been a little while now?

21:00

P14: Besides that sharp pain which happens if not daily, but then weekly that sharp pain the other the other so that happened that sharp pain happens, like it could happen once every few days or you know, maybe once a week it varies could even happen a couple times a day. You know, but it pretty much goes away straight away that then that general stiffness in the achiness that doesn't happen very often the last time that probably happened, it does happen sometimes actually probably once every couple weeks or every three weeks, but it's not anything that stops me from, you know doing my normal daily life. It's just something that I'm aware of in that, you know when I compare it to my other knee I think if I hadn't, you know returned back to sport all those times. My knee will probably feel the same as my left knee because it was all on my right knee so I think about that sometimes when it's really achy and a bit swollen and stiff

Interviewer: Do you think about what could happen if you were to get injured as well.

T:Well, yeah, even if I didn't return the third time, you know, how my knee probably still won't feel the same, my right knee probably won't feel the same as my left knee which is my good knee. But, or the non-injured knee, Yeah, you know probably, I just think that if I didn't return back to soccer or if I didn't try it again, then, you know my knee would probably feel a lot better.

Interviewer: Yeah, so I didn’t clarify that at the start, all 3 knee injuries are on the right?

Yeah, All right, and you mentioned also stress in your families, you said something like I couldn't do that to the family again. What was the effect it was having on your family?

P14: Oh, I'm quite family orientated. So I'm really close with you know, my immediate family and my step family. But yeah, It just put a lot of stress on them. You know for one of the Recos when I did my medial meniscus. So the most recent, the latest one. I was in a Zimmer splint for like six weeks.

My sister has a traumatic brain injury from a car accident from years ago. She actually has like some spasticity in her foot. So she was going through surgery at the same time. And so we'll both kind of in splints, you know at the same time and my parents were running back and forth between hospitals to manage both of us and to look after us and all that kind of stuff

and it just puts a lot of stress, because obviously they care about me and my well-being, so just put a lot of stress on them as well.

Interviewer: Yeah sounds very stressful and traumatic as well.

P14: Yeah, if it wasn't good timing as my sister's operation was planned for ages. But, you know my knee me, I didn’t know that was going to happen, It just kind of happened. But yes it was pretty busy

Interviewer: In regards to all these kind of things you've mentioned a lot of current worries, anxieties and apprehensions. Are you doing anything at the moment to address any of these worries or thoughts? Or do you think there is anything you could do?

P14: I do well, I exercise and I do try to you know do lower limb exercises. I tend to focus more on my right side. So I tried to. I'll probably be, you know, even just for, I don't know how to explain it. I do try and incorporate exercises where I am strengthening more the right side. I probably should do more. Like I said before proprioception type exercises, which I haven't really done as much of, so yes and no I guess so some things I feel like I have done. I feel like the bikes really good for my knee. Just because there's none of that weight bearing or the impacts that running has so I tend to do that more than you know go on the treadmill or run and I'll try to do you know some just general, you know lower limb strengthening exercises and things and that I probably should focus and do more of them. Yeah, like balancing and proprioception type stuff I think, which I don't really do as much,

Interviewer: So just to clarify you think if you got some more of those more mechanical training aspects a little bit better, all those anxiety, worry would reduce.

P14: Nah, actually, I don't think so and that's probably why I haven't really done it. I think because of that sharp pain that comes through my knee. IDon't think any exercise is really going to help that. I could be wrong, but that's just what I think so. That's probably why I haven't really incorporated as much because I don't feel like it will make that much of a difference.

Interviewer: So can your knee function be improved at all at this point.

P14: Well, I haven't tried so I don't know. I don't actually know.

I feel some things would probably improve, it would probably improve a little bit.

In terms of my confidence and anxiety though. Probably not. Yeah.

Interviewer: So you haven't tried, what do you mean you haven't tried to improve, what things could you possibly try to improve the confidence and reduce apprehension and things like that.

P14: Well, probably if I were to try things I would probably do like I said proprioception exercises and probably some agility drills, but obviously starting quite easy and then see how.

Interviewer: So if you were to push yourself back into agility and proprioception and mimic some of those end stage rehab things you think that'll maybe improve the confidence and the apprehension and the worry.

P14: Well now I'm thinking about it. Probably. Yeah, probably I just haven't done it.

Interviewer: You said you don't really think it could be improved much at this point.

P14: I don't think that sharp pain that I get is going to improve at all. But yeah, it might improve my confidence in my knee because it might make me realize that I can do more or I can trust my knee a bit more than what I do. But yeah, I don't think it will help that sharp pain which I feel is one of the main limiting factors for the confidence in my knee, if that makes sense.

Interviewer: Yeah. Okay. So let's move back to maybe the start we will go to when you first got injured your initial injury. Prior to that initial injury. Did you have any fear?

P14: No, no,

Interviewer: Yeah, and then after that initial injury. Do you think that after the initial injury you had any fear after that. Or was there some fear of reinjury

P14: There was a bit of fear, but I pretty much was the same. I probably wasn't, as you know confident or you know, I guess before the first one I would just kind of throw my body all over the place and like slide tackle everywhere and you know do whatever and I still did that after my first injury but there’s probably a little bit of reluctance but honestly I was pretty confident still so I don't feel like I got yeah, I don't feel like it affected it that much

Interviewer: And then over the next couple of injuries when you first doing them. What about that second injury after you first year? Were you a bit more fearful?

P14: Yes. Yeah bit more. I just I didn't have any intention of returning, my knee actually felt really good as well. After that one. I didn't feel like I had any issues. I was working out and everything like that, but I I probably didn't go back more for my family because they were didn't want me to go back and they were just like no that's enough, but then after a few years I was like I can play indoor and then it happened again.

Interviewer: Your family actually asked you to stop did they.

P14: Yeah. My surgeon as well said ‘no more, you should take up golf’.

Interviewer: Your surgeon told you to take up golf?

P14: He said it after my last one. He said to take up golf. He was really angry about my last one because it ruined all of his beautiful work.

Interviewer: How did you feel about him being angry.

P14: He wasn't really angry, but it was just a bit like oh my gosh, because he could still see the ACL it was kind of intact it just kind of pulled off I think so he could see you know, he just said it had healed really nicely and everything was really good. But yeah, I felt bad but you know, what could I have done I injured it again and I can't really do anything about it.

Interviewer: So your family asked you to stop. Were you just stressed or worried or anxious more so about returning than just the family asking you to layoff soccer. Yeah, so it's just hard to remember because it was a while ago. But um, yeah, I would have had some you know anxiety at the time as well returning. Otherwise if I really wanted to I would have done it. But yeah, it's just because I was a bit older as well. And the first time that I'd done it I was playing state. So it was just a bit higher level kind of thing. So I could have potentially, I was supposed to go to England to play and all that kind of thing so I could have potentially done more. So when I was younger there was more motivation for me to go back but then as I got older that boat had kind of sailed so I couldn't really do anything besides play for fun. So I guess it wasn't as important after my second one, but yeah, it did make me sad, you know not being able to, well not going back you know, even watching games like watching the EPL or you know watching Friends Play I couldn't really go because it just made me really down, because I couldn't do that. Yeah, but then I I actually thought my knee was okay. Like I was

pretty confident in it. That's why I went back and started indoor and I thought it was a bit different but you know, it's not it's probably worse.

Interviewer: That was quite a few years later though, you said 18-23? So when you were considering during that middle period when you were considering going back you were getting sad. What was stopping you then when the knee felt? All right, pretty good.

P14: It was just my lifestyle as well because I went to uni at Ballarat as well. So I would be there during the week and then I'll come home for weekends. I wouldn't have been able to train or anything like that anyway. I guess also my family like I said before and it just wasn't as much of a priority after that.

Interviewer: Yeah. Yeah would have yeah.

35:41

P14: It made me sad not playing and I missed it but I just thought it was probably best for my knee not to play. You know just to not reinjure it again.

Interviewer: So you thought it was best just not to re injure it, what would happen when you imagine that kind of re-injury what's the series of events that you would imagine a reinjury would set back into motion.

P14: Sorry, can you repeat that?

Interviewer: What was the effect of re-injury, what, if you had of re-injured in your head when you're weighing it up going? It's not as important anymore and you know, I would potentially like to do it but I've got other commitments it might be not worth it. Like what was it about the re-injury that wouldn't have been worth it. What are you what are you trying to get across by that?

P14: Fear of the re-injury did that stop me? Yeah, it stopped me a bit, probably initially like after I had rehabbed it and everything like that. Definitely, there was that anxiety returning and the fear of re-injury and then after a few, obviously because it was a few years later. I felt pretty confident in my knee after a few years, but definitely initially after I had rehabbed it and everything had gone, you know, and then I just kind of got my normal life back. Yeah, there was that. Yeah the fear of re-injuring and just the whole rehab process as well. You know, it's just such a long process.

Interviewer: Yeah. So the rehab process when you consider that fear re-injury you talked about, the rehab process is the big thing that pops into your mind that you're like,

P14: To be honest the um the pain like yeah, like really hurt but I feel like I could deal with that a bit better. I wouldn't want to do it again. Like go through the whole pain again, but I could deal with the pain but the rehab process especially during my last one. Yeah, I just you

know, I just got really down. I got quite depressed actually especially during the last one I think just because when it was just the ACL like when it was just the ACL after my second one. I just had just injured that ACL. You know, they encourage you to start walking pretty quickly and you know, you're on your feet pretty quickly and you know, all that kind of progresses not bad and you kind of get your Independence back pretty quickly. But then the last one when I was in the zimmer splint for six weeks. I struggled heaps. Yeah I got quite depressed during that time just because I couldn't really do anything.

Interviewer: So you mentioned before that your parents had to help you and that made you feel guilty, or was it the fact you were just unable to do normal activities that made you feel so bad.

P14: Yeah both. The fact that I had to rely on my parents so much and then they had to do so much for me if we referring back to my last one with the zimmer splint I had a boyfriend at the time. He did heaps for me as well, which was really good, another boyfriend at the time, and then yeah, it's just the fact that I couldn't drive like my mom had to drive me to work. So luckily my work found me other duties that I could just do it the desk because I was working as an exercise physiologist at the time doing clinical work. Luckily they found me some desk duties that I could do and Mum had to drive me like 45 minutes to work or 40 minutes to work and then, you know, drive me home and then had to go to my best friend's wedding in Queensland and I couldn't really do anything. I just had to sit down. Well I was in Queensland and I couldn't even sightsee or anything like that. I was really I was really depressed at the time.

Interviewer: Yeah, so that last knee injury specifically, you mentioned how kind of distressing it's been several times. Do you think now reflecting on it that all those other things occurring at the same time have contributed to this overall view of it is kind of this really bad distressing thing.

P14: Yeah. Yeah, probably. Yeah, all together definitely.

Interviewer: Yeah, missing out on your friends. Not being able to participate as much as you want in your friend's wedding.

P14: Yeah, and my boyfriend at the time had to push me in a wheelchair because I could literally just walk 10-20 meters and then I'd be exhausted because I was on, you know, crutches and everything as well. Even on the plane, you know, yes extremely uncomfortable.

They were really accommodating, on the way they were able to get me three seats so I could put my leg up but on the way back the plane was too full so yeah everything and I literally just had to stay in bed until I could start actually getting up but when I first had the surgery and I was first in the Zimmer splint I was just in so much pain and literally just in bed and it's just like I said because I'm quite an active person and I like to do things. I got really depressed during that time and then as soon as I could start and because I couldn't actually couldn't do anything in either like he gave me really minimal exercises. Like it was just leg raises and static quad kind of exercises, you know, getting the VMO. It was just really simple exercises. And so there's only three of them so I did them as much as I could but there's only so much, you know, you can do, as opposed to you know when I could start taking it out of

the Zimmer and then start doing the range of motion and all that kind of stuff I felt like I was contributing more to my rehab whereas at the start when you actually advised not to do anything makes it even more difficult because you can't do anything.

43:13

Interviewer: So You've mentioned distress and also anxiety you mentioned earlier. How would you describe all these different words. How do you describe distress? What is distress to you?

P14: Um, I think it's like constant, I don't know, like it's just constant tightness I guess in your chest where you constantly feel like there's something wrong. Yeah, I guess also being you know fearful of re-injuring or fearful of pain. I feel like that's a bit of distress. I guess during that time, the you know, the lack of Independence as well, not being able to do things for myself and then relying on other people, that stresses me out because I don't really want to rely on other people, I'd like to be able to do things myself. Not just rely on other people, like rely on people to take me places or to do things for me or to help me shower all that kind of stuff. So yeah. Well that was quite distressing

Interviewer: so that helplessness again you've brought that up a few times, that aspect of relying on other people that seems to be the driver of why this incident, especially that last incident was so negative really.

T:Yeah definitely. Yeah,

Interviewer: We are jumping around a little but maybe we can go into a chat about what your process was so during your rehabilitation you mentioned that you are you were chatting and seeing physiotherapist throughout were they chatting to you anytime about your anxiety or distress or any psychological contributors to the rehabilitation?

P14: I'm just trying to think of the last, because I can't actually remember the physio for the last one. I actually think my surgeon didn't recommend one. I'm just trying to remember.

Interviewer: You mentioned that your surgeon told you to do quad squeezes and take it pretty easy.

P14: Yeah, it was yeah, and then he's the one who kind of directed me to just go on the bike. I would have seen someone. I dont think it was much though because even when I had the reviews it wasn't for long because when I'd have the reviews with my surgeon he would constantly give me more exercises and you know based on how I was going he was a bit like you're going okay. I'm just trying to remember because I don't remember if physio was a really significant part of my last one. I'm just trying to remember now. No, I think I went to Berrick physio. Yeah. It just wasn't for long. I don't think, no we didn't really go through mental health or anything like that. We didn't really talk about that, especially with the shorter sessions because the sessions usually lasted for like half an hour or so. It was just more.

Interviewer: The physical stud maybe a bit more.

P14: So maybe then if that was the last one that was obviously very traumatic and at that point would you say you were disengaged a little bit because you weren't wanting to go to that higher level because as you mentioned you maybe werent looking to return a sport at that time, especially after that last one. Yeah. Well, yeah the first one I yeah, I remember was sort of easier and my dad was researching for really good ones and because I was trying to get back into soccer and you know, all that kind of stuff so we went to quite we tried to you know, see quite good physios. The last one was probably more the direction of my surgeon as well because he was a bit like, he was giving me exercises and I guess I've quite trusted him as well because he, although it's not really his area of expertise, but yeah, just because he's got such, like he's pretty highly regarded I guess in the industry.

Interviewer: So just really quick back to that first physio that you said you kind of did go all the way through with and were trying to get to that end stage how were those session structured to improve your confidence? If he didn't necessarily talk about, the confidence and psychological aspects of rehab, what kind of stuff was he doing to build your confidence and reduce your anxiety, fear and distress as well as other things we talked about.

P14: Well, I don't think we went through the whole, you know, nine months. Although yeah, I did see them for a little while. But I think it was the whole nine months. To be honest. I

don't even remember them talking to me about psychological, like psychologically how I'm going.

Interviewer: So you maybe you were more completing the exercise progressions and things like that.

P14: Yeah. Yeah and I actually don't even recall any physios really talking to me about, or my surgeon or anything like that about maybe just in general, like ‘how you going?’ How's it feeling, didn't really go in depth about…

Interviewer: So how did you know you were ready to return to sport after that first time. At that 12 months you said you roughly went back at? I think you had the delay and then you went back so I’m guessing a year and a half or so from 16.

P14: Yeah about a year and a half. Yeah fine. I felt really good and that was more probably because I wasn't in any pain. You know, I was exercising I did pre-training pre-season training and I didn't have any issues so that was fine but I didn't.

Interviewer: So you're kind of end of return to sport outcomes in a way were just literally feeling this is all right, and you just went back into training.

P14: Yeah, pretty much

Interviewer: And then obviously the second time around, because of all those other things going on. You didn't have that that push to get back at that time. And then that went out obviously there was no kind return to sport measure anything was there or was it the same when you return the next time at the five years?

Just so in this the current moment and I've touched on this but if you were faced with a fearful movement, how would you respond?

P14: Now, yeah, do you mean if I did it or?

Interviewer: If someone was like Hey, can you run cut pivot twist move?

T:No way

Interviewer: it's a bit of a bit of a hard question. But in terms of the whole experience with your knee injuries, you know mention that it did get you down multiple times. What is the overall effect you think these knee injuries have had on your overall well-being?

P14: Well, I wouldn't know where to describe it. Oh, gosh, I don't really know.

I guess that has been quite traumatizing if I reflect back on it reflect back on it now. Yeah, just because now I'm constantly…I was talking to Sam, like my partner about it the other day, and there's just things that he does and he doesn't even think about it and then I would say

‘oh, I can't do that because my knee’, like I'm a bit worried about my knee and he just couldn't really understand that that's constantly on my mind. So that's probably how it's affected my life. You know, it's something that I'm always aware of and thinking of, not always thinking of, but aware of so if someone were to ask me to do something or just even for example skydiving or something like that. I'm just like no because I am worried about the landing or you know just doing like other activities or you know, I don't know anything really, um, yeah, I just yeah, it makes me question or doubt and question even tasks or movements and things like that.

Interviewer: You worry about it. You question it. How would you describe? the worry if you were going to try to describe what that worry means to you. How would you put that two words?

P14: Well that's a hard question to describe the worry. It just be like the just re-injuring it, the worry of re-injuring or causing pain or you know affecting my knee again will I have to you know go through the whole surgery or rehab process or anything like that or I just don't want to be put in that situation again, so I just prefer to avoid situations or even I guess know it is a bit of a pain component to I don't really want to aggravate it where I can't walk or it puffs up or I'm in pain for a little while. Yeah, its affected my life heaps.

Interviewer: Yeah, and even to the point where you were saying, you're not sure about getting pregnant because of the weight bearing.

P14: Yeah, like I will but it stresses me out because I just yeah I'm just thinking about the weight-bearing kind of thing because I know you put on weight and I just don't know how my knees are going to cope in that situation plus all the hormones. I don't really know, but just with that, you know I could actually imagine myself having to use like a crutch or something like that because the pain in my knee might get really bad so that that stresses me out you know for my future as well.

Interviewer: So you believe that you could possibly get like disablement from this knee

P14: Yes

Interviewer: What about long term. What about when you're older, what's your view of what is going to happen then?

P14: I try not to think about it too much but I'm trying to accept the fact that I will probably need like a total knee replacement or something like that. But yeah, I try not to think about it, but I do try to stay like active or I guess I'll try and do things to try and prevent that from happening, but I will probably need it.

Interviewer: Why do you say that? has someone said that to you

P14: No one has said it to me. It's just because there's just been so much trauma to the knee. So I think it would just develop arthritis and I'd get some pain in there and all that kind of stuff. I just think it's gonna happen.

Interviewer: Where does this message come from?

56:55

P14: I guess maybe working in the industry maybe, because I used to do groups for hip and knee replacements and not that anyone's really talked to me and said to me it's because I had 3 knee recos or anything like that. It's just you know, it's just happen to a lot of people so I don't know. I just kind of think that's probably going to happen to me. I'm going to probably going to need one.

Interviewer: Is there anything you could do to change that

P14: to change the thought?

Interviewer: To change the belief that, that is going to be you? is there anything you could do to change that?

P14: Yeah. Well, I guess if it was like studies or evidence to show you know, that that might not potentially be the case, you know I probably won't believe that. I think the main reason why I believe that is because of that sharpness in my knee still, so if that wasn't there I probably think it would be okay, I probably would still think I would still need it. Anyway, just because I do sometimes get that general swelling and pain throughout sometimes, but yeah, If a treater or someone that knows the area well was like you might not need one I'd probably believe it a bit more but I just feel like this point. That's probably might happen my future.

Interviewer: So if the sharpness could be addressed in some way have you gone and seen anyone specifically for the sharpness?

P14: Yeah, I have and physios generally think it's nerve pain which I know it's not because I can tell the difference between nerve pain and this type of pain. I don't think it's nerve pain. So I have seen physios and I don't trust a lot of physios, but Im kind of picky with who I go to just because I guess some physios I feel like don't fully, or they have an air of knowledge in one area that I don't have knowledge in particularly what's happened to me or anything like that. So I do, I am quite picky with who I see I guess and so once I did see that Physio and they told me that, I was a bit like oh, nah and then I kind of knew, I know it's not nerve pain. I think it's something else. Yeah, just kind of put me off seeing another one. Yeah, I guess the only way that someone would really know what's going on is if we got a report from the specialist and then they could kind of determine that because I can't really, I don't fully know exactly.

Interviewer: So a report from the specialist meaning you would need to see another scan?

P14: Yeah, maybe another scan. I'm not too sure it would show up fully.

Interviewer: Okay. So how would you say the perception of yourself has changed throughout the course of these injuries?

P14: Yeah, well I guess my confidence physically has gone down in terms of my physical ability because I was quite, I'm quite confident. I was quite confident. I guess you know, physically what I could do specially like on the field and everything like that yeah, and just, yeah, I feel like I'm quite coordinated and everything like that. Look I was but maybe not so much, not my lower limbs anyway, not So Much Anymore.

Interviewer: So you have lost a bit of that confidence in your physical ability. What about other people's perception of you, do you think they've changed, you think other peoples perceptions of you have changed?

P14: I haven't really thought about other people's views actually. Probably, yeah, I'm not a hundred percent Sure. Yeah, just another thing about like I guess my mental health as well. Just I remember as my knee was recovering. This was after the last surgery. Yeah, I would get really frustrated and really actually quite down, even when I was walking and got that physical ability back. I was getting quite frustrated with the pain and I would often just cry

sometimes just because it just I was just like this pain is never going to go away and you know what have I done, Over time I've kind of just accepted that its going to be there. But initially it was quite hard. I remember at work at Eastern Health I would just walk down the corridor and I would just get that sharp pain in my knee again, or I couldn't really fully extend my knee properly just because it had been straight in a zimmer splint for so long, or I couldn't sorry bend it or you know completely straighten it. I wouldn't completely straighten it because of the pain in my knee so I would be walking with a bit of a limp and so even though I physically tried to be aware of it and change that, I know I was aware I was still doing that because of the pain and that actually really got to me quite a lot and sometimes I would just cry, just because I would really be frustrated about all that. Yeah quite difficult the last one.

Interviewer: During that time where you sleeping soundly or were you getting a lot of thoughts about the knee function?

P14: Yeah, I wasn't sleeping well during that time because yeah, I was staying up with thoughts, you know, like anxious about what my future was going to be like with this injury and all that kind of stuff. So, yeah.

Interviewer: In terms of that what did you believe, I mean you mentioned this multiple times, but did you believe there would be ongoing issues or what were the thoughts that were kind of the worst, if you don't mind me asking around that time.

P14: So what was the worse?

Interviewer: What were the thoughts that were maybe the worst?

1:04:30

P14: They got pretty dark. Yeah, it was more. I remember I got bombed it got pretty bad. I haven't really told anyone this actually when we're in Queensland like when I was in the Zimmer splint for my friend's wedding we were quite high up and I just kind of had like a quick thought of what if I jumped, it got pretty dark but I kind of brushed that away straight away and was like, what the hell am I thinking, like no way blah blah blah and then after that I was just trying to focus to be more positive, but it got pretty bad. Yeah during that time, there's no way I would have done anything but yeah just a thought quickly popped in

Interviewer: so is there a lot of other things that coincided, you mentioned the wedding did anything else coincide with that last injury.

P14: Not that the wedding was that traumatic like it was fine, you know just a small wedding and all that kind of stuff. I don't think that was a big deal that I wasn't a hundred percent. It was just more the fact that I was in this situation and like I couldn't do anything or you know, like I was on crutches and just struggling so I think it was just more… it had to do more with my injury and the rehab and what I was going through it wasn't so much that events were happening or anything like that. It was predominately because of the injury. Yeah.

Interviewer: Did you feel like you had any control of how that process was going along around that time?

P14: No because I was in the splint so I couldn't really do anything, you know couldn't try rehab myself and then I guess I didn't really think about seeing anyone for my mental health at the time, to be honest it probably took me a little while to come around to like psychology and like seeing a psychologist because I had seen some in the past and I just felt like not to do with injuries or anything like that was more to do with my sister. What happened to my sister, but I just felt like it wasn't… Maybe the psychologist just wasn't right for me or something. I just didn't feel like it was very productive like I've changed my views on that now also it is probably a cultural thing my parents didn't really believe in psychologists and all that kind of stuff either. So probably had their beliefs, you know of that in me, so I was just a little bit reluctant or didn't really believe in them as much anymore like at that time. But yeah, that's all changed now. Looking back that now do you think that would have helped if someone maybe the surgeon or whoever else had just run through how you were feeling after the injury the last time?

P14: Yeah, probably or maybe recommend, I think, probably would have opened my mind up a little bit more. I don't know if I still would have seen one. Um, but yeah, I guess it would have helped to have known the other people who have kind of gone through that as well. You know just no one really talks about the effects of… I think this is why the studies really good because no one really talks about psychologically how you know, you know the effects following an injury has

even with my friends like we talked about a little bit but not really in depth.

You know, yeah,

Interviewer: Why do you think that is? Would you guys talk about it or do you think it's something that's just a little bit too taboo or hard to discuss or why don’t you think you have had a chat about it with your closest friends?

P14: Yeah, I'm not too sure. We haven't talked about it actually like we've talked about it a little bit but my friend Monica, you know, I don't think she ever returned to sport because I'm assuming it's the fear but I'm like we haven't really spoken about it that much actually, I'm not too sure why, like when she was going through it I had talked to her about it, but just not in depth about how she's mentally feeling, not that I can recall anyway. She might recall differently. But yeah.

Time: 70 minutes 45 seconds
