# Supplementary material for: Understanding Fear after an Anterior Cruciate Ligament Injury: A Qualitative Thematic Analysis Using the Common-Sense Model
Source: Int J Environ Res Public Health. 2023 Feb 7;20(4):2920. doi: 10.3390/ijerph20042920 (PMC9957354; doi:10.3390/ijerph20042920)
Supplement: Supplementary file 1 [file ijerph-20-02920-s001.zip › File S1-P15 Transcript.pdf]

P15 Transcript

Interviewer: Yes, awesome. All right, well thanks for having the time to have a chat. Today you will be participating in a study that is researching fear post ACL injury. The interview will consist of a series of open-ended questions that will explore your fear, levels of distress, and your confidence after your injury. Please feel free to say as much or as little as you want on any topic, and if you feel uncomfortable at any time let me know and we'll move on. Does that sound okay?

P15: Yes.

Interviewer: Awesome.

P15: Yes, more than fine.

Interviewer: Tell me about your ACL injury.

P15: I did it skiing. I'm not sure if it's within the same action that's similar to yours, but I did mine essentially on the landing. I was at Hotham and I'd like to say that I'm a decent skier, but then I decided to hit the park, the Terrain Park, which I've got no experience in. Going down the mountains and whatnot I'm very familiar with and I'm very comfortable with going down and just bombing a hill, but doing tricks and jumps in the Terrain Park was something that I've got no experience whatsoever, but I just decided "Hey, it's almost the end of--" It was the end of our trip.

We spent about four days at Hotham, and it was the last half an hour, so we decided just to have a bit of fun before heading back home. We were going up and down in the Terrain Park

and the incident happened after landing two consecutive jumps in a row and hitting that third ramp with a lot of pace. It was three consecutive jumps I had to hit. The first one obviously the one that you land before you pick up more momentum and speed and you carry that into the next jump. After landing the first one, I picked up more speed and momentum, hit the second one, again picked up momentum and speed. Then by the time I hit the third one, I was a bit out of control. I wound up and I landed.

I initially landed with the right foot down the mountain, but then I just lost it. My right foot stayed straight down to the mountain that was great, but then my left one decided to scoop the other way. Then as I was going down, I just kind of did the splits and that's how I did my left ACL. Yes, I just landed very awkwardly. My right leg faced down the mountain, it was all right on my leg, my left one didn't. Then as I was going down that ramp, it just did the splits and I crashed, I tumbled a bit. That's how I did it, I did an ACL and I also did MCL as well. Then in terms of treatment, which I'm sure we'll get into, it was just the ACL that I got reconstructed. The MCL, after consulting with the necessary specialists, we decided to wait for the MCL to heal on its own and then we just worked on the ACL. I did the ACL and MCL in a skiing incident, it was just a landing on a particular jump.

Interviewer: In regards to that experience in that questionnaire, you mentioned that you had a little bit of fear associated with your ACL injury. Do you want to tell us a bit about that?

P15: Yes, definitely. Do you mean as in fear now like post-op or was it more so fear in relation to starting with sports just in general, I'm assuming?

Interviewer: Yes, just in general.

P15: Yes, definitely, I definitely had fear, I think the main concern was having to rehab all over again. Having to rehab is one thing. The financial cost and going through getting it

reconstructed is another, a big one for me because with my background financially, we're not as well off as some others may be. I think by just following sports the way that I do and knowing how difficult it is for someone to come back from an ACL injury, I think just knowing the severity of this particular injury having done it, the risk of redoing it, and then, I guess, decreasing your chance to play in any physical activity or particular sport or game.

Yes, there's a number of concerns, but it's the risk of redoing it, having to go through rehab, the financial cost of doing it. It's also a 12-month rehab commitment as I'm sure you know. That would be number one for me is having to do rehab again, the time and the costs associated with that.

Interviewer: Can we break down that apart a little bit, what would the cost for the first part, mean to you for redoing it?

P15: Quite a bit, almost to the point where if I was to do it again, I would almost have to really consider undertaking elective surgery in the private sector. Depending on where I'm at physically, if I am in a competition or if I'm in a team sport going for a premiership in a particular season knowing that we've got a good group, that might push me to get it done more quickly, but right now if I was to redo it at this point in time, the cost would almost put me off getting re-elective surgery and having to wait in the public system where there is almost no financial burden. Yes, quite a bit financially.

Interviewer: Another part that you mentioned was the severity of injury, you said, "Knowing the severity of the injury," what did you mean by that?

P15: I think it's just growing up watching sport, playing sport, knowing people that had done ACL injuries, and even just seeing idols and professional athletes, the people that you look up to on a day-to-day basis seeing them with ACL injuries and then just hearing their stories that

they can't go back and perform at a level which they used to. Look, you hear stories that people get back to performing at their very best, but I guess, for the case studies that I've seen, that happens very rarely very, very seldomly.

Just knowing that you've done it, it's difficult to get back into the sport but also, playing at that lower level is crap. For me, I've done it once, I know that it's really difficult to get back to where I was. I know other people might have a different mindset, but to me, it's almost like just on a day-to-day basis if I was to quickly change direction or find myself hopping on one knee, it's always that I'm always second-guessing myself that "Hey, am I ready to do it again?

Even I'm starting to think of that now that if I was to redo it again then it would really affect my quality of life. I think the just severity, just knowing that doing it the first time is bad enough, it's constantly on my mind now in everyday life whether that's walking, running, standing up, every now and then I feel like there's tension in the knee. I think knowing that I've got these lingering issues after the first time I've done it, I just know that it's only going to get worse if I keep redoing it, and the fact that once you do your ACL the chances of redoing it, I'm not really sure what the percentage is in the scientific sectors, but I'm definitely sure that you're more susceptible to rupturing it again. Sorry, it's a bit long-winded.

Interviewer: You mentioned a few things in there, the professionals and the idols you mentioned or you mentioned you knew other people that had done it, what kind of messages have you been getting from other people around your injury?

P15: Messages that I've received or messages that people that I know have received?

Interviewer: Yes, just messages that you have received in from various sources, what kind of stuff were you receiving?

P15: Definitely I'm extremely fortunate from a working capacity to be in a position I am. I work at the Collingwood Football Club and I'm constantly in very good hands. My physio is the club physio. My surgeon is a very good friend of the club surgeon as well. Just with that I've had the chance to speak to some of the Collingwood players and some of the athletes there. Again, I'm very fortunate to be in a position I am to have access to those resources. Just knowing the work that they put in makes me feel as though there's a lot to do.

To answer your question in terms of the messages that I've received, the number one key for me was just stick to the rehab, do the rehab. I can't even count how many times I've been given that advice was, "Stick to the rehab." Other comments were, diet plays a massive factor. I'm jumping around with all the questions, with things just come in my head. One thing that popped up that I relied on quite heavily a suggestion was, to get as much bodyweight on it as soon as I can, which sounded odd, but I did it and it worked for me.

It was practically just as soon as I got up trying to move around self-assisted, even though if it means limping or anything, but I was told that the more bodyweight you put on it daily without having that assists, the better it would be. That was a feedback that I got and I really used that and I'd like to think that really helped, rather than the first couple of days out of surgery, just staying bedridden. I was actually already walking around the place. That was another unique comment. The number one thing was do the rehab.

Also, after the three months period, I had the opportunity to compare with other people, with athletes about where they were at three month mark to where I was and feedback about-- For me, specifically, I was always progressing really, really well. A particular comment was, was

my power was great in terms of getting the power back into the rounded muscles, but my balance is awful. I didn't have that prior to surgery. One of the feedback was just to continue doing the exercises, specifically that was tailored to me. It was all about rehab and sticking to that, keeping to the exercises. That was on the physical side. There was also feedback about my mental state. It was more like just giving me confidence in what I was doing was right and that was key to me. I get very agitated, I get nervous quite often about anything I really am a stress head. I tend to overthink things way too much. When it came to something as important as my ACL rehab, and because I'm on sports so much I really wanted to get back onto the field, onto the mountains, or whatnot. I often compared myself to other of people.

As I was rehabbing, objectively, I always thought in my mind that I wasn't doing enough. I was always comparing myself to other people saying, "It's true. Should I be feeling this way at this particular stage of my rehab. Should I stop? Why can't I extend my whole leg? Why's my knee still have a bit of a kink stuff like that. I'm taking an individual approach. Some of the feedback I received from a mental point of view was, "Hang on, you're doing really, really well." I can't quote any one at this stage, but it was really good feedback about how I was progressing, putting self-confidence back in me.

14:56

This was all the physios and the surgeon that I saw on the three month mark, six-month mark, and the 12 month mark.

Interviewer: Were they also giving you this advice at the initial phase as well?

P15: What was that, sorry?

Interviewer: Were they giving you this advice at the initial phase as well?

P15: Yes, they did, but it didn't have as much of an impact than not untill about a three-month mark. Did I really take that? I was always like, "Yes, I'll do it." I've never gone through this process before. I've never had surgery ever. I've never broken a bone, never done a muscle or ligament or anything. This was the real big thing, what a step to take. I didn't know what I was getting myself into. It was the three-month mark that really hit me. I was starting to compare myself, "Should I be able to run at this point. Why is there still kink on my knee, I can't fold it, the extensions are not there.

When I do hamstring curls, my hamstrings killing me and whatnot, at the time I can't kneel down without it hurting, my but can't touch my ankles, that kind of stuff. The feedback was really good in terms of pushing me through the three, six, nine month period. I can't really quote exactly what it was, but it was more so just really focusing on myself. It was an emphasis on everyone's different, everybody's different. That really put me at ease. It will come as we keep talking. It was mostly just really emphasizing the self-confidence in myself. Objectively, these guys know exactly what they're talking about, they've seen hundreds of knees before.

To hear that I was progressing as well as the next person, it was almost vital for me just being the stress head that I am. It was good.

Interviewer: In that initial phase, you mentioned that you're a bit of a stress head. Were you fearful at the initial time of the injury?

P15: Yes. From injury to diagnosis, it was about a week. Obviously, I'd crashed at the mountain and just speeded off, which wasn't a good idea, but I did. We went home and then I went straight to the ER because once I got home, it was a six hour ride. I noticed that all of a sudden, coming out of the car, I really couldn't walk properly, I was just limping all over the joint. I went to the ER straight away. Initially the diagnosis was I've just sprained my knee, it wasn't too bad, then they were like Stil getting an MRI. But because the initial diagnosis wasn't super urgent, I didn't get an MRI until the week after.

Initially, if you ask me what was going on in my head, I was fine, I was absolutely fine. There was a bit of ACL In my mind, in my mind I had no idea I had done the ACL. I already brushed it off a little bit but then after speaking to the doctors at the hospital, being confident that it was just a sprain, I was all good. Going through the MRI and then seeing the follow-up result and hearing it was an ACL, I almost just froze. After that, it absolutely hit me. It was all different types of emotions. Like I said, I've grown up as a massive sports fan so I know about the injury, I also studied sport. Again, I know what the repercussions are for an injury as such.

19:20

I think in the moment which I still remember to this day, being advised that I had torn my ACL and MCL, I still remember sitting where I was in this specific room and being given that diagnosis. I almost laughed it off at first, I thought it was a joke, because I spent a week with this knee. I'd love to share just one thing. In that week in which I was waiting for my

diagnosis, I was all good in terms of I was still walking around, obviously still hobbling and stuff, but I was still walking around and the fear wasn't really there.

It must be the power of the mind or whatnot, but as soon as I was told I had the ACL, the week after it hurt like a bitch. It killed, it hurt and I couldn't really walk or anything, but it was funny because it was just the week before I was fine. I don't know if it's just the mental thinking, just having that positive mindset that prior to knowing that you have done the ACL and really thinking that, "Oh, it's just a sprain. It's nothing serious." It could go away in about three weeks. Just got to get icing and doing these exercises and whatnot. It didn't hurt at all.

Then being told that I did do an ACL and still doing the same exercises that I was doing in a week before, it was almost impossible. I think my mind just gave up in a way and it just allowed my body to take over. My mind just stopped fighting because I knew that I had done the ACL. I don't know how to word that any better, but it was just weird. It was like I was fine, but as soon as I was told I had an ACL I didn't get depressed, but mentally I wasn't in a strong state and that really affected the pain that I was starting to feel; whereas the week before I was in a really positive attitude.

I was thinking very optimistically that I hadn't done such a serious injury. What I had done was something still significant, but was minor. I could get over it. It didn't need surgery and stuff, but yes, I don't know how to put that in any other way than that.

Interviewer: You emotionally found it very challenging. What do you think, reflecting on, what is the biggest thing that made it so emotionally hard?

P15: For me, the cost is always one thing, just given our socioeconomic status and how our family is situated at the moment. Again, that's just me speaking, I know other people are in different circumstances and situations financially. That was definitely one thing. Obviously,

because you are not prepared for something like this, it was a big surprise. Also, the other thing was the 12-month rehab involved, not being able to play the sport that I like.

The sports that I played do involve a lot of changes in direction. I am not sure if you are familiar with Ultimate Frisbee but I played that. There is a lot of direction changes and whatnot. I also played a bit of social netball as well. Just knowing that those sports, in particular, are very high risk to the knee, in particular, the ACL it hit me hard because I knew that what happened has happened, but for the rest of my life, I have to be really careful in the changes of direction.

If I played in a sport which didn't involve that I don't think it would hit me as hard. I don't know, If I was an archer or if I played sport which didn't involve really high-intensity change of directions, 360 spin events, and whatnot, then it would be okay, but just knowing that I love the sports that I play and I really want to get back to that but knowing that it won't ever be the same or at least it will be the same but I'm constantly thinking about it.

Just that burden that I know that I will have to carry for the rest of my life hit me as well. It was cost, it was 12-month rehab not being able to play the sport that I love, but also when I get back to play the sport I love I've always have got this cloud over me. Whether I'm feeling 100% perfect physically, I just know that mentally I'm always going to be always questioning it. Whether that be landing and my knee buckles or my knee caves in or whether that's change of direction, I step wrong and move the other way and my knee gives in.

24:20

I am sure you know the different movements that can trigger the ACL to snap again.

Interviewer: At the time of this initial injury when you were going through all those thoughts, did a health care practitioner at all ask about your mental side of things?

P15: It's a good question. The fact that I've got to think about it probably speaks volumes. Very minimally. When I first spoke to my physio, I remember him asking me about what my goals were, what I want to do. If you call that asking about my mental health then yes, but I did share how I was feeling, how I was nervous about undertaking elective surgery. I also questioned because I've read a few studies that there have been some athletes that don't undertake or don't undergo a surgery and they are still able to return to full competitive sport.

I was really questioning and I asked a lot of those questions, but I don't know if I was asked about my mental state in terms of fear, confidence, anxiety and that. It was probably touched upon in terms of the surgery or when I naturally just asked how should I be feeling and just the nerves that you normally get with that, but in terms of return to sport and everything, it was probably brushed upon very mildly or very little. I did see a number of physios. That was just my first physio. Not naming names, there was a few physios that I saw. My first physio that was the person that I got into the discussion that we had and then my second physio which I saw after surgery, we started to mentally ramp it up.

26:59

I think for me and my experiences with the health care practitioners the mental aspect only came in-- Sorry, I won't say only, I would say heavily came in and started to be a real big

focus of mine was in the rehab stage. It was more so getting mentally prepared to go back into sports. It was like doing the rehab, doing the exercise I do, being asked to do box jumps, being asked to do single knee hops and stuff, and because that was early on in my rehab, I was quite fearful that I wasn't ready to do this that was probably at the six-month mark and that's when we started talking about mental advice, mental health, and getting that confidence and that perception that I can do it. To answer your question, it was probably wasn't more at the initial diagnosis that we talked about mental health and that advice. It only came in probably two spurts, just before surgery and probably six months after surgery. Just before surgery was more so just to generally know things so i.e, the normal questions that you get with surgery about, "Is this the right method to take?"

"Am I going to experience some sense of tingling in the leg for the rest of my life?" just being nervous around all the implications and side effects of having surgery and questioning whether it's going to be successful or not. That was pre-surgery and then post-surgery didn't happen immediately, but at thta six months when I started getting back into exercises which challenged my knee, mental health has played a huge part since then. Being positive and really being self-confident that I can do this and I can jump on one knee, I can land on one knee without giving up a knee. To answer your question, not so much in the first initial part, but more so towards.

Interviewer: What was the response in that initial part when you were asking all those questions? How should I be feeling? Their outcomes.

P15: I wish I had of gone to multiple practitioners at the time. It's hard to compare what it should have been, but how was the response? It was fine. I got answers to every question I had, but I didn't feel as though that particular practitioner went out of their way to really put me at ease.

And It's not to feel as though I was just a number. I never felt unease, I always felt like I was in the right hands. Me probably saying that I didn't get all the advice and the mental health aid that I should've gotten probably puts this particular person in a bad light.

I don't feel that way at all; in hindsight, I really do think that it was a good experience, but I guess asking the question that you're asking now and upon reflecting, yes there could've been a bit more but in no way did I ever feel as though, at the time, that I wasn't in the right hands, if that makes sense. I felt fine, I got all the questions that I was asking how I should be feeling and the implications long-term, and I was often worrying about can I return back to the sport and just being anxious and nervous about that.

Look, I got all the answers that I asked but at the same time, I knew them all, it was just more so just asking out of the sake of asking. I had studied sport and I've read quite a bit on this particular injury. I knew the answers already prior to asking but because I'm a stress head, I was asking them anyway. I don't know how else to answer that by saying I got the answers that I needed to hear, but I didn't feel as though they went above and beyond. Sorry, I can't be any help. Fair enough?

Interviewer: That's good. Thank you. How did you manage the stress? You've mentioned the anxiety How did you manage that throughout the ACL injury journey?

P15: I didn't like rehab, which I never knew was a thing. When I first done it, all I was thinking was, "Get the surgery done ASAP and then really smash into it in the rehab," but like I said I'd done the MCL prior as well. I did MCL and ACL and upon speaking to surgeons and physios the best plan for me, just because I wasn't a professional elite athlete, there was no rush to get me sorted right away. The plan of attack was just to let the MCL heal on its own and then once that heals, get the ACL done.

The MCL healing took about an eight-week natural process. For me, I was like, "Well, what do I do in the eight weeks and everyone said 'Prehab. Do the prehab.'" I had no idea what prehab was. I think what got me over that stress and anxiety was I was doing the prehab, I was very into it, I was very strict on it. I think my way of thinking was, "I want to get back to--" I'm sure everyone else feels the same way, but everyone when they're enduring such an injury want to get back to where they were. In that mindset, I wanted to do as much as I can to get better.

If you put in-- [laughs] I don't know the quote. I did a lot of prehab and I was seeing the physio quite often leading up to the surgery, and I was advised that I was in a really, really, really good position and waiting to go for surgery. That put me at ease, it was almost like a distraction from the mental side of things was seeing the physical results. Seeing the physical results from doing the prehab and being able to extend my leg completely before going into surgery and then obviously coming out of surgery, you go through all that again and you have to straighten it out and whatnot.

It was just getting into a routine for me, that was why. Then while I was doing that, I reached out to all my ACL buddies, which is sad to say because there's more people who have done the ACL than there should be. That's just my opinion. I reached out to everyone that I knew that had done the ACL and hearing their stories, getting advice from what worked for them. Then after all that, collating all the information and feedback that I've received from all my mates and colleagues that have done the ACL, it appeared to me that yes the rehab was consistent with everyone, but almost everyone had a different recovery process in terms of a timeline.

It was weird because it was uneasy to hear that everyone was different but at the same time, it was empowering to know that as long as I just focus on myself, the more I put in it, the more

I'll get out of it. To answer your question, it was prehab but also seeking a lot of advice and feedback from other people, what worked for them, what didn't work for them, taking notes of that and then applying that to myself and seeing what worked. I used the prehab period before surgery as a bit of a trial and error, and then I smashed it out.

The first few weeks after the surgery, I was up and running. I was in the gym already, so apparently that to some people, that was unheard of in terms of how quick I bounced back from surgery. To me, I account that to all the prehab that I did. I like to think that all the effort that I put in prior to surgery helped me really in the first few weeks coming out.

Interviewer: Then as you continued along so towards more the return to sport, you've touched on it a little bit, but how is the stress and the anxiety? Maybe just before that question, how do you describe, what is the stress? You mentioned stress and anxiety, what do they mean to you? Like, what does anxiety mean to you?

P15: In my opinion, the stress and anxiety to me from an ACL point of view and injury, it's having to think about it in everyday life. Anxiety to me is this feeling of helplessness in a way where-- I don't know how to word it. It's just helplessness where it's really you're struggling in a way which there is no answer to it. That's what I think when I get into these bad moods and I do experience some level of anxiety.

37:52

It comes up initially when there was anything to do with the knee, so when that was even just running and seeing an even or a sloped ground, just starting to really think that, "Oh this is

going to hurt." It's just the unnecessary-- I don't know how to answer this question, it's a good question. In short, I define anxiety in a way where your mind gets uneasy over an apparent stimulus. For me, that stimulus is anything that could affect the knee, anything that could make it jerk or make it lockout.

I often do get nervous and anxious throughout the whole rehab period when it came to almost anything, whether that's getting out of bed or jumping up and down or lifting something, bending the knee.

Interviewer: What would have been the effect then if you had have affected the knee? What would have been the result of that?

P15: If I hadn't of? what was that, sir?

Interviewer: How did you had have affected the knee if you had have got out of bed or jerked it you said?

P15: Right

Interviewer: What would have been? What would that have meant to you?

P15: Shit, really bad. What's really bad the way that I was putting it--

Like I said earlier, I often compare myself to the very best. I always just question myself, "Should I be progressing much more than what I'm progressing currently?' that is what I was constantly asking myself. "Should I be feeling this way?" and whatnot. Any time that I did feel anything that twitched the knee, or it buckled or it didn't feel right in that first six months, it was almost as detrimental mentally than it did the knee. I was, like, "Whoa, I've got a long way to go." every time, every time.

If I was to get out of bed and when they were just tweaks suffered and I felt it, then I'd be, like, "I shouldn't be feeling this. Why is it feeling like this?" and then the rest of my day, I'm just thinking about it. "Well, my rehab is not working and I'm questioning everything again." That just brings undue stress and I'm anxious for the rest of the day in everything I do, because of that one internal-- that movement.

I don't know if that answers your question. I just get an overload of nervousness and anxiousness for the rest of the day. I'm thinking about all my movements, but it all leads back to my progress. I often think that I should be a 12-month knee when I was only a 3-month post-op knee. I think expectation plays a huge part in recovery. Setting realistic goals, unlike myself, I was often comparing myself to other people. Just going back, when I was saying that throughout the rehab process, just getting that reassurance from the practitioners, that I was absolutely killing it-- It was great for me, but it was also very bad for me.

Hearing that I was killing it, objectively, during the tests I did and knowing that I was doing extremely well, because I was told I was doing extremely well, but then going home and every now and then experiencing the low levels I did just questioned myself, "Am I going to have to live with this kind of feelings for the rest of my life, if I hopped out of bed the wrong way, or if I jumped up and landed a bit awkwardly and feel a tinge, am I going to feel this way? I've been told that I've absolutely killed it to this point, so to me I couldn't get any better but why am I still feeling this way?"

Every time it went awkward on me, it was really bad mentally, because I absolutely overthought everything, I questioned everything. Yes, I started to panic because just thinking, "Well, if I'm told that I'm in a really, really, really good position and I'm absolutely pulling my weight, then why am I still feeling like this?" I tend to give you very long-winded answers. I'm sorry.

Interviewer: Thank you, thank you. No, they're great. You've mentioned your health professionals again. Did they specifically ask you about any psychological considerations during early three-month you return to sport, or did you bring it up with them?

P15: I would bring it up. I would bring it up. Sometimes I got asked a little bit, but I don't think I gave them the chance. Just who I am being a stress-head, questioning everything, overthinking everything, naturally I just lead to asking questions that emulate how stressed I'm feeling. I don't think I gave them a chance, no. I just went straight for it. I was just, like, "Hey, should I be feeling this way?" or, "My knee tweaked this way. I'm nervous. I'm scared to jump on that exercise that you told me to do the other week. I only do half of the reps, because I feel as though my knee's caving in." that kind of stuff.

I don't think I gave them a chance, but in saying that, towards the return to sport it was often a focus that they brought on to me in terms of saying, "How are you feeling?" At the 12-month mark-- Maybe I don't know how it works. I've never seen and then consulted someone else that has had an ACL from surgery throughout the rehab period from surgery through the 12-month and return to sport, so I don't know what those conversations and when those conversations should be had. To me, when I was returning to sport, there was a lot of focus on my mental health and just being self-confident. That was a real big focus that was put on to me which I took.

Again, to answer your question, it was more so I was asking the questions of mental health and advice on stress, anxiety, and stuff, but again like I said, I don't think I gave them a chance. I was often coming in with questions locked and loaded, ready to go. [chuckles]

Interviewer: You've also mentioned confidence a few times. What does self-confidence mean to you?

P15: To me, it's everything throughout the rehab process, because if I'm confident in what I do in the gym and confident in what I do around the track, then I know that I'll bring that confidence into play-- into return to play, into sports onto the field in team sports. Just that confidence of knowing that my knee won't give in-- I celebrate the small wins, as I do kill myself over the small losses about the little setbacks and whatnot. I definitely do celebrate the small wins, say every time I do get a… or every time I jump or land on one knee, and it doesn't give in, I'm really, really happy about it. I take all of the confidence out of that.

47:25

I think it's almost just psyching myself up, knowing that I've got it. It's almost like I'm overdoing it in terms of really making myself feel good to a point where it becomes a bit artificial, but I still do it, because I need to make myself feel good. I then bring that confidence back into sport and onto the field and what not.

Interviewer: Currently you're quite confident, are you, in sport?

P15: Lockdown hasn't helped because sport has stopped. I always feel as though I'm cheated. I only did my knee 14 months ago. I only did it 14 months ago. At the 12-month mark, which was September, I was ready to get back into sport completely, because sports is slowly starting to come back. I was still testing the waters there. I do feel betrayed in terms of I just had a wish-- Everyone wishes that COVID wasn't a thing, but selfishly I wish COVID also wasn't a thing, because I could actually see my knee from a full 12-month process.

I was locked out of the gym so obviously, I couldn't go to the gym. I was doing a lot of bodyweight exercises between month 7 to month 10 of the ACL rehab program, which didn't help, but again-- I know should have probably stated this. but at the sixth-month mark with I saw my surgeon, I did express my concerns that I was missing too much rehab because I couldn't get into the gym. I didn't have access to a gym. We have a gym at Collingwood obviously, but staff weren't allowed because of COVID protocols. It was only high performance and the boys could use it. I couldn't use the staff gym. Obviously the commercial gyms were not there because of COVID.

I did mention that. I was, like, "Okay." I felt incredibly concerned that I killed my first six months of rehab and I'm flying currently according to all the tests, but I've all of a sudden hit a rock-- hit a walk sign. He then Flipped it on me

49:58

I remember my surgeon saying, this is probably a blessing in disguise, and I don't know if he said this to make me feel good. It did work. What he said was-- Again, probably because, I needed to hear something like this, and that's why I believed it, and I bought into it so much.

He mentioned that the graft, which was my hamstring. He said, "The graft needs to take time to strengthen up." He said, in these last six months. He then drew diagrams, and he showed me these little graphs and whatnot. He was saying, "This next six months, despite not doing anything, in the same vein, it's allowing that graph to strengthen completely. He reassured me that despite not having or not doing, all those weighted, loaded exercises, it would be okay,

that it would give my graft time to heal. Then, once I get to hit the gym, again, the graft would be fully strong, and I can always start again.

Again, I'm not sure if he's making that up, I don't think he's making it up. I hope he's not making it up. It definitely did help because I then took that attitude. I was like, "Okay, well, if I'm locked away from the gym in one light, or at least it's allowing my graft to heal." I looked at a different aspect. It wasn't like, I stopped the rehab completely. I was still doing body-weight exercises, I was doing therabands

51:33

I was doing body-squats and whatnot. It was just the way that stuff which-- Yes, it was just all the weight and stuff that I couldn't do, and all the stuff in the gym and whatnot. I just used his guidance, and I trusted his words that I looked at it in a different way. Initially I absolutely doubted it.

Interviewer: Yes, it's giving that message. Currently, now how do you respond with fearful movements or things that are challenging?

P15: I still get nervous. Absolutely, because I have been returned, well, I haven't played a competitive game, of anything of netball, or 40-year soccer, or anything. I've only just thrown in at the park, or kicked it at the park, or just really socially. I haven't had the opportunity to test it, but when I am out there, I noticed that initially, yes, I was definitely thinking about my foot plays, the landing of my leg and knee, really soften the blow, or landing on my opposite leg to take that weight.

I was doing really unnatural stuff, but I've noticed over the last few weeks, it started to just pop out, in terms of, I now don't even give it a thought. It definitely was a thing initially, and

I think that's because I'm now used to that movement. God, the first game back, I think I'll be absolutely fearful. I think it's different, and you know this, it is different to kicking a ball socially, than to be a 360 environment where there's people actually charging for you, and there's competitive, there's that competition.

For me, at the moment, now I'm really focusing on that self-confidence that I can take the next level. I feel I am ready to take that step, but until I do play a game and get that under my belt, I think I will always be, I will still be fearful until I just get back into it.

Interviewer: Okay. Has your perception of yourself changed because of this injury?

P15: Yes. I'm a lot more resilient, I'd say. I'm also a lot more determined prior to ACL. I've never had any routine of sort. The ACL has made me get into a routine of rehab, hitting the gym and number of X amount of times a week, of getting up and doing this, and that. There's a few factors that go into it. I won't lie by saying that every now, and then, I think that I have to do rehab because of the financial burden that it costs to do the surgery, so every now and then I am motivated to do rehab because I want to get what I put into work without it, in terms of financially. I want to make the most of this investment of doing an elective surgery and that, which isn't the best motivation. Spending money isn't the best motivation, but sometimes, I'm not going to lie to you, I do think about that. Another thing is, yes, just knowing that if I don't get it right, in the first 12 months-- I've heard that your first 12 months of rehab is the most important because it defines how the knee heals up following that. Just knowing that I really want to get back into sports, Knowing that I have to put in the effort now in the short-term, to have the long-term gains for the rest of my life, that was also a motivating factor.

I think, my perception, my resilience is absolutely changed for the better. My determination to stick to routine has never been stronger. I was never lazy in the past. If you want to say that, that's a silver lining in terms of, the way I think, and the way I do things now, that has taken a positive change since doing my knee.

56:20

Interviewer: That's great. Has the way that other people perceive you then, is that also changed?

P15: Yes, definitely. Definitely. Yes, even to now, all my hopes, I like to think so. I've definitely heard feedback that I'm a lot more stuck to a routine.

Interviewer: Okay. Thank you. There's a lot of good information there.

P15: There's a lot. Sorry about that. I'm just trying to answer your questions correctly. I'll give you a lot more contexts.

Interviewer: Thanks.

P15: Do what you will with it.

Time: 58 minutes 15 seconds
