# Supplementary material for: Understanding Fear after an Anterior Cruciate Ligament Injury: A Qualitative Thematic Analysis Using the Common-Sense Model
Source: Int J Environ Res Public Health. 2023 Feb 7;20(4):2920. doi: 10.3390/ijerph20042920 (PMC9957354; doi:10.3390/ijerph20042920)
Supplement: Supplementary file 1 [file ijerph-20-02920-s001.zip › File S1-P16 Transcript.pdf]

P16 Transcript

Interviewer: Good. All right, well, thanks for meeting with me today.

P16: All right.

Interviewer: Today, you'll be participating in a study that is researching fear post ACL injury. The interview will consist of a series of open-ended questions that will explore your fear, levels of distress, your confidence after your injury. Please feel free to say as much or as little as you want on any topic. If you feel uncomfortable at any time, let me know and we will move on. Does that sound okay?

P16: Sure.

Interviewer: Cool. All right. Tell me about your ACL injury.

P16: I tore my ACL playing Ultimate Frisbee while I was in a tournament playing in Hawaii. I was overseas when I tore it. I didn't get knocked, I just changed direction too fast and didn't really work on my footwork, and changed direction and then pop, [chuckles] so I felt it go bang. I didn't really know what I did, I thought I just sprained it. I tore it. I didn't bother getting an MRI in the States because it would have been too expensive. I just got an x-ray and went to a surgeon to make sure everything was all right. He said, "You probably just tore a knee meniscus. Come back to Melbourne," and then fully ruptured ACL.

Tore my ACL and I tore a little bit of my meniscus, which was repaired. I had surgery two months after I came back. I tore my ACL in February 2019, and then had surgery April 2019. The reasons why I tore my ACL, I was at a party tournament, so I was probably very

dehydrated, did not warm up, was very hungover, and in the middle of a 10 day bender. Just a recipe for disaster really. Tore that, and had surgery, I've done all of my rehab so far, I'm getting back into playing now, and it's been since April 2019, so it's about a year and eight months since I've had my surgery and I've got back into playing at least, and probably due to that, that's due to COVID as well too.

Interviewer: How did you feel about the whole experience?

P16: It was pretty average, to be honest, as in average as in pretty poor. The whole thing from tearing it to figuring out that I'm not going to be able to play sport for a long time. The surgery was fine, that was fun, sitting around for a little bit. The whole thing has just been limiting, to say the least.

Interviewer: The surgery fun, is that sarcastic or was that serious?

P16: No, the surgery was good. It was pretty not painful. Obviously, painful recovering in the days afterwards and all that, but hadn't done surgery before, so coming out of it was pretty straightforward. I was out of work for a few weeks, and then back into it really. It was just time spent recovering and being really into it, I guess. At least I got stuck into to my recovery, so could focus on that, but then again, it was a bit harder later on.

Interviewer: What was harder later on?

P16: The recovery, the waiting, and the, "Why isn't it getting better sooner," type of thing. Especially around the three months, the six months, around nine months when you're starting to feel considerably more able, but if you do anything or you go out or something for a night, and then you just get a massive balloon leg because you're swelling so much. It's just really disheartening, and it brings you down a lot.

Interviewer: Can we break that down a little bit? At the three-month mark, when you're starting to return to sport, what was happening there--

P16: Three month back, I really wasn't returning to sport, I was--

Interviewer: Oh, sorry, returning to function. Yes, my bad.

P16: Yes.

Interviewer: What were your thoughts and feelings at that part? You mentioned it was disheartening.

P16: Yes. I do my training and my physio and stuff at the gym, I'd be getting back into that, just fine. However, I'd want to be more able than I was currently at and that was fine, but it was more so just do physical activity to strengthen yourself swell really badly. Rince and repeat for the next six months or whatever after that. It was just every time either you want to go out or you might be limited by how far you can walk and run around and things like that. Definitely, that was a thing. Knowing that, if I got here, if I'm going to do that, I'm going to get swelling, I'm going to cop it, I'm not going to be able to walk around for the next day or something like that.

Interviewer: How did that make you feel, that thought?

P16: To me, I felt pretty disheartened at least by the fact that I was going to do work. It's more of a time thing, it was really unrelenting that, okay if no matter how much effort I put into doing rehab and things like that, there's this certain time component that physios and surgeons will say, they'll be like, "Oh, you're not up to that yet because you're still swelling." Or, "You're not up to that because you are doing this. Your body's reacting in a certain type of way, so you're not at that level yet."

It was weird because I didn't start really jogging until five months or so, and normally people jog around three months. Then, I'm thinking, "What the hell is going on?" I understood that I wasn't able to jog without it having detrimental circumstances or whatever, but it still did make me think, "Why is my surgery taking considerably more time when I'm doing everything right? At least I think I'm doing everything right. It's just, I'm healing slower for some reason."

Interviewer: You've mentioned that disheartening aspect to it, what does that mean to you?

P16: It's like a pressure essentially. The pressure that you've put on yourself and the expectations that you have just keep on getting-- You have to keep revisiting your expectations essentially, and they'll always change throughout your course. It's not a set prescription of, "This is your ACL recovery, this is what it should look like over 12 months." Whereas, all the goals and the goalposts kept on getting pushed back and back and back and back after setbacks of me either doing something that I didn't know that I was doing, or I twist my leg on something somewhere, I wouldn't know where, or how, or when that I would end up with a ballooned leg for a week. We'd just push everything back. In terms of that, it was just unrelenting in the sense that no matter what goal you had, it would just keep on getting pushed back, and it's circumstances out of your control because your body is recovering the way it's recovering.

Interviewer: During these periods of being pushed back, did any healthcare professional ask you about any psychological considerations or how you were feeling?

P16: No. [chuckles] They say, "Oh yes, cool. ACL simply sucks, the recovery process is long," but no one asks, "Hey, how's it going? How are you feeling," things like that because even the people around me that I was with say my family or my partner at the time, had

noticed that I had changed in my demeanor over the course of time when I had torn my ACL to when I had my surgery to 6 to 9 months to 12 months later down the track of, "Your demeanor has changed significantly. It's not as positive or as fun." I don't know. I didn't think that I had changed because I hadn't really noticed it, but in hindsight, pre-ACL, it's just something a little bit different that I didn't really see it coming, really. I didn't think that I would change in my demeanor, and things like that, and how I respond to other people and how I respond to challenges and things like that.

Interviewer: In that regard, is the way that other people perceive you changed after this injury beyond change in demeanor?

P16: Yes, for sure. I still hang out with the people that played sport with. In terms of people, people make jokes. A lot of my friends were there with me on the team tournament, or whatever, when I tore my ACL. It was very painful and excruciating. I remember every part of it. Everything that I did that was cringy during it because I felt my knee twist, I felt a pop. I screamed, "I heard a pop." Then I tried crawling away from the pain because I was that in pain. [laughs]

I was trying to crawl away, but anyway, that always comes up. It brings a sour taste in my mouth, but it's something that I've had to-- I'd never thought that people would actually rib you, like get stuck into for tearing a ligament, and then how you tore it. I guess I could've been sour and shitty at them still, but it's something that you got to take in your stride, I guess. Either you cop it, or you be shitty about it so In the way that other people respond to me pre or post-surgery, people that have met me afterwards, no issue, but people that have met me before, it's a little bit different because it's always there. It's always two working knees or being seen as the invalid, almost.

Interviewer: Who's seeing you as invalid?

P16: Oh, friends, all the time. It's always ribbed. It's always banter. Anything with two working knees, or running back to sports, things like that. Even during my recovery, when I was involved with sport whether it's coaching, or just being generally involved, having two knees, yes, it's a good rip. It just brings you back to earth every now and then. That really doesn't make you feel welcome or inside, I guess.

Interviewer: Sorry about that, that's rough. Has your perception of maybe yourself changed as well, because of injury?

P16: Yes. In some aspects, it's gotten better. Sitting on your bum for three, four weeks, prior to that, in recovery, you learn you have to deal with certain things about yourself. I went to the gym quite a lot during my recovery, six days a week. I put on more muscle than I ever had. I'm at a heavier weight than I've ever been. In one way, I feel fitter and stronger physically, and mentally as well too because of having to go through this whole ordeal of setbacks, constantly. Just being patient, working hard. Being really, really patient, and tolerant, at least.

Interviewer: You mentioned that, "I heard a pop" when you first went down. Have you had any other recurrent feelings or thoughts or remembering the incident?

P16: Yes. Just getting back into playing. Only this Sunday, or something like that, so I've been doing my return to sport and everything, it's been 18, 20 months since my surgery, so I'm feeling fit and ready, trying my best, and then I turn around one way, and then I feel like a little-- It was so uncanny. It was almost like it was the same movement that I did when I tore my ACL. I felt my knee buckle a little bit. I thought, "Oh, shit." I just went off to the side,

checked my knee out. I was looking around, bending, stretching. There's no way you can tell because it's so split second of just one incident. I was pretty shocked after that, at least.

When I'm running, when I'm playing sport, I don't feel anything. It's normally when you stop, you walk around, and you think, "My left knee is filled with fluid right now, and it's hard to move around, I guess." Just being present that it's there, not in the moment when you're playing sport, but when you're going back into it. Even going back into it, I feel a sense of dread. [laughs]

It's not so much that I'm going to re-injure my knee, it's that I'm not capable of playing at the level I was before. It's due to the time that I've spent away from the sport because of my knee. Not directly because of my knee and the strength in my knee, I know that I have to trust in it, it'll be fine. Trust will only get you so far as correct footwork, slowing down, things like that. It's difficult.

Interviewer: What does that mean to you, not being able to play at the same level, possibly not being able to play at the same level that you did before?

P16: To be honest, it's very-- I think because of all these setbacks and being on the sidelines for two years almost, has already softened my expectations and gotten me used to, "Okay, things aren't going to turn out your way." I'm okay with the experiences that I've had. However, coming back to sport and not being able to keep up with people that I was previously able to keep up with, things like that, it makes me really anxious that, okay I'm in really good physical condition, I've been running, I've been exercising, things like that, but returning back to physical sport and playing that and not being able to play at the same level, it's crushing.

Then again, it's not like I'm not equipped to deal with this because of all the setbacks and things that I've-- The journey that I've been on of goal expectation, push the expectation back type of thing. Even speaking to other people that have done their ACLs, some will tell you. "Oh, it's not the same." Some will tell you, "I don't remember which one." Everyone's different, really, in their experiences with ACLs because of your surgeon and what you get done. You've had a few ACLs, I'm guessing.

Interviewer: In that questionnaire, you mentioned that you've experienced a bit of fear associated with your ACL injury. What does that fear mean to you?

P16: The fear of either re-rupture or rupturing my right ACL. That comes from just the sheer-- I know that the joint will hold up fine, hopefully. I don't know, because you never know, because the joint was fine to begin with in the first place and still ruptured. How that happens, I don't know, because I ruptured it on my own. I wasn't knocked, I just twisted and it popped. That's the thing I'm freaking out about if I push myself too hard to try and play at the level that I was playing at before, then, I tear my ACL, that's another year of recovery, plus surgery, plus the mental toll that it will take on me knowing that everyone else has progressed. I'm left behind. I'm 24 years old. 23, or 24 almost, meant to be playing sport, meant to be paying fit, healthy, but I keep doing this to myself, because I keep pushing myself. It's like that fear of risk-reward. I'm scared of tearing my ACL because I want to be able to enjoy as much as I can, where enjoying myself is by pushing myself in elite sport.

Interviewer: The mental toll and the risk-reward, do you want to tell me a little bit more about those terms?

P16: For the mental toll, at least, the last time I tore my ACL, my demeanor changed over the course of a month. Sorry. [coughs] Last time, my demeanor changed over the course of a

month. Not over the course of a month, over the course of a period of time. I'm not sure that I would want to go through that again that whole ordeal of just going on that journey pretty much of-- Because when you tear your ACL, you've got yourself to deal with and you've got to push yourself through it all. There's no one else that's going to do your work for you. That's the mental toll of doing it all alone, by yourself again, for the second time. You think, "First time, unlucky. Second time, what are you doing?" [laughs]

Interviewer: How would you say your mental health was throughout this injury, in your experience?

P16: Pretty up and down, like incredibly up and down. There would be times when it's great, doing things, progressions, but all those progressions would be short-lived by either setbacks of time and things like that. If you do one thing, great, you can start running again, cool. That just means more work and more hardship. No one else is going to push you to do that. The only way that you're going to get stronger and better is if you do the work. No one else is going to push you to do your work.

At the end of the day, your recovery is solely based on you. That depends on if you want to recover really quickly and get back into sport as quick as you can because all your friends are playing sport, but you keep getting pushed back because either you don't know why because your body is not healing fast enough, or something like that. It feels like it's out of your control.

Interviewer: How would you describe that feeling of being out of your control?

P16: Not very good because I like to have control over certain aspects of my life. There is a saying that like, "Control the controllables." It's bullshit because there's only-- Yes, control the controllables, cool, but doesn't stop shit things from happening to you. [laughs]

Interviewer: Presently, in the present, as you're thinking now, can the function of your knee improve?

P16: Yes, it should be able too, in theory. Whether it will, time will tell because as everything with ACL is time, time, time. At the moment, it's been 20 months. 18 months, or something like that. 20 months. I'm still getting swelling that was comparable to me at seven months. Either I've had a pretty poor surgery, or I'm pushing myself, I don't know. I don't know if my knee will ever get back to the way it was. To be honest, I don't think it ever will. Whether that will hinder me from playing sport and going back into the type of competition that I was in before.

Yes, it will probably affect me, but you've heard of other people coming through from ACL surgeries and returning to pretty elite levels of sport, and going further and beyond to the point where they don't feel it anymore. I am hoping for that. If I'm being realistic in that I don't have time to be an elite athlete and train six hours a day type thing anymore. I work full time. It's just not viable. If I had more time and I was at uni still, yes, maybe. Working a desk job eight hours a day is not really conducive to recovery unless you really push yourself outside of work.

Interviewer: It sounds like your confidence isn't through the moon right now, for want of a better word, how fearful are you right now in regards to your ACL injury, at this point in time?

P16: Afraid of rupturing?

Interviewer: Just in general.

P16: I'd say I have 50% confidence in it because even with all of the work that I've put in and the time, it's one of those things where until I am able to do something and my knee doesn't swell, then, I'll be happy. I guess I've got to use it more to be more confident with it. It's a catch-22 of, how hard do I want to throw myself back in just because I feel really able. It's been 20 months.

In terms of fearfulness, it's one of those things where it's risk-reward of how much do you want to get in versus how much do you want to get out? Just that, how much fear are you willing to put up with? How hard are you willing to push yourself? Not really super confident. The thing is, I'm stupid because I want to play, and I'll put myself through a little bit before. There's no way to tell how much is too much, so you don't know. You don't know. At least I don't. [laughs]

Interviewer: This lack of knowing, is it distressing to you at all?

P16: Yes, definitely, because in all intents and fairness, it should hold up. In the back of your mind, I did it once before just by twisting and going too hard. The circumstances were different. I was very dehydrated and hungover. My body was not in a state where it should have been playing. However, the reason I tore my ACL was because of that, and I pushed myself too hard. Who’s to say when I'm playing again. I won't push myself too hard, overdo it, and then tear a knee, or tear another ligament. More so my knee, because that feels the weakest.

Interviewer: What would the tearing of the knee again mean to you at this point in time?

P16: It'd be devastating. [chuckles] It would be crushing. I spent a year and a half rebuilding my legs from scratch. I was a pretty skinny guy, to begin with. I had some muscle mass. Then, I had no muscle mass whatsoever. Because I had a hamstring graft, with my ACL. My

hamstring has been affected severely, I would say. It considerably weaker and affects my mobility. It doesn't take the same range of motion anymore. If I did it again, I think I'd just stop playing sport altogether. I'd stop playing sport at a competitive level. It'd only be a social thing, or something like that.

I'd get the surgery re-done, but in terms of heading back to sport, I would be not interested, I don't think, because one surgery, I can put up with. It's unlucky. I'm still young, that's fine. Another surgery, it's another two years plus. That would kill me. It would be pretty devastating. I'd go to some pretty shit places in terms of mental headspace wise. That's pretty much the main reason of why I don't want to tear my ACL again and why I can't afford to.

That's why probably there's so much fear behind it because I place so much value on not tearing my ACL again because I don't want to go through anything like what I've been through before. Even though I probably would be well-equipped to deal with it, it doesn't make it any better or worse. It makes it worse, probably, but doesn't make it any better.

Interviewer: It sounds like you've got a very hard decision, going on between the benefits of playing--

P16: Yes, I'm in the middle of tryouts, essentially, for the club that I play for. I play division one, which would force me to push myself, or I could play with a division two team and just coast through that and strengthen myself up. On the one hand, it's wasting time. On the one hand, it's playing competitive Ultimate, which I like. On the other hand, it'll be playing a bit more still competitive, but not at the same level, not in terms of the physical requirement of the physical toll on my body.

Interviewer: It's tough.

P16: I guess that comes with non-confidence and not knowing how my body pulls up after certain sessions, because obviously if I play in the higher category, I'd want to push myself harder. Whether that's better for my knee, or for worse, I don't know. Obviously, easing into it, it's probably better after coming back into the sport. Coming back into changing directions, et cetera, things like that. A bit of a crossroad. [chuckles]

Interviewer: Big, big, big crossroads. Back to that initial injury, you mentioned that you did the injury, and you tried to get yourself off and crawl off.

P16: That's just me realizing that there is pain behind me and wanting to crawl away from that. The adrenaline came in real quick. I jumped up and I hobbled away. I was like, "Yes, I think it's fine. I think I just dislocated my kneecap." [chuckles]

Interviewer: How was the levels of fear, or distress, at that initial time?

P16: Huge. I was in the States by myself pretty much because my teammates were still playing. I called an Uber to get myself x-rayed hobbled in without crutches. [laughs] No one came with me. I went got an x-ray by myself. I can't remember the name for the type of doctor, could be orthopaedic but it's probably not, He did an ACL test where you pull the knee, and it's like, "Oh, I don't know. It probably just the meniscus."

I was still hopeful, but yes, it was pretty scary at the time. At the time, I didn't know that I'd done my ACL. It was in the back of my mind of, "It's just the meniscus, it's just something like four months. It's not the whole ACL recovery." When I found out that I had an ACL, like a fully ruptured ACL back home, I was dead. I was crying for two, three days of, "What the hell am I going to do? How do I fix this? What do I need to do?" I had a lot of support from people that have previously torn their ACL. They had told me, "Do this, do this. It's going to be shit."

Interviewer: You were upset for days, where did your knowledge or impression of the severity of an ACL injury, where did that come from?

P16: Hadn't really thought about soft tissue injuries because I never had one, or a ligament injury. I never really had one. I never really thought about it. I started playing competitive sport in university time, so that's probably two, three years. In saying that, I never really had an idea. I knew It was just out for a year or something like that. You're going to get surgery, you're just out. The feeling and the inability, knowing that you're going to lose all of your strength really quickly, and you're not going to regain your strength for a considerable amount of time is pretty scary.

When someone says, "You've fully torn a ligament," when you were not expecting to have torn a ligament at all, it was a big shock as well, too. I had my expectations set on four months recovery. No, it was a year plus. Even now, two months from two years out of tearing my ACL, you don't think that it's going to take you two years. Everyone's back after a year, right? Granted, COVID has stopped us from playing, but--

Interviewer: You mentioned that you had a good support group. How were you managing your fear and distress at that initial time, and then throughout the ACL journey?

P16: Because there was a period of time where I didn't have surgery, so it was about a two-month period, I was gathering information from people in the Ultimate Frisbee community who had torn their ACLs before, and just leaning on them about what to expect. I think the not knowing what to expect as part of the process and what the protocol is and how to go through it the best way is probably really, really hard, and really important. Finding out how to go through it the best way is something that gave me a lot of peace, or some peace.

If I didn't have a support group of people that had torn their ACLs before, and in all seriousness, I don't think my recovery was that bad, I think it was just not to my expectations. Which some are set by surgeons who have got this as a timeline, this as a sheet, or whatever, of 12 months, but they'll always say, even a lot of orthopedic surgeons, I got three opinions, they said, "You're looking at more than 12 months. If you want to return to sport long term, you're looking for more than 12 months." All these literature in sheets that had 12 months in front of them, they're just big fat lies anyway.

Interviewer: <u>I think you already touched on it, but did any health professionals, were asking about the mental aspect of the injury? Did anyone ask?</u>

P16: No. Not really. A lot of the orthopedic surgeons, you go to them, and they'd say, "Surgery. Let's go." Book you in, you're in the next week, or you're in two weeks from now. "When can you meet next for surgery? You ruptured it? Yes, we'll have a look. We'll just put you in." Then even after, in the post-op consultations, it's, "Yes, travelling well. I've done a shit hot job. Nice, good stuff. Pat on the back for me. Good job."

Even physios, they'll just say, "Oh, why are you swelling? Okay, don't do that. Do this instead. Just take it easy. Take it easy." It's always, "Take it easy." You don't want to tell your allied health professional anyway that you're sad or anything. They're physio, they're not [chuckles] your mate or something like that. They're physio, they help you physically. They're not psychologists, even though they see people that do ACLs all the time. They'll tell you practically how to get better. I mean practical in the sense of what was practical, they'll tell you what you practically have done.

They'll tell you, "This is what you need to do. If you're experiencing this, do this." Or, "If you're experiencing that, don't do that." You don't talk about them because you've got so

much limited time with them anyway because you see them once a week, or you see them once a month after that. You want to figure out, "How do I get better? Why is my knee doing this? What are the next steps that I need to do?" It's not so much, "I'm feeling really sad a lot, because I see my friends playing and I can't." Or, "I'm feeling very sad because you keep telling me three months, or you keep telling me, we'll be doing this by next time, and then you say, "We're not doing this by this time.""

It's not like you're going to accost them for that because they're not telling you what you want to hear. They're going to tell you what is the correct thing to do for your health. You have to be really lucky, and have people around you to support you. You have to cultivate those. You specifically have to cultivate those unless you've got really good mates that would come around and check in on you all the time. You have to cultivate your own support group.

Interviewer: How have you found that experience throughout this whole thing?

P16: It's difficult because you don't want to push people away because they don't understand what you're going through sometimes. They'll say, "Do you want to go here?" It's like, "I'm not able to." Then you feel like a burden. In saying that, you don't want to be a burden, in the most part, when you are recovering, which is fine, but you, at least I did, I pushed a few people away that were interested in my recovery, probably more so than I was. To me, they didn't understand what I was going through because they had never had something like this happen to them, essentially.

They've never had a reconstruction surgery where they've had to literally rebuild, re-learn, do all the things that you've done, run, jump, twist, things that you take for granted when you have functioning legs, and re-learn correct ways how to do them or physically understand

what your body's doing. For someone that didn't grow up playing sport that much, while all they did was run in a straight line and play downball at lunch, I don't know.

Some people would be more invested in your journey than you are. They want to know how you're going, this or the other. Then, they'll compare you to other people that they've known. They'll say, "Oh, but he did this in X amount of time." It's tough. You don't understand anything about my specific journey. You can understand what other people are going through, that's cool, but please stop telling you that it's going to get better because you don't know that and I don't know that. [chuckles] The worst part is someone's saying, "You'll be back." It's like, "You'll be back stronger than ever." It's like, "Shut up." [laughs] Its really patronizing.

Interviewer: These people were obviously very close to you. What kind of effect has this injury had on your relationships with them, with these people--

P16: It made me definitely more anxious and a bit more, I'd say anxious is the right word because I have developed some trust issues, I don't really trust people as much about what they're saying. I'm very attentive to what they're saying and what they might be thinking because what they might be saying might necessarily not what be what they're thinking. It's hard for me to believe that most of the things that they're saying are quite genuine because it's mostly small talk. I guess that's a trust issue that I've developed with other people from my ACL. At least I think it's from the toll that I've had during my recovery, at the least. Just people saying one thing, but then not really meaning or not following up through with it. These are people that are close to me, or they're not close to me. It's just about, for me, realizing what your motive is for talking to me. That's the lens that I stare through post-ACL. It's giving me a bit of trust issues with people with that, that they wouldn't really say genuine things like that. You can kind of tell if he's talking shit or not. I guess that's a whole separate

discussion, but it's probably part of that demeanor change that people close to me were telling me about, about being a little bit more guarded, at least.

Interviewer: Are you presently doing anything at the moment to deal with some of these anxieties, and psychological, like, the fears and the worries and the stress of these psychological aspects, to return to sport?

P16: Not at all. I'm far too busy with work and study. I am far too busy. It's one of those things where it's just it is what it is.

Time: 47 minutes 28 seconds
