# Supplementary material for: Understanding Fear after an Anterior Cruciate Ligament Injury: A Qualitative Thematic Analysis Using the Common-Sense Model
Source: Int J Environ Res Public Health. 2023 Feb 7;20(4):2920. doi: 10.3390/ijerph20042920 (PMC9957354; doi:10.3390/ijerph20042920)
Supplement: Supplementary file 1 [file ijerph-20-02920-s001.zip › File S1-P17 Transcript.pdf]

P17 Transcription

Interviewer: I will introduce the interview. Today we'll be participating in a study that is research in fear post ACL injury. The interview will consist of a series of open-ended questions that will explore your fear and levels of distress and confidence after injury.

Please feel free to say as much or as little as you want on any topic, and if you feel uncomfortable at any time, just let me know we'll move on. Sound okay? All right, tell me a bit about your ACL injury.

P17: I did it jumping a fence at school. It was not a netball injury like most people would assume. I just landed badly, then had the probably about six months to a year after. I think because I was relatively young at the time, that wasn't a full tear. I was hopeful it would all heal in of itself, but it didn't. It's interesting that you say that 45% never return to that previous level that-- whatever sport they were doing. I didn't actually play netball, so I could've never been a netball injury, but I played a lot of tennis. Probably, at the time, I was playing about seven days a week, and the knee was probably one factor of not going back. It was also getting to that pressure time at school, and then there is school travel and all of that stuff, which would have interrupted that anyway. Definitely, the fact that I still don't feel comfortable playing tennis because of all the side to side movement. I'd go and have a hit, but if someone wants to have a bit more of a proper game, I'd probably tap out even now.

That's about the injury. Oh, stop me if I ramble.

Interviewer: No, you're all right. So, you said it feels uncomfortable. What does-- What do you mean by "uncomfortable"?

P17: I just don't feel confident. I guess the context of the post-- Immediately after my surgery, I had some sort of reaction to something. Basically, my body convulsed for several hours, so the repaired ligament really stretched. While I've had the ACL surgery, and I've been through the 12 months of rehab and got a lot of function back, it's still a very wobbly knee. Anything that involves changing direction, that powerful change in direction is something where it just makes me feel nervous because I know it's not a very stable knee.

Interviewer: So you've said that twice now; that it's not stable. Where does that message-- Who's giving you that message? Where have you come up with that wobbly knee or unstable knee?

P17: Well, basically that it's not very stable. Experience, I guess, where it feels like it's kind of a bad knee. It feels like it pops in and out, but also, every time I go to see a different physio, I'll say something like that, and then they'll test my knee, and they'll be like, "Oh, yes. That's really not doing very much at all there, is it?" It's usually like that initial one where they're like "Oh, yes, whatever, sure," and then they'll do that thing they do where they try to pull your knee in different directions and it's not doing a lot, so I'm really reliant on muscle strength, I guess, to keep the knee stable. After that, how confident I am in my knee probably varies depending on my general fitness at the time and how much energy I've been able to spend keeping it strong.

Interviewer: How would you describe your confidence then? What do you mean by "confidence"?

P17: Just feeling like I can do stuff, and feeling like my knee isn't going to do that thing where it pops in and out. Not feeling like I have to second-guess what I'm doing, and whether or not it might-- Except, it's kind of ridiculous because, probably, when my knee does usually do that thing where it gives way, it's often at a really innocuous time. This has always been the case; I'll just stretch in a funny way, or I'll get up off the couch, and it will give way.

It's not always necessarily always happening because I'm doing something that involves kind of a more complex or side to side movement. That's interesting, actually, because that's where the nervousness tends to come in. I don't get nervous every time I get up off the couch. [coughing]

Interviewer: The nervousness, how would you-- What do you mean by "nervousness"? How would you describe the feelings that you have around nervousness?

P17: I think the best way to-- Sorry, my daughter's calling in the background. The best way to describe it is just, without second-guessing, it's just like a little voice. It's saying, "Should I do this? Is this a good idea?" It's not something that's stressful, but it is something that probably makes me stop and think about what I'm doing.

Interviewer: How would you describe that word? The non-stress but. Is there any other way to describe these feelings that you get, or is nervousness the best word for it?

P17: Nervousness feels like the best word for me.

[child in background] Sorry, what's happening? You can grab the iPad if you want to.

Sorry.

Interviewer: How do you feel about this lack of confidence in the knee? What kind of effect, or what are your thoughts and feelings around the effect that's had on you?

P17: Sometimes, it's a bit annoying—

(talking to child) I'm talking now. You can listen to an audiobook, or you can watch a show.

[child in background] Okay, your iPad is over there.

Sorry, sorry, could you repeat that question? Sorry about that.

Interviewer: How have you been feeling? What're the effects of the lack of confidence you've just described when your knee innocuously giving way? What kind of effect has this had on you?

P17: I mean, it's annoying that it's something that I think about. Sometimes, I get-- It's quite frustrating to feel like I can't do everything I would like to do, but then it's also-- It's interesting because sometimes there's a bit of, probably, self-blame or something in there too because I know that the more I've been exercising or sticking to things that I know strengthen and stabilize it, the less I worry about that.

The times when it's most frustrating is because life gets in the way when it all-- Like we all aren't elite athletes. You can't spend lots of time doing rehab and keeping fit. We have to fit that in around kids, and jobs, and life. Then you get into that self-blame, and it's like, "Oh, well, it's doing this because you haven't been doing whatever."

Interviewer: Do you think there's anything else that you could do that would improve your confidence or reduce that annoyance, currently, to improve the knee?

P17: Yes. Again, this is a complicated subject because I am currently rehabbing from a different knee surgery on the same knee. Yes, I think what improves my confidence is actually during the exercises that I've been given. There's definitely a thing for me around weight; not that I have ever been particularly overweight, but within, probably, a range of 10 kilos when I'm at the bottom range of that, everything feels better, which makes perfect sense because there's less impact on the knee. I'd say that's something that is kind of helpful for having a bit of confidence because it tends to feel better and feel stronger when I'm at the lighter end of that range.

Interviewer: Yes, so do you think in the future your knee function will improve?

P17: Sorry?

Interviewer: Do you think in the future that your knee function will improve?

P17: Yes, I think it could. [chuckles] Yes, I mean it's something that I'm paying a lot more attention to it in my mind because I've just had this other surgery. While I'm, probably, currently in more pain than I have been in for much of the last 15 or so years. Most of that's probably about I've just had surgery on a different problem with that knee, but because I've been focusing on keeping that strong, it is feeling quite good, despite the increased pain. I am pretty active at the moment. Not at the same levels yet that I was before I did this injury, but I think I'll get back there. Yes, I do think it will improve.

Interviewer: You also mentioned in the questionnaire that you filled out that you had some fear associated with the injury. Do you want to talk a little bit about what the fear means to you in relation to your knee?

P17: The fear means to me in relation to my knee. I don't really know how to answer that. There are specific fears, like I am afraid of-- Actually, I'm realizing, I'm not that afraid of doing an ACL because I'm almost functioning without a functioning ACL at the moment. I am afraid of doing something that means that I have to go through that level of rehab again because it sucks, as you've known. You would know. [chuckles]

I guess my experiences is not having had a perfect outcome and also not knowing what caused the reaction after that initial surgery. It's like I might end up going through all of that again at a more complicated life stage with a child to look after and for not necessarily any improved function. I do have a fear of doing damage such that I would need further reconstructive surgery.

Interviewer: What would the further reconstructive surgery, or another rehab, mean to you? How would you feel about that, for your life?

P17: It would be wildly inconvenient. It would also place a lot of stress on other people around me. I think back to that initial rehab and being young. I was on a gap year, I think, at the time that I had the surgery, so I had no responsibilities. After the first month to six weeks, I had a part-time job, and later in the year, I went traveling, but really, it was just all about me. That was easy.

I know it was still probably a burden on my parents, in them having to make sure they could ferry me around to appointments before I could walk myself there because I didn’t have a license yet wither. Not being able to care for my daughter as much as I normally would, going through another rehab, and just the stress that would put on her and my partner, and probably family that helped through that process. Juggling all of that with a grown-up job. [laughs] Sort of harder.

I have a fairly good pain threshold, I think, but it's tiring to be living with that pain that you do experience after the big surgery, and that pain lasts in a decent amount of time. I don't like taking painkillers. I never take the strong ones. It's how I am. It's because I end up with other side effects that feel worse than the actual pain and just dealing with the pain. There is a fear of that, of just living with pain.

Interviewer: The surgery for you was quite-- How would you describe the surgery? In your life, what was the effect of having to do that?

P17: I guess, similar to what I just said. It probably came at a good time in my life where there were few responsibilities. It had a big impact for a month to six weeks, whilst you're really focusing so much on just getting that basic function back but outside of that physical part.

Interviewer: Just those six weeks?

P17: Yes.

Interviewer: The actual surgery itself, did you have any issues going through that process, or how is your psychological state after the surgery?

P17: Going into the surgery, I wasn't stressed about it at all. It was interesting that a friend of mine had been through it about six months before, which is interesting two young women in their teens and two ACL injuries. Pretty unlikely, neither of us really, athletes. That helped, I guess, having heard from a peer about what that was like.

For the actual knee itself, the surgery was fine. It was the other impacts of the surgery, like having that reaction post-surgery. I think it's quite common for young people to have a pretty strong reaction to the anesthesia. I do remember just vomiting non-stop for a day, and that's

interesting because that's created fear in me for further surgery, because that was really awful. It wasn't really about the knee.

Interviewer: At that initial time, you mentioned that you were maybe that fearful after you initially injured it. Were you distressed at all?

P17: After the initial injury?

Interviewer: After the initial injury, yes.

P17: I was pretty distressed when I found out that I would need surgery because it just seemed like a huge, huge barrier to finishing Year 12, to doing the gap-year thing, and backpacking. It felt like like it was going to completely disrupt everything I wanted to do, and I think that was just-- Initially when I found out that I would need surgery, my friend hadn't had it, it really felt like a big thing for me at the time because it was all new and a bit scary. Once I got to the actual surgery itself, by that stage, I think I was just feeling a bit more pragmatic about it like, "Let's just get this done."

Interviewer: <u>Did any healthcare professionals ask you about your psychological health at any stage in the initial injury?</u>

P17: No. I definitely don't remember that at all. I do remember leaving the surgeon's office and just getting into the car with my dad and just bursting into tears. My parents were a good support around that, and as I said, my friend, who was experiencing a similar thing. Peer support is-- Sorry, this is my mental health brain coming through. Peer support's an incredible tool and hearing about the experiences of others, but I don't think that a health professional at any stage checked in about how I was feeling about it.

Interviewer: That initial time, you found out, you burst into tears, why? What kind of thoughts and emotions were you having at that time?

P17: What I was saying before about what does it mean? What impact is this going to have on-- This was all happening at a stressful period of my life anyway, being that last year of school, which in retrospect, there were so many stresses going on in that year, so it was just an extra one that probably wasn't needed. Just a bit of fear of the unknown. This is something that's probably lasted with me because of the effects that it had, but having a general anesthetic, that was quite a big thing for me. I'd never had one before because I'd never had to have surgery before. I haven't had appendicitis or anything.

That's an interesting thing for me, thinking about someone's personality traits and how-- Looking back and, I guess, having had 15 years or so to reflect on my personality traits, having some control over process is really important to me. Here's my knee. It's busted. I've got no control over that. There's nothing I can do via physio to fix that. To fix that, someone's going to have to give me a general anesthetic and that's obviously-- It's a fairy helpless situation. I think there was probably some of that going on. That's not stuff I would have been able to articulate at the time, but I suspect that was a lot of what was behind it, of how I was feeling.

Interviewer: How did you manage the helplessness and these feelings at the time?

P17: Suck it up. [laughs] I think again because I wouldn't have been able to articulate that-- Looking back, I can see that's what was behind a lot of that initial distress. In the absence of having, as you say, health professionals asking or even giving information, it probably was a lot of leaning on my friend and that advice from someone who had experienced the surgery.

Interviewer: You've had a really interesting journey as well, obviously, with your rehabilitation. When you began to get back to walking and all that stuff at three to four months maybe, did anyone ask you, through your rehab process, about any psychological aspects of the ACL injury?

P17: No, not that I recall, certainly not the surgeon. My physio was probably quite good technically but not really someone that I related to interpersonally. That just wouldn't have been the kind of discussion that we would have had there.

Interviewer: How did you address your fear? I think you've mentioned a few things, but how did you address your psychological health throughout your rehab journey?

P17: I don't think I probably consciously did. This is really interesting. I think to the extent that I did, it's about that kind of talk about the strategy that helps me to feel more confident and less nervous about what I do, is the physical side of it. It's not thinking about that. Instead of talk about that, what's going on in my head when I think, "This might happen if I do this." It's not something that I've consciously tried to do ever, addressing those thoughts and whether they're helpful or unhelpful or true or not true.

It's very interesting. I would do those for other thoughts that I would identify as being unhelpful thought patterns, but it's not something I've thought of applying to my thoughts about my knee.

Interviewer: Even now or during that process, do you believe you did have, in your words, unhelpful thoughts or fear and distress specifically?

P17: Not so much distress. I think fear, yes. I feel like at the moment because I'm rehabbing from a further surgery, some of those thoughts that I have at the moment are realistic. They're

about where I am in my current rehab. Sometimes, that's about thinking, "I feel like I can push something further today. The last time I did that, then I really couldn't walk the next day. Let's try and dial it back." I'm almost trying to call myself back this time and not getting too excited by the first time I can run. That's something that I love doing, so it's really tempting to just go for it.

I've lost my train of thought, but I do think at earlier stages, I probably have had unhelpful thoughts that-- It's not necessarily that I can't do something.

Interviewer: Just towards the end of the rehab, maybe the 12-month mark, when you were expected to maybe go back to more things or jumping or normality, did anyone ask you then? Did any health care professionals ask you about psychological stuff then or later on since, at all?

P17: Probably not at all.

Interviewer: Not at all? Okay.

P17: Certainly not in that 12-month stage because I think by that stage I wasn't seeing anyone, so there wouldn't have been an opportunity for anyone to enquire. Probably the only time that's happened has been with acquaintances who aren't treating me but who are health professionals. My partner works in a sporting club environment. We ended up spending a lot of time within that environment this year and so the physio, for example, would engage around my injury, I felt like there is a recognition of the psychological impact.

That's probably not surprising because he's working in a professional sporting environment where that psychological impact of injury is something that they have to address with the people they are working with. That is interesting because I wonder if that kind of reflection is

more likely in that professional sporting environment than if you're working with someone who's definitely not going to be any kind of even serious, hobby-level athlete. Whether there's an attitude thing there for health professionals, it's not front of mind for them because I guess the impact on people is-- It's not their life. It's not their living.

Interviewer: True.

P17: Sorry, I'm talking a lot because I'm thinking as I talk and these are quite interesting things, I think.

Interviewer: That's all right.

Interviewer: You've had the injury. Maybe at that 12-month mark, did you have, do you think, fear or distress or even a bit further on before this latest knee injury? How were you feeling about your knee?

P17: Look, I think some level of fear has been constant ever since that initial injury. Maybe's there's probably one time, probably about six years ago, so before I was pregnant with my daughter, probably for about a year before then, I was the fittest I've ever been. Everything felt strong and I felt quite confident. There was probably a really short phase where—you know exercise is a huge part of my daily life and I just felt that all the muscles in my legs were really strong and holding that knee together pretty well. I'd say that's probably the only period since the injury where I've felt completely free of fear or off that little voice going, "Should you do this? Is there a different way you should do this?"

Interviewer: How would you define that fear or describe that fear? What does it mean to you, that word?

P17: What does the word mean? It's a bit limiting. I think it can be something that at times has held me back from doing things or playing in different ways with my daughter and -- her friends. Limiting is probably the best way to describe it.

Interviewer: What kind of effect has this injury had on your overall wellbeing?

P17: It depends very much on how it's feeling at the time. If it's not feeling stable enough-- This is something that I've literally just realized after this winter where-- I injured it in probably May and wasn't able to have surgery until October just because of this year. Where it's impacting on my ability to exercise in the way that I like. I couldn't run all winter and just now that I'm getting back to that, I'm realizing that actually had a huge impact on my wellbeing. That's a really useful strategy for me, even if it's really short, a daily run. If it's good enough to do that then I would say that its impact is really minimal. If it's not-- I've talked about how it feels like it clicks out and in. It's going through one of those stages where running just feels really risky, like if I land badly on it, that's going to do some actual damage, then I'd say a moderate impact on wellbeing.

Interviewer: Your wellbeing throughout your ACL injury journey is to do with the ability to exercise, particularly?

P17: Yes.

Interviewer: Would you say that was limited at the start? Obviously, looking back it's a bit hard, but did you have, do you think, the same effect on-going through your knee ACL injury experience specifically?

P17: Probably not because in my early twenties, I was not a runner. In my early twenties, I was not really wanting to do much that my knee held me back from doing. I have no interest in skiing and I'm not really drawn to a game of social netball.

Interviewer: Would that be more of an effect if you were to re-injure, say? what would be the effect of ongoing issues?

P17: If I were to re-injure, I think the impact would be more because I-- Any impact on my ability to exercise would impact me more now because it's a more important part of my life than it was at the time of the initial injury.

Interviewer: Do you think your perception of yourself has changed because of this knee injury? Your ACL injury?

P17: A tiny bit and only to the extent that it has impacted my willingness to do some of those other kinds of sporting activities. I would say only a tiny bit because even though at the stage of my life when I injured it, I was playing tennis seven times a week and swimming four times a week. It's ridiculous. I was really active. That's just stuff I did that's a part of my innate self-image or self-worth.

Interviewer: Do you think other people's perception, then, of you changed because of the injury if maybe not your own?

P17: I don't think so, particularly. Actually, possibly in a positive way because I feel like I've probably done a fair bit of whinging in this interview, but it's not something that I tend to do in life. I guess even over this winter, the context is we ended up in an AFL hub for all of winter, so that was an unusual part of it.

I had a number of people who I really don't know very well commenting on-- Sorry, I know this is a later injury, but it's probably similar to how I managed the initial one, that they thought it was something that I'd dealt with quite well and hadn't complained and just kept on an managed things. I think to that extent-- so possibly a positive impact.

Time: 37 minutes 25 seconds
