# Supplementary material for: Understanding Fear after an Anterior Cruciate Ligament Injury: A Qualitative Thematic Analysis Using the Common-Sense Model
Source: Int J Environ Res Public Health. 2023 Feb 7;20(4):2920. doi: 10.3390/ijerph20042920 (PMC9957354; doi:10.3390/ijerph20042920)
Supplement: Supplementary file 1 [file ijerph-20-02920-s001.zip › File S1-P18 Transcript.pdf]

P18 Transcript

Interviewer: Awesome, thanks. Today you'll be participating in a study that is researching fear post ACL injury. The interview will consist of a series of open-ended questions that will explore your fear, levels of distress, and your confidence after your injury. Please feel free to say as much or as little as you want on any topic, and if you feel uncomfortable at any time let me know and we will move on. Does that sound okay?

P18: Yes.

Interviewer: All right. Just tell me about your ACL injury.

P18: This was in May 2019. I actually used to play lacrosse a lot when I was younger and then stopped playing when I turned about 16 or so. Then when I was about 22 I decided to get back into it. I played a whole season of lacrosse and then just halfway through my second season back, and then I think it was just mid-game, running towards a goal, had the ball. I didn't think I tripped on anything, I don't think anyone tripped me or anything like that, I just think that I tried to pivot and I distinctly remember hearing a noise. A snappy kind of noise and now I just completely collapsed.

I remember I got carried off, sitting on a bench row with ice and then my mom took me to the hospital. This is where it all went wrong, because I wasn't there completely sporty. I wasn't super sporty for quite a long time and I'd come back into it. I'd heard of an ACL but I didn't think it wsa anything that I could have had. I went to the hospital, I told them that I heard something in my knee and I couldn't stand on it.

The hospital doctor just pretty much said that I'd maybe just strained something. I'd sat in the waiting room for hours and hours and hours, waiting for them to tell me I'd just strained something. They put a velcro cast on my knee, gave me crutches told me to rest for a bit. That happened. Later that day I tried to stand up, completely collapsed and it was excruciatingly painful.

Then it wasn't till probably-- Took a few days off work, I used to ride to work every day for about 40 minutes each way. It was a big part of my commute so I couldn't do that so I stayed at home for a few days, worked from home. Then about a month later when I was off crutches but hobbling, I hobbled to the tram. I ended up deciding that it's probably best for me to go to a physio because I couldn't really bend my knee and I was just like this doesn't seem like it's getting better.

I went to a physio, and the physio told me, "Oh, it's like you may have actually hurt your ACL." I was like, "Oh, okay," because the hospital doctor didn't say anything. Then they sent me to get an MRI, so I got an MRI and then I got sent to the physio and the physio was like, "You need to go see an orthopedic surgeon." I don't know the exact ones but there was multiple. There was the ACL and there was the ones on the side, but I don't exactly remember their names. This is about a month after it actually happened.

Then I went to see an orthopedic surgeon, and he said pretty much-- By the time I got the appointment with him, it was almost like two months after I'd done it. By the time that that had happened he had got me to get another MRI. He said that because it had been left for so long he would not recommend getting a surgery because he thought it had actually started to heal. I said to him, I was like, "Look, I still can't bend my leg properly." I'm trying to go to the physio but I can't mentally do things. I still had this limp and I couldn't ride my bike because the motion of my knees, doing the pedal I just couldn't physically get there.

Anyways, I'm meeting this guy, and then I went back I think for a second follow up with him, telling him that I can't ride my bike, I can't ride my bike. This is why I wanted to do this thing because there's almost a complete miracle and I almost think that it was in my mind that-- He said to me, I don't know what they call it, but they grab on the side of your knee, and they do a jerk pull kind of test where they're testing-- I don't know what it is. He did it and it clicked in place. He said if I can do that then you can ride a bike. He was testing to see if it was healing and whatever. He said if I'm capable of doing that with my hand without force you should be able to pedal with a bike.

Previously I'd had to get my boyfriend to hold me up on the bike, try and pedal and it's like in my mind I couldn't do it. Then as soon as he told me that the next morning I said, "I'm going to go try and ride around the block." It was like I could just do it. It was really, really strange, It was like I just completely put myself in a headspace rather than being capable of bending my knee. Then when he'd done that it gave me a little bit of confidence.

That was probably about like three months or so after I did it, then I started riding my bike again so now I ride my bike and I can jog. I could do certain exercises, but there's a lot of things I can't do. I get really worried when I go for hiking, where I'm standing. I've pinged it before. It feels like I twisted my knee the wrong way and I can feel it. When I went back to him he said to me--

Interviewer: Did you say you've "kinged" it before? What was the word?

P18: Pinged it. It feels like a little twinge like it's gone the wrong way and I can feel it twinge a bit. That was obviously like May. I did it May 2019 It was just trying to do some exercising like that. There's just certain things that I won't do anymore like my partner likes skiing and I've done it a few times with him but I would never, ever do that again.

The orthopedic surgeon said that if I wanted to go back to sport and if I did it again I would definitely have to have surgery to fix it. I feel like I'm a part of my team and would help coach them and stuff but I don't think I'd ever play again. It was so just very expensive to if I need to, and I don't want to go through that. It was too painful in the first place.

Interviewer: You mentioned the word "worried" before, you were "worried" about it. What does "worried" mean to you?

P18: This worry when I think it's like a multitude of things. It's physical worry that I will damage it again, and the pain, but there's also the worry of the cost of getting surgery, the ordeal of getting all that pretty much put me back to where I was in May. I'd like to obviously changed how I use things. I'm very cautious of where I step.

Even if I step off a curb I still have it in the back of my mind that I should try and land on the other foot because about a few, about three weeks after I did it, I was taking my dog to the park to walk. There was a little low metal rail around the oval and I stepped over it. Just trying to get over. It was quite low but I stepped with my bad knee and I just completely buckled and collapsed, it was so painful. The thought of that happening again is--

Interviewer: You've just mentioned bad knee. What's your thoughts and feelings? Is there a difference in your knees and how you perceive them now, how you feel about them?

P18: Yes. Definitely. I can still tell because I didn't know for so long what had happened. I was using my right knee which is the good knee a lot to do things. If I just stood up from a chair I would put more weight. The physio was telling me that I was over building a muscle on my right side because I didn't want to put pressure on my left so I was making the other one stronger.

In my mind now I still think physically the difference between the two and I know it's the weak one. If I do an exercise where it's some kind of squat where I have to balance on one leg I'm always completely happy to do it on one side but I always like to think a little bit before I do it on my other side.

Interviewer: Okay. You also mentioned the ordeal, you wouldn't want to go through the ordeal of it again. What does that mean and what would a re-injury mean to you?

P18: To me, I consider myself not crazy sporty but I'm active. To me walking around, getting around on my bike are really important. I try and avoid getting public transport in general because I was trying to save money. Obviously just the pain but the feeling like I was incapable of moving like I wanted to move, just around, so I was spending a lot of money on Ubers too and all of that kind of stuff because I was just really worried that if I had to walk anywhere or kind of thing. Obviously, the physical ordeal of the pain and the recovery and doing all of that stuff, but it was also just movement, like going and having a shower was so difficult the first few weeks.

Interviewer: How did that make you feel, the difficulty of the ordeal you experienced, the having the issue about the shower and things?

P18: I think it was it made me feel a bit useless and I said to the physio when I did it, I was like, not like is it my fault, but I was like, "Was it because I actually wasn't-- the muscles in my leg aren't strong enough or was it--" Try to pin it on something or, "Was it because--?" She told me it's a really unlucky thing.

I think that made me feel a little bit better because originally I felt like a bit of a dickhead, "Did I do this because I tried too hard doing something that I was not physically capable of doing?" It did made me feel better when she said it was just completely unlucky, but now

even when-- My boyfriend loves football, and when we watch football and someone injures themself, I can't even look at it. I was like, "Ugh."

Interviewer: How does make you feel and what are the thoughts and feelings when you look at someone? What's happening there?

P18: I actually think I feel-- Obviously I watched football for years and whatever, but I never really had a thought for professional athletes that injure themselves like that, but now I just feel unbelievably terrible for them because I know how it impacted my life, but I don't make money from playing lacrosse or whatever. It just it actually makes me feel really, really, really sad for them. Yes, just the thought of in my mind-- I guess they've probably got more medical people around them, but in my mind, it's almost like it's very difficult to recover physically and be 100%. When I see a young football player do it, I'm like, "Your career is ruined in a way."

Interviewer: Do you get any when you say that, does that relate or do you relate to that quite a lot through your own experience?

P18: Not that I relate to the future aspect where I feel like my future is impacted, not obviously financially, but just I think I will always be cautious, so that's in a way. I used to make a joke to my friends that I started jogging again during lockdown. I said to my friends, I was like, "I don't necessarily want to be an athlete again, but I want to, if I had to run away from a serial killer or something stupid like that where I have to run to save myself, I want to be able to do that." I want basic things even if it's stupid. I still think in the future that I'll also be worried about it and also it still would impact me.

Interviewer: Do you think your knee function will improve into the future or can improve?

P18: I'm into the point where actually probably should go to the physio again, but I feel that I'm at a point where I'm probably doing the most activity right now that I've done obviously since I did it. Part of my mind thinks that it won't get better and it's just I can hike, I can ride a bike, I can jog, I can squat, but still you can hear a noise every now and then or you feel a little thing. I personally don't think it will get any better unless I maybe went to a physio and tried to really focus on it which I don't think I will.

Interviewer: Now, what emotions and feelings do you get when you consider that you think it maybe won't get better?

P18: I feel like originally I was obviously really upset because I actually did, obviously just from a sports perspective, I was actually really enjoying playing sport again. I was a little bit sad at the start because I knew that that would never happen. Now, I wouldn't say that it makes me sad to think in the long run, but it just makes me think that I know I'll always be cautious and it's the lock in with things that I have to deal with. It's something that I have to always consider.

Even certain things like I sold a bunch of really tall high heels that I had because I was like I know that I'm not going to be comfortable wearing these going out, especially if I'm drinking. I just don't see myself wearing these, so I sold a whole bunch of really nice shoes. I still wouldn't do it now. It's a bit sad, but it's also just I feel like now I have to accept that it happened and just be cautious or whatever.

Interviewer: You mentioned sad twice, have you had any other psychological or other things that have occurred because of this knee injury?

P18: I think just like, I don't know if you'd consider this an emotion, but they call it FOMO, not the jealousy in a way, but jealous of I went out with my friends that I used to play

lacrosse with and they're talking about starting the season again and it's a little bit of jealousy that I can't ever do that again. Obviously, yes, I can probably do lacrosse a bit as I am now and now that I could do most things that I did beforehand besides sports, there's just things in my life that I know I won't do. I don't really like skiing anyway. There's a few things that I won't miss, but I guess jealousy a little bit.

Interviewer: Yes, okay. In that questionnaire, you indicated that you had some fear associated with your ACL injury, do you want to tell me a little bit about that?

P18: Well, I think obviously as I mentioned before I think the fear ultimately stems from doing it again and the pain of that and the cost of that and the time that will take from my life again and the lack of movement. I just don't want to be, I know it sounds like a really lame word, but I don't want to be a victim to it again, so it is fear. I run every day now or jog, but it's just there's certain ways I wouldn't walk. I wouldn't walk down a really steep hill thing. I went hiking a few weekends ago and I'm constantly looking at my feet because if I trip somewhere, it's the thought of that is scary.

Interviewer: There's a few things in there. What would being a victim mean to you from an emotional standpoint?

P18: I think a bit helpless like I considered myself like I had to get a lot of help with the crutches, with the struggling to even just bend and move my knee in the way that I needed to. If you had help and it's like to me it's quite humiliating to have to get on the tram in crutches, that sense of I want to be independent in that sense with my own movement and it's that sense of uselessness.

I also really don't like being in hospitals and you're lying there, you just feel like you're completely useless and everyone has to look after you and I don't like that.

Interviewer: You also mentioned that it was the thought was scary, so what's that when you're looking at it, what's the scariness of that re-injury you mentioned and what does that entail?

P18: I think the scare factor is the pain.

Interviewer: With the pain--

P18: Yes, I don't know.

Interviewer: What? What?

P18: I don't know if I could elaborate that anymore, I just don't like getting bloody hurt, you know?

Interviewer: Yes.

P18: You don't want to get something to happen to you that hurts.

Interviewer: Yes. Do you think you've mentioned the pain a few times, so what does the pain or what did it mean to you or what thoughts and feelings did you get because of that pain or when you're reflecting on it?

P18: When I'm reflecting on it I always tell people or I always mention it as probably the most painful thing that's ever happened to me. I remember when it happened, I just physically collapsed and was in a ball and it was almost like I, not like I passed out, but it was like I don't really remember. I remember it being really terrible and then after that subsiding a little bit, it was the really intense pain of the moment and then it fading slightly, but that shock pain when I mentioned that I'd stepped over a railing and done it again, I got the exact same thing. It's almost like an intense shock moment and it hits you like a truck.

Interviewer: At that time, how would you describe your distress at the time of that initial injury, when you stepped over the rail, or the initial climb at the end of that sport?

P18: The initial time was completely like-- You could have told me that a bullet hit me and I would've told you that-- Because as I mentioned, I was playing sport but I would not call myself a super sporty person. When it happened actually, they had no concept of what it was. Obviously now, I think my cousin-- I've got a few cousins lacrosse and they've all done it, but I obviously never talked to them about it.

To me, the first one was like a complete-- I could've been shot by a bullet, I could've been hit by a bus, I had no idea. It was almost a bit of an awe. I had no understanding. The second and third time, I did it again that night when I tried to stand up, [chuckles] because obviously, I wasn't told of what happened. I tried to stand up and just completely collapsed and that was also terrible.

Interviewer: That sounds pretty traumatic actually. Obviously, the hospital didn't pick it up, but through your initial injury and your physiotherapy-guided stuff and the surgeon, has anyone actually asked you about the psychological-- Do you have any psychological aspects of your injury?

P18: No. The only thing I really remember is just telling the orthopedic surgeon when I was seeing him the second time that I don't think in my mind that I could ride a bike. It was the first time I said anything to-- Because obviously, the only time I had ever saw anyone was the first doctor in the hospital and he didn't say anything about ACL. Then the physio there I saw a few times, she only mentioned it after she saw my MRI.

I did maybe one more session with her and I couldn't afford to keep going back every few days or a week or whatever. It was only until I mentioned to the orthopedic surgeon on that

second time around that in my mind, I don't think I could ride a bike. He then said to me, "I think that's in your head a bit," because then he did that knee thing where he tried to show me that it was strong. Him doing that was probably the one thing.

Then I ended up not seeing him again because he pretty much said to me-- He said he wanted to do a follow-up appointment, then COVID happened. Didn't really get to do that. He pretty much just told me if I'd do it again, I'd have to get surgery. I haven't really spoken to any one of those people about it besides him, besides that orthopedic surgeon briefly.

Interviewer: At that time or around that initial injury, did you do anything else to manage the psychological aspects or did anything else occur or was that mainly just that surgeon thing? What other aspects you were going to employ?

P18: Not really. As I mentioned, for the first month or so, I didn't actually know about what I've done. I had thought I had strained it really badly. For a moment, I thought I dislocated my knee because I heard the noise. For a long time, I was that. That actually made me feel worse because you read a lot of stories about people dislocating their knee and it keeps happening over their lifetime. With some, it I found out it actually doesn't get better. I think that actually felt like it stressed me more.

When I found out actually about my ACL, it was all a bit shocking. I got a bit angry at the man from the hospital because I felt like I could've avoided a whole bunch of not knowing and upset over what I thought it was if he'd actually maybe could've been...

Interviewer: Where did the shocking come from? What kind of thoughts you have over an ACL injury and what messages made it so shocking when you found out specifically it was an ACL injury?

P18: I remember actually thinking it was funny, because I found out-- I did it in May. It would've been a month or a bit after I actually found out that I've done it. That was obviously peak AFL season. In my head it's like an injury that AFL players get, professional sportspeople get those injuries, not just me who trains one day a week and plays a game on Sundays kind of thing. I thought that was a bit funny. I was a bit in awe. I can't believe that I've done something like that. I don't think I'm much of an athlete to do something like that. That was the first shock.

Interviewer: You're always impressed by yourself in a way. [chuckles]

P18: Like I said-- [audio cut]

Interviewer: Hang on. Your microphone's **[unintelligible 00:24:59]**.

P18: Sorry. I just had to **[unintelligible 00:25:01]** my dog.

Interviewer: Okay. What about at the initial phase. Well, you've touched on it, but what effects this injury had on your confidence?

P18: To me, a bit part of my confidence was-- I know it sounds a little lame, but I was always very proud of the exercise that I got from riding my bike to and from work and being very mobile. I could pretty much tell myself "If I can ride there in an hour in Melbourne, I wouldn't take the train, I'd get there on my bike." To me, being helpless a little bit at the whim of an Uber or someone driving you, it just made me feel a little bit weak.

Obviously, I didn't go do much out with my friends. I remember the first night that I actually went out somewhere, I wore the brace underneath the skirt. I had to try and walk down the steps at a bar almost a bit like a straight leg. It's pretty embarrassing.

Interviewer: How did that make you feel having to do that?

P18: I think also too because I'm a designer, I work in graphic design and stuff. Not obviously the La Cross people because it was a random, out of the usual for me, but a lot of my friends had no idea what it was. Obviously, my boyfriend knows because he's into football and stuff. It was just like no one even got it. No one even understood why I was on the crutches and why I couldn't do things. No one I knew that I really talked to closely had ever had it happen.

Interviewer: What are your thoughts and feelings around the fact that no one had known the significance of it?

P18: I think it actually made me talk about it more. I wanted people to know just because it sucked so much. [chuckles] Obviously, their lives, they've not thought of anything like that before. It obviously made me feel, not necessarily alone, but I would tell people about it because obviously, I'm not in a group of, in this circle that this kind of thing happens.

Interviewer: Fair enough. During that period when you were doing that, and also, I guess going along with your rehab stuff, maybe about that mid-rehab three or four months, did you have any fear or distress? You said helplessness and pain and you were angry and shocked. What were your emotions at that time?

P18: [inaudible 00:28:07] cutting out a bit.

Interviewer: Sorry.

P18: Sorry, I'm cutting out a bit. I may have to move around. Apologies. You cut out a bit in that last question. Sorry, do you mind repeating yourself? I'm sorry, you cut out.

Interviewer: Can you hear me?

P18: Yes.

Interviewer: At that mid-period, that mid-rehabilitation when you were mentioning you were starting to get back to normal things like going out with your friends, during that period, did you have any fear or distress or strong feelings? What were you feeling in terms of your knee at that time?

P18: I think there was a point where I got a big confidence boost from getting on my bike again. I wasn't thinking too much in the future and I was actually just very, very happy that I could ride my bike. I had one moment where I fell off my bike and actually landed on my leg and nothing happened. I think that actually just made me very happy. This was a few months out. I was very, very happy about it. I started to try to a point where it was not just riding my bike, it was like I was doing things like squats and all that kind of stuff again.

Then it came back in my head that I was like, "Oh, I actually probably do this again." There was a peak where I was actually feeling really good. [chuckles] Obviously, I don't think walking your dog is a thing, being home for six months or whatever it is and they're doing the same thing every day and trying to stay fit because they were bored out of sitting inside all the time because of COVID. I've been trying to exercise more so I think it's come back into my thought process a little bit more in the last few months.

Interviewer: In that kind of way is there anything you've taken away from the injury that's maybe a positive thing?

P18: Oh God. I think it's just made me **[unintelligible 00:30:22]**.

Interviewer: Doesn't have to be.

P18: The only thing I would say it's not even a positive was don't always trust. I got pissed off a lot with the doctor of the hospital. Then I was like I think it's made me a little bit more skeptical and wanting people to listen to you. I don't know if it's necessarily more like a woman thing. I feel that if I was a 17 year old guy who was playing footy and went into the hospital for the same injury they would have said ACL. I've learned to be a little bit more, not tell them exactly what I think but I was a little bit-- Obviously I didn't know, I was a little more timid, I think that definitely impacted how they treated me. I've definitely changed how I talk to people like that.

Interviewer: Okay. Currently as you go back to sport how fearful are you in regards to the ACL?

P18: It's the point where I wouldn't actually-- I would never play, I couldn't see this work the sport lacrosse there with sticks and whatever. I would **[inaudible 00:31:47]**.

Interviewer: It's just cutting out a little bit.

P18: There's no way I would ever play yes.

Interviewer: [unintelligible 00:32:00]. It's just cutting out slightly at the moment.

P18: Sorry. Is that better?

Interviewer: Yes. I can hear you now.

P18: I'm sorry. Is that a little bit better?

Interviewer: Yes, I can hear you now.

P18: Okay. Cool. No, no. I was just checking on Kenny. All good.

Interviewer: Awesome. Sorry so you just said you unlikely. What were you saying that not playing again?

P18: I definitely know in my mind I'm not going to play again. I would consider doing light training where I wasn't in a game situation. I don't think I'll ever put myself back in the situation where I would be playing again. Especially now that obviously I'm going to get older eventually and I feel like there's a lot of people that may go back to sports when they're in their 30s or 40s just so they get some fun. I just don't think that would ever happen because-- Or maybe if I forget. I don't know but I just don't think I'll ever do that.

Interviewer: You were faced with a fear for movement. You've just described looking at that. How would you respond beyond just the pure avoidance? How would you respond with a fear for movement now?

P18: It sounds really unhealthy when you say like that like a pure avoidance. In a way I said that I would do some things, like I would go out for a jog or I would train lightly with people I used to play with, it's not in the game situation. I would do subtle things, certain things. Like for example, I said I would never ski again but I would maybe go hiking in the snow or something. There's steps that I don't think I would ever get further than.

Interviewer: Okay. Overall how would you say right now your knees, what effects is it having on your confidence?

P18: Just constant vigilance. Obviously it's just frustrating. Being constantly vigilant. Being 25 you don't think that you should be constantly worried if you're stepping off a curb the wrong way. It takes up much more of my soul than I would want it to and I maybe probably could be less worried of it. I feel like I'm cautious enough that it does. It will impact me for at least probably the near future.

Interviewer: It's impacting you for the near future so what effect do you think it's had on your overall well-being then?

P18: It's not my physical well-being at all, it's mostly medical. Obviously as I said yes I would like to go play sport again, but I'd probably not, but that's not going to stop me from earning money. All my friends, the only friends I have play sport and I can't do that. A lot of the things I do in life I can still do. It's certain things but in the long run I just think I will always be cautious, because then I have to accept that that's it, I can't change anything.

Interviewer: Has it got you down at all or had a negative effect on your mental health do you think?

P18: As I said before I think definitely the first few months, and then I'm being positive again. In the long run mentally I think it should be fine. As of right now it's just being vigilant really. Like today I was doing some exercises and I did a squat and my knee made a funny noise, and then I had to stop what I'm doing and then I have to worry about it. Gone on me that I start things and then-- I just think that the vigilance is probably the worst impact on my leg with mental health at the moment.

Interviewer: Do you have any issues sleeping or anything where you're thinking about your knee and you don't want to?

P18: I usually dream about lots of things. I never had any things like lying in bed at night worrying. Obviously when I first did it it was painful of course you think about it, but I don't think I've lost sleep over it.

Interviewer: Has the perception then of yourself changed because of this injury?

P18: I feel a little like damaged goods. As I said before it's not like everything I'm doing everyday is revolving around just being active and having to be an athlete. I do feel that put in the situation as I mentioned as a joke running away from a serial killer, put in that situation I cannot perform to the level that I used to be able to.

Interviewer: Can you elaborate any more on that feeling of being damaged goods? Or is it just that situational stuff that makes you a bit uncomfortable now?

P18: When I went to first see the orthopedic surgeon, because there had been so long since it first happened, he told me that there was a lot of healing and whatever. It wasn't perfect. He said obviously if you get surgery they train you, make it, I don't know how they do it but make it as good as new kind of thing.

I have a feeling that when he mentioned how it was healing that maybe it wasn't healing perfectly, but it was healing so they didn't want to disrupt it. In my mind I've got the notion that it's not actually healed to the level that it would have if I had gotten surgery and that happened to be perfect. In my mind I've always got that kind of thought that it wasn't quite right when I finally figured out what it was and then I always just had to deal with that. You can't just go even get surgery, done just out of nowhere, you have to have it happen again and obviously I don't want it to happen again. In my mind it's always just going to be a little bit off.

Both my cousins I mentioned had it happen playing lacrosse, and they've both had surgeries and then done it again, and then had another surgery and are still playing. Even that actually makes me-- I had to see it from a family member. To me like you said that the surgeon said 99% but I don't know, actually there's a lot of girls that like lacrosse, they get their knees. They do it again while playing like see you later.

Interviewer: Okay. Agreed. Has the way that other people then-- I know you mentioned your teammates and the different things you had going on with the girls but has the way that other people perceive you changed because of the injury?

P18: I really think so. This sounds terrible. I think my Mum-- This sounds terrible. My Mum is very watchful of money, so if I was doing something that maybe she thought could hurt me again, she would be like, "You don't want to have to pay for a surgery and blah, blah, blah." If I were to go to say to my Mum I was going to play lacrosse again, she would be like, "I don't think that's a good idea."

I think my boyfriend would be like, "If you want to do it, you should do it." But I think my Mum because she's the one who saw it happen, she's the one who put me in the hospital, she's the one who I called when Physio told me that I had torn my ACL and I was a little bit shocked so I called her. I think most of my friends have really forgot about it because as I mentioned, all of my friends who grew up aren't sport people.

Interviewer: What does that message from your Mum mean to you?

P18: I think it probably just compounds my own cautiousness. Maybe if I had gotten it into my head that I did really want to play again and I told her and she told me, "I see that as a bad idea." I'm very close with her, I would definitely take that on board and I would maybe change my mind. Whereas the opposite is if, my boyfriend, he was like, "Oh, no. I think you should do it." Then I would probably be more confident? I guess it's that they have very different personalities.

Interviewer: I know. With the fear range, what is your biggest fear?

P18: Physically or mentally?

Interviewer: Mentally. What would it really mean to you to have a range, or--

P18: It would mean going back to square one? It was very compounded because I was in a bad job and the injury happened at the start of winter. I couldn't really move and when I needed to move, there was a long time of just walking my crutches. It felt like a kilometer to get to a tram. It was very grim. It was a very gloomy time. I was trying to be self-sufficient but it was failing. As I said before, I don't like feeling weak like that. It would just be going back to a bleak time.

Interviewer: Thanks. I love that. It was very helpful. A lot in there. [crosstalk]--

P18: [laugh].

Interviewer: No. It's good. It's really good. That was great. Great in terms of the information provided. Not great in terms of the experience.

Time: 35 minutes 15 seconds
