# Supplementary material for: Understanding Fear after an Anterior Cruciate Ligament Injury: A Qualitative Thematic Analysis Using the Common-Sense Model
Source: Int J Environ Res Public Health. 2023 Feb 7;20(4):2920. doi: 10.3390/ijerph20042920 (PMC9957354; doi:10.3390/ijerph20042920)
Supplement: Supplementary file 1 [file ijerph-20-02920-s001.zip › File S1-P2 Transcript.pdf]

**P2 Transcript**

**Interviewer:** I'll just start the things. Thanks for meeting with me, P2. Today, we will be participating in a study that is researching fear post ACL injury. The interview will consist of a series of open-ended questions that will explore your fear, your levels of distress, and your confidence after your injury. Please feel free to say as much or as little as you want on any topic, and if you feel uncomfortable at any time, let me know, and we will move on. How does that sound?

**P2:** Wonderful.

**Interviewer:** Wonderful. All right. Do you want to tell me a bit about your ACL injury?

**P2:** Yes. I've actually had it twice. I've played basketball my whole life. The first time I did it, I was over in Japan competing for the-- It was the under-23 Australian team. I got there, it was the World Winter Games. That's where we went for, which is not like the Australian University Games which is like a piss up like a big event, and it was a huge deal, we were super excited, and then the first game, we were playing in the US I went down and did my knee the first time.

It also happened to coincide with what was going to be my senior year. However, I was playing basketball in the states, so terrible timing. I went home. I ended up having surgery here, went back to the States to rehab that year in the States. Then, nine months almost to the day later, I was back in Australia, saw my surgeon here, got cleared, everything was great. I thought I'd crushed the rehab. I felt really great.

I was in this phenomenal setting where everything was there for me. I was then playing like this controlled one-on-one two weeks later, with someone who'd been taught how to play

against me in a rehab setting, so he was two feet away from me. It was more about change of direction and stuff, testing the direction. That was two weeks after I got cleared. I changed direction and did it again, same knee.

Then, that time, I decided, so I was going back for my fifth year in college, which I didn’t want to red shirt my senior year, I didn't want to do six years, so I didn't want to red shirt it again, so I decided I was going to come back quickly. I wanted a lars ligament, which was a three-month recovery, and my surgeon wouldn't let me do that, so I ended up having a-- What do they call it? Like a cadaver graft, essentially.

**Interviewer:** An allograft, yes.

**P2:** Correct. It's been a while now. Allograft. I had an allograft through my knee, and then I had a-- They pulled the ITB band, and they stretched that out over the outside of my knee. Came back again in four months, just over four months, played out the rest of my season which was 2019 That was last year. We started last year, so Jan through March, I played.

I finished my college career there and had the opportunity to come back and play pro here, and I've just never been able to grapple with the idea of playing. I've gone on to play semi-pro. I don't know how well you know the basketball leagues here, but they're the second tier, and they all want a gold. I was going to play that, but obviously, it got canceled because of corona in the end.

I was training for that, but the WNBL have just-- I haven't been able to cross that path because I always go, "Oh, what if I do it again?" It's not worth giving up my corporate career for a basketball career when the risk is too high. That's my idea trying of to understand this, and obviously, mine was twice. I tried to come back but didn't. The thought of doing it a third time has just stopped me. I can't.

**Interviewer:** What about that thought of doing it a third time is so confronting?

**P2:** I always go back to the same thing. I know when you're having kids of your own, I want to be able to run around with my kids when I have them, and I think that's the biggest thing that I always come back to. To play pro, I'd have to give up or scale back on my career right now. I have to make the decision. The risk versus reward, the risk is so high, or I see it as so high to do it again.

The thought of not being able to-- Even now, I can't sit cross-legged for a while. I can move freely, you wouldn't know that I've ever done it. I still work out all the time, I can still play basketball if you asked me to, but I just don't want that future, and I don't want to go through the shock of it at the time, and the rehab's brutal. I guess that just going through it all over again as well, that's something I want to avoid.

**Interviewer:** How do you feel about the rehab process?

**P2:** It's tough. Obviously, I did one that was very condensed. I was doing it for six to eight hours or nearly half the day for a month. Then, there's the other one, it's brutal, and it feels like you're doing the same thing over and over again for such a long period with no-- There's times when you're running in a straight line for a two or three-month plan that you find no reward. It sets you back nine months because then, you spend nine months trying to get back to just when you were, essentially.

People always say, "You're going to come back better," and you do, you're stronger and that, but still, you've lost a lot in life. That was nine months of playing time when I could have been getting better, and I was just trying to get back to where I was.

**Interviewer:** How do you feel about that thought that you haven't progressed in terms of your abilities? What are the emotions that you feel there?

**P2:** My coping mechanism was just to find another avenue, which was a job, a nine to five job. The emotions were-- It was frustrating I would say. I've grown up as someone who's always been able to go to the next level. I've been very lucky. I've worked hard, but I've always had amazing teams and done this and done that. I've never been injured until this one here.

It was traumatizing almost of just not being able to do what I want to do, but now when I play basketball, I'm not as quick as I was, I'm not as agile, and I think that's-- but I am. If you do a sprint test in comparison, I was quicker post-ACL. I was lifting heavier, I was more agile, but, on the court, I was mostly slower and less agile. I think that was just fear.

**Interviewer:** With the fear, what does the fear mean to you?

**P2:** I was thinking of this the other day. It's frustrating. Sometimes, I look at all the people like, "Oh, I can't do that anymore. I can't play basketball anymore. I can't do that anymore because of my knees," and I'm like, "I'm 25, and I have that same thought?" For me, what it means to me, it's disabling a little bit. Physically, I can do those things, but it's the mental factor thats stopping me. It's frustrating. It's things that I know I can do, but the fear is what's stopping me.

**Interviewer:** What do you think that mental thought pattern is that what is stopping you from achieving the next level?

**P2:** Mental thought pattern. It'd be like if I see someone doing something, I see it and internally know I could do it. Maybe it's more the fear that I wouldn't be doing it as well as I

was because I couldn’t. I don't know. I've never really thought about that, but that's interesting, the action of thought pattern, or it's just that I don't want to try it because it may hurt or it may not go as well as I want it to go. [chuckles]

**Interviewer:** Did you have pain as a significant problem during your experiences through either of the ACLs?

**P2:** Yes, pain and swelling were my 2 big ones

**09:36**

**Interviewer:** You mentioned that fear of not doing as well. What would it mean to you not to play as well if you were to commit, for lack of a better word?

**P2:** You see, that's the thing that, I've come to this acceptance that I actually won't ever play as well. It's not even this fear of not playing as well. I want to say realization because I could if I really put my head to it, but I've convinced myself that I never will play as well, and that's why I'm not doing it. I wouldn't know it. I haven't really tried it. Apart from that four months I played after my second rehab which I played fine, like I played well. Maybe the fear has turned into this thought process where I've convinced myself, "That avenue is gone because you'll never be as good because you don't want to do it again." It's not even whether I would play as well, it's just I don't even consider that as a prospect anymore.

**Interviewer:** What about if you were to consider it as a prospect? What do you think would be holding you back from an emotional, psychological point of view?

**P2:** The fear of doing it again. If someone said to me, "You have to play this season," it wouldn't even be the fear of not playing this well, it would be the fear of doing it again would be the main one. I could handle sitting on the bench or not being as good, but the reason I would likely say no would be because I don't want to do it again.

**Interviewer:** What would doing it again mean? How would you feel about that? What would that mean in terms of your life at this point?

**P2:** Maybe doing it again, it's like now, I know I could still play. I know if I really push myself, I could, but a third time is probably like call it a day. My surgeon always says that three, four, definitely. Three, you'd be pushing it. At least now, I know myself that if I really want to try, I could give it a shot, but doing it again, it's like, "No, you're done. That's it."

**Interviewer:** Okay. At that initial phase after either the first or second injury, did you experience fear at that point?

**P2:** No, I went through these like incredibly motivated period straight afterwards. The first time, we don't know what to expect. You don't know what the recovery is. You just think like, "Oh, I'm going to crush this. I'm going to be better than ever." I'm not a journaler, I don't write journals, but I have journals from back then where I'd be so motivated, but I couldn't do anything about it because you would be two days post-op. I'd write it all down. I look back at them, I'm like, "My God, I had massive goals."

Even the second time, knowing what to expect and knowing what I'd done, the crappy position that put me in. I still found that-- I was just super motivated I'll get over it if I could do it the first time, I could do it the second time. The fear didn't really creep in until I actually started contact again. That's when the fear crept in because the chances of me redoing were so

slim at the start. It wasn't fear then, it was fear when I could physically-- The thought of redoing it was a real thought and was a real possibility.

**Interviewer:** At that contact, did you overcome those psychological aspects?

**P2:** Yes. I was a mess. I was an absolute mess. I would be like that person where we'd be at practice and-- I only ever did non-contact the first time with my team because I was back in Australia when I re-did it. I was with my ex-boyfriend, so I wonder why that didn't work out. We were playing one-on-one and he'd been taught how to play with my trainer and the physio, he'd been taught how to literally just slide in front of me so I could change direction.

I don't really have a memory of what it was like with contact then. The second time, I had a really great trainer, he would just push me to this real uncomfortable level where he'd-- throughout my rehab process, there would be like perturbations every time and real kind of intense ones where I was landing on my leg with contact way before I even got to the stage of contact in the game, but it was still like the second you walked out of the game, it was just terrifying because it became real and it was so uncontrolled. I didn't deal with it very well. The first few games were very messy.

**Interviewer:** Messy in what way?

**P2:** My skill level.

**Interviewer:** Oh, okay.

**P2:** I was just thinking so much about my knee and worrying so much about that. I probably over-compensated with my left knee or my left leg, and over-compensating by a lot. Suddenly, I became a 3-point shooter, and that was something that I wasn't really before.

Things like that. My skill level definitely went down the first few games, though I've gotten over that pretty quickly. That first period of out-of-control contact was very scary.

**Interviewer:** You mentioned the word "worrying." What does worry mean to you?

**P2:** Constantly thinking about it. It's always on my mind. Even when I shouldn't be, when I should be listening at a time-out or thinking about the next play, my knee was always on my mind. I was also wearing a massive brace that they would put you in at the states, so it was hard not to think about because you'd just look down and be like--

**Interviewer:** Was worry a theme of your journeys throughout the ACL injuries?

**P2:** Yes, I would say so, but it was more worry about when I was going to get back and how I was going to be when I would get back, more than my actual knee at the start. It wasn't until I got to the end where like I said, the thought of redoing it was real that the worry had shifted from that to my knee. I would definitely say I've always worried about something, yes.

**Interviewer:** What about now in your current level of sport, are you still worried about it?

**16 40**

**P2:** Oh, yes. When I was training with the team at the start of last year like before the world was shut down, this year, earlier this year. I was training with them I think I jarred it one day. I went to push off really hard and go the opposite way. I knew straight away I was fine. I knew I hadn't redone it, but I was in so much pain and also just the worry and the stress, I just burst into tears.

I had to walk off the court. Granted, I couldn't actually walk. I was limping. It's like rolling your ankle, you roll it and it really hurts, but you know you're fine. I'm not a crier either. I

didn't cry when I first did it. I didn't cry when I found out. I'm not a crier. That said a lot, just the fear at that point. Yes, I knew I was fine and I knew it was just a little bit of pain. I figured I just jarred it, but the worry of like, "Oh my God, my knee," just turned me off.

**Interviewer:** How distressing is your knee to you at this point then?

**P2:** When I'm playing or in general life?

**Interviewer:** In general, yes, and when you're playing.

**P2:** In general, it's just annoying because I would just do things like go for a run, and halfway through my run, I'll be like, "I'm not running anymore."

**Interviewer:** Because of pain?

**P2:** Yes, pain. It would just be pain. I'm pretty bad with-- I know you're supposed to watch the swelling and that's supposed to be a tell, but I'm pretty good at just ignoring that. Only when there's pain to a point of I can't run anymore that I will stop. That's just annoying. Things like if I go for a picnic sitting cross-legged, I can't do that. I can't kneel, or I can but not for a long period of time. It's more annoying in my everyday life. I'm not stressed about it. With basketball or any kind of contact sport-- I play a game of netball, and even then, I was playing goal shooter and it was a rep game, and the whole time, I was like, "Uh-oh." It just stresses me out.

**Interviewer:** What does the stress mean to you?

**P2:** That and the worry are very similar for me. It's a constant thought process revolving around my knee. What could happen? What could go wrong? I'll think about things that have

just occurred, like if I landed differently then, it would have gone, and things like that, just a constant thinking about it when I really don't need to be.

**Interviewer:** How do these terms, the stress, the worry, and the constant thinking, how does that relate to fear in your mind?

**P2:** The stress and the worry is thinking about is because I'm fearful of hurting it again. The fear is the reason for the stress and the worry.

**Interviewer:** Say you were to hurt it again, what is your biggest fear?

**P2:** Back then, when I did it the first time and the second time, I had so much to lose with doing it. Especially the first time, I had my life set. I knew exactly what I wanted to do. then I did it. The second time, it was like I had already thought about other avenues of life. Now, if I did it, I don't know what would change. My whole life, the trajectory I'm on wouldn't change a whole lot because I've just moved away from basketball because of my knee.

I don't know why I'm so fearful now. I think it's just the trauma of doing it maybe, and like I said, the thought of not being able to run around with my kids and the knowledge of like you actually can't play basketball again now. You actually can't do this anymore because three is one too many times. It's three too many times.

**Interviewer:** When you talk about trauma, do you have any dreams about the injury?

**P2:** Not anymore. When it first happened, I used to have flashbacks. I'd just be driving my car or something, and I was like-- Oh, God, now that I think about it, I can hear it because it pops. It's that sound of like-- The first time when I did it, it was like, "Oh, that's weird. I think I've done something to my knee." I jumped back up and I was like, "No, I'm good." I tried to

walk and I was, "Oh, that's not happening." The second time I went down, I started screaming, "I've done it again, I've done it again."

I knew it straight away. After that time, after that time I'd just be driving or walking or anything and I'd just have these flashbacks to it happening. It was so dramatic. I felt so high maintenance that I just try not to say anything about it. It got to the point I had to mention it. It was happening a couple of times an hour like it was all the time. It wasn't dreams. At nighttime, I'd never really stress. I have dreams about other people during their ACL and I still do, random people.

I would just have dreams and I'd always wake up and I would think like, "You need to so some extra squats today. I just had this dream last night," but not so much anymore. I saw a psych for it once over there because I was like, "This is ridiculous." I didn't really like him, so I didn't go back and see him. I just dealt with it myself, and eventually, they went away.

**Interviewer:** What kind of strategies did you employ to deal with those psychological factors?

**P2:** Not many. [chuckles] I just hoped they went away and think of other things pretty much.

**Interviewer:** You mentioned the psychologist. At what point in your rehab did you see him?

**P2:** It was the second time. It was probably two months in, so two months before I returned to play as well. I was struggling so much with the flashbacks and all those things. Also, I'd be like, "How am I ever supposed to play again if I can't even go for a walk without thinking about it?" I was just quite worried that I wouldn't have been able to overcome the fear in time to get back on the court. I went and saw him once, and I just decided it would have to be something that I would figure out when the time came around. I did.

<u>**P2**</u>

**Interviewer:** Was there anything that you particularly remember that helped you get back or was aiding your psychological aspects?

**P2:** I became very, very good friends with my physio trainer over there. I refer to my trainer as the physio essentially, athletic trainer. He was quite young, a couple of years older than me. We became really close. We were spending day together rehabbing as well. He was always there. He was there every practice every day, things like that. He became my sense of calmness and strength in a really weird way. Having him there was almost this reason of like, "I'll be okay."

If anything goes wrong, if I hurt myself, if I'm not feeling good today, I knew that I could just look at him and be like, "No, which I never did, but like it was having that option. He was probably a massive factor for me. I also think that he is probably half the reason that I have yet to really be able to commit here because he is not here. I don't have that person here. Anyway, it doesn't have to be him per se, but I don't have someone here that I can look at and be like, "This isn't what I want." I've got a physio and I had a PT for a while, but they're not there all the time. I see them once a week, twice a week, or once every 2 weeks. Having that person for me was a big thing.

**Interviewer:** Did any health professional ask you about the psychological aspects or considerations of your injury at all throughout the initial part or rehab or return to sport?

**P2:** My trainer did. My trainer did, but it wasn't so much--

**Interviewer:** At what point?

**P2:** When I wasn't doing well, he'd pull me aside and be like, "What's wrong?" It wasn't related to the injury as much. It was always related to me like during that-- my Grandma

passed away after the first one. He thought it was the reason for a lot of things when maybe it wasn't, or I was having a really crappy day. It was after my comeback and an article had come out. It was talking about me coming back, but they didn't know I had re-torn it.

That was a bad day, and he was, "What's wrong?" but it was to do with that. I don't know if we ever really spoke about the psychology of just the injury in itself. It was always about me and my headspace which, in a weird way, are two very disconnected things like the psychology of me, and there's a whole lot going on there, but the injury is something separate, although they come together at some point. No one really spoke about the injury. It was always like, "How are you doing with this? How are you doing with that? How's school?" It's like taboo a little bit in a way.

**Interviewer:** What do you mean it was taboo?

**P2:** Not taboo, it was just like avoid the question because the answer is not going to be good because I'm in the middle of rehab. That's not going to be fun. The psychologist did, obviously. We spoke about it. The surgeon couldn't give a crap, but that's not his job. I saw him three times, four times, and then plus surgery. The doctors didn't care. It was just probably my trainer who was the only one that actually kind of addressed it, but it was usually if I wasn't doing well, it would get brought up.

**Interviewer:** That was throughout your whole rehab. Was that through both of the first and second?

**P2:** Yes.

**Interviewer:** What about now? Have you discussed the psychological aspects with anyone?

**P2:** No. It was just an understood thing people would be like, "Are you going to play again?" I would say, "Oh, no." "Are you scared of your knee?" and I was like, "Yes," and that would be the end. That's the conversation. More like an assumption, not a conversation.

**Interviewer:** Okay. During that return to sport phase, you mentioned that you chatted a little bit and everything else. How confident were you about your return to sport at those phases?

**P2:** The first time, I thought I was about to be the best player in the NCAA, I swear, at the time. I was so confident. I've always been very skinny and not slow but definitely not the quickest person. I gained so much muscle. I was so strong. I'm trying to like doing it again. I was distraught because I was just at the top of my game. I had worked so hard. My shot was on point. Everything was just going great. That time, I was super confident.

The second time, it had been four months and I could only be on court for two, if that. It was more like let's just get through this season and figure it out as we go. I wouldn't say I was confident, but I knew what my team was. I knew what they needed. I thought I was able to provide that. I was a little confident being good for my team. I definitely wasn't confident in being the player that I was.

**Interviewer:** What about at this point in time, how's the knee injury affecting your confidence?

**P2:** It just changed my expectations for myself maybe. I would never have gone into a team being like, "Oh, I'm happy just to be a good leader and be a bench player and be a role player." That was never me. My 15-year-old self inside me hurts a little bit hearing myself say those things out loud, but now, I am happy to do that. I'm more than happy to just be in the team, live up the experience and the leadership, and just come in for a couple of minutes here and there.

The team that I'm on probably needs me for more for than that, and I don't want to be more than that. That's such a weird dilemma to be in now, and they're like, "We need you starting and playing the whole time,and was like, "I don't really want to. Bye." I'm happy that my perspective has changed a lot.

**Interviewer:** In that regard, has your perception of yourself changed because of the injury?

**31: 20**

**P2:** Yes, but that's probably a whole bigger and broader sense.My perception of myself has changed, which I think is for the best long term. This is one good thing I've taken out of it. I was a basketball player, and that's what I related to everything. If I was getting caught in a time where I wasn't playing well, then that affected my whole life. Whereas now, it's like the injury definitely-- this is so irrelevant, but being a female, you're never going to make that much money unless you're the top top, which I never was.

The injury has changed my perception of like, "I'm more than a basketball player." There's more to me. Basketball is part of it, not even that much right now, but my life and my self-worth is beyond basketball.

**Interviewer:** By the same token, do you think that the way that other people perceive you has changed?

**P2:** Yes. I get different comments on this. I earned a lot of respect over in the States for how I went about my rehab, especially, obviously, the second time. I don't think people were really

surprised when I stopped playing as much over there because they were like, "All right, we can see you're a hard worker. You'll be good with whatever." Over here, I get two different responses. I either get, "Yes, you're smart. Go find something else. Go pursue a career outside of basketball. That's the smart way to go about it," or I get that like, "Oh, you could play. Why aren't you playing? You could play. Why aren't you?" Because the WNBLin Queensland right now.

I get a lot of questions of like, "Why aren't you up in Queensland?" You can just tell. No one's going to judge me and be like, "Oh, you're soft. You should be up there," but the questioning is there and the assumption of, "Oh, you're just taking the easy route," is definitely there.

**Interviewer:** How do you feel about that?

**P2:** That doesn't bother me because I know what I'd be earning if I was up there. [laughs] It's not much.

**Interviewer:** Perhaps if they were earning more, do you think you'd be more-- would that assist with the--

**P2:** If it was a more viable career, yes, absolutely. If it was a more viable career, it might have been something that I actually pushed for a little bit more or really tried to overcome more because it was definitely much easier to say, "Oh, why wouldn't I stay here?" Like I say, risk versus reward. My risk to stay home, working out like I'm still super fit and healthy, is so low, but my reward is much higher.

If you're talking monetary reward or even just personal reward, I'm quite happy. I'm in a good mental space and all that now, but if I went up there, my risk of retearing my knee is

astronomical. Who knows what mental states I'd be in, but I know what being isolated with a bunch of people can do to you, and it's not usually that great for your mental space. Obviously, the monetary reward is definitely not the best. That's my thinking right now. I have zero regrets of not going up there.

**Interviewer:** How would you feel at the moment then if you were faced with a fearful activity?

**P2:** You mean fearful being?

**Interviewer:** Something that--

**P2:** Playing basketball?

**Interviewer:** Yes, or anything that you would consider fearful. Playing high-level basketball, some people consider maybe skiing or something like that. How do you feel when you're being faced with something like that?

**P2:** I don't know. I really avoid those moments. I think about skiing and I go, "Woah, no," but I would love to ski. I love skiing, but I haven't even thought about doing it. Definitely avoiding is my first thought process. Then, if I absolutely couldn't avoid it, I would just be very cautious. I would change everything about how I was doing that to try and be as safe as possible, if that makes sense.

**Interviewer:** When you say avoiding-- Sorry. You go.

**P2:** Oh, I was just going to say I'm not going to go and ski down those massive slope at the start**.** I would definitely start on the kid hill.

**Interviewer:** When you say avoiding, is it something that you would think about and then deliberately not think about? Or do you mean avoid as in you would think about it a lot and then maybe not do it?

**P2:** Oh, I would avoid doing the activity if possible. If there was a way I could get out of it, I would get out of it. I went to Hawaii with my partner and his friend, and they were playing one on one and they're like, "Come on, jump in," and I always would've jumped in. I would love to do that. We're in Hawaii, the palm trees were beautiful, and I was just like, "No way. Not risking it." I was playing after this, so I guess there was that risk of if I do something, there actually is something to worry about. I go out of my way to try and not be in those situations.

**Interviewer:** Reflecting on it, what's the effect this injury has had on your total wellbeing?

**P2:** In a weird way, it put me through crap. I was in a really bad space obviously for a long time. The rehab is a really, really lonely place to be, especially when you're part of a team sport. Long term, apart from the fear and the avoidance, it's changed the trajectory of my life, but I also think that the actual injury itself and what it made me deal with has really-- and I know you've probably heard this cliche a lot, but it's really the best thing that's ever happened to me because it forced me to face things that I didn't want to face and taught me how strong I can be and what you can push through.

**Interviewer:** You've developed some resilience in a way.

**P2:** Yes, that's a good way to put it. Exactly.

**Interviewer:** All right. Thanks so much for that. One more thing. The loneliness, can you just elaborate a little bit on that? I'm very conscious about time right now though.

**P2:** Thanks, I didn't notice that as well. The loneliness was every lift I ever did, every workout I ever did, every pre-injury, anything, the only time I would be by myself would be like an individual, one-on-one workout with the coach, and even that, the coach was there. To suddenly go from that, and especially at the time when I first did it, I was traveling.

You go from that to a hospital bed by yourself, everyone's there for you, and then suddenly, everyone has to go to work and you have nothing else to do. Then, your rehab is "Okay, just go over there and do it with the band over there," or, "Go do your ab work upstairs," or "Go for a run," and everything is by yourself. You go from being in this team environment and having people to support you. Everyone was amazing. All my people were amazing. I have no complaints, but you have got to do 90% of it on your own.

**Interviewer:** That was difficult?

**P2:** Yes, it sucked. I'm a people person. I like to be around people. That's why I play a team sport. It definitely was not fun.

Time: 39 min 30 seconds
