# Supplementary material for: Understanding Fear after an Anterior Cruciate Ligament Injury: A Qualitative Thematic Analysis Using the Common-Sense Model
Source: Int J Environ Res Public Health. 2023 Feb 7;20(4):2920. doi: 10.3390/ijerph20042920 (PMC9957354; doi:10.3390/ijerph20042920)
Supplement: Supplementary file 1 [file ijerph-20-02920-s001.zip › File S1-P3 Transcript.pdf]

P3

P3 Transcript

Interviewer: All right. Cheers, so today you'll be participating in a study that is researching fear post ACL injury. This interview consists of a series of open-ended questions that will explore your fear, your levels of stress, and your confidence after injury. Please feel free to say as much, or as little as you want on any topic, and if you feel uncomfortable at any time, let me know and we will move on. That sound all right?

P3: Will do. Yes. Sounds good.

Interviewer: Right, so tell me about your ACL injury.

P3: I did it at soccer training, just during a full-blown practice game. Ball came to me, pushed it aside, heel got caught in the grass, went over, the knee gave way and I felt it snapping. Because I'd worked at the snow previously and seen a lot of skiers do it, I knew straight away just from how it reacted that that's what I've had done.

Interviewer: What was your timeline on that? When did all that happen?

P3: I think that happened in 2016.

Interviewer: Then surgery and everything else?

P3: Yes, so pretty well the next day I went and saw the physio that we had attached to the club at the time and he was working-- Not for, but with Leo Pinczewski, the knee surgeon that I ended up seeing. He started me off on the pre-hab straight away and just sent me down that track for the surgery and everything like that. I just went from there.

Interviewer: Now, so you had that surgery pretty soon after the injury in 2016?

P3: Yes, I think I'm pretty sure I had to wait or had to do four or six weeks prehab before I could like get in and get the surgery done. Then, I got in as soon as I could to get it.

Interviewer: On that questionnaire, you filled out online, you indicated you might've had a little bit of fear associated with your ACL injury. Do you want to tell me a little bit about that?

P3: Definitely. Yes. I think, about it a lot. As I mentioned, I was a snowboard instructor for four or five years or whatever. I saw a lot of people do it there and a lot of them had redone it again. The fear I had was not so much sport-wise, but I'm a builder. It stopped me from working for quite a long time, and it was just really hard and I'll feel like, now I know how often people redo it again when they do go back to sport, I'm super paranoid about that happening. I've stopped playing soccer. I haven't really gone to the snow much since.

I still play. I'm a decent golfer, I still play a fair bit of golf, and a little bit of cricket, but even cricket, I still have problems with my hamstring and stuff like that. Work-wise, I have dramas like walking on the roof when the roof's not on and hopping up and down ladders and that stuff. It's always in the back of my mind.

Interviewer: What specifically is in the back of your mind?

P3: Well, potentially doing it again or having it give way or, I started tearing my hamstring. My hamstring tends to go still. I don't know.

Interviewer: Is that as a result?

P3: Yes. Well, that's where my graft came from, so I'm not sure what the numbers or whatever, but I'm pretty sure sometimes it just doesn't repair properly. Does it? I feel I had all the time off work to rehab properly, did rehab, to the letter with my physio, and yes, I still have problems with my hamstring.

Interviewer: What would doing it again, what effect would that have on your life at the moment?

P3: Well, it'd be really bad work-wise and financially. That's the main problem, work-wise and financially I'd be stuffed. If I had to go through the whole surgery and rehab process again, it'd be pretty bad, I think.

Interviewer: Would you think about that say when you think about possibly returning to the snow or soccer, is?

P3: Definitely soccer. All my mates are still playing soccer and they're always like, "Oh, come and have a run next year and we'll play over 35s now. I do sometimes miss it. I will get excited and I think look If I just went and played and took it easy it might be okay, but then, the common sense of me is just like, "No, just don't do it. It's not worth the stress and the chance of doing it again," I guess.

Interviewer: So at that initial injury? How were you feeling? What were your emotions after that initial injury?

P3: I was pretty devastated just because I was aware of like how bad of a process it is, to be honest. I'd seen so many people do it on the snow. As I was saying, I knew how bad it was, straight away.

Interviewer: What messages have they told you or had you processed because of seeing previous experiences of the injury?

P3: Well, what was really bad was probably six months of the ski season, that had just gone. My girlfriend at the time had just done hers, right in front of me. I'd seen that whole process and, I knew what I was in for because of that.

Interviewer: Your girlfriend had done it six months prior to you doing it?

P3: Yes. Maybe not even that maybe not much before that. I was standing in 10, 15 meters away from when I saw her do it, so it wasn't very good.

Interviewer: Wow, so at the time of your injury then knowing all those things, would you say you were distressed?

P3: Yes, definitely. I was very distressed. I was even worried about what would happen work-wise as well. At the time I had a really good job, a guy I had worked for a long time and he relied on me a fair bit and I knew that having that-- because I wanted to do the rehab probably that I'd have to have a fair bit of time off work. I'm always worried that I might get replaced and all that stuff.

Interviewer: What is that work?

P3: Well, that ended up happening. That was stressful as well. When I was fit to go back to work, I had to then go and find another job and all that stuff.

Interviewer: That word, "worry", what does that mean to you?

P3: Yes, I just worry, because of that and what I went through, I worry that I'd have to do that again and I'm not sure how well I would deal with it. [chuckles]

Interviewer: From an emotional side of view or a mental health point of view, where would that leave you?

P3: Oh, it left me pretty devastated. Losing my job. Not that I lost my job either, he needed someone to replace me, which is fair enough, but then having to go and find another job and all that stuff was pretty difficult and hard to take just because of a stupid soccer injury. Then not being able to play soccer again has been pretty disappointing. I've lost my interest in going to the snow, if I'm honest, because of it. Ultimately, I love golf and that's probably the sport that I'm best at. I just wanted to be able to get back and be good at that again.

Interviewer: You've just touched on it, but how has your perception of yourself changed then due to the injury?

P3: I've gotten super lazy and fairly unhealthy compared to what I used to be. I'm probably 10 kilos heavier than then. Not that I was ever a huge fitness buff, but I was pretty fit for someone. I could play two games of soccer quite comfortably on a Sunday, in first grade, and be fine. Now, I'm pretty lazy in that regard just because I used to run a lot for fitness and now I just don't like doing it anymore because it hurts. That's pretty disappointing. There's obviously other things that I could be doing, but I've just let it get to me, I guess.

Interviewer: What do you mean? How's it got to you?

P3: I don't know. I think I just got lazy and I've gotten into a bad routine of being lazy, fitness-wise. I think that's it.

Interviewer: Is that something you think that could change?

P3: Oh yes, definitely. It's obviously just self-discipline, but that's what started it and it was-- Yes.

Interviewer: Do you think your knee function could improve then, into the future?

P3: Do I think it would improve?

Interviewer: Do you think it can?

P3: Yes. I think so. Overall, I'm pretty happy with how my knee feels. It's just the problems that I have with my hamstring. It is like sometimes I do think, "Oh yes, shit, that feels a little bit wobbly" but It's mainly the tearing of my hamstring that bothers me.

Interviewer: Yes. You mentioned the words bother and disappointing. With the word bother, what does that mean to you? What does being bothered mean?

P3: It bothers me that it dictates me from doing things, I guess. If I wanted to play soccer Or if I don't want to play soccer, I could make that decision myself but this makes it for me, if that makes sense.

Interviewer: Yes.

P3: It bothers me that that's not even a possibility for me.

Interviewer: Do you think that bother or do you think your confidence could improve into the future, to go back?

P3: Yes, I think so. It could if I spent a bit more time, I guess, doing more, getting back into rehab stuff for it again, and trying to sort my hamstring problem out. I guess I would probably feel a lot more confident with it. It's just that, it happened, I can tread on a piece of timber at work and stumble a little bit and my hamstring will semi tear. Or sometimes when I hit my first drive at golf, on the first hole, my hamstring will tear things like that. It's constantly in the back of my head that, "Oh, shit. I better not trip or--" Yes, stuff like that and have that happen again.

Interviewer: Overall, what kind of effect then has this injury had on your confidence?

P3: I don't know, it's just always there. I've just gotten used to it like, "Oh, shit, don't do that, don't do this, be careful with that thing." Even today, I was up on a roof, getting up and down the roof all day. I'm still super worried about where I put my feet and stuff like that.

Interviewer: Yes. Does it ever get you down that you just mentioned the word "worry"?

P3: Yes, sometimes I do get a little bit down that I can't-- Like, all my friends when we're sitting around and then talking about playing soccer and stuff like that. I do feel a little bit left out and stuff like that.

Interviewer: Yes. What kind of effect has that had on your life? That feeling of being left out?

P3: I don't know. I try not to let it bother me too much. I guess like at the time, I think it would be nice if that was a possibility for me, but then I'm also grateful that I'm still all right. It's just that little thing. Yes. I try not to let it fully bother me. It's just a little bit annoying. It bothers me. It's a little bit annoying.

Interviewer: When you're sitting around with friends and whatnot, do you think that their perception or has anyone else's perception of you changed, because of the injury then?

P3: I don't know about that. A lot of them understand because a lot of people were, we know a lot of, after I did it, two of the other mates did it fairly quick after me, within a couple of months, the same season, after me, they know how bad it is. A lot of my good friends are PE teachers, so they're fairly aware of how severe it is and how bad injury it is, and how hard it is to get over. Yes, I think they understand but, yes.

Interviewer: When you say that it helps people know how bad it is and how hard is it to get over it. Do you want to tell me a little bit about that?

P3: I guess, people, it's pretty well known how bad it is. If you're interested in sports or if you see a professional sportsman do it and how long it takes them to come back from it, with their professional help compared to just me, some carpenter [chuckles] that's just has to do it all myself and stuff like that, I guess. They're all super supportive and have been pretty helpful over the course of me doing it.

Interviewer: When you mentioned the professional help, did at any time, did any health professionals that you're involved with ask about any psychological considerations of the injury throughout?

P3: No. Not at all.

Interviewer: Not at all?

P3: No.

Interviewer: Did anyone, did you ever chat about any emotional aspects or psychological?

P3: No, not particularly just a lot of people started checking in on me to see how I was doing but not proper conversations about mental health or anything along those lines, because of the injury. It's just people being decent people.

Interviewer: Do you think that's something that would have been appropriate or helpful, or do you think that's an aspect?

P3: Definitely, yes. It should definitely be made aware that it's long term, it's not something that you have the surgery, do the rehab in and everything's all good. It probably should be mentioned at some point that it could have a long term effect on you.

Interviewer: Yes. What stuff like long term effect, emotionally or psychologically, do you think this injury brings with it?

P3: Just the ongoing worry, I guess, about redoing it or doing my other knee. There's so much information about that stuff like, "Oh, yes, the chances of you doing it again now are blah, blah, or, the chances of doing your other knee now have increased massively." There's heaps of that information and everyone talks about that stuff but not the long-term effect of it.

Interviewer: Yes. How does that make you feel when you consider that long-term effect of injury?

P3: It's a little bit-- I don't know, because it's been a little while, I guess I've just gotten used to it, but I think it's just a little bit, I don't know what the right word is, It's just a bit daunting that now, it's something that I've got to live with now.

Interviewer: Yes. What would it mean to you to go back to skiing without worry or distress?

P3: It would be awesome. It'd be the best feeling ever, just to go back to doing anything without worrying about it would be awesome.

Interviewer: Do you think that's all? You said with the rehab maybe you could get there?

P3: It probably is just a bit of a confidence thing, I guess. I guess I just have to get into it and say how it went. It's just that, I guess taking that first step, getting back into it and going over a 30-foot jump and landing badly and not having it be a huge problem.

Interviewer: How would you think about that, or view that

18:20

P3: Yes. That'd probably be a massive help, to be honest. I'm not going to play soccer again. I don't want to go out there and-- I guess, the comparison with soccer would be like getting out there and slide tackling someone or whatever, or getting like a big tackle or whatever, and not hurting or not having any problems with it, I guess, something like that, along those lines, will be similar. I guess there's certain things that I'd be willing to take on. The snowboarding would be one of them, because I feel like I'm a lot more controlled at that anyway, I can get my way around quite comfortably anyway, just from past experience.

Interviewer: Okay. How else throughout your injury experience, have you managed your confidence or your fear or your psychological aspects?

P3: I guess I haven't really done much, I just deal with it myself. I talk to my wife about it a bit when it bothers me and stuff like that, but I haven't really sought any help ever or anything like that. I know my limits at the moment and just stay within that, I guess.

Interviewer: If you don't mind me asking, when you're talking to your wife about the aspects that bother you, what kind of conversation is that or what kind of things come up?

P3: Well, it's like returning to soccer or skiing or whatever aspects. If I was to do it again, the financial stress, it would put us both under and that sort of thing.

Interviewer: Yes. Okay.

P3: I don't think it's, like I obviously don't want to hurt myself again and go through the pain of it and all the punish of the rehab, and everything, but it's the loss of income and that sort of thing. I'm a subcontractor, I run my own building company. If I had to stop work, it would be quite bad for both of us. It's just weighing up, if I think going for skiing or whatever is more important than being able to work and provide. Yes.

Interviewer: Yes. Pretty, pretty serious.

P3: Yes. [laughs] I said it pretty seriously. [laughs]

Interviewer: During your whole rehab, when you started to return, maybe just to walking, maybe at three or four months, did you have any fear or distress at the time?

P3: Not really, I was really happy with how the rehab went, because I had accident and sickness insurance through my work. I was able to do the rehab every day because I was off work, I was able to do the work every day and just tick off everything as I went, I was pretty happy with how it all went. It wasn't until however many months it is after you stop seeing

your physio, every week or whatever. I remember the first time, I think I was sitting on a wall somewhere and, something gave way and I had to catch myself and my hamstring just tore like that. I was like, "Fuck, that's not good." Just things like that have happened ever since.

Interviewer: Yes. That was after the 12 months, was it or?

P3: I'd say that would have still been within the 12 months, but it was a fair way after, I think I'd already gone back to work.

Interviewer: As you start to go back to work in about 12 months or so, what were your emotions at that time regarding your knee?

P3: I was just -- Obviously, I'm a carpenter, we have to get up in a lot of high places and stuff like that. I was always a little bit worried that it would give way and I'd stack it or I wouldn't be able to hold things and stuff like that. We stand on planks and stuff a lot and it's like, on and off it's just me worrying about, or I feel like I'm wobbly and stuff like that. It stresses me a little bit.

Interviewer: Is that even now

23:02

P3: Sorry, Yes, today. I don't know if it's me being paranoid or whatever. I'm not terrific with heights, but I'm not bad but I'm still always like, "Oh shit. Where am I? Where am I standing? Hope there's nothing that I can trip on and stuff." [chuckles]

Interviewer: Let's say then, currently, how do you respond when you're faced with a fearful movement?

P3: I'm quite cautious, to be honest. Especially at work, I'm always setting things up. I'm pretty slow mover along the planks and that stuff. I'm always mindful, make sure it's as safe as possible for me to walk on, I don't have to worry about my knee and I'm not tripping, I'm always like, "Okay, I'm not going to do this or that and stuff like that" just, like, precautionary stuff, I guess.

Interviewer: Yes. I think of this word "worry", you said a few times, what does "worry" mean to you?

P3: It just comes back to not being able to work thing.

Interviewer: It just ties right back?

P3: Yes. Just worried about it causing me another problem basically, that's what it mean.

Interviewer: Throughout the whole injury process how's your mental health been?

P3: I'd say pretty good. I'm pretty much realistic going, I knew that it was going to be bad, and I knew that was going to take ages to get it right and all that stuff. I haven't had any mental health problems for now I don't think-- That's not true, I've got super down about it but I don't have ongoing issues, I don't think.

Interviewer: When you were super down about it what was the main thing at the time or was it mainly worrying about work and worry about not being able to provide?

P3: Yes, the worry part of it was the work and not being able to provide and that sort of thing. The thing that upset me was the not being able to do sport, that was upsetting, but it wasn't the be-all-and-end-all, it was more worrying about not having a job and that stuff.

Interviewer: Yes. All right. Awesome. Thanks. That's quite a bit of information there, isn't it?

P3: Yes, I hope it's been helpful. Sorry, it's been a mission its been so busy, things just keep going wrong at work.

Time: 26 min 05 seconds
