# Supplementary material for: Understanding Fear after an Anterior Cruciate Ligament Injury: A Qualitative Thematic Analysis Using the Common-Sense Model
Source: Int J Environ Res Public Health. 2023 Feb 7;20(4):2920. doi: 10.3390/ijerph20042920 (PMC9957354; doi:10.3390/ijerph20042920)
Supplement: Supplementary file 1 [file ijerph-20-02920-s001.zip › File S1-P4 Transcript.pdf]

P4

P4 Transcript

Interviewer: Okay, thanks for taking the time today. Today we are participating in a study that is researching fear post ACL injury. Interview will consist of a series of open ended questions that will explore your level of fear, your distress, and your confidence after injury. Please feel free to say as much or little as you want on any topic, if your uncomfortable at any time let me know and we will move on. How does that sound?

P4: Sounds good

Interviewer: All right, so tell me about your ACL injury.

P4: I play hockey, that's my main sport. I was playing in the emergency services game, so I'm a paramedic. It was just your ambulance, fires, and police, all playing together. Essentially I was running one direction, a guy was running in the other direction, and I just had a lot of friction on my new pair of shoes and I went down like a sack of potatoes essentially.

Instant excruciating pain. I've been hit multiple times before playing hockey, broken multiple bones, but this was-- "I knew something was wrong thing," instantly. I did ACL, PCL, MCL, and full-thickness meniscus tears on both medial and lateral and ended up having three surgeries to correct. I think the number of surgeries was slightly related to having a very cautious surgeon, and then also the fact that it was on work cover, so money wasn't really an issue. Otherwise I think I probably could of squeezed it into one maybe two. It just meant it was quite a long recovery.

Interviewer: You hit each other? What do you mean there were friction issues?

P4: I had a brand new pair shoes on. I hadn't warmed up at all, it was really social, not drinking beers, but super social. I had a brand new pair of shoes, so the ball is coming, basically towards me and-- The ball's going this way and I'm running this way, and there's a guy coming from the other direction and I've got to do a poke tackle so I stepped- so it was my left ACL- I stepped forward almost into a front lunge with my left foot forward.

He's come through to avoid colliding, kind of like forward up, and as I done that, I hyperextended my left knee and he then fallen on top of me. It was the initial hyper extension, probably ACL, and then everything else in a twisting motion around as I was sat on.

Interviewer: Okay. What was the timeline on this? When did this all happen?

P4: When did it happen? April 15, 2016.

Interviewer: 2016? The surgeries straight after?

P4: All that in the year, yes. Three months and then a couple of months after that, and then another one six weeks after that. Quite quick succession.

Interviewer: In regards to the injury, we've got a little online questionnaire. You mentioned you experienced fear as part of your knee injury. Do you want to tell me a little bit about that?

P4: I guess I very quickly realized that the type of work that I do that this injury instantly changed my life in terms of that I potentially didn't have a job. Not being physically able to get around or get around in the same way I used to be able to**.** It's a surprisingly physical job anyway so already it put a damper on my longevity in that sense and then anything I was

doing I was well I need to protect myself to be able to work because at the end of the day I can't work, I can't pay rent, etc, etc.

I guess that was probably the biggest driver of fear. It was just re-injuring or just not being able to function in daily life, let alone the fear of not being able to do things that I enjoy or like. It became like a survival thing, I think. Sorry. It sounds really dramatic but in all honestly, it was was. [chuckles] If I can't do my job, at that point in time I didn't have any other qualifications so it was pretty much I now need to change how I do things to make sure I can keep working.

Interviewer: Yes. So how did that make you feel, those feelings that your very survival was at risk?

P4: I guess initially it was a little anger and frustration and the whole "Why me?" kind of thing. Angry at myself in terms of playing a competitive sport for a very long time and just not warming up and things like that, just running through things in my mind thinking, "Would that have made a difference?" Now, I guess, all these years later it pushed me into going back to do some study and diversifying a little bit more. I guess it probably was a good eye-opener and it helped in that sense. I know I say like this is a benefit out of it in that sense, but I wouldn't go through it again. I guess just anger really just ended up really pissed off.

Interviewer: You said angry at yourself. Do you blame yourself?

P4: Yes. I had this weird pain in my knee at the gym a couple of weeks before, ignored it. It was a couple of weeks before, like after hockey season started, and so I just always think that if I had warmed up a bit more and if I maybe hadn't of gone because I wasn't supposed to be playing, because I had a practice match the next day and all these things. I just wanted to go

for a bit of fun and it's like well, "Really? Was that worth it in the end?" Maybe if I listened to my body a little bit more that it might not have happened.

Interviewer: What is that anger mean to you? How, that word, how would you give that meaning, operationalize that word?

P4: What do you mean?

Interviewer: What does the anger -- I understand you are angry at yourself, but what does that mean in regards to your knee?

7:28

P4: I guess it was like a frustration. Hitting yourself over the head kind of thing, like you're an idiot-- I don't know how to explain it. [laughs]. I don't know sorry.

Interviewer: What were your emotions at the time of the initial injury?

P4: Fear. I guess that spiraled in terms of what does this mean? I had a very good feeling of what I'd done, I thought "This is definitely an ACL." I guess I was a little bit peeved that people didn't really want to say what it was. It was "you'll be fine, you'll be fine." Just sitting there in a hospital chair just wanting someone to be a bit more honest with me about it.

I was also moving house that night, I was meant to come home from playing hockey and move into a new place. I guess I just decided to go into a bit of panic mode in terms of, "Okay, crap. There goes hockey season." At that point in time, the sport feeling wasn't even

present, it was back to the life activities of me moving house and work and everything like that. Just uncertainty is probably a good way to describe it just a feeling of uncertainty

Interviewer: You mentioned "panic" a few times as well. How do you describe or what does this "panic" mean to you?

P4: I guess I'm a type of person that likes plan to make plans, so to speak. Panic in not knowing. I guess in that point in time I didn’t know anyone else who had an ACL apart from older people and I was like "Okay, I can't really compare myself to them." I didn't have any understanding, really, of what the recovery would be and what I'd need to do or how it would change how I do things in that point in time. I guess my mind just went to worst-case scenario and I was just left to sit and fester and wonder. Spiraled, in that sense.

Interviewer: Yes. Where did you end up with the spiraling?

P4: In tears [laughs] I guess, yes, I just felt sorry for myself for the first week or two. I wouldn't go as far to say I was depressed, but I was certainly just a bit mopey and you know, "Why me?" kind of thing. Then it didn't take long to I guess just accept it and just go, "Okay, I've now got to do something about this and just get on with getting it better."

Interviewer: How did you manage that fear and distress then, in that initial phase?

P4: I guess good family and friends' support to begin with. The best thing for me, in a weird way, is that one of my very good friends he did his ACL about a week after me. We ended up being able to I guess just talk about it. Being each others own little support network. He'd get told something from his physio and it helped me and made me querying that with my physio and just-- it was a good way to I guess assess myself to see if I was on track with my

recovery. Even though they were the same injury but different injuries, obviously. It was still a nice little way to make sure I was on the right path.

11:55

Interviewer: Did you guys discuss any psychological considerations of the injury?

P4: Probably not to begin with. Not in that initial phase. It was more, "How many degrees are you at," kind of thing. Just, yes, almost competitive in that sense. Yes. I think it changed-- I just moved away from a lot of friends. I wasn't able to drive initially. It's changed my entire friendship group. I think that it sounds so dramatic but it really did. There were friends who I thought were better friends but weren't-- I guess we would chat about that.

He would come up and meet me, kind of thing. Because he was a little bit further ahead because I had the extra surgeries. We discussed how certain friends were I guess a little bit more supportive and willing to travel and hang out with me and other friends who weren't. I guess how I felt about those friendships would be psychological trust that we had. And without really realizing it.

Interviewer: Do you think that the way that other people perceived you changed because of this knee injury?

P4: I think other people maybe didn't understand what the injury meant to me and how much of a big deal it was to me and therefore were like, "Oh, it's just an ACL. Everybody does an ACL." Everyone knows someone who's done it or you've done it yourself or you’ve done it multiple times. I guess when my behaviors changed, they weren’t so understanding of that

because I couldn't explain to them what it meant to me or how much of a big deal it was to me.

Interviewer: Yes. And if you could have explained it to them, what things do you think would have helped?

P4: I guess it would probably go along the lines of, "Wow. I wasn't able to work for basically nine months." I was pretty new in my career at that point in time, so there went work. Friendships changed. I was very competitive at hockey, I was playing a good grade, and I just wouldn't be able to get back to that.

What physios were saying to me: "Don't push yourself. Give yourself plenty of time." There was a couple of years of work. At that point in time I was giving myself a couple of years until I was then planning on slowing down. All of a sudden all I had planned for my future just was put on hold immediately and I didn't get that time to pull away from my sport, I was made to pull away. Sorry. Yes.

Interviewer: Yes. I guess going on from that, how has the perception then of yourself changed because of the experience?

P4: Well, I never really thought about that. I really don't know how to answer that question. I think- I mean, I'm still the same person. I just am more cautious. I think I was always that kid that would be jumping and climbing off everything and broken multiple bones and no fear really. [chuckles] No fear as a kid and now everything I do, I would probably think twice. I guess I'm just well. I guess I would perceive myself to be slower and just think things through a little bit more.

Interviewer: What kind of effect has this had on your emotional wellbeing?

P4: I think I felt really isolated when it first happened. I was saying how moving away and not being able to get around and see friends. I felt super isolated and that was probably the worst feeling. Even though all these people were there to support me, I was doing it away from work, away from school, away from sport, away from friends. Sitting at home in an apartment by myself just waiting for people to come home because I couldn't do a thing. Yes.

Interviewer: Yes. Doesn’t sound like fun.

P4: Now I think of it as a positive thing. It has definitely changed the course of my life for the better I would say. I guess it eventually pushed me back to university to just add a few things. Gain some more qualifications and just diversifying in that sense so I can-- It's almost like a plan in case I do hurt myself again. I've almost excepted that I’m probably going to hurt myself at some point. Therefore I don't want to let myself go through that crappy feeling of oh no, what do I do in that panic. It's like, "Okay, that happened except it. Get over the rehab and you’ve got your backup plan and the other stuff to get you through it.

Interviewer: What then would another ACL injury mean to you?

P4: If I did it again, I would not go back to sport after that. I can tell you that much. It would be-- I've gone back to hockey since, but nowhere near as competitive. Partly because of work just being difficult, doing the shift work. I wanted to play footy this year, and I had every intention of joining a club down the road. Pretty much everyone that I would tell, they'd go, "Oh, but what about your knee?"

There's just always this thought of a doubt in my mind that I can do it. Just underlying there and then other people bring that to the surface. Sometimes that makes me feel, "What's stopping me if I can?" Then at other times it's like, "Am i being stupid? This is a silly idea. Should I just let it be and just go to the gym, which is I can control that." If I was to say play fully and I did it again, I did another ACL, I wouldn't do it again. Being older as well just--

Interviewer: You said this word, "doubt", as well. How would you describe "doubt" in regards to your knee?

P4: I guess it's just not being a 100% confident and that it'll do its job. That the surgery is-- That I rehabbed it properly and that it's functioning at a 100% capacity, I know it'll never be the same. Because I was very diligent with my rehab and everything like that, but just every now and then you'll twist, or sit, or just do something, and there's always a little niggle there sometimes that constant reminder that, "Okay, it's not the same." I guess I just doubt, I doubt that when I move, will it hold up.

Especially with the new sport, I feel, with hokey, confident I guess in the movements that I'd be doing and almost being able to go into a tackle or something, knowing, or I guess withdrawing a little bit more from the more physical-- Hokey is not a contact sport but it can be very contacting, [chuckles] but yes, I guess even so it's just just how I would receive the ball and the EP that I work with from my rehab, it's helped me with specific hockey movements, so I guess it's doubt in doing a different movement that I haven't done before in that uncontrolled environment of a match.

Interviewer: You mentioned never being the same a few times, where is this message of never being the same come from? How is this message- how are you processing this thought, this belief?

P4: I guess it's come from just the feeling that I have in my knee in that. Even though I think I've got to full-range movement during rehab, it's still-- You know when you do a quad stretch? I can't get it all the way. It just feels different. It feels like it's not got the same amount of mobility through it. Every time I do anything and go downstairs, it's still, I'm less confident going downstairs, like step, step, step. I find that I always lead with my right and my left just catches up.

In daily life, I've got this constant reminder just from how it feels and how I move my body that it's not the same as my right knee. I don't know if I know what it used to be like before but I know that I treat it differently.

Interviewer: Do you think the knee function will ever--? Do you think? It can improve?

P4: I think this is what it is. From trying to remember what was really said and done when I was doing all my physio and everything but I was meeting all my milestones. Excelling at them and essentially I literally had nothing else to do in, so I would do this rehab and I did everything. At the end of it, I was thinking it pretty much it, this is- the knee, this is good and I think, at the time, I probably felt like it was moving better than my right knee, I rehabbed it so well and working with the EP and moving my body properly, but it's always going to be different.

Interviewer: Do you think it's possible to improve your confidence, or your fear or distress in this current time with it? Or really your perception of it?

P4: I think if I did get to play sport and it felt good, I think that would improve my confidence. It's telling myself that I can do new movements with it, having some more impact and things like that, I think that would improve my confidence. I think I just need to try a bit more, I was sort of mentally ready to try something different at the beginning of this year and then COVID just happened. I put a hold on that, but I still have every intention for next year to go and have a crack. It's just one of those things of like, "Well if it doesn't feel good and it feels bad, then I just won't do it," I'm just going to give it a try and see if I do have that confidence in that.

Interviewer: You mentioned "confidence" a heap. Could you just maybe give me a bit of a timeline of the initial injury, confidence, how it was feeling during rehab and then maybe how it was now?

P4: Yes, it took about nine months to finish up everything to get back to work. I guess during rehab I didn't really have any expectations other than just getting the mobility back into it. I guess that was my biggest fear at that point in time, is that I wouldn't be able to sort of straighten it and bend it and just didn't want to have a funny looking knee. At the end of rehab, that was, what they said, it was all good, that was a big confidence to me in that sense.

Getting back to hokey, I went back to rehab halfway through 2016 and then went back to Hockey in 2017, that felt more confident than back then and then probably 2018, I played full season at a different club at quite a high grade as high as what I was playing. It felt really good then and I performed quite well that year. I guess maybe last year I just had a couple of hammie niggles and things like that until then it just come back down.

It fluctuates, I guess, but definitely when I was playing sport and I guess that two-year mark afterwards, it felt good. That was probably the best feeling I had, but that was because I was pushing it every day and I was just like, "Okay, it's not like doing anything that I feel like It shouldn't be doing."

Interviewer: How were your levels of distress when you were playing sport?

P4: I wouldn't really say I was really distressed playing sport. It was-- In terms of the actual game, it would be out of my mind, I would go out on the field and I wouldn't even really think about-- Not consciously think of it, but every warm-up session, every time I'd warm up and maybe it would be just feeling a little bit off that day for whatever reason, I would tell myself, say to myself, "Okay, that hurt, maybe you won't do this drill because it's a lot of lateral movement. Okay, maybe I should just sit down." I would be talking to myself, monitoring it in my head.

Interviewer: Yes.

P4: I don't know if that makes sense, but-

Interviewer: Yes, it totally does.

P4: In the game, I didn't really have time to have those thought processes. That's why the exercise physiologist that I worked with, he was really good in-- He was the best thing ever I had for building my confidence. I didn't really know what it was, what an EP was at that point of time, and It was free. I was just like, "Whatever, its free". In terms of working with me towards specific hockey movements I now knew that I had that ingrained in me, so I was trying to rely on that.

Interviewer: Obviously, you guys had a great bond throughout that rehab. Did he as a health professional or did any other health professional ask you about any psychological considerations throughout the injury?

P4: The surgeon definitely not. He was as old and as cold as they come. Just know that it's none whatsoever. The physio, I went to a couple of physios because it was with work cover so I had to go to someone that my work told me to go do that at that point of time. he was lovely but he didn't really do a great deal and then I got palmed off to someone else. The latter half of it basically had no relationship with him. The EP was good and he was a friend of a friend so I think we just had maybe more of a nicer conversation. as opposed to… I don't ever remember anything directly in terms of how is this making you feel or anything. I think potentially it was just a lot of 'not to get frustrated', just give it time, they were probably sensing the frustration in myself so maybe they were. Nothing really direct from what I can remember.

Interviewer: Okay. You have mentioned that throughout that rehab journey you were very focused and you smashing it out, I think you said. From the mental health point of view how'd you find the whole experience?

P4: Draining. I particularly remember one day I had my full brace on and I would go to the gym every day and just do upper body and do my rehab at the gym, cause if I did it at home I would have absolutely gone batshit crazy. As I said I was isolated and bored and then I just started to feel sorry for myself. I really need to motivate myself to not just lie around.

The gym that I was at was quite a small gym and they were lovely and a couple of older ladies owned it and they were actually…..They weren't directly helping with my rehab but

they would ask how I was mentally and they were very much -- it's okay to be upset about this," because I just remember feeling really stupid. Feeling very flat and down over my situation. With my job as well where I see people in literally the worst of the worse and here I am moping around a bloody sore knee.

P4: Yes. Keep going.

Interviewer: How did you find that from your experience that how did you find the help they provided to you during your rehabilitation from a psychological point of view? The difference between-- You mentioned the surgeon was cold and old and that more the nicer, the ladies listening and being warmer. What kind of effect do you think those two different approaches have?

P4: I think that the owners of the gym, they helped me accept it in that they validated that it is an injury and that it can have, and it's okay that it has a big impact on my life. Just because other people might be going through really, really bad things, my life experiences are different to the next person. If this is the worst thing that ever happened to me, that's okay.

Versus the surgeon, I was literally a number and a dollar figure. As I said before, I'm pretty sure there wasn't really much discussion in regards to why I had to have the three surgeries and that always puzzled me I'm pretty sure it was just you don't really need to rush back to work because you are on work cover and you don't need to worry about how much these, three lots of anesthetic cost and blah, blah, blah don’t worry how much this is going to cost you because work's paying for it. It was just very much you were just a money-making machine.

Interviewer: How did that make you feel? That thought that maybe the surgeries were unnecessary and you were a financial target?

P4: I guess just sort a fed up and over it and just I can't be bothered. I just didn't really want associate with him. I was like, "Cool. Whatever." It was just the smallest amount of time I can spend with you the better. I was just disengaged from him. The first consult was fine, he was quite nice. He was fine, I didn't really know what to expect but then as we went on he wasn't able to explain things, wasn't willing to answer questions. Basically would just say "Come in on this date you are doing this?" I was very disengaged from him.

Interviewer: What kind of effect do you think that disengagement from you had subsequently on you and your ACL injury journey?

P4: I resented him and I resented having to have the multiple surgeries. They weren't told to me from the get-go either. It was I had one and then it was, oh now, you need to have another one, and then how about you need to have another. In my mind I was rebuilding and planning ahead. I just had to get through these few months in this amount of rehab timeframe, My life starts again on this date and then he kept pushing it back so I thought he was holding me back. Was probably some anger and resentment towards him and never really thought of how that probably made me feel but I probably was why I was so frustrated to begin with early on. I felt like I had a lack of control over my body and my injury.

Interviewer: You mentioned that lack of control actually quite a few times. What contribution is there and where does that sit in terms of this journey?

P4: I have a good understanding of anatomy and physiology with my job. Clinicians are never good patients for one, so I think my job doesn't help with that, I'm usually in control of

the situation. I guess it wasn't really what I would classify as patient-centered care. The surgeon told me what I had to do and to begin with that was fine but then it was never really much of a discussion. After that first time, and the physio side of it-- I just wish I could have sped up the process. I think maybe communication could have improved between myself and physio in what to expect, what timelines were realistic.

I thought if I just did this a million times a day that would maybe help speed it up. You know what I mean? I thought how can I get through this. I think just setting out from day one a better understanding, or a bigger picture, of what this could be, I think I was getting fed drips and drabs and that's why I didn't feel like I had control. If someone said to me from day one, "You may need to have multiple surgeries because you've done all this different bits and pieces. From the date of your last surgery expect a minimum of 12 months and from that date expect six months to be back to sport." I think I could of worked off that rather than lets have a surgery and maybe do some rehab with physio we'll see how that goes. The timeline around it is why I felt so out of control because I couldn't move on from it because I was just waiting. Then I was having people at work saying when are you coming back and I'm like, well I don't know. Then saying I'm coming back on this date and then the surgeon saying no your coming back in for a surgery, or the physio going well you can't go up and down stairs, so can't go to work yet safely. I think it just it all just comes back to communication really.

Interviewer: You mentioned before that your kind of personality trait is you like to be in control not to know what's going on at least. Do you think [crosstalk] the aspect of this lack of control injury was particularly maybe confronting or do you think that's had an ongoing affect about your particular aversion to returning to certain things?

P4: I think I missed the bigger part of that question. "The injury has had"?

Interviewer: Because you mentioned that your personality type earlier is you liked being in control, and then this whole experience sounds like it was very you know for want of a better word, you're out of control. You know however you want to say that. Do you think that's contributing to the current whole picture of being a bit worried or a bit fearful of the experience again?

P4: Yes, because at the end of the day. I won't know until I go out there and I play. There is no way to plan or prepare for it. I just have to go out there and do it. It's taking that leap of faith in terms of…. I can do as much as I can in terms of making sure everything is strong or rehab is as good as I can. At the end of the day, I'm not going to know what's going to happen until I just go out on the footy-field and have a crack at it. I think I just need to learn to accept that and I think I'm at that point now where it's sort of a 'well, you can't live wondering forever' because then you basically will do nothing.

In saying that, it's funny-- I'm willing to go and play footy, but I'm not willing to ski. I will not ski. My partner was planning on going to New Zealand and I'm like, "No." I will not do it because in my mind the risk is too great with skiing. I do my own risk assessment. [chuckles] Not based on any sort of fact or anything, just my own personal opinion of stuff. Just jumping and stuff that kind of thing, at the gym—they will want us to do a single leg jump and things like that. I'll do it with my right but not my left.

Interviewer: What's the biggest thing holding you back there, the jumping, or what's the risk assessment and what's holding you back from skiing?

P4: The thing with the jumping and stuff is the risk is there and I think it's an unnecessary risk in that I really don't need to do that jump. I can easily just do a step. I can easily do a

normal squat-- this doesn't impact anything. With the skiing, I don't really love skiing anyway. It's not like it’s something that I'm super passionate about. It’s almost an excuse in some respect in that I don't love it, I'm not really wanting to do it, and there is that level of risk there in my mind-- too hard kind of thing and just saying that I'm not doing it.

Whereas with footy I want to play footy and then the social side of moving to Richmond and joining a club, meeting people close by, and all these other things that are benefits weigh into that-- there is seriously no real method to it but it’s just hanging.

Time: 43 minutes 20 seconds
