# Supplementary material for: Understanding Fear after an Anterior Cruciate Ligament Injury: A Qualitative Thematic Analysis Using the Common-Sense Model
Source: Int J Environ Res Public Health. 2023 Feb 7;20(4):2920. doi: 10.3390/ijerph20042920 (PMC9957354; doi:10.3390/ijerph20042920)
Supplement: Supplementary file 1 [file ijerph-20-02920-s001.zip › File S1-P5 Transcript.pdf]

P5 Interview:

Interviewer: I will introduce the interview. Today we'll be participating in a study that is research in fear post ACL injury. The interview will consist of a series of open-ended questions that will explore your fear and levels of distress and confidence after injury.

Please feel free to say as much or as little as you want on any topic, and if you feel uncomfortable at any time, just let me know we'll move on. Sound okay? All right, tell me a bit about your ACL injury.

P5: I injured it at the start of 2017. It was just before a tournament and so I play Ultimate Frisbee. We do three-day tournaments in another city. We were just training in Perth and I was turning to defend someone and I think my foot just caught out, obviously injured my ACL.

I talked to my physio and they were like, "Look, we don't really think you should play." But I was a bit younger and really just wanted to play in this tournament and so I tried to play at the tournament about a week later and that's when I think I fully did it.

I think I had probably a grade two or three tear. Then afterwards it was just completely gone when I injured it the second time. Then I got it all fixed up probably about two months after that cause I had Uni and stuff.

Interviewer: In regards to your injury, on the online questionnaire you mentioned that you had a little bit of fear associated with the injury. Do you want to tell me a little bit about that?

P5: It was probably the worst coming back into sports. After I had done all my rehab and stuff and we're starting to actually play games again, if people came out from somewhere that I wasn't expecting or frightened me or just popping up or getting too close, I actually started having what I could call panic attacks, like hyperventilating and just shutting down.

I would always have to take myself off the field and just sit until I settled down again. That happened probably three or four times, but each time it got shorter and shorter for how long it would go. I don't get them any more, but in the beginning, it was very fresh and obviously, I was just freaking out because I was like, "Oh my God. I can't let this happen again." [chuckles]

Interviewer: You were freaking out. How were you feeling leading onto these panic attacks?

P5: It would always just be fine. I was obviously nervous coming back into playing sport, but it was mostly just people bumping into me. If I was running straight ahead, someone coming from behind me where I wasn't-- I didn't know they were there and cutting in front of me, because I am thinking, "If I'd just been a step further forward, they could've taken me out."

It was just very instantaneous. The event would happen and then I would get a panic attack and I would start freaking out because I was like, "What if I had been hit or something my knee would just go again or something like that." But it was never just-- On the field playing, I would be fine. It was just when those specific circumstances occurred that I would freak out.

Interviewer: freaking out and the panic attacks, how did you get over that? How did you deal with these issues?

P5: I would normally sit through them. People would just come over and pat me on the back, give me some water and typically just sit down, hyperventilate for a little bit. I was getting used to it and reminding myself, that it's not always going to happen.

It was an accident the first time it happened and I was irresponsible afterwards, but you don't do your ACL every day. There is a reason why you don't, like they work. Just trying to remind myself that it's not going to happen on the regular.

Interviewer: What was the biggest thing that you were really worried about or thinking about that would lead to those episodes?

P5: I don't think it was really anything that I was thinking about, it was literally just in-game. People would come up and I'd just get scared and I would be like, "Oh my God." It wasn't a cycle of thinking or anything like that. I wasn't playing the game and thinking, "Oh my God. What if something happens."

It would just be playing and then something would happen and I would freak out. [chuckles] Does that make sense?

Interviewer: Yes. What were you scared of or freaking out about? What would it mean to you if say--

P5: I didn't have a great time when I was in the splint, I pretty much was just at home in bed for that whole month and couldn't walk around much. That was very miserable. I was like, "I would really hate to have to do all that again" and the pain. I couldn't take anti-inflammatories or anything like that, so it was quite painful. Waking up at night and just being in pain, things like that. I was like, "I don't want to do that again."

Interviewer: How would you describe these feelings of being miserable?

P5: How would I describe the feelings of just being miserable?

Interviewer: Yes. What was being miserable? What kind of thoughts and feelings were you having during that time?

P5: It was pretty lonely. I didn't have any friends over or anything like that. It was hard to interact with people. It was really annoying not being able to go anywhere. Just being stuck in the same room, in my house. My house has got stairs and so I couldn't climb them, so I was just stuck in my bedroom.

It was very isolating and it's just so monotonous. You don't feel anything and that sucks. There's no really happiness, you're just like, "You're going to be miserable because you are in pain." You're having to do stuff that you wouldn't normally have to do. I'd never been injured before like that so it was all very new to me and so it sucks just having to deal with it.

07:30

Interviewer: How did you deal with, the emotional side of those feelings at that initial phase of rehab?

P5: My partner definitely helped a lot. He visited quite a lot, but I don't think I did my initial rehab very well. When you are at home in the splint and you've got to do those sorts of exercises, I don't think I did those very well because I just didn't have any motivation for it.

Life just really sucked and so I wasn't really prepared to do anything about it because I was like these also suck. This isn't going to help me right now. It was only later once I could start walking around my Frisbee team gave me an assistant coach position or a training coach position and so that meant I could start going down to trainings and watching and try help coach and learn things from that perspective.

I think that that's when my rehab and getting back into sport really picked up. I dont think I did much in that first initial phase at all.

Interviewer: During that first initial phase it sounds like it was difficult.

P5: I'm sorry. I can't hear you.

Interviewer: Sorry. Can you hear me or is that not working? No luck?

P5: I'll grab some headphones.

Interviewer: Yes.

[pause 00:09:16]

P5: Try now?

Interviewer: Yes. Can you can hear you now? Awesome. Yes, so back to that initial phase, prior to your injury, did you have any fear or was it just stress to do with injuring your knee? When you first did it, did How did you feel after that initial injury? Were you experiencing fear or distress?

P5: I was definitely worried and I would say distressed. It hurt a lot afterwards. I think it was blown up and I think I was not really able to walk much the next day and maybe the day after that as well.

That was pretty shocking but it's just like the first time that I'd been playing sport seriously, so I didn't know much about getting injured or gym programs and how to stop that. I was worried and I did go to a physio and ask for their opinion, but I just really wanted to play and so I just kind of pushed aside. [chuckles]

Interviewer: How would you describe or what did the worry mean to you? How would you elaborate on what that word, what it meant to you?

P5: It was very unknown. I didn't really understand the consequences of an injury, especially one like this. I was worried because people talked about how serious injuries could be and they were also obviously worried like other people had talked about it and been like, "It sucks."

I don't think I was worried mostly because of that and they're talking about it and how they had obviously said that rehab is hard work and things, but I didn't really understand it. When you say initial phase, is this right after I first injured or are you talking about after surgery?

Interviewer: Right after you were first injured. Does the effect of surgery, how did you feel about the process of the surgery and what kind of thoughts and emotions were you having during that period?

P5: It was just very much something that I had to do like going into it. I wasn't really that worried and I wasn't stressed out or freaking out that I had to get surgery or anything like that

because I had to get it fixed. There is no if or buts here. You have to get it fixed and coming out of it, I think I was not worried about the actual injury anymore. It was just the emotions of having had surgery now just being stuck at home.

Interviewer: Yes. What were those emotions?

P5: Just a bit miserable and lonely. [chuckles]

Interviewer: You also mentioned the pain. What did the pain mean to you or what was the effect that the pain was having throughout this experience?

P5: I think it was just very, very frustrating. Especially waking up at night and not knowing why you've woken up. Just being not panicked, but you know something is wrong and you've got to wake up and try figure that out.

When you're sleepy, it's so difficult to pinpoint what has happened or why you're awake. That was probably the worst part and not looking forward to that happening again. In a night or two. Apart from that, the pain was pretty manageable.

Interviewer: Thinking about the fear of the re-injury now, what would be your biggest emotional concerns or feelings about that?

P5: Knowing that I'm not prepared, obviously cause COVID happened. All the gyms shut and no sport was really happening, especially here in Melbourne and coming back into sport now that everything is opened, I know that my body is not prepared yet.

I'm still playing sport and it's niggled, it's been in a bit of pain and stuff, so I've gone and seen the physio and they've given me more rehab, but Knowing at the moment that if I do push too hard, it could just not be as stable as it should be.

Interviewer: How do you feel about that?

P5: It's not great. I feel like I have a better understanding of when to stop pushing it than I did before. I'm more willing to not push it. I would just go, "Cool, I'm not going to play anymore." It doesn't really worry me as much when I'm playing or when I'm doing things, but when It starts playing up I'm a bit worried about it and I'll typically just go see a physio. No matter what. [chuckles]

Interviewer: You mentioned you worry a lot. Are you fearful at all when you're looking back at going back to sport now?

P5: A little bit, but that's also why I've gone and seen a physio, I've got a gym program. That's all really amped up quite quickly. Once gyms opened it was like cool, back.

Interviewer: Would you say in this return to sport phase have got any distress associated with your knee?

P5: It is always in the back of my mind, especially when it does start niggling or if we have had a longer session. I can feel that the muscles around it are tired. It does play on my mind a bit. I'm a bit of one-track-minded person. Once I am on the field and we're playing a game, it's out. I don't think about it at all until I get back onto the sideline and I've got a chance to think like, "Oh, it's feeling all right."

Interviewer: How has your injury affected your confidence in this return to sport?

P5: It's definitely made me more cautious when coming back. I'm less willing to.. if I don't like my cleats, I'll just get new ones. I don't want to mess around with things that I can control. If I don't-- That's mostly it. My cleats. My pretty boots. If I don't like how they're

fitting anymore or something like that, I'll just get new ones and fix that problem that I can control. Yes, it does make me motivated to go to the gym. Make sure it doesn't happen again.

Interviewer: Can you improve your confidence or reduce your worry in any other ways or it's just mainly the cleats and the gym program?

P5: I'm always going to worry about it if it's sore. I don't think I've ever had a season where I've not had an instance where I've been like, "Maybe I shouldn't do this drill or maybe I shouldn't do this." Just because it's feeling a bit funny.

Interviewer: Feeling a bit funny?

P5: Sometimes if I just turn, not specifically, but every now and again you will have a turn where you've just done it badly. You be like, "Oh, I shouldn't have done that."[chuckles] I'll be like, "Cool, I'll just not do." I'll just like let it-- I'll leave it alone for now. I won't push this.

Interviewer: Now just looking at the experience from you know, you said you were seeing a physio. Has the physio chatted with you about any psychological aspects of the injury at all at the end-stage? Has any healthcare practitioner at all talked with you about any psychological considerations to do with the injury, throughout the entire process?

P5: I don't remember.

Interviewer: What do think about the psychological aspects of the whole ACL injury experience?

P5: I think at the beginning, once I first had that first panic attack, I very much understood myself. I was like, "Yes, this makes a lot of sense. I'm freaking out because I've just had this kind of experience that I really don't want to do again." For me, it made sense which I think

made it a bit easier because I could then self-talk and go, "You don't have to worry about this. You're doing what you need to do to make sure it doesn't happen again."

I think it would have been nice to have been more prepared for that, but it's hard when there is something wrong with your body. You know that you have to take care of it but knowing that that's going to happen for the rest of your life is a bit hard to prepare for and I don't think there's any good way to prepare for it though.

Interviewer: Do you believe that this is going to happen for the rest of your life, where do you think that message has mainly come from?

P5: Because it's still giving me trouble. It's come to a point in my sporting where I don't need to worry about it, when you're doing all the right things, you're going to the gym, you're doing all the things, but I think if you're going to continue playing sport, you're going to have to keep doing those things to not worry about it, but that's part of worrying about it.

If you're going to go to the gym to make sure these things don't happen, you're doing that because you're worrying about it, you can't do them automatically, and that's what it's turned into, but you just have to keep managing the problem.

Interviewer: Long-term, yes. You've mentioned it's worried you. During your rehabilitation process maybe three or four months, as you've moved on off the crutches and you're progressing. How were you feeling at that stage, did you have any fear?

P5: I wasn't playing any sport or anything like that, I was just going to the gym and walking around at the training. I think that I was definitely helped by the fact that I was coaching.

There was no in for me to actually participate in any of the trainings, and I wasn't going to go find other places to participate.

It was really walking around and then gym. I had no worry about that because gym was going well, and I knew that wouldn't be able to hurt me.

Interviewer: Any distress or anything else, thinking about outcomes or anything during your rehabilitation process, or did it mostly come later?

P5: It's mostly a little bit later, once I'd started playing again.

Interviewer: Does that include around that nine to 12 months, were you pretty non-fearful then, or when did you start to return to sport and get those challenging emotional aspects?

P5: Long time ago. I think I was just running at that stage, just a straight line and not really turning or anything like that, or maybe starting to turn in a controlled manner. It's not fear unless it's those in-game shocking moments, it's always just a bit of worry.

I was still definitely worried about it, and I was always afraid I was going to push myself too hard, but at the same time, I was worried that I wasn't putting in enough, and then I wouldn't be prepared for when I actually went back to sport.

Interviewer: When you went back to sport did you feel that all throughout this injury process, I should say, did you feel that your perception of yourself has changed because of the injury and the process?

P5: I don't think my perception of myself has changed. I think of myself as the same person and things like that, at least in the way that I think about certain things during sport, and going to the gym has changed.

Interviewer: You mentioned that during sport that you were okay most of the time. When do those thoughts, is it mostly afterwards, or when are you getting those thoughts?

P5: During breaks. We do a drill and then we go and get water or something, or we'll be playing a game and then you'll have a point off, and so it's during those times where it's thinking, "Oh, my legs are definitely getting a bit tired."

You can feel the fatigue around the knee, those times. Again, I don't think of myself differently, it's just-- I don't know how to describe this. [chuckles] I guess I think of myself as not attached to my body, my knee is an issue but I don't think of myself as any different, if that makes sense.

Interviewer: Yes, it does. How do you view your knee then if it's not part of yourself?

P5: I know it's trying its best, it's obviously not the best knee in the world but it's trying its best, and I'm trying to give it the opportunity to perform again.

25:56

Interviewer: When you say words like, "It's not the best." What are your thoughts around your knee? How do you view your knee? Are you viewing it any different than the other?

P5: Yes, because my other one's never given me any trouble, and obviously this whole leg gives me issues now, because of the ACL injury. It's a source of instability, which has given me ankle injuries and other things that I have had to deal with now as well.

Just in general, muscle tightness and stuff is more prominent on this leg and so it's annoying. [chuckles] I do get frustrated at it but there's not a lot that I can do. I just have to deal with it.

Interviewer: Do you think there is anything you could do, do you think your confidence, or your confidence in the ability of your knee to function will improve at any stage?

P5: Yes, but I think it's a constant thing. Once I've built back all my muscle mass and I'm back to normal on both legs and stuff like that, I think I'll be a bit more confident in it and the maintenance will be less.

It'll be more capable of dealing with the load I'm putting through it. Once you stop putting an effort like going to the gym and rolling out, and stuff, I think it's just going to degrade back to being worse, than my leg if I hadn't had an ACL injury.

Interviewer: How do you feel about the future then, and that thought that it's going to be potentially worse than the other leg?

P5: It's definitely frustrating. I think it's just mostly going to be if I'm playing sports. I didn't notice it at all when we were in lockdown, and you couldn't play any sport or anything like that, but when you're playing sport, it's different, you have to put in the effort. Did that answer the question?

Interviewer: It's all right. Frustration, you've mentioned that a few times as well. What kind of things do you mean by frustration in regards to this knee?

P5: It is very easy to just go like, "I wish it had never happened." It's frustrating having to spend money to go to the physio all the time, to put my worries at ease. It gets frustrating having to put in the extra effort to do all this single-leg stuff at the gym to make sure that they're even and I think you'd take a little bit less care with rolling out if this hadn't happened, but because it has I need to make sure I put in the effort, and sometimes that's hard.

Sometimes you just don't want to. [chuckles] That's what you're stuck with now. It's happened, you have to deal with it even if it's frustrating.

Interviewer: How could you deal with it to improve, maybe reduce some of this frustration and distress and other things? Have you developed any other dealing strategies beyond the training?

[silence]

P5: Probably not. I just really like my sport. That's obviously a big driver. I do these things because I just love playing the sport. That definitely-- When you have good days with bad, it definitely takes a lot of the load off. I find myself a lot more willing to do what I need to do to keep playing. I do notice myself being hard on myself with negative talk so just trying to catch that early and be like "No you're doing okay." [laughs]

Interviewer: What kind of stuff is that, the negative talk? What kind of thoughts and what does that comprise of?

P5: If you miss a gym session thinking "It's just going to the gym. Why can't you do it? It's not that hard." It's just like, "Well you're having a hard day at work or something." Making sure you're not too hard on yourself, but also trying to keep yourself accountable in a positive way or using mechanisms like going to the gym with someone else to try and keep you on track.

Just if you haven't put in the effort earlier just not getting-- Once you get into that negative cycle of like, "You suck because you didn't roll out." or something like that and like, "Now

you're in pain” haha." It sucks. Trying to make sure you break that cycle and be like "Oh okay, you didn't roll out but you can roll out now." Then help the issue.

31:50

Interviewer: Obviously, there's a bit of stuff talk that is just going on there. What kind of effect has the knee injury had on your overall well-being?

[silence]

P5: I don't think it's had that much of an impact because I'd probably still be going to the gym regardless.

[silence]

P5: I think it's just a little bit of worry when I'm doing sport and if it's getting sore and stuff like that. I think that's the only real impact that it's had on me now.

Interviewer: Have anyone else's perceptions of you changed because of the injury?

P5: I don't think so.

Interviewer: I guess with all that worrying and everything else, obviously you're playing sport, you're getting through it now. Is there anything else, how would you respond when faced with a fearful movement at this point in time?

P5: At this point in time because I'm not entirely prepared for it, I haven't got my gym back to where it was before COVID, so I think I'd be a bit cautious of it especially if it's very turning orientated, but I think I would still attempt it in some form. It might just be slower or I'd just be thinking about it a lot rather than just diving straight in to try and make sure it's controlled.

Time: 33 min 59 seconds
