# Supplementary material for: Understanding Fear after an Anterior Cruciate Ligament Injury: A Qualitative Thematic Analysis Using the Common-Sense Model
Source: Int J Environ Res Public Health. 2023 Feb 7;20(4):2920. doi: 10.3390/ijerph20042920 (PMC9957354; doi:10.3390/ijerph20042920)
Supplement: Supplementary file 1 [file ijerph-20-02920-s001.zip › File S1-P6 Transcript.pdf]

<u>P6 Transcript</u>

Interviewer: I'll just start that one, start that one, and where's record? All right. Great. So, thanks for spending the time with me, P6. So today you'll be participating in a study that is researching fear post-ACL injuries. This interview will consist of a series of open-ended questions that will explore your fear, your levels of stress, and your confidence after your injury. Please feel free to say as much or as little as you want on any topic and if you feel uncomfortable any time let me know and we will move on. That sound okay?

P6: Yes. Sounds good

Interviewer: So tell me about your ACL injury.

P6: I've done my left knee twice. The first time, I was skiing and I had a pair of demo skis on. The bindings weren't calibrated to my weight appropriately so I fell over and the ski didn't come off so my knee went instead. Relatively quickly got operated on and they put in the lars ligament. Yes, that was all good, did the rehab stuff, got back to sport, got back to skiing. Then about five years later, I just landed a bit weird at netball training and the ligament went, again.

Obviously, five years before that I didn't really know that Lars ligaments only have a five-year life span. I went with the hamstring reconstruction, for the second time. I still ski but I've decided netball is not worth it.

Interviewer: In regards to your ACL injury, you mentioned in that questionnaire that you filled out online that you had a bit of fear associated with the injury. Do you want to tell me what this fear is?

P6: Yes. I'd definitely pick and choose what activity I do. Now that I've done it twice, I'm pretty cautious, that's definitely a cost-benefit sums happen in my head when I decide to participate in different things. I think the benefit out of going skiing because it's my favorite thing in the world. That's totally worth it. I'll make sure I wear a knee brace and all of that stuff to make sure I don't do it again.

I definitely ski a bit more cautious than I would have if I didn't have my knee done twice, I don't think the enjoyment I get out of netball's worth it so I've decided not to go back to netball and probably would think pretty hard if I wanted to play tennis, which is not something I normally do, but yes, sort of picking different sports based on that. Even just having a kick of a footie or go to a barbecue or something, I'd be a bit more cautious and less likely to participate.

Interviewer: You've mentioned 'cautious' three times already. Do you want to just tell me about what this word, 'cautious', means to you in regards to your knee?

P6: Yes, okay. So I guess I just am more thoughtful of different activities such as side to side movement, and that sort of thing, I wouldn't think about it. Whereas if I think if I hadn't done my knee, you just would never think about it. You would just be like, "Oh yes, whatever, I'm just doing my thing." Whereas I'm like, "Whoa, should I do that? Should I not?" So that's what I mean.

Interviewer: So what's the effect? What's this cost-benefit or the effect in your mind of doing these movements or this cost-benefit relationship? What are we weighing up? What are the thoughts and feelings around that?

P6: I guess like is this something that I would be willing to do to my knee again? Would I be willing to do my knee again for an awesome day of skiing? Probably. Running around doing something silly at a barbecue? Definitely not. That is how I would picture it. I don't think that I would get my knee reconstructed a third time. Although a still have a patella to reconstruct with

[laughter]

Interviewer: Is this something that is constantly, you mention you would weigh it up, is this something that you think about a lot?

P6: Probably not because I'm not playing netball. I've had a friend ask to fill in for a team, and I'm always like, "No. I don't think I want to." I don't think I've rehabbed myself enough after the second ACL to be back to that level. More so, out of laziness and not wanting-- probably that I’ve said I'm not going back to netball so I'm not pushing myself to get back there. Yes, I think I'm a better skier than I am a netball player so I'm more confident in my abilities to ski, probably not fall over.

Interviewer: Okay, so what would it mean to you in this cost-benefit if you were to have an issue?

P6: So I guess, it would be painful, and I work as a midwife so I’m on my feet all the time at work, so not just like an office job where I can sit down and still doing my work like normal

like it's not much time off. It's very hard. Everything. When I had another reco or not, that's definitely something that when It would be a bit of a financial thing as well. I guess it's just an annoying thing to do. [chuckles]

Interviewer: What is this word, 'annoying', mean to you in regards to your knee?

P6: Getting around in crutches is a nightmare, you can't carry food and walk at the same time. You just got to put all those hours at the gym, spend lots of money at physios. Yes, all that kind of stuff.

Interviewer: How do you feel about all that kind of stuff? The rehab and then the money and stuff you've mentioned?

P6: Well, I have a very expensive left knee. I don't want to make it more expensive than it currently is. I think I've spent enough money on it. Yes, to me it's a lot of effort. This probably would be good for my fitness to have to do it all again but I don't want to have to do it all again.

Interviewer: How you feel about the prospect of doing it all again?

P6: I'd be disappointed I think, that I was doing it all over again and I think that I would just conservatively manage a new injury and just do the rehab rather than the surgery and the rehab.

Interviewer: Going back to that initial injury, prior to your initial skiing injury, did you have any fear associated with the knee?

P6: No, not at all. It was always something that you would-- I'd thought of lots of other people that had had knee injuries. Like, "Oh, you don't want to do that." Yes, definitely it's

pretty-- I was generally a relatively cautious skier anyway. I don't like falling over it's not fun, I'm not someone that was just going to throw themselves off cliffs and do all that sort of stuff but it wouldn't faze me if I did. So definitely I would be a bit more aggressive skiing and whenever I think about playing netball and sort of thing. So I don't think there was really any fear.

Interviewer: So then after the initial injury, how would you describe your feelings?

P6: I think I would be more fearful afterwards, after the first injury. Definitely, I started doing a bit of risk mitigation, in terms of maintenance, I'd rehab pretty well. I worse a knee brace if I was doing anything that I thought would involve lateral movements and that sort of thing so that I could not worry as much, using those sorts of things to best reduce my fear of doing it again and just make me sure that I wasn't going to hurt myself again.

Interviewer: So at that initial injury though, right at the start when you first did your knee, what were your feelings there as well?

P6: I fell over and felt it go and I went, "Oh no, I'm going to be one of those people with knee problems," was the very first thing that I thought and I was like, "Oh, shit." That didn't feel good. Yes, I was really annoyed, pretty upset that I had done that. I immediately knew that there was going to be the consequences from that I thought oh bugger why did I do that.

10:18

Interviewer: Yes, and how did you feel about those consequences, though?

P6: I never really thought about them much straightaway. I was just like, "Oh, yes, cool, let's do it. Let's get it done." I was at uni at the time, so it didn't really affect my ability to go out and play sport. It didn't really affect me too much. Definitely still went to the pub and did all of those sorts of things. It was just maybe a nuisance, but.

Interviewer: When you had surgery, and how was that whole, what were your thoughts? What was your thoughts on that whole procedure? I mean, you've been through two now.

P6: The first surgery, it was pretty okay.

Interviewer: How did you feel about it?

P6: It was painful and I got frustrated at not being able to do as many things and being a bit more reliant on people. Yes, it's just frustrating. Not having that same level of independence and movement and having to think about how you're going to do things or how you're going to carry a plate and that stuff was annoying. I wasn't too scared of the procedure, or anything like that. I'm a medical-ish background, I was studying science, doing anatomy, all of that stuff at the time, so I've got a pretty good understanding of what happens in the procedure and found it interesting. That academically was pretty cool. The surgery was not the issue, it was more the recovery.

Interviewer: Yes, so during the recovery then, when you began to go through recovery and resuming normal activities, were you experiencing, what were you experiencing then? Were you experiencing any fear?

P6: Not so much. Just fear that I would hurt myself if I wasn't careful with what I was doing in terms of movement. I've probably enjoyed, I think actually during my ACL was a pretty

good thing for me the first time because I would come out of school, and be pretty active and sporty and got to uni and I was like, "Cool, let's go to the pub." and wasn't as active and then when I did my knee, I had to get back and be active again. It was a really good thing for me to actually get back into sports. When I could run, that was really great.

I'd never been someone that enjoyed running before that and then all of a sudden you can't do something and then when I was able to run again, I was like, "This is so cool." I started actually enjoying running. It was one of the better things that had happened. The consequences of the first injury was really really positive actually. It forced me to get active and get back to sport and enjoy that sort of stuff because probably if I hadn't have hurt myself, definitely could have just ended up becoming more unfit and then not gone to that effort, to actually being fit.

Interviewer: Just enjoyed the pub that much more.

P6: I know it, yes. [laughs] I still did.

Interviewer: Were you distressed at any time during the initial phase through your ACL injury journey?

P6: Sorry, did you say depressed?

Interviewer: Distressed.

P6: Stressed. Not so much the first time. I think maybe after the second one I was a little bit more stressful, I was getting a bit more independent, in terms of my life as I had started working as a nurse so I had to take a lot of time off. I guess I was a bit more stressed and frustrated at being reliant, and not being able to move my knee again. When I did my knee

the second time, because that was just the lars ligament that went, I actually really didn't get any pain in between the first, from doing it to getting the operation. That period wasn't painful.

I just had less stability. I remember waking up from surgery, and it hurt and I was just like, "Oh, why did I do this?" This is really painful now. That was really annoying. I second-guessed why I got the surgery done the second time.

Interviewer: Yes, so that word, that stress, how would you describe or what does that stress mean to you?

P6: Stress. I guess that's when you can't process your emotions properly. If you're not in that headspace where you're able to think as clearly, more likely to do silly things. I was, definitely, after the second one more likely to snap at people. I was just a lot more frustratable. I'm usually a pretty easygoing person and I don't think I was always as easygoing after the second one. Probably the first one as well, just when you're not able to move around as much. It's just really frustrating, and your tether is just not quite as long as it used to be.

Interviewer: Yes. Did you experience any other changes to your emotional well being during this process?

P6: Probably.

Interviewer: In what ways?

P6: Things bothered me more. Yes, I don't know. [chuckles]

Interviewer: What about changes to your overall well being? Do you think these injuries--what was the effect of your overall well-being of these injuries?

P6: Probably neutral, I would say. It has inhibited my mobility, but then also made me work more to my mobility and get enjoyment out of doing all of that hard work and seeing the results and being more mobile, but then also reducing enjoyment because you had to think more about doing things. I'd say it's pretty neutral overall.

Interviewer: I know we touched on it before but you're going back to sports, maybe around that 9-month to 12-month end of rehab cycle, how were you feeling during that 9 to 12 months?

P6: The first time I was pretty optimistic. I was feeling pretty good, rehabbed pretty well, not sustaining because I did the lars ligament, not sustaining that hamstring injury-- Was really good. I was feeling pretty good. I started running-

Interviewer: Were you having much fear--

P6: What was that?

Interviewer: Did you have any fear associated with the knee during that period?

P6: Yes. Probably again, feeling quite cautious. Not fully trusting my body.

Interviewer: What does not fully trusting your body? What's the manifestation of that?

P6: Not trusting that my muscles were strong enough to support my new ligament. Yes, not having full faith in the work that I'd put in or not put in. That's probably what I mean by that.

Interviewer: How did you feel about lacking some of that, for want of a better word, the confidence or did you feel like you have reduced confidence or faith in your knee during this period.

P6: Yes, I definitely had reduced faith in my knee. I guess that's an annoying thing to have. I don't really like it much. I probably didn't put too much thought into it at the time really.

Interviewer: Maybe what about earlier on during the rehab process, how was your faith in your knee throughout the beginning to the end of the rehab?

P6: It was just guided by what I was allowed to do, faith that I could do straight-lined stuff and then quite timidly start to move into the more side to side. Direction change kind of movements and things that you're cautious and you test it. As you are able to do more, you gain a bit more faith. I've probably got back to that level where I did have more faith in my knee.

I got back into all of the sport, got back to skiing, got back to doing all of those things. Pretty much trusting my knee. Probably 90% trust in it. I'd still wear a knee brace, but it felt like that was less and less necessary as I got along. As I was getting back, we were at that five-year mark before I injured it again, I would say I almost had full trust back in it.

Interviewer: Did you have any strategies for managing the psychological aspects of your knee, like fear or distress?

P6: Not really. I guess just putting in those practical things of if I wear my knee brace and I don't do anything too silly, it will be okay. I'm not too much of an anxious person. Didn't really bother me. I didn't put in any other thoughtful things. I naturally did stuff to make it a bit more manageable probably, but I didn't actually intentionally use any strategies to get there.

Interviewer: In the current time, how fearful are you about your knee?

P6: Yes. Currently, I'm much more fearful after the second one. I think because I haven't returned back to that level of sport. Really I have less time to put into the rehab. I've got back to being able to functionally do everything. I've focused on doing more running and that sort of stuff, but I haven't focused too much on going back to those lateral activities. I've given up netball but I've taken up rowing instead where you actually can't do your knee rowing. Just changed my activities. I'm definitely more fearful at the moment and conscious of doing something.

Interviewer: How would you describe this fearfulness or the fear now?

P6: It's not there all the time. It's probably just if I was getting myself into a situation where I'm like, "Oh, should I be doing this? Do I want to go out and play tennis that I haven't played for years? Is that something that's going to be worthwhile or silly?" It's not there all the time. It's not something that affects my mental health day-to-day. I'm just a bit picky about what I do.

Interviewer: What does affect your mental health then?

P6: I've generally got pretty good mental health. It's just things at work sometimes get a bit stressful and I feel a bit burnt out from work and that sort of thing. That probably affects me the most. Generally, my mental health is really really good.

Interviewer: In regards to your knee, nothing has been-- you haven't noticed any issues throughout your ACL journey?

P6: No. Not really. No. I wish I could just go and play netball. Probably could. I'm probably just stopping myself.

Interviewer: You've said the cost benefit relationship in terms of netball. How do you feel about the prospect of going back to netball, or is just not an appropriate when you weigh it out? It's just not.

P6: Yes. I don't think it's appropriate. I don't know. I love skiing, so I think that is appropriate. I'll risk it for skiing. Netball, I think it just doesn't really fit probably with my life at the moment. Doing shift work and that sort of thing. It's hard to commit that time anyway and so I don't think, "Oh, the knee's not really up to it anymore." I think it's something that I've said goodbye to, and it is fun. I would love to be able to answer my friends' text of like, "Hey we need an extra player." I'd be like "Cool. Yes. I'll come and play." Probably.

Interviewer: Do you think your knee function could improve?

P6: Probably, but really I've got back into going to the gym regularly and decided to be more active. I don't know. I feel like I've kept in pace with my activity levels and how much I like to do. If I got really back into being active and going to the gym all the time, then probably my function would improve. I'd have more confidence in the muscles around my leg to be able to support it twice rehabed and operated on leg, all the other muscles, I think that's the most annoying thing about it is that all of those other muscles are really really good beforehand. Then it's amazing in that week period of being inactive, how much you loose those muscles. That wastage is just so incredible and annoying you had those perfectly good things and then you stop using them for just that little bit of time, and then all of a sudden, you've got so much work to go back.

Interviewer: How do you view your knee now? You've just mentioned the twice-rehab knee or my injured knee, I think you said before. How do you view your knee?

P6: It's my bodgie knee. [laughs] I put a lot more thought into my left knee than I do my right knee. That's a bad knee. It's definitely got negative aspects to that rather than my right leg's which is the the strong one. The left one's definitely a bit weaker.

Interviewer: Throughout your experience, through your ACL injured knees, has any healthcare professionals asked you about any of the psychological considerations from the injury?

P6: I don't think no. No. Probably not. I'm trying to think. Maybe I think when I was rehabbing in between the two injuries. There was one of the physios had a bit more of that holistic wellness aspects and was thinking more about visualization in terms of getting back to sport and improving my abilities as a netballer and that sort of stuff. It's probably a little bit more mentally involved, but yes. I don't think so, but then I don't think I was someone that was having too many psychological things going on. Really I was coping pretty well.

Interviewer: Now, with your knee, you have already kind of mentioned that you don't want to expose yourself that cost-benefit relationship, but how do you respond in general to fearful movements or activities?

P6: Yes. I'm just really mindful of wherever I'm putting my feet in and how I'm moving. Yes, I'm more cautious. Probably if I went hiking, you're paying more attention to how you're moving whereas if I'd not done my knee, I'd probably be less mindful of how I'm moving my feet.

Interviewer: Mindful, what do you mean by mindful?

P6: I think just picking and choosing where you're putting your feet and that sort of thing, focusing on the movement rather than just letting your body do its thing and thinking about other things.

Interviewer: How do you feel about that, the focus if you're doing an activity or your attention going to your knee?

P6: Well, I would like to not have to think about it. It doesn't bother me too much but guess a little bit of, you do have to think?

Interviewer: Yes. okay.

P6: Yes, I'm probably used to it now, I did my first knee reconstruction was in 2011. The second one was in 2016, '15. Actually 2016 it would have been. It's been a while of having to think about what you're doing, so I'm pretty used to it. I don't really think about the fact that I'm thinking about it.

Interviewer: Do you ever won't think about it?

P6: Pardon.

Interviewer: Do you think you ever won't think about it?

P6: Probably not.

Interviewer: How does that make you feel?

P6: Disappointed that you suck at one little thing. It does have lifelong consequences for your movement, you definitely worry when I'm older, needing new replacements and that sort of thing. I'm sure that I'd be more likely to then had I not hurt myself initially. Yes, annoying.

Interviewer: Do you think with all the annoying, and all that, do you think your perception of yourself has changed because of your knee injury experiences?

P6: Probably positively and negatively. I should be proud of myself in terms of how I have rehabbed and worked really hard. If I hadn't done my knee problem, I wouldn't have needed to and you don't have that reward from having done all of that stuff, makes you feel a little bit like you've already failed you the first time. Although both knees, the first I would have been able to blame it on equipment, whether it be the skis the first time or the lars the second time. I don't think it was my body that really let me down, so I guess that's probably been helpful to have something else to blame.

Interviewer: Yes, so throughout that, do you think then other people's perception of you has changed because of the ACL injuries?

P6: Well, yes. People, my friends definitely remember all the times that I've hurt myself with my knee and probably a little bit of a defining feature of me in some people's memories in that time. I hope they're also proud of me for having gotten back in-- Probably it has but not in a way that bothers me.

Interviewer: Now, after all this, what is the biggest fear to do with your knee?

P6: Probably that it's not going to last, and that one day I will end injure myself some way or another. That I might have to go through all of that again. Yes, that's probably my biggest fear is that this one won't last either. I don't know, I worry that one day I've got kids back that I might actually be more thoughtful about chasing them around and all that sort of stuff, not something you'd like to have to worry about, but probably will. I think a lot of the ongoing, not so good.

P6

Time: 36 minutes 30 seconds

Audio file name: P6.m4a
