# Supplementary material for: Understanding Fear after an Anterior Cruciate Ligament Injury: A Qualitative Thematic Analysis Using the Common-Sense Model
Source: Int J Environ Res Public Health. 2023 Feb 7;20(4):2920. doi: 10.3390/ijerph20042920 (PMC9957354; doi:10.3390/ijerph20042920)
Supplement: Supplementary file 1 [file ijerph-20-02920-s001.zip › File S1-P7 Transcript.pdf]

P7 Transcript

Interviewer: Going on. Turn this one on and turn this one on. Awesome. Hi, P7. Thanks for taking the time.

P7: No worries.

Interviewer: Today you will be participating in a study that's researching fear post-ACL injury. The interview will consist of a series of open-ended questions that will explore your fear, levels of distress, and your confidence after your injury. Please feel free to say as much or as little as you want on any topic and if you feel uncomfortable at any time, just let me know and we'll move on.

P7: No worries.

Interviewer: All right. Tell me about your ACL injury.

P7: I had a full rupture of my ACL and a small tear in my medial meniscus as a result of a mixed netball injury. Mixed social netball injury. It was the first real major injury that I've had on my knee. I've had just strains and things of that nature before but nothing where I've been out of action for multiple weeks or months or needed any surgery for. That was the first injury on my knee. That was in July 2017.

Interviewer: In regards to that injury, you mentioned on that questionnaire that you had a bit of fear associated with the injury, do you want to tell me a bit about that?

P7: I guess, when you tear your ACL and you go through surgery and do your rehab, there's always the fear that you might injure it again. Luckily enough, I had a great physio team and everything like that. They put me through the test from a third party, a sports scientist to test my knees again, so I know that it's good to go and I've played sport again since then, but I guess you always have the fear of re-injuring it because you don't want to have to go through the same process and the pain with the surgery and rehab again. That's probably where my fear aspect is, not wanting to go through the whole process again.

Interviewer: What would going through the whole process again mean to you?

P7: It would be having the surgery, having multiple weeks and even months where you are dependent on other people whether it be driving to work or helping you around the house, just with daily chores, and just the dedicated rehab because if you don't do it properly you just leave yourself at risk to re-injury.

Interviewer: Do you want to tell me a bit about what was happening with people helping you and how you were feeling about that during the initial injury?

P7: I initially was going to physio post-surgery three times a week for the first three weeks, just for general exercise and mobilization. Then that dropped to two times a week probably for three to four months. Then once a week for, I'd say, another four months, which it was more just helping me a lot with my flexibility, getting my hamstring strength back up again because I had a hamstring graft. They were really good in giving me good at-home exercises. Also giving me really clear goals and targets about when I could start running again, when I could play sport again, things that I should try and avoid, that type of thing.

Interviewer: How important did you think that relationship between your health practitioner was for your outcome?

P7: If I didn't have a good relationship with my physios, I don't think I would have had as good of an outcome. I really felt like they cared and they had other people in their practice going through the same thing with similar timelines as me so it's good to have a bit of, not competition, but comparability with other people. I think the relationship is really-- it's vital. It has to be. If you've got no faith in the professionals trying to get you back to where you want to be and you have no self-determination because I mean you can get a little bit down when you get an injury of that nature and you become dependent on other people. The relationship is very important.

Interviewer: How were you feeling about the dependency on other people?

P7: I hated it, I hated it. I went from someone that is really autonomous in terms of just getting myself off to work, all that kind of stuff and not even thinking about like, "Do I want to go out to the pub? Do I want to go to the footie?" Something like that, it just became an extra level of thought that you had to put into. I had it on my right knee so I couldn't drive for a while. I lived 45 minutes away from work at the time so I had to get my girlfriend at the time to drive me to work every single day. There are things that play on your mind and you feel like you're a burden on other people. I guess once I started rehab and started getting more faith and more functionality in my knee, I guess my feelings probably-- It's, I guess, just like the stages of grief kind of thing. You go through the fact that you're a little bit down and then get to work and trying to make it all better.

Interviewer: How did you deal with being a little bit down? What kind of strategies did you find helped?

P7: I really threw myself into my rehab. I knew that was the thing that was, I guess, going to help my physical health but also my mental health as well . I was really dedicated, really threw myself into it, developed great relationships with my physios. That was probably the thing that was most important to me at the time.

Interviewer: Throughout the process, did your physio ask about your mental health?

P7: Yes. I was lucky, actually. I got referred to that physio from a mate of mine who did an ACL a couple of years prior. The first girl that I went to there before I had my surgery was really good in terms of diagnosis and next steps and all that kind of stuff. She, unfortunately, stopped working there about a month after my surgery but I tagged onto the list from another physio that was there and we realized that we had mutual friends and we went to the same school and he was just like, I think he's 15 years older than me. We had mutual friends, and we just bonded. We're still mates to this day. He was extremely caring about just the holistic approach in terms of what an injury can do to you in terms of how it may get you down mentally but also physically as well.

Interviewer: These feelings of getting down, what did you experience when you were getting down?

P7: When you're depending on other people you just feel a little bit hopeless, you feel like a bit of a burden sometimes. They were more the main feelings of feeling down. I never felt like, "There's no reason to live," or anything like that. It wasn't that dire but it was just like

having to ask someone to help you get up and go to the toilet or something like that. You just feel like a little bit of a burden. That's probably the main feeling in terms of feeling down.

Interviewer: After that initial injury that you mentioned, at the time of the initial injury, did you experience fear?

P7: When I had the initial? At the time of the injury?

Interviewer: That initial injury, yes. Did you experience fear in relation to the initial injury?

P7: As soon as it happened I knew what I'd done, which sucked. Then you start to think, "Christ, how is it going to affect all of these other aspects of my life?" It wasn't fear as in scared of what might happen, it was more like trepidation, like the fear of the unknown about what the journey was about to be. I think in terms of fear, it would be more of that because I hadn't gone through something like that before. I'd read up on athletes and a lot of athletes go through very public rehabs these days, so you have some kind of idea and understanding of what it's going to be like. I hadn't gone through something like that before so I guess it was the fear of the unknown. You don't know, I guess, what's to come on the journey. My physios and the doc and my surgeon was really good at outlining these are the stages and the roadmap of how we're going to get back and things that you may be concerned with and always available to answer questions.

Interviewer: What was the message you were getting when you were looking at that stuff about the other athletes and their experiences?

P7: I love sport, so I always stay in tune of who's getting quite major season-ending injuries and things like that. On the good end of the scale, there's the LARS surgery where they can

get back in just a few months and there's been really good evidence behind that. Then there's also, at the other end of the scale, there could be infections and things of that nature, from hospital, and it can take people maybe over two years just to get up and run again. I guess the fear of, and probably me just being a bit of a sports nerd is just like, "Okay, where am I going to fit in on that spectrum? I hope it's just going to be the normal one," and it absolutely was. All the timelines that my physios and doctor set out, I was able to achieve easily within the timelines easily.

Interviewer: Back to that initial injury, how did it affect your confidence at the time?

P7: It definitely negatively affected the confidence in my body but just my confidence in general, like being around people. Being around people was really good because I was just hanging out with my mates just like nothing had happened and confident in that way, but confidence in your body in that if I played something like this again, am I going to do the other knee or am I more susceptible to some kind of other injury? It would more be a confidence in the body thing rather than just a confidence, just a personality overall.

12:05

Interviewer: Do you believe that you are or were more susceptible to other things happening there?

P7: Having read some of the literature it's obvious that you are more susceptible to potentially doing an ACL on the other knee. I don't think that I'm more susceptible to major

injuries because I've done it mentally. I don't have pre-thought and think that, "Oh, I've done this injury, that automatically makes me more susceptible." Those kind of things don't go through my head. Just having spoken to the experts and reading the literature, obviously, there's a, not higher chance but there is some correlation and doing your first one. You might either re-tear or do your other leg as well. Correct me if I'm wrong.

Interviewer: No, there's definitely a lot of evidence around that. What about now? How has the return to sport gone?

P7: Return to sport was good. I got back to playing cricket, which is my main sport, which probably doesn't have as many key touchpoints in terms of ACL tears but there are a few there. My first game, I started off for a quick single and had to stop and put all my weight through my repaired knee but I had absolutely no pain going through there. I was able to stop immediately and that gave me real confidence to think, "Okay, no, I actually can go on and do this." I haven't played netball again but I am signed up for summer mixed comp again, pending COVID restrictions. I'd like to get back into that. Again, it hasn't stopped me from doing anything.

Interviewer: Throughout the process too, would you say you've had any distress associated with the knee injury?

P7: Just the pain after the surgery. That would be it. Potentially because I was being extremely active with my rehab and doing it three times a week, I was being very active at night, sleeping was difficult, being comfortable like in bed was sometimes difficult, and I didn't take much time off work either. That would be the only, I would think, distress. I had a pretty supportive network around me at the time. I think I was pretty lucky.

Interviewer: In terms of the pain, what was happening with the pain that you were experiencing?

P7: Just my whole right leg was in pain, just throbbing pain a lot. I wasn't taking very strong pain meds-- I was only taking panadol and ibuprofen. Getting my hamstring back to normal, I had extreme hamstring tightness following the graft. That was pretty painful. It was just like general soreness and waking up in the morning and your brain slowly rebooting and then all of a sudden the pain just hitting you which is one of the first things which is frustrating. That's slowly dissipated and I got my knee and the muscles around it more and more stronger.

Interviewer: During the rehabilitation process, as you moved away from the fear at the start, the initial injury, what kind of emotions were you experiencing during the process?

P7: I think it was probably at the nine week mark, I think, I had a huge breakthrough in terms of my rehab where I started doing a lot more strenuous exercise and I got real confidence out of that. I was really determined to keep it going. That was more type of the emotions that I had. I was really self-determined to just keep working really hard and putting in the effort. I think that was where my main motivation was.

I could count on one hand, only a couple of times where I would have just got really, not really down, but just thought, "Oh, I can't. I can't be bothered to go to physio today," and that would probably more be as a result of what was either happening at work or something else rather than actually just doing the work for my knee. Getting and having that 60 to 90-minute session was my escape and good motivation on that journey.

Interviewer: Those feelings of being over it, they weren't continuing throughout the whole rehabilitation process?

P7: No, I had a few mates that have done similar things as well so they were really good saying like, "Oh yes. You'll start to be able to do this," or, "You'll do this," or, "Sometimes it gets a little bit tricky when you do this." I had good support and normally feelings of doubt or negative feelings were fleeting rather than day and week-long agonizing thoughts.

Interviewer: Did you have any other strategies when it came to getting over those thoughts and feelings?

P7: I'm a pretty literal guy so a lot of the times when annoying stuff happens in my life it's just like shit happens like 'it could be much worse' scenario. Like you see someone in a wheelchair or something like that. I work in hospitals so I was still going to work every single day and I could see someone that actually couldn't walk and I was just like, "Well, it could be much worse," and then I'd be just like thinking, "I've got physio this afternoon. I'm going to try and do X." It was more like self-motivation.

Interviewer: Along the continuum around that one year mark were you experiencing anything? What were your emotions around that one year mark?

P7: One year, I guess they sent me off to do the testing, the fitness testing of my surgery and my operated knee was far more tested than compared to my non-operated knee. That gave me real confidence. From then on, I've really never thought that, "Oh, what about my knee?" I've never really thought about that at all. I was still totally living life to the fullest. I did it in July and in the December, I went to a music festival and was dancing around, everything like that. It was just like normal. Didn't even think about my knee.

20:23

Interviewer: In terms of the psychological contributors to the knee it sounds like you've had a very good kind of progress and outcome. What do you think the biggest thing that helped you progress so well was?

P7: Definitely the network of support I had around me but also just probably my upbringing as well. Just trying to stay grounded and thinking that things do happen, but you just work hard and make the best of a bad situation and if you work hard, you'll get the result at the end of the day. Yes, I had a really strong family network, friends were really good, work was really good. Apart from the actual injury, I couldn't have asked for anything more in terms of rehab.

Interviewer: What were the kind of messages that they were giving you that were so good?

P7: That you're not a burden and if you need help going to the toilet we can help you. They totally just, my family anyway, they just help you out in the day-to-day functioning that you think you might not be able to do or you just can't do in the short term. I guess the other messages, key messages for me would be like your work manager understanding that you need to leave work half an hour early to go to your physio appointment and then buying in, saying, "No, no, this is important. Go and do it because it's going to help you holistically." I thought that was pretty important and I had a pretty supportive manager at the time. Just friends, just because I might have been sore or I couldn't run around or anything like that, it didn't mean that they were stopping inviting me to things or leaving me out or things like that. I still just lived my normal life afterwards.

Interviewer: What effect has this injury then had on your entire well-being?

P7: It's a good question. You still have the fear of suffering some kind of major injury and having to go through that whole process of what I've just outlined as like the injury happening, having surgery, doing rehab, trying to coordinate the rest of your life, be it family, work, friends, whatever. There's still that, you don't want something like that to happen but I've tried to always keep a pretty positive mindset in that it was a pretty freak accident. I haven't had any other major injuries up until that point in time and haven't had any since then and you've really worked hard during your rehab, so just go and do life as you normally would.

The only thing that I haven't done since then is play rugby, but I wasn't playing rugby at the time anyway. I'd stopped playing rugby a year before then anyway. It's been a little blip on the road and it hasn't been a really long journey. I know some people who've struggled with their knees for years post-surgery but it hasn't stopped me from doing anything at this stage. I still have determination to go. I want to play netball again with my work colleagues. I'm not a skier but I want to go skiing. I still have determination to do that kind of stuff.

Interviewer: Has your perception of yourself changed then at all as a result of the injury?

P7: Yes, a little bit. I thought I could deal with pain pretty well but I was really, really sore for a good two weeks to three weeks post-surgery and I normally think, "It's okay. Just deal with it." I was really sore. Now I think maybe I'm a little bit of a baby with pain but apart from that it's been all right.

Interviewer: What about the perception that other people have had on you?

P7: I don't know, you'd probably have to ask them. I try not to worry too much about what other people think of me, or people that don't matter anyway. I don't know. I guess getting back into cricket straight away and everything, my teammates might have thought I was pretty resilient or strong for coming back straight away but that was more just because I love playing. I love playing with that bunch of guys and I really wanted to play. I don't know what other people would have thought. No, I probably couldn't answer that.

Interviewer: Their perception hasn't changed or if it has it might even be--?

P7: I wouldn't have thought so. I tried to go through life as normally as possible during that time.

Time: 27 minutes 36 seconds
