# Supplementary material for: Understanding Fear after an Anterior Cruciate Ligament Injury: A Qualitative Thematic Analysis Using the Common-Sense Model
Source: Int J Environ Res Public Health. 2023 Feb 7;20(4):2920. doi: 10.3390/ijerph20042920 (PMC9957354; doi:10.3390/ijerph20042920)
Supplement: Supplementary file 1 [file ijerph-20-02920-s001.zip › File S1-P8 Transcript.pdf]

P8 Transcript

Interviewer: No stress, yes whatever it is you need to do. All right, cool let's get started. Today you'll be participating a study that is researching fear, post ACL injury, the interview will consist of a series of questions that will explore your fear, your levels of distress and your confidence after injury. Please feel free to say as much or as little possible as you want on any topic and if you feel uncomfortable at any time, let me know. And we will move on. Sound good?

P8: Yes.

Interviewer: Awesome! All right, so tell me about your ACL injury.

P8: First time was 2011/2012, that was in America, I was competing in snow sports. I flat landed a 30 ft jump, missed the landing, just went straight over it. It was a really rainy day; it was raining on and off and sleeting sometimes so the run-in for the jump was changing every single time. Sometimes it was really icy and sometimes it was slushy and I sent it and just flat landed and completely blew the ACL part, that was gone and then really tore the medial meniscus. Then I flew back to Australia a week later, because I did all the MRI's and stuff there.

Spoke to my surgeon over here and really wanted to get back skiing as soon as possible. That's when The LARS were popular, so The LARS ligaments had already been out for quite a few years and I had a few problems with the earlier ones so we went for LARS and then I went skiing three and a half months later in Whistler on a spring camp, a moguels camp on a

glacier. I was more nervous about not being able to ski well rather than reinjuring it and that basically really got in my head to the point that I was skiing like absolute shit. Then I actually had a crash, bucket handle tore my medial meniscus and my knee locked at 60 degrees.

I had to get evacuated of the mountain and then flown back to Australia. That was bit of a nightmare that one. I wasn't afraid so much of reinjuring or damaging my ACL, I definitely wasn't doing anything to protect it. A lot of the coaches were like, "There is no way that you should be skiing blah-blah-blah-blah. You won't be able to keep up with the training regime and things like that." It really got in my head and I ended up overthinking and overcomplicating all the skiing. I crashed definitely because of that. After that like with your knee just heaps of work on the meniscus and things like that scraping out fissures and bone spurs and things like that. Then in 2012 I did my ACL again but I was coaching at Perisher.

The ACL did not snap because it is a lars it stretched, but it stretched so much that it was useless. No meniscus damage which was good. Anyway, again oof to the surgeon, I was going skiing in Europe at the end of the Australian season so I did not want to stop. I did not want to not go skiing in Europe and that was about four months away. So, I saw the surgeon in Sydney and he said he could give me an Achilles tendon from a doner and use that as the ACL and that would be absolutely bomb proof, that was what he had been doing for the rugby players over there. It was really strong; it is basically living tissue as well.

Since that, it has actually been really great. I cannot fault the stability of my knee at all. I get meniscus pain and I have got arthritis in it, so I manage it. I don't do any long runs. As far as thinking about it post 2012, honestly the ACL component of it doesn't really enter my mind. It's all about for me, managing the-- Like really being in tune with what sort of pain is fading back through my knee. I know what good pain is and I know what I had on that weekend,

nope you've have torn something, you need to stop, it is really swollen now and go see the doctor.

When it's like this, with this knee pain, fuck I don't want to do anything on it because I am really scared that it is going to slip and then potentially snap my ACL or something. Prior to the weekend, nothing really goes through my head around reinjuring it.

Interviewer: Quite a bit in there. So, you mentioned that after 2012 you stopped thinking about it. Before 2012, what were your thoughts?

P8: Before 2012, it was interesting because there was always a lot of swelling because I was still trying to compete and I was training so hard on it and doing moguls and jumps and then trampoline in the gym with the weights. A lot of running, a lot of box jumps and really high impact repetitive movements. I was constantly having to manage the swelling in the knee. It would always be a little bit of a niggle or something like that, so it was always on my mind. I knew something was quite not right, it was not 100% but my legs were strong enough and felt stable enough.

I was just always thinking about-- At the end of the day I would have to ice it for two hours, elevate it. After competition I had to ice it, and if I didn't, I would not be able to train the next day because then it would be too stiff. I would have good weeks and bad weeks but it was constantly the knee.

Interviewer: What were your thoughts process? How did you feel about the consistency or the constancy of the knee problems?

P8: It was a really conscious routine, life revolved around what I could and couldn't do with my knee at certain times of the day. First thing in the morning fine, go to the gym, do all your warm-up stuff, a quick ice and an elevate before then going out skiing for three-four hours, coming back and having lunch, icing and elevating and then back out for skiing, back in, ice elevate, gym, cool down, ice elevate.

I had the routine down to a Tee but it was pretty tiring and exhausting. You'd have all the coaches telling you, especially because I was in America, they were like, "This is not what you should be doing, blah- blah- blah-blah-blah, you should be having twelve months off and getting more surgery," and I was like, "No way! That's not going to happen. I'm not taking twelve months of."

Interviewer: So, did that kind of message ever get you down or, how did you feel about the coaches telling you and not being supportive?

P8: A couple of times I remember that they sat me down and said, "We want you to take a couple of weeks off and get tests and things like that. It really upset me. Really, really upset me. They didn't understand the LARS ligament as well. That's not very popular in America. They're used to guys coming out of The Steadman Clinic over in Vail and they perform tests on my knee and stuff like that and I did strength tests on my knee there and stuff and passed all the tests. I did incredibly rigorous strength tests to prove my point and things like that. Coaches would always be like, "Oh, maybe he shouldn't do this," or before a competition, they'd be like, "Oh, maybe you shouldn't do this comp, your knee's not looking great at the moment," sort of thing, blah, blah. It would really, really get me down because I always had to fight to tell them that, "No, I think it's fine."

Interviewer: It got you down but what did it mean to you that your coaches were telling you that your physiological health wasn't there?

P8: For me, I just really felt they didn't understand. They didn't understand the procedure I'd had and what it would allow me to do. Australian doctors and physios said I'm fine, cleared for skiing and rigorous fitness and all that sort of stuff, but then go to America and it's the way their healthcare system works and things like that, it's completely different. They have set processes in place in there. I just couldn't get them to understand. That was basically it. Then I remember they made me do a really rigorous strength test and I remember I was on the squat rack and they loaded it up with a ridiculous amount of weight and they were like, "You've got to do three reps of this, " or something like that and they kept jacking the weight up and I made it to the last one and the guy running it, the PT coach, he was just like, "What the fuck? Three months after ACL surgery." I was like, "Yes, mate. I've been doing this in the gym at home. I was doing this after a month." [laughs]

Interviewer: Obviously, that was quite good for you but what was the effect on your confidence, of the injury initially?

P8: Jumping was pretty dodgy, so I backed off. Skiing the moguls, fine. You ski the moguls right, you're not really going to hurt yourself too much, but jumping, there's so much more room for error, especially landing backwards. I really backed off training on the jumps quite a bit because I was really scared that I'd land backwards and then I didn't have 100% flexion in my knee, and so that was the biggest issue. Just spent a lot of time on the trampoline instead and a lot of jumping into the foam pit and things like that, so I basically changed how I wanted to train I suppose.

Interviewer: Why was that? What would a reinjury at that point have meant to you?

P8: Another reinjury in America, massive repercussions financially, and then because of my age, I start later, it would basically, in my mind it was like, "No, if you have another reinjury, another 12 months off, it's just not going to happen." It was a bit of an all or nothing mentality.

Interviewer: You've mentioned your main fear I guess was not being able to ski well and you just mentioned it again. Do you want to just elaborate on what that fear means to you or what fear in this regard was meaning?

P8: It's performance-based fear, so it's just fear of failure. You put everything on the line. You don't go to uni, you don't earn a living, you tell your friends and family what you're doing and they're like, "What the fuck?" Then you have the injuries and stuff and then it's all just a compounding effect, that fear of failure. That's where it all stems from. Then if you don't have support from coaches who you work with day-to-day, that further compounds it because you feel like you have to prove yourself, so the injury itself is not-- In my mind, it was a double-edged sword especially when I injured my knee in Whistler, they were all like, "Oh, you've ruptured your ACL, blah, blah, blah," and I was like, "No, I haven't. I've actually injured my meniscus. My ACL's fine." I was more worried about proving that I was right, I didn't bugger up my ACL again, and it wasn't an issue with that, and that wasn't the reason why I failed.

Interviewer: You mentioned buggering up the ACL. What's your relationship with your knee? How do you view your knee and your ACL and how do you feel about the whole thing?

P8: I feel that's my most connected body part to my brain pretty much. I know every single pain, niggle, where it swells up, where it feels tight, all that stuff.

Interviewer: Your knees, do you view them the same? When you picture your knees, how do you feel about-?

P8: I don't even pay attention to the other one, I ignore it. I don't think about it. Don't think about it at all but if I go for a run, I'm just constantly analyzing it.

Interviewer: Even now?

P8: Yes, if I'm out. Yes, yes, even now, constant analysis because the slightest-- Like when I was surfing on the weekend, just constant analysis of the pain and what the niggles are and dings and as soon as I feel something that's out of the ordinary, I'm like, "No, you've got to think about that really hard and try and pinpoint or try and replicate that pain and you need to decide whether or not you think that's new and you need to get off it, you've buggered something up again."

Interviewer: What's this constant analysis? What kind of effect's it had on your emotional wellbeing?

P8: I suppose instead of being able to go out and 100% enjoy something or really be in the moment like if I go out skiing backcountry and things like that, even hiking up, skinning up mountains and things like that, I tend to spend a lot of the time-- You spend 50%, 75% of the time thinking or analyzing your knee. You're not thinking something's going to go wrong, but you're just making sure that you're okay because you're in a strength sport sort of a situation

where if you fall over, you're potentially going to really hurt yourself, so you're just constantly thinking about that rather than the task at hand, I guess.

18:43

You lose a bit of being able to be in the moment and enjoy just being in the mountains or just enjoy being out for a run or things like that. I suppose the only sport I do now is surfing, is the only time where I can really turn off because you have the lulls between waves and because it's my back foot it's not really taking a huge impact, just being able to drive the tail is an okay movement for me, that doesn't really affect it too much.

Interviewer: You mentioned before that you were a bit scared currently, I guess after the last injury. Do you want to tell me what that being scared means to you?

P8: Sorry, you'd have to refresh me.

Interviewer: You mentioned that since this surfing one, the last day or so you've been a bit scared about the knee so what's that? Describe that.

P8: yes. If I've got any unexplained pain in my knee that I can't explain away or if I felt that before and it's just-- so that's just the arthritis or that's just the bit of my knee that's bone on bone, "blah blah blah," then I'm really afraid to do anything other than just walking. I don't take the stairs at work anymore. I've made it to work three times over the past-- since I did it last weekend, don't take the stairs. I hardly drive because driving can aggravate it. Obviously, no running. I've done swimming, that's about it, but even then, it's just really--

One is because the pain I felt was super strong and acute, and two, from what I understand, it can, if I did push it, I went for a run or went mountain biking or something, I could really destabilize my knee and then put my ACL at risk after that, so yes.

Interviewer: So, you mentioned the coaches a few times. Did anyone ask you about the psychological or any health professional specifically ask you about any psychological considerations of your knee?

P8: No.

Interviewer: Of the ACL?

P8: No.

Interviewer: Has that ever happened throughout any of your initial, immediate, or return to a sport

[21:30].

P8: PT coach offered a psychologist. That was 2014, so after my first one. That was because I'd had three or four scopes after the ACL, but I didn't follow through with it. They just suggested that it might be helpful, but that was about the extent of the conversation.

Interviewer: Did you think if that had been a conversation that would've helped you with a bit of your-- obviously, you mentioned with a coach to have been a bit more supportive you

think that would've help you ski better, but do you think health professionals in general being a bit more open about chatting with you would've helped psychological-wise?

P8: Yes, yes, yes. Definitely, definitely, on reflection. One, it probably would've, in hindsight, everything's great in hindsight. One, it probably just would have made me slow down and two, just dealing with the fear of failure thing and really unpacking that probably would have helped me ski a little bit better and then not put all my other body parts at risk, basically.

Interviewer: So how did you manage, internally? How did you manage your fear of failure?

P8: Oh, just the go-hard, strong-headed approach. [laughs] Ball it all up and just go really hard. Just put your head down and just muscle your way right through it, and hope for the best, pretty much, which is not healthy and doesn't work 99% of the time.

Interviewer: Perfect. [chuckles]

P8: [chuckles]

Interviewer: Did anyone help you deal with these emotions or did you really just not…

P8: Yes. Back in Australia, I started seeing psychologists and stuff, because this is all still taking a pretty a pretty big toll on my mental health and stuff, because after 10 or 11 scopes, before 2014, before my last ACL and then I had the ACL. So, prior to 2014, yes, I started going back to Uni because I was having a lot of downtime. In Perth, it hurt, I started seeing psychologists, and stuff like that. Yes, just gave me a different perspective, I think. Yes, just a bit of a different perspective about, "Why do you want to compete?" and dealing with the fear of failure.

Really just asking the pointed questions basically, like "What's the big deal if you do fail? Blah- blah-blah," but yes, that sort of helps because now I can just-- I go out and I enjoy sport a little bit more. I'll go out and I'll do backcountry skiing with Sean, go out with him quite a bit. Just enjoy it again, still going really hard and dropping off cliffs and doing big jumps and really pushing it, but it's the performance in my sport has really improved because I just don't have that fear of failure stuff running through my head all the time.

Interviewer: So, what's been the biggest thing that's helped get you over that fear of failure? What aspects?

P8: That took years of working with a good psychologist, a lot of self-analysis and meditation and mindfulness, and things like that. A lot of that was just self-help. I felt, in my circumstance, I just had to do that myself. So, in turn, yes. Even the decision to have this knee surgery today, right? Normally, in the past, I would be dying to get a surgery, like "Hurry up, get this done, I've got to get out skiing, and blah- blah- blah." Well, this time, those thoughts are still at the front of my mind, but then I have that little bit of extra sense now where I'm like, "Well, hang on. One, do you actually need the surgery and do you need it now?

What other priorities do you have in your life right then? What's your financial situation? What's happening at work? Blah-blah-blah-blah-blah." Run through the list and think about a bit more where I'm actually second guessing myself, which is great, because I never used to do that.

Interviewer: So, currently, then, right now, how is your fear of failure in regards to the ACL?

P8: In regards to ACL, not really existent. Honestly, I think it's already proved itself, in my mind. Yes, the number of times-- for me, it's a no-brainer. It's worked, it's definitely serving its purpose. Surgeons may have even taken photos of it since, like in a brace and I'll get more photos of it today and then it'll come out. Looks great its doing its job.

Interviewer: So, are you--

P8: That helps.

Interviewer: Sorry, I was going to say, so, do you think your knee function can still improve?

P8: Yes, I've got pretty poor flexibility that's impacting hammies and quads in particular, really tight IT bands, calves, and all the big muscle groups around it. If I don't keep on the stretching and foam rolling routine, knee function decreases quite considerably, in terms of flexion. Then pain throughout it, because IT band might be pulling it in a different direction, things like that. So, yes, I can definitely improve it, for sure, yes.

Interviewer: Would fixing those things help your confidence? Or reduce some of that fear?

P8: They do, yes, they do already, yes. If I haven't sort of stretched out and I go for a run, I won't push it too hard on the run like in terms of distance and sort of how hard I am running and what surface I'm running on, but if I've stretched out and it's feeling like really good things like that, I'm in barefoot running shoes, I'll hit pavement like it's fine. I'm fine with that. That just comes back to that self-analysis thing, I really have to think about how it feels on the day.

Interviewer: So, with the running and things on the hard surfaces, for other fearful movements, how do you normally respond currently?

P8: With my knee?

Interviewer: Yes, let's say you were confronted with a fearful movement. How would you respond at this point in time with your knee?

P8: Some of my most fearful movements are yoga poses, to be honest [laughs], especially if I have to flex too much, it actually feels like my knee is going to burst.

Interviewer: Oh no.

P8: Yes. Everything just feels really tight, like it's going to pop. It's not swelling, it's really strange. I think it's just a combination of meniscal shards and crap going on in there and an ACL that's probably a little bit more rigid than the original one. I'm not really sure. Sorry I don't know how to answer that one.

Interviewer: Has your perception of yourself changes or how is your perception of how yourself changed because of your ACL injuries experiences?

P8: I've learned I'm very good at sort of like regimented things and routines, sticking to tried and true sort of processes, having to go through rehabs countless times like I can rehab from this scope today in four weeks. I've already got it mapped out in my head exactly what I need to do day to day, hour to hour. I'm completely fine with that.

Interviewer: Yes.

P8: I suppose like that's actually-- That comes out, like I work in finance and that that comes out in finance I've been sort of keeping to that lines and process and things are definitely working out the way you want in projects or whatever you're doing if you stick to the process**.** I suppose that's something I didn't think which would come out or even flow on into other parts of my life.

Interviewer: It's like a positive kind of outcome of all these things?

P8: Yes.

Interviewer: What about the way that other people perceive you? Has that changed because of the injuries?

P8: Oh, I mean most people see me as a bit of a hell raiser already. I'm not surprised that I get questionable surgery in terms of a LARS and an Achilles tendon in my knee and stuff like that. They're a little bit surprised, they're definitely not shocked but I suppose my parents in particular give me the resilience to keep going, the keep telling me that they're like, "Jesus, has anyone told you, you have to stop?" I'm like, no they haven't told me I have to stop, they've told me I need knee replacement when I'm 50 but that's about it. It's almost just cemented my mentality [chuckles] for life is I suppose.

It's just a flow on of that. Just a flow on of my general mentality on how I approach life and everything is 100%, everything is 100 mile an hour.

Time: 33 minutes 34 seconds

Audio file name: P8.m4a
