# Supplementary material for: Understanding Fear after an Anterior Cruciate Ligament Injury: A Qualitative Thematic Analysis Using the Common-Sense Model
Source: Int J Environ Res Public Health. 2023 Feb 7;20(4):2920. doi: 10.3390/ijerph20042920 (PMC9957354; doi:10.3390/ijerph20042920)
Supplement: Supplementary file 1 [file ijerph-20-02920-s001.zip › File S1-P9 Transcript.pdf]

P9 Transcript

Interviewer: Hello ….. how are you going?

P9: Good how are you?

Interviewer: Good

Interviewer: So today I am going to ask you some questions in regards to your ACL injury, about how fearful and distressing it was throughout various time points. If you feel uncomfortable at any time you do not have to answer. If you have any questions let me know and we can pause or stop the interview

P9: Yeh no worries at all

Interviewer: So tell me about your ACL injury?

P9: So I did mine when I was 16 so a few years ago now, I am 22 now. So I was playing netball and went to land and then got taken out by a girl. She was quite a strong girl and yeh I didn't realize at the time that I had probably torn my ACL, but, pretty naïve as a 16 year old at a sports academy, so sports was pretty much my whole life, I basically went to a physio, I didn't get the actual best prognosis from him, he was quite young and I couldn't get into the person that I normally see and he pretty much told me it was just a medial strain he strapped it even though there was pretty severe swelling. So I don't think my ACL, now, as I have gotten older was fully ruptured at that time, I think it was on the verge of rupturing. So he pretty much strapped it and I kept playing and I was actually training myself to land on my opposite leg. Because I was like okay, I want to keep playing and I don't want to miss out so until it heals I will just land on my left leg. So, it was working for a little bit. I was quite slow

though, I don’t know why I played and I got a quick pass from a player and instantly my brain just went to land on my dominate leg, my right leg, and it just went and that’s when I could feel it was pretty bad. Yeh, basically went in to see my sports doctor, I got a scan straight away and confirmed ACL. I had torn a lot of my cartilage which now by surgery had lost a lot of blood so I ended up loosing a lot of my cartilage and I had badly strained my medial ligament, so yeh that was probably the most painful the medial ligament. I had quite a bit of damage there, as well. So yeh, I also had severe bone bruising, so they originally thought that I might have had fractures in the patella as well so they put me on crutches for 6 weeks. Which turns out it was bone bruising and I ended up losing so much muscle in that time in that right leg that it became below critical so my surgeon Peter Wilson was like I am not operating on you until you build that muscle up, so it took another 6 weeks to build that muscle back up and then had surgery and started from there.

Interviewer: So you are 22 now?

P9: 23 in December

Interviewer: So you did it when you were 16 so 6-7 years ago.

P9: Yes about 7 years ago.

Interviewer: Yes so 2013?

P9: Yes 2013 from memory, about that time.

Interviewer: So after that initial injury you said that the physio strapped you up and then you went back into sport, how long did you have off before you were back into sport? From the first injury when the physio thought it was your medial ligament.

P9: So I kept playing until it fully snapped. And then I had off, when I did it, and he thought it was medial, I played for 2 weeks.

Interviewer: Straight? Wow.

P9: Yes, straight afterwards but I was landing on my opposite leg and I was being quite careful on it.

P9: I think I was playing a lot of sport at that time and I think I had cut back, knowing that it was strained, but I didn't really think I never had done a serious injury so I just kind of thought that it would heal. The total amount of time I had off was about 15 months before I went back to sport for the full injury.

Interviewer: So with the initial injury, you mentioned that you weren't really sure what happened and you had some bad advice were you fearful at the time of your first rupture?

P9: Definitely, I knew I was in a lot of pain and I knew something wasn't right, but I think at that stage I wasn't that fearful because I didn't know exactly what it was. I think the fear really kicked in when I heard it was an ACL. I had heard of a lot of people doing their ACL, a lot of football players. At that time I didn't realize how popular it was and how many people had done them through sport and even just through life, so once I realized what it was and the severity of the injury that's when the fear kicked in. I was like oh my god and then I realized that I had lost so much muscle I was like I am never going to be able to go back and play. I think the fear kicks in as well when you know its like 12 months out, at least, I think that was quite shocking.

Interviewer: Why did the fear kick in more at that 12 month mark?

P9: I think being so young and having sport be 5 nights a week, pretty much training, playing competitively I think knowing that you are having 12 months out and you have to do quite intense rehab and you have to do a biggish surgery to reconstruct the knee and then because of all the other issues I had when I found out it was pretty much like, Ruptured ACL, lost your cartilage, quite bad severe bone bruising, you have done a number on yourself, so I think then I was like how am I ever going to go back to playing the way that I was, I kind of thought in my mind that, nope, it was so painful that I don't think that I will ever be back to playing how I was, so that is probably what initiated that fear.

Interviewer: So you had the initial injury then you had the 'real' fear kick on when afterwards you had the surgeon tell you, you would have the 12 months off.

You also said about the muscle wasting, was that immediate straight after the injury?

P9: I don't know, I think in my body that I just loose muscle quite quickly and I have always had muscular calfs and quads and as soon as I was on those crutches and only using my left leg, when they thought I had those fractures that's when my muscle just wasted away, I look back at photos and it is so different to my left leg. I really did lose a lot of muscle and I really do think now that I have done a lot of rehab, you wouldn't be able to tell now, but I can tell myself that the muscle isn't as strong as the left leg. But probably once that muscle had gone, they did say to me that most of this 12 months will be building back that strength around your knee to protect that ACL. Looking at my leg in the mirror, going this is never going to come back. It's so below critical, its so wasted, I don't know how, that's probably where the fear started. 12 months seems like a long time, but, I don't know with the pain and the muscle wastage I don't know how this is going to work.

Interviewer: So It was more a bit of fear of the unknown and a bit of fear about not knowing how you were going to achieve your previous physical state that kind of really got to you. Not so much that initial injury but it was the perception of that feedback from the doctors particularly the surgeon. Did the surgeon deliver that message or is that a global message from multiple practitioners.

P9: Yes, so my sports doctor Don Macintosh who I have seen for years, pretty much since I started sport. He told me the message, but then when I went to Peter Wilson my surgeon he confirmed everything. He said "Look, you know, we're not operating on you for six weeks until you build that up. That's below critical. I won't touch the knee because if I touch the

knee now, they'll be a good chance you'll never get that muscle back". And I think that was also, like, 'woah', like is in it would never get to where it is now.

And yes, I think it was similar messages between the Sports Doctor, my surgeon. He wanted me to have the best opportunity to come back to sport. It was like, look, I think you need to strengthen it otherwise, yeah, you'll struggle a lot more. So I think that kind of scared me as well, a bit. Yeah kicked in that fear.

Interviewer: It all sounds very distressing as well. Would you, would you say it was a quite distressing experience?

P9: Yeah, I think at the time yeah, it seems like it was the end of the world to me being 16 as well. I think because I was young and it was like, 'oh my God, injury' Like I never had an injury really like it. Had minor ones but nothing severe. I think yeah, it was quite distressing at the time. I think if you just hear 'ACL' or you hear it, like I don't know, any major injury that you hear people do, I think that kind of kicks in that fear like, 'wow, this is bad'.

But then I think my mind over time now has kind of reflected on it and been, like, okay, and if I heard that now I probably would be able to, you know, process that information a little bit better. But at that time, yeah, it did seem like the end of the world. Kind of seems like the worst thing you could hear.

Interviewer: Yeah, so it kind of changed and rocked you a little bit.

P9: Yeah.

Interviewer: Yeah. Okay and did it have an immediate effect on your confidence?

J Yeah, definitely. I think confidence is the one thing that, throughout the whole injury it was, like, my main concern.   Because as I said before and because I think because it was a painful experience, rehabbing it and everything and because it was so wasted.. You know, I'd go watch my teams play and I'm just like, uh, I'd look at mum and be like I couldn't imagine landing on that leg. Like that fear ..I just can't imagine ever going back to playing at that level.

Ummm. So, I think after I got the clearance it was really like…. until I got asked to fill in and Mum and Dad said, "like just go play just don't think about it". It was a bit like, I don't know when the right time is. Like you're sure it's rehabed? Like I just, yeah, I was doubting myself a lot and doubting the work I put in, but, yeah, touch wood, paid off and it was all right.

Interviewer: So you had changes to your confidence in terms of what you thought you could achieve with your rehab or with your end goal?

P9: Yeah.

Interviewer: Yeah. Okay.

P9: Yes. Yeah.

Interviewer: It's interesting. So at the time of the initial injury, did anyone ask you about your fear?

P9: I mean it was a long time ago. I'm not … I think more when I spoke about it. I don't think I was ever asked like, you know you worried it was more like what are we going to do to get your knee better? It was never really about getting back to sport. I think that's what I noticed a lot of the sports play…unless it was my coaches and stuff, it was, like, you know, 'we want you back'. But I think the sports doctors and physios and stuff it was more like, how can we rehab this so you can get back. It wasn't really that the confidence kind of came in later when they were like, 'All right, you're good to go". Like "You don't need to keep coming back".

'Are you sure, are you sure I can run?' But yeah, they were really good though. My physio was fantastic and he always, yeah, gave me a lot of reassurance, and, yeah, got to the stage where he's like, "I'm not gonna answer your calls. If you keep coming back, you can play. Like go give it a shot." And then yeah did and since then I was fine over time. It yeah got better

Interviewer: So it sounds like at that end stage. They particularly kind of really reinforce that physically you were doing quite. Well, what about earlier on maybe that at that at that initial time. Did anyone ask you how stressed you were or how fearful you were or talked about those confidence issues?

- -

P9: Not necessarily. I think it was more how are you feeling with it in the sense of how the pain lies and how the injury is healing and how have you gone sort of thing. It was more about the actual injury and I don't think it was mainly about the confidence because my issues were mainly directed at, Like is it looking right? Like I've done this I've got this pain. A lot of

the time is spent talking actually about the injury itself not myself. Yeah combats and things like that.

Interviewer: So in that regard how do you think you dealt that initial time with the fear and the distress?

P9: I think, I wasn't too bad. I mean I knew I couldn't do anything about it. I knew I just had to put my mind to rehab and get it done. But I still yeah, I was quite disheartened with it all because I went from doing such intense sport to barely being able to walk. I had to kind of teach myself how to walk correctly so that I wasn't doing damage and things like that and then yeah, like a few things happened like I got at one stage I got really bad shooting pain down my calf and had to get rushed in for a scan for that because they thought it could have been another complication. It just like piled on top of things, I was like I'm getting pain here, but I thought I wasn't meant to get pain and I don't know. I think I just didn't know what was going on and I was just over the negative kind of news of this injury, but then it just became the new normal, probably six months in I was like, okay, this is what I've got to do to get back and I was pretty motivated when it comes to rehab when I'm getting back to sport. So I think I kind of motivated myself as well.

Interviewer: Yeah, so at that initial phase where you kept getting all that negative information brought in. Did you feel like you were dealing okay with that or did you feel like you had people helping you with that or did you feel like you kind of had a management strategy that you found helpful?

P9: Yeah. I feel like everyone was quite positive around me. They're like, you know, it is 12 months. But you know, you'll be back before you know it and I did have a good support network, but I think I'm quite an independent person when it comes to things like that. It's just like well, I've got to do the work. There's no point in sitting here complaining about it. If I want to get back. I've got to put in the rehab and my mum's like the same she was literally every second rehab rehab.

Like I really did do the extra miles to get it to where it is. I did struggle, like some days like I was just like im over it like I don't want to walk up the hill and do this. I don't want to just lay there with my leg out straight and contract my muscles. Like I was over it, I was bored as well and I went to a sports academy so I was watching my team train every day. I was going to like my sporting events. So yeah, it was annoying but there was nothing I could do about it. I just had to yeah, and I also do a lot of water sports. So we have a boat and go water skiing and things so and we go surfing and stuff as well. So they're like don't go in the waves because you don't want to you know, get knocked and twist your knee. I was like I can't do anything. This is so boring. But… eventually I can do it now

Interviewer: How far did that message get reinforced, about not twisting your knee in the water sports?

P9: I'm honestly not too sure because it was so long ago now my brains a little bit confused. I think it was because he say when you first get your surgery, it was definitely after surgery, he said it'll be at its strongest and then there's like a period of time. I remember that it kind of weakens off a little bit where like you do all your rehab and then it eventually forms into the

ligament. So I remember I think it was within that stage that I went and he was like, you know, just be careful you can go in but you don't… It was when I was trying to keep everything straight. I was trying to limit the twisting and rotation of my knee. But yeah, I can't a hundred percent remember exactly what period it was now it was so long ago

Interviewer: So talking about that three month period that's kind of when you started to resume your general activities or normal activities. How did you go at the time with your fear?

P9: Probably. Yeah, I think my fear was more, just, I was scared I was going to redo it. So I had a few instances where I kind of forget in my mind and go to do something and I'm like, oh my God, or I get a pain or something and I am like oh did I just redo it, I was just scared the whole time. I was scared of redoing the injury because I knew it wasn't as strong as it was previous to doing it. So yeah, that was always a fear. Because you would forget because sometimes it would be fine and then the pain goes away and I was used to it and then I do something and I was like oh no, like have I done damage? I'd get achy because I have probably been on it too long, which now I know, but at the time I was like, okay, I've redone it, ill need to have surgery and longer out, it was just kind of that negative. Like I'll be really worried about it. So yeah, probably the whole way through that was consistent.

Interviewer: Where did you feel like that message of potential damage was coming from

P9: I think just probably from, you know my physio and my surgeon things like that, like they would enforce you know what you can do and kind of what you can't do and I don't know I just guess I didn't trust in what I was doing, I felt because it was painful below the muscle, they would say, you know, you know you can run in a straight line now and kind of if I slightly divided. I'm like, oh did I go out of that? Like I was just very like to the point, I was that annoying kid. Yeah, so I just I think I see it as a lot of self worry. I don't think it was the team around me. I think they were really positive and gave me, you know, the best advice they could have, I had a great team. But I think I just worried a lot about it myself.

Interviewer: So at that time would you consider yourself quite distressed about the stability and the function of your knee.

P9: Yeah, probably definitely yeah. I was more worried myself then probably what I needed to be but because I just wasn't used to it and also because when I like look at it, it was so much smaller than my other leg. I was just like, it just felt like it was never increasing and it was like all of a sudden one day, ‘like they're kind of the same now and yeah, they're okay’. But yeah, I think it was it was definitely a mental thing because I remember like post-surgery, Sorry no, post-injury like I'd gone back to Sport and everything and we were going to go waterskiing and things like that and I remember them telling me like yep you can Waterski, you are back to normal now, like you can do everything. I do know a lot of people that wear braces when they waterski, because it's a lot of pressure like you can feel that pull. So I was like awesome I've got to get a brace. But he said it's not necessary. It's just a mind thing people feel comfortable. I was like alright, I gotta get a brace and I'm short Im 5 foot 3 and I had this massive brace to water ski with. Then mom used to always remind me like, he's your

brace and it was fine. Like it was a bit limiting, but I could do it and I felt mentally yeah, it's protecting me and then one day Mom didn't come and I just jumped on the ski, completely forgot about it and skied like normal and came back and moms like what did you forget? And I was like what? she's like you brace, you didn't wear it, you don't need it and I was like, oh, so it was that mental thing and since then I am fine, I don't ski without it. I'm fine without it. But yeah, that kind of taught me like oh, yeah it was my mind telling me I had to wear it for safety.

Interviewer: So this was after the 12 months was it? how far after the 12 months?

P9: I think I was about 15 months until I went back to sports so just over 12 months. I remember It took a little bit longer to go back and then I probably didn't water-ski. Probably maybe another four or five months after that. Yeah, I'm pretty sure I took a little bit longer to go back to that just because I was a bit more worried about that. Yeah changing direction and things with the skis, I didn't feel as in control with that. So I think I took a little bit longer using the brace and then probably not using the brace that was probably a good two summers after it. Like I did use the brace consistently for a while

Interviewer: Even up to the one year was it

P9: yeah after the one year, so I didn't use it

Interviewer: Was that just for activities?

P9: Yeah, just the water skiing.

Interviewer: Did you use it for anything else? like any other sports or anything.

P9: No, so. I didn't use a knee brace or anything in sport or anything because I think, for me, I think my physio knew I was like a real mental kid. Like he said if you have that brace on you don't need it. But if you have that on that signifies that that's your bad leg and mentally I'd look. So he was like “you don't need one, your legs strong you rehabbed it”. You don't need a brace, but I think he just recommended it for water skiing knowing what I'm like and I know because it's a lot of pressure when you're coming out of the water and it's quite like, you know your knees quite loose in the…. when you're skiing. So I think he said you know for water skiing if you do want one it might help you get back to actually skiing. but yeah for sport I actually never wore a knee brace. I think I maybe put one of the compression bands on once just to let me know you know this leg is bad, but he told me you don't need it. Yeah, I don't play with anything now.

Interviewer: So when you say bad leg, describe what you mean by bad leg?

P9: That's just what I used to say, like good knee and bad knee. It's my bad leg like even now like if we talk about it. like I sprained my ankle the other day and told my parents about it and

I was like oh its on my bad knee and they're like, “okay. It's been seven years let It go, like give the knee a chance.” Yeah. So I just always referred throughout my injury that my right knee was my bad knee because that's the one that I did. So yeah, that was just a self-thing that I created.

Interviewer: How would you class that? Do you think that is a sign of reduced confidence in that leg or was that an altered perception of how that leg looked or was that just a result of the injury and the loss of muscle.

P9: Oh, I think it was a bit of all three sort of thing. I think it was just you know, you've got the injury this knee isn't working as well. This is the painful one, kind of just reminding myself this knee is not as good as the other leg, this knee, you know isn't going to do the same job at that stage. It was like because you are asked so many times at so many appointments, okay which knee or you know get your knee out. It was sort of like, okay. Well, this is my bad knee. I just labeled it my bad knee, sometimes I find myself saying it now, if I hurt myself or have a pain or something. My parents will be like, ‘which knee’, ‘you know, the bad knee’, but now even though I know it's as strong as the other one. Yeah, it's just a mental thing.

Interviewer: What do you think that reinforcement by your parents about it being the bad knee, even up to now, does using that term precipitate the fear?

P9: I think mum was like ‘you don't have a good or bad knee’. She was kind of the opposite. She was like stop referring to yourself as that, but, because I always do they'll be like whatever if that's what you want. Like they know which knee I'm talking about. But yeah mum was kind of the main one that was like you need to go in now and think that both your knees are the same there's no difference, treat them both the same, go back and play like normal once I was cleared, of course because I was really stuck in that, you know, and I think it came up a lot like, you know, you track things like yeah, but I have done my knee so, you know because you worry it's just I allways have that worry that something was going to happen again. I don't know I just stressed about it a bit. So I think it was a lot of self doubt.

Interviewer: So back to when you were first looking at that brace at that 3 month mark or so around when you were getting back to general function. Did anyone at that point have the conversation with you about your stress? You're feelings about the worry, the confidence and the fear.

25:10

P9: I probably definitely did with my family and things like that and I think a few teams wanted me back and I was like, ‘ah, I don't know ‘like I just was still a bit iffy about it. But I don't know I remember because I used to play both basketball and netball now, I'm just netball now, but at that stage, I was both so I remember I felt a lot more comfortable going back to basketball first. So I remember I went back to like my domestic club I was training with my rep club and I felt a lot more comfortable. I think it was because I wasn't having to land on the leg and it wasn't the sport that I did the injury in and I felt I think I classified netball as the one I did, you know my injury and the one I would take the longest to come back to so I remember yeah, I started out with basketball and then I didn't really have any

ideas on when I was going to go back to netball until a friend asked me to fill in and it was quite last minute and Mum dad would just like just go just try out, you know, see what happens. You'll surprise yourself. You've been playing consistent basketball now for weeks go back. So I did, I filled in and after that I was quite shocked at how well I did. So I think after that I kind of joined teams and now I'm full netball again. So I think it took me having to just go and do it, to get over that mental game of worrying about it.

Interviewer: So what was the most fearful thing in that mental game when you were initially holding back from netball?

P9: I think it was just the landing on the knee because it was my dominant leg. I think it was just that that landing and that force I just I think with basketball your free moving, you're running and I knew I could run, like as soon as I could run and they told me I could run I would go running, that was my thing. I just wanted to do exercise in any sort. So I wasn't too worried about running and things like that and probably in Basketball it is the same thing you are changing direction and things like that, but just mentally it felt a lot better. I think it was the stop-start in netball and that landing and putting a lot of the force through that bad knee that I was not comfortable with, but then I did it and I think my confidence just built after that and then I was fine.

Interviewer: So do you think when you exposed yourself at the end stage to that Landing action in netball and basketball, sorry how many months was that past your initial injury?

P9: Yes, I think it would have been I always say 15 months. And I think I went back to basketball at around maybe 14-15 months like post-injury to just over a year, but I think netball. I know I went back to netball later because I went and filled in and then by the time I joined teams it was a little bit later. So I don't know, I'm not a hundred percent sure, but it was yeah a little while after that time that I went back to basketball so basketball is probably 15 months. And then maybe it was a few months after that, that I was consistently playing netball. Yeah,

Interviewer: So on reflection? Did you think you had a process that actually built back this the confidence to be able to go back during your rehab? Or was it just get to 15 months get exposed to it and you were good.

28:23

P9: Yeah, I think I just had to do it. I just had to get out and give it a go and I think after that game because yeah, I think I had an all right game and I was… I really found that like netball's like my sport, like a really do love it. So I think that came back and it was almost like I really do want to be doing this so I kind of kept filling in for that team until I eventually became part of it and now yeah, I'm play full netball. So yeah, I don't know. I think it was just something I had to just go and do and I think just being told, 'like go fill in', getting that kind of push from my family to go do it. Yeah that helped. I don't know how old I was. Yeah, so I did the injury at 16, I'm not even sure if I could drive then, maybe I didn't drive then but I made like my parents come with me. I was like 'come because if I have to get off this court you're going to be taking me, they're like, oh my God, you're so ridiculous. But I remember I was so nervous. I was so nervous before going and taking that Basketball Court. I wasn't too

worried. But netball I was, yeah, I was so stressed and I think that was also due to the fact that, how was I going to play?, you know, I didn't want to be terrible at the sport and I was like, I don't know how I am going to go what if I am really bad. I think that was also on my mind and then worrying about my knee it all just kind of annoyed me.

Interviewer: Yeah, so going back what were your biggest fears, you were fearful of re-injury or?

P9: I was definitely most fearful about re-injury, but I think also the ability. Would my leg, would my knee, be able to… would I be as fast, would I have pain every game? I didn't know how my knee was going to react because I did lose part of my cartilage. They did say, you know, you may experience pain. It's been okay, like it's been pretty good so far, touch wood. But yeah, It was just all those things. I didn't know how it's going to react. Is it going to be a strong? Would it just randomly give way? like I had no idea what to expect going back. So I think that all just made me really nervous but then after the game… I just slowly kept playing and then yeah until eventually I just forgot and yeah, it's been a few years since I've done it now.

Interviewer: So your confidence had maybe taken a hit due to some of those distressing messages that were fed throughout the rehab and the initial injury. Obviously. It really tied into that belief.

P9: Definitely. Yeah. It's because I still remember because I went and got my scans done and my sport doctors really good. He got it. He got them faxed to him straight away because I couldn't get in for an appointment. So we actually called my mom to let her know it, because they thought I had the fractures. They were like we need to get her off that leg. So my mom, being my mum, so she just like came down, she was just like look Jamie. It's not great. You've ruptured your ACL, you know, you've really strained your medial ligament, you've damaged cartilage, it's going to be a good 12 months out. You're going to have to rehab hard and I remember just sitting on the couch with my friend like what, I just thought it was a two-week strain? Like I thought I genuinely thought I'd be back playing, like I had no idea of what I'd done. I remember like that initial thing of just like whoa. This is going to be a weird long time out and I remember going like, oh my God, like 16 almost. I think I was like almost 17. I was like, I'm going to be 18 by the time I play like that's crazy to me to think that. I was just thinking all these things. But yeah, then when I was in it, kind of, just, you get used to it and then yeah ended up going back. So yeah.

Interviewer: Yeah, it's kind of reinforces that initial message. It said, that it wasn't necessarily the pain or the injury at the start as so much as what the effects of the injury are.

P9: Yeah. I just wanted to click my fingers and be back playing I didn't want to do the work, because it was such boring rehab as well, until it kind of got strong I had to do, you know contract the muscles and like building back that muscles just yeah, it was so annoying and I have to go to the gym and I just because I was doing rehab and I would only be able to do certain things. I just felt like that weird person in the gym and just doing the leg press. I just hated it. But I'm glad yeah, I still do all my exercises now to try and keep it strong.

Interviewer: So just on that how fearful are you now in regards to your ACL injury?

P9: I'm not really fearful at all now. I've gone back and, yeah, I probably played better and harder than I did probably before. I think over time I built up the confidence filling in for like Stadium teams, where it wasn't as intense and then kind of went back to a higher level, where, you know, it was more body contacting and was a bit of a tougher game had to be, you know, make sure I was playing as hard as I could and yeah, I don't know, I think over time that's just kind of gone out of my brain. There's certain times I'll get a pain or something and I do still worry about it. I know I've done it. But yeah, actually when I'm playing I'm fine. It's not really my biggest fear anymore.

Interviewer: You said you've gone back even harder, have you achieved the previous level sport that you were playing.

P9: Yeah definitely. So when I went back even with basketball, because when I left I was in like Juniors and then I was like all of a sudden an adult was a bit weird. I went and played youth-league basketball women's basketball. And yeah for netball now, I've Captained local clubs and things like that where I was like, 'no, I'm never going to go back to that'. Yeah, so I definitely have yeah gone back to exactly what I was doing and probably played a lot better netball then what I was before as well. So yeah.

Interviewer: You mentioned occasionally you'll get worried about the knee. Is that, do you think that's on a continuum to distress and how worried would you get at any time or how distressed do you currently maybe feel to do with your knee?

P9: I don't really think about it, I don't worry about it when I'm playing, it'll be more, for instance I was running and my knees kind of locked back a little bit, you know hyper-extended a little bit and I'm like ohh or I've landed a bit funny and I've kind of corrected myself and I'm like ohh it felt a bit off, like that made me a bit nervous, or seeing other people go down with knees. I'm like, oh I know the pain, so yeah, I get a little bit worried if I do something like I go oh I bent my knee back, but I'm pretty yeah, I don't know, I try to do my exercises and keep on top of it. To be like well if it's going to happen, it's going to happen. I can't do anything about it. But I've got my knee to the point of where I want it. So if it's going to happen, I can't really avoid it now, you know, I'm just going to go and play like I haven't done it before but if I yeah, keep my knee is as strong as it could be I think that helps as well.

Interviewer: Basically. Oh, so when you see and you feel a bit ugh. Is it when you watch people do their knees or is when they do the movement?

36:11

P9: I think it's more like I've seen a couple girls from my team and other teams were playing go down and do their knee in games and you see it in football games, you know, I watch the AFL quite a bit and I'll see them land and they suspect a knee and just watching that movement. I'm just, it makes me like cringe. I just I feel, I feel for people who do it because I know it's such a long process and especially with that fear. It's not just 12 months and I don’t think people realize it's not just 12 months, you've got to get used to you know, transitioning back into Sport and then you forget like the rest of your body hasn't been doing much for 12 months. You've been really focusing on this knee. So you get a lot of Sorer and there's just a lot more issues that come from it, and a lot of mental, kind of what we're talking about today. So I think I just know how long it's going to be and when you really do love sport and playing or even just training or going to the gym. It does impact your life a lot.

Interviewer: For you now, it sounds like you don't feel threatened unless you see any injury? you think you're pretty good with all movements. You don't think about it during a game?

P9: Yeah. No, I'm pretty much over time. Yeah, I’m pretty much good now, like I'm not too worried about it. My fear is probably more about how I'm going to play that game, not really my knee. So it's switched. Yeah.

Interviewer: So just overall tell me about how your knee injury has had an effect on your well-being.

P9: I think originally there was a period of time within my recovery. And when I was rehabbing it post- surgery and I just was really over it and I remember that's when I was quite disheartened. I was probably was never too bad or to disheartened, like I was quite motivated, to get back but I just remember thinking like it just feels like it's never going to end. I think overall it definitely made me test my mental strength and how I would go getting through because previous to that I've never really had to deal with anything, you know, 16 years old just like playing Sport and chilling but it kind of impacted every aspect of my life. Like I couldn't wear high heels like going out to parties or, you know, you have to be cautious about everything and everywhere you go you have to worry about your knee. I remember like, you know, you go to a party and I don't want to get knocked over because that could you know hurt my knee I don’t want to fall down a step and hurt my knee like I was very cautious about everything I did in life. So I think getting over that as well and remembering like yeah, I guess I can wear heels now, I guess I can do this now that made a big change on my well-being once I got over that. but, definitely during that time. I think my mental well-being was probably a bit affected because I was missing sport, but also trying to deal with this injury that felt like it was never going to end.

Interviewer: You mentioned worry again. So for you how do you describe your worry? Was it a form of distress or was this something else?

P9: I think worrying was probably the main thing I did. It was always worrying about everything to do with my knee and I think it was just that it was that distress of redoing it. I just didn't want to redo it and I didn't want to go back through the process. I just didn't want to undo all the work I had just done. So yeah, I kind of forgot but yeah, just talking about it

then. Yeah everyday life I'd worry because I couldn't just… like friends would go do things for birthdays and I'll be like, oh I can't do that because of my knee, like I couldn't it kind of changed my whole life in a sense for that time because I had to be cautious about my knee and what I was allowed to do and what I couldn't do. So yeah, I definitely did worry, I think that would be the word I would use to describe that whole 12-month period I was just worrying about everything

C:just like constant underlying worry throughout.

P9: Yeah. Yeah constant. Yeah this constant worrying about it the whole time

Interviewer: So reflecting on it now has your perception of yourself changed because of this knee injury?

P9: Yeah, I think definitely I think now like I've been able to help other people going through injuries and things like that. It probably wasn't too bad because I was doing VCE at the time so it probably did help me focus a little bit more on my studies and a little less away from my sport which at the time was like, 'this is the worst thing in the world what I have to study' I couldn't use the excuse 'I got training' It ended up yeah being really really good for certain areas of my life. But yeah, I still, would have, if I could have taken back that I'd done it, I definitely would. I think overall I learned a lot about myself as well and that I'm quite self-determined and it kind of enforced like you have to do the work to get where you want to be. So I think that helped and mentally I think it made me have to deal with something that

was out of my control. So I think that definitely helped my overall life now. I probably didn't realize it at the time but I realize it now as I'm getting older and further away from the injury I can kind of reflect on what I went through. A friend of mine actually just did quite a bad injury wasn't an ACL but she did some damage around her knee. And yeah, I've been helping her get through and she was struggling a little bit but I found that I could kind of give her little things to do to try to help with that. So I guess that was a positive out of it.

Interviewer: What kind of little things? Are you getting her to do.

P9: I'll just suggest just doing little bits at a time. Your knee isn't going to be the exact same as it was before at that period of time so, you know, get excited when you're learning to walk again and you can maybe walk a kilometre. Originally you'd think walking a Kilometre, is like, a grandma could do that, but I think I had to like be like, oh no, that's a big step towards the end goal, doing little bits that will help chip away at the end goal. Running in a straight line for the first time, she could eventually run and she went on her first little run. I was like, well don't overdo it just because you can run don't overdo it, because you don't want to get really sore muscles after or you know get really fatigued and then you can't run for another two weeks. Just do little bits at a time until you build up to it and that's what I learnt along that way. It wasn't zero to a hundred. You had to build towards that hundred goal. That's probably something I took away from it.

Interviewer: So you've learnt some resilience factors and some ways of self-motivating and dealing with the injury in terms of compartmentalizing everything to certain parts.

P9: Yeah, definitely.

Interviewer: Have other people's perception of you has changed because of this injury

P9: I think probably family, because they probably didn't know how I was going to react either being young and things like that, we've always been quite an active family we have gone away and done, you know, lots of water sports and camping and things like that where I kind of just had to deal with it myself. I think the one thing I was doing, was like 'I don't want you guys to be affected because I can't do it'. Like I'll eventually be able to do it. So I think they were a bit like, oh gosh, she's being pretty good about it. I think once I got over the initial shock and upset. I was just like motivation mode. I was like, there's no point crying about it. You've got to just do what you got to do to get it done and that's kind of been my message all along with everything I do now, it's just like well, there's no point sitting down and worrying about it, that word again, you might as well just knuckle down and do it because you're not going to get anywhere if you don't, so yeah, I think their perception probably changed slightly, but I haven't really ever talked to them about it.

Interviewer: That sounds like a change in the positive. You think that you have shown early when you were 16 some characteristics of self-care and self-management, determination and drive.

P9: Yeah, definitely. I think at the time I just felt like it was what I had to do, but now I'm probably like okay it was maybe, it was teaching me something and I never really have gone through anything where it was soo mentally stressful for myself. So I think it kind of showed how I reacted to that and yeah wasn't in a negative way. It was quite positive and then I was kind of rewarded by being able to get go back. All my hard work kind of paid off and I've been out of play and be back to kind of normal with what I'm doing now. I'm also a PE teacher and I also study outdoor environmental science, so I'm always going away and doing things. I also didn't want it to impact my life as well because yeah, like going on camps and things I didn't want to be that kid that can't go on the camp and can't pass the degree if I cant do the activities that I have to do to get by, so that also motivated me because I knew what I wanted to do quite early on. So I think I that helped me push on as well.

Interviewer: Yeah, so you developed some really kind of driving factors, to improve your confidence and you held onto them throughout your rehab.

P9: Yeah, and I think it was a bit of a show, I'm just like that, even if I'm worried about something I won't ever show that I'm worried about it. I'll just I'll secretly worry about it. And then I'll figure out a way to overcome that rather than publicly worrying about it, f that makes sense. I kind of work out my own personal strategies to overcome it and then I won't necessarily display it, I might reflect on it later. Like 'I was worried about that'. But at the time you probably wouldn't know I was worried about it because I'm like, well, I'll be fine and I was.

Interviewer: How would you describe that ability to identify something, not vocalize it but internally deal with that worry. Was that just a constantly changing thing where you would push yourself challenge yourself? How did you get over that worry at all those different steps?

P9: I think, I just had to mentall be like, okay, I'm worried. I'm going to do it but I am doing everything that he saying I've done everything the physios recommended me to do up until now. I have trust in this physio. I'm going to trust that what he's telling me is the right thing to do and I think because I got that bad prognosis at the start, though my physio now was like there was severe swelling that indicated damage like he shouldn't have done that. That actually kicked in a bit of fear towards, you know, physios and doctors and like, you know health professionals because you go ‘Oh I think that did me more damage’ and I mentally was like I was a bit worried. So I think that actually did increase a bit of fear, I was always like, okay do I trust in what I'm doing? Like are you sure? I'd say like ‘are you sure I can do this’. I suppose a little bit out of my zone and be like, yes, you can do it. So yeah, I think that definitely affected my fear as well. I kind of forgot about that but yeah just set little goals as I was going. I kind of put all my trust into my physio who is fantastic he was really good along the whole way and then I just chipped away at it. I just kind of tried to focus on the end goal, which I've always been a person that's like I can do it. I want to be the best straight away like I always like to jump five steps. So that really tested my patience having to like chip away at it. Yeah.

Interviewer: So your practitioner relationship actually was a big determinant of fear in the initial phase. It might have even increased fear for you, but then having a good relationship

with your physio afterwards improved it. What about that relationship? What about that physio made you trust him more,

P9: I think because I had never done an injury, I always had seen my Sports Doctor before and I'd seen the physio that I ended up rehabbing with, who was fantastic and has done a lot of work with knees.

Because I couldn't get into them at that time to get checked up on. I went to this other physiotherapist. I just trusted that everything that he said, I just thought okay. Well, yeah I don't need to get a scan, he never recommended to do a scan or get anything checked even though it was really swollen, even my mom was like I just feel stupid now because I trusted him as well. I Just thought okay. Well, he's does this every day he must know it was just a medial strain which in the end caused me a lot more damage. So I think if I had have known that and was a bit more aware of the injury I could have made that decision to be like, ah 'I know that it doesn't feel right'. I shouldn't go and play on it. But also it was a lot of self-stupidity in a way like it was swollen. It was painful. I shouldn't have tried to play on it myself. Like now if I have an injury ill rest and be like to my friends, I'll take rest days and things like that, but at that stage of my life, I just wanted to be the best and didn't want to miss out on anything. So it was a lot of, I should of listened to my own body and been like no, it's really sore. I shouldn't have played. It did affect my trust in practitioners because I was like, well it kind of didn't help me in that scenario and it was only one person and I'm sure that they… My knee did present as very stable the whole way through. So I'm sure he probably genuinely did think it was just a medial strain in my knee and my medial was strained, but yeah, it kind of did mentally affect me.

Interviewer: So that initial consultation caused some distress, then that obviously has had an effect throughout your whole Rehabilitation with you needing to check back in and even though you trusted your physio checking, Yeah, you sure?

P9: Yeah and like I have really a really good relationship with him now, but he's just like funny. Saying, ‘Oh, you're a pest sometimes I am just s annoying because I know him so well know because I've seen him for so many years. I always joke Like, am I okay? And he would be like, ‘yes, you're doing more than you have to. Look your fine’. But it was just a lot of self-doubt and I wanted a lot of reassurance from to be like, ‘are you sure’ but now I'm okay, like now I'm fine, but I think getting that initial kind of information scared me a little bit.

Time 49 minutes 03 seconds
