# Supplementary material for: Understanding Fear after an Anterior Cruciate Ligament Injury: A Qualitative Thematic Analysis Using the Common-Sense Model
Source: Int J Environ Res Public Health. 2023 Feb 7;20(4):2920. doi: 10.3390/ijerph20042920 (PMC9957354; doi:10.3390/ijerph20042920)
Supplement: Supplementary file 1 [file ijerph-20-02920-s001.zip › File S2 Interview Schedule.pdf]

Interview Schedule

Interview questions/script are guided by the Common-Sense Model (CSM) with the CSM constructs matched against the questions

General Procedure

Different parts of the interview schedule (or Frames) were guided by specific timeline points of rehabilitation:

For example: Opening Frame was an overview of the ACL injury, Frame 1 was prior to ACL injury and Frame 2 was 3-4 months post ACL injury/surgery. End stage of rehabilitation was 9-12 months and after 12 months was current function.

Questions were determined by the answers given to allow participants to guide the interview process. General probes were used to encourage elaboration on an existing topic.

General probes (determined by the initial response): were things such as

- Can you tell me more about that?
- What do you mean by…?
- Why might that be the case?
- Did you expect to have such changes?

Whilst specific probes were required if topic was moving too far away from the subject matter of fear and emotions. For example, if participants begun discussing their specific physical rehabilitation programs with sets and repetitions, interviewer guided discussion away.

Specific probe examples:

- Can you tell me what that meant to you
- Can you tell me how you felt about that
- Can you elaborate on how that made you feel
- What contributed to those thoughts and emotions

Introduction:

Today you'll be participating in a study that is researching fear post ACL injury, the interviews will consist of a series of open-ended questions that will explore your fear, levels of distress and confidence after your injury, please feel free to say as much or as little as you want on any topic and if you feel uncomfortable at any time let me know and we'll move on.

Are you happy to proceed?

**<u>Common Sense Model Construct</u>**	**Timeline/ Stage**	**Example Question**
**Interpretation & Representation of fear**<br>*Identity*<br>*Cause*<br>*Consequences*<br>*Timeline*<br>*Curability/Control*	Overview	Tell me about your ACL injury.<br><br>In regard to your ACL injury you indicated on the questionnaire that you experienced fear associated with your ACL injury.<br><br>What did this fear mean to you?
	Initial Stage	Prior to your injury, did you have any fear of injuring your knee?<br><br>Initially after your injury, what were your emotions:<br>(Did you experience Fear?<br>Were you distressed by this fear?<br>How would you describe distress?)<br><br>How would you describe the effect this injury had on your confidence?<br>How would you describe confidence?<br><br>At the time of your initial injury, did anyone ask you (health professionals?) about any psychological considerations of your injury?<br><br>How did you manage your knee related: fear &/or distress.<br><br>How did you build your confidence?<br>(did others help you approach the fear)
	Return to general function/ Resumption of normal activities<br><br><br>Time point 3-4 months.	When you began to resume your normal activities, (about 3-4 months) in relation to your knee:<br>Do you believe you had fear? Or distress?<br>How confident in the knee were you at this time?<br><br>At this time, did anyone ask you (health professionals?) about fear or distress?<br><br>How did you approach your fear and distress at this time?<br>How do you feel you improved your confidence at this time?<br>(did others help you approach the fear)

	Return to sport<br><br>Time point 9-24months	As you started to return to sport/more vigorous activities, what were your emotions at the time?<br>(Did you have any issues with fear or distress?<br>How confident were you about returning to sport?)<br><br>At this time, did anyone ask you (health professionals?) about any psychological considerations such as fear or distress?<br><br>How did you approach managing your fear and distress at this time?<br>(did others help you approach the fear)<br><br>How did you improve your confidence?

	Currently	Currently, how fearful are you in regards to your ACL injury?<br><br>How distressing is your knee injury to you at this current point in time?<br><br>How does this knee injury currently affect your confidence?<br><br>Are you presently doing anything to address your psychological knee issues?<br><br>Do you think it your knee function can still improve?<br><br>Can you improve your fear, distress or confidence in relation to your knee?<br><br>How was your mental health during your rehab?
Behavioural response	Currently	Currently, how do you respond when you are faced with a fearful movement or activity?
Emotional response<br>*Emotion*<br>*Coherency*	Currently	Tell me about the effect your knee injury has had on your overall wellbeing?<br><br>How has your perception of yourself changed because of this injury?<br><br>How has the way other people perceive you changed after this knee injury?

Specific Probe (if required at the end of questioning): What is your biggest fear related to Re-Injury?
