# Supplementary material for: Understanding Fear after an Anterior Cruciate Ligament Injury: A Qualitative Thematic Analysis Using the Common-Sense Model
Source: Int J Environ Res Public Health. 2023 Feb 7;20(4):2920. doi: 10.3390/ijerph20042920 (PMC9957354; doi:10.3390/ijerph20042920)
Supplement: Supplementary file 1 [file ijerph-20-02920-s001.zip › File S3 Braun & Clarke 15-point Thematic Analysis Checklist.pdf]

Braun & Clarke 15-point Thematic Analysis Checklist

Process	No.	Criteria	Response
Transcription	1	The data have been transcribed to an appropriate level of detail, and the transcripts have been checked against the tapes for 'accuracy'	Data was transcribed to a high level of detail, and appropriately checked for accuracy.
Coding	2	Each data item has been given equal attention in the coding process	We reviewed all transcripts and gave the data items equal attention in the coding process.
	3	Themes have not been generated from a few vivid examples (an anecdotal approach), but instead the coding process has been thorough, inclusive and comprehensive	Themes were generated through the coding process and was inclusive of all quotes generated from the articles and comprehensive
	4	All relevant extracts for all each theme have been collated	Yes.
	5	Themes have been checked against each other and back to the original data set	Yes.
	6	Themes are internally coherent, consistent, and distinctive	Yes.
Analysis	7	Data have been analysed- interpreted, made sense of- rather than just paraphrased or described	Yes, evident from the results.
	8	Analysis and data match each other- the extracts illustrate the analytic claims.	The analysis and findings closely match the data set.
	9	Analysis tells a convincing and well-organised story about the data and topic	Yes.
	10	A good balance between analytical narrative and illustrative extracts is provided	Yes. Illustrations have been used within the results section.
Overall	11	Enough time has been allocated to complete all phases of the analysis adequately, without rushing a phase or giving it a once-over-lightly	Yes.
Written report	12	The assumptions about, and specific approach to, thematic analysis are clearly explicated	Yes, stated in the methods section.
	13	There is good fit between what you claim you do, and what you show you have done- i.e. described method and reported analysis are consistent	Yes.

	14	The language and concepts used in the report are consistent with the epistemological position of the analysis	Yes.
	15	The researcher is positioned as *active* in the research process; themes do not just 'emerge'	Yes.

REFERENCE:

1. Braun V, Clarke V. Successful qualitative research: a practical guide for beginners. London: SAGE Publications Ltd; 2013.
