# Supplementary material for: Understanding Fear after an Anterior Cruciate Ligament Injury: A Qualitative Thematic Analysis Using the Common-Sense Model
Source: Int J Environ Res Public Health. 2023 Feb 7;20(4):2920. doi: 10.3390/ijerph20042920 (PMC9957354; doi:10.3390/ijerph20042920)
Supplement: Supplementary file 1 [file ijerph-20-02920-s001.zip › File S4 Participant Injury and Sport table.pdf]

Appendix 2

Participant injury and sport:

Participant	Pre-injury Sport(s)	Level of Sport*	Injury occurrence	Post-injury Sport(s) & level	Year(s) of injury	Return to Sport Rating#
P1	Football (Australian rules) and basketball	School	Football competition	Cricket, amateur club level	2016	2
P2	Basketball	National/ International	Basketball competitions	Basketball, amateur club level	2017, 2018	2
P3	Football (soccer)	Amateur/club	Football training	Nil	2016	0
P4	Hockey	Amateur/club	Hockey competition	Hockey at amateur club level	2016	3
P5	Ultimate Frisbee	State	Ultimate Frisbee competition	Ultimate Frisbee at State level	2017	3
P6	Netball, skiing	Amateur/club	Skiing (first), Netball training	Rowing at amateur club level	2011, 2016	2
P7	Cricket, mixed netball	Amateur/club (cricket), social (mixed netball)	Mixed netball game	Cricket at amateur/club level (only)	2017	3
P8	Skiing (moguls)	National/ International	Skiing training	Casual skiing (non-competitive)	2011, 2012	3
P9	Netball and basketball	State	Netball competition	Netball – State level (only)	2015	3
P10	Netball	Amateur/Club	Netball competition	Nil sport (gym for fitness)	2018	1
P11	Netball	Amateur/club	Netball competition	Nil sport (runs, gym for fitness)	2015	1
P12	Basketball, skiing	School, casual	Motorcycle accident	Nil sport, still skis	2008	2
P13	Netball	Amateur/club	Netball competition	Nil sport (gym for fitness)	2008	1
P14	Football (soccer)	Amateur/club	Football competitions	Nil sport (gym for fitness)	2006, 2009, 2013	1

P15	Skiing	Casual	Skiing holiday	Nil sport (gym for fitness)	2019	1
P16	Ultimate Frisbee	National/ International	Ultimate Frisbee competition	Ultimate Frisbee at amateur/club level	2019	2
P17	Tennis, netball	School	Accident at school	Nil sport (gym, running for fitness)	2006	1
P18	Lacrosse	Amateur/club	Lacrosse competition	Nil sport (bicycle, gym, hiking for fitness)	2019	1

* Level of sport was identified as either: 'social' or 'casual' (non- competitive), 'school' (playing in inter-school competitions), 'amateur/club' (amateur clubs playing in a local league), 'State' (eligible to represent an Australian State such as Victoria or NSW), or 'National/International' (eligible for inclusion in a national level team with opportunities to play internationally).

# Return to previous level of function scoring: 0-3 where 0= no return to any exercise, 1= Return to basic exercise (gym etc.) but no or minimal sport (i.e. non-competitive), 2= Return to sport at a lower competition level, 3= Return to previous level of sport.
