# Supplementary material for: Understanding Fear after an Anterior Cruciate Ligament Injury: A Qualitative Thematic Analysis Using the Common-Sense Model
Source: Int J Environ Res Public Health. 2023 Feb 7;20(4):2920. doi: 10.3390/ijerph20042920 (PMC9957354; doi:10.3390/ijerph20042920)
Supplement: Supplementary file 1 [file ijerph-20-02920-s001.zip › File S5 Reflection.pdf]

Reflection: Pre and Post study.

On commencement of this study in 2020, I had some beliefs about ACL injuries gained from my own personal experiences and training as a Physiotherapist. I wrote a reflection at the commencement of this project which I have paraphrased below.

An ACL is considered a musculoskeletal injury, which means it is predominantly biomechanical. The ACL has an important role in stabilising the knee - losing the ligament must therefore de-stabilise the knee and cause the sequalae of issues and poor return to sport rates, right? This theory made academic sense and is the primary supported notion within healthcare and the literature. This is supported by the high surgery rates and what I was taught in Physiotherapy, with stage-wise ACL rehabilitation protocols that have objective physical markers for progressions. My best guess, as to why even those with good objective functional measures were still not participating in their previous level of sport, was perhaps changing life circumstances such as having a child, new job etc. Maybe they were done with sport? I did however feel that this was not a satisfactory explanation for why 'sporty people' would give up something so important to them.

For a back story on myself, I have suffered four ACL ruptures and three ACL reconstructions. I found it challenging to question why I had not gone back to sport. I was diligent in rehabilitation and had completed my objective functional measures within acceptable side to side metrics. However, when I was presented with the opportunity to play any sport that involved cutting or suddenly stopping, I immediately stayed away. This aversion to anything risky, to 'protect' my knee had a significant impact on my life, dictating not just sports but also social activities and my day to day. Getting out of a car, walking on an uneven path, even minor things such as these would occasionally lead to intrusive thoughts about my knee, and the need to be careful.

I suspected there was a lot more significant effects of an ACL injury that has not been explored. This was confirmed when during a lecture at university I was shown a series of ACL injury videos. I remember vividly the effect these videos had on me. Feeling ill, nauseous and completely flat, I was in fact 'written off' for a significant portion of the day.

I expected through commencement of this research to find some level of psychological drivers for fear and dysfunction. I expected this would likely correlate with trauma, pain or biomechanical issues.

How my perspective has changed.

The results surprised me in the polarity that the ongoing fear and dysfunction drivers were overwhelmingly social and psychological, *not* physical. The commonly managed physical issues which are key to a 'successful' ACL rehabilitation, such as, pain, weakness, range of motion, objective functional measures appeared not important and hardly mentioned (with the exception of a traumatised few who vividly remembered their injury and pain). Rather, the primary drivers seemed to be around the social and psychological effects, particularly around rehabilitation and the effects of the injury to an ACL injured individuals quality of life.

On reflection I can see a clear disconnect between the current management practices for ACL injury and what these individuals report is driving their fear. If we follow in the footsteps of the chronic low back pain literature to explore bio-psycho-social drivers, we could improve the ACL injury management process, de-threaten it, provide appropriate support throughout the rehabilitation process and better educate ACL injured individuals. Perhaps individual, quality of life outcomes would be significantly better.
